# Supplementary material for: Ribosome Pausing Negatively Regulates Protein Translation in Maize Seedlings during Dark-to-Light Transitions
Source: Int J Mol Sci. 2024 Jul 22;25(14):7985. doi: 10.3390/ijms25147985 (PMC11277263; doi:10.3390/ijms25147985)
Supplement: Supplementary file 1 [file ijms-25-07985-s001.zip › FigureS1.pdf]

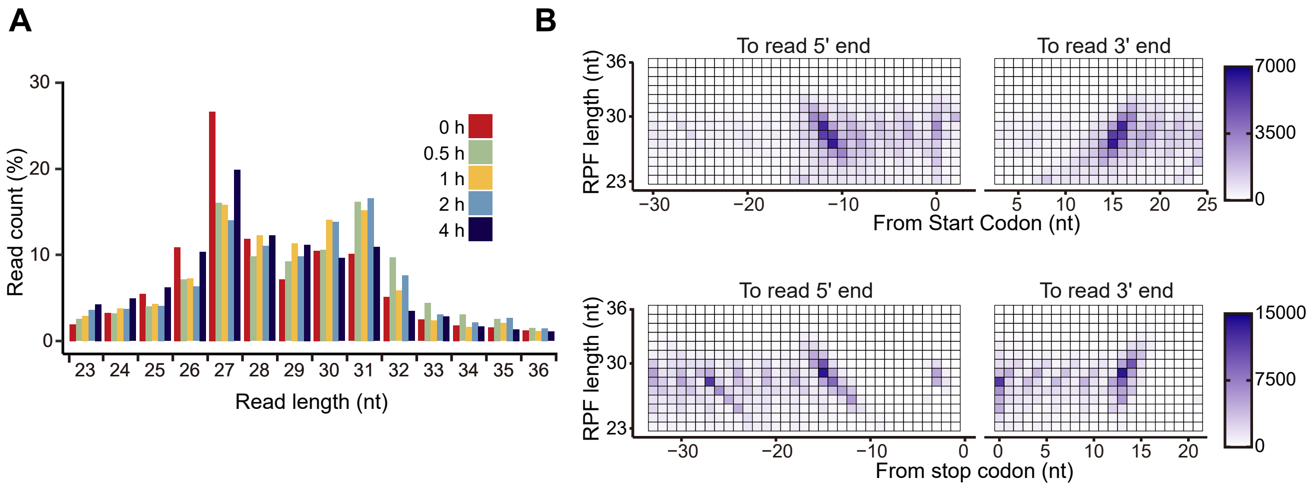

Figure S1 Length distributions of ribosome-protected fragments
A. Distribution of RPF length from Ribo-seq libraries at each time point. **B.** Meta-analysis of RPFs that map to the start (top) or stop (bottom) codons. The data from the 15 datasets (five time points and three replicates) were parsed as footprints.
