# Supplementary material for: Ribosome Pausing Negatively Regulates Protein Translation in Maize Seedlings during Dark-to-Light Transitions
Source: Int J Mol Sci. 2024 Jul 22;25(14):7985. doi: 10.3390/ijms25147985 (PMC11277263; doi:10.3390/ijms25147985)
Supplement: Supplementary file 1 [file ijms-25-07985-s001.zip › FigureS10.pdf]

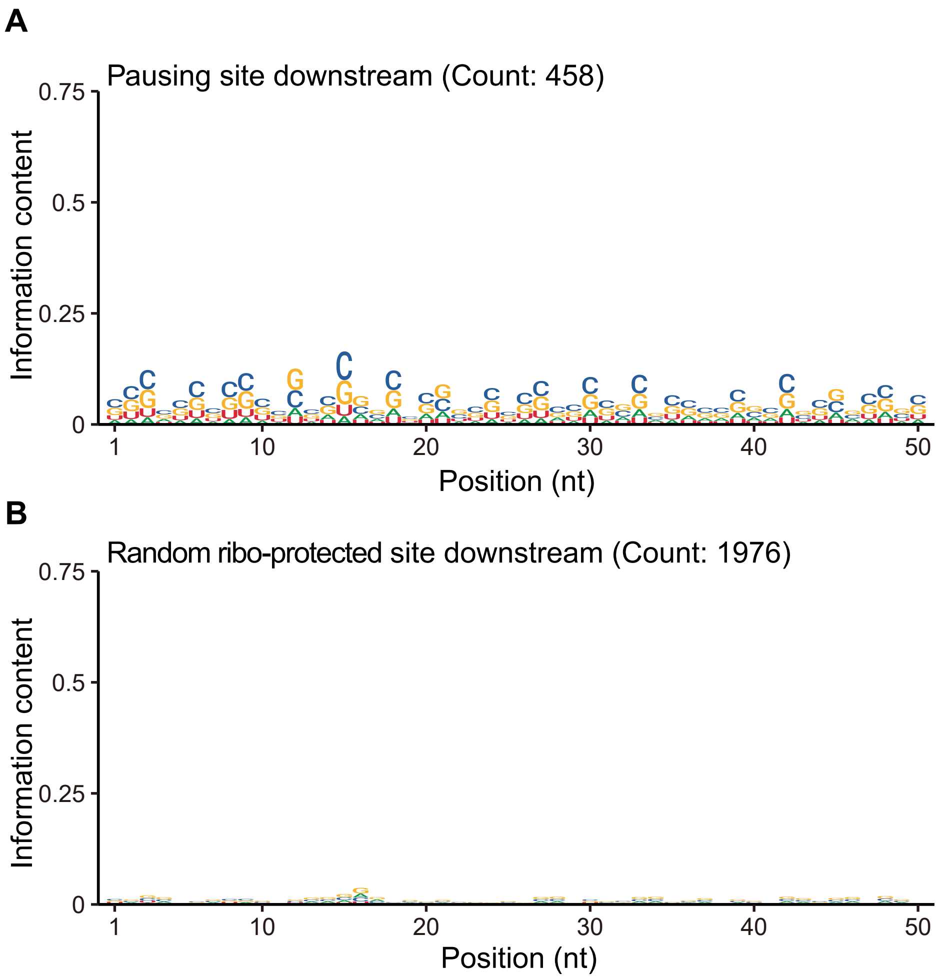


**Figure S10 Characteristics of the sequences downstream of the ribosome-pausing sites**
**A.** Sequence logo of the nucleotide sequences downstream of the pausing sites. **B.** Sequence logo of the nucleotide sequences downstream of randomly chosen RPFs without ribosome pausing. The height of each letter indicates their probability at that position. The numbers along the x-axis refer to the distance to the ribosome-pausing sites. The +1 position indicates the downstream 1- nt of the ribosome paused site.
