# Supplementary material for: Ribosome Pausing Negatively Regulates Protein Translation in Maize Seedlings during Dark-to-Light Transitions
Source: Int J Mol Sci. 2024 Jul 22;25(14):7985. doi: 10.3390/ijms25147985 (PMC11277263; doi:10.3390/ijms25147985)
Supplement: Supplementary file 1 [file ijms-25-07985-s001.zip › FigureS11.pdf]

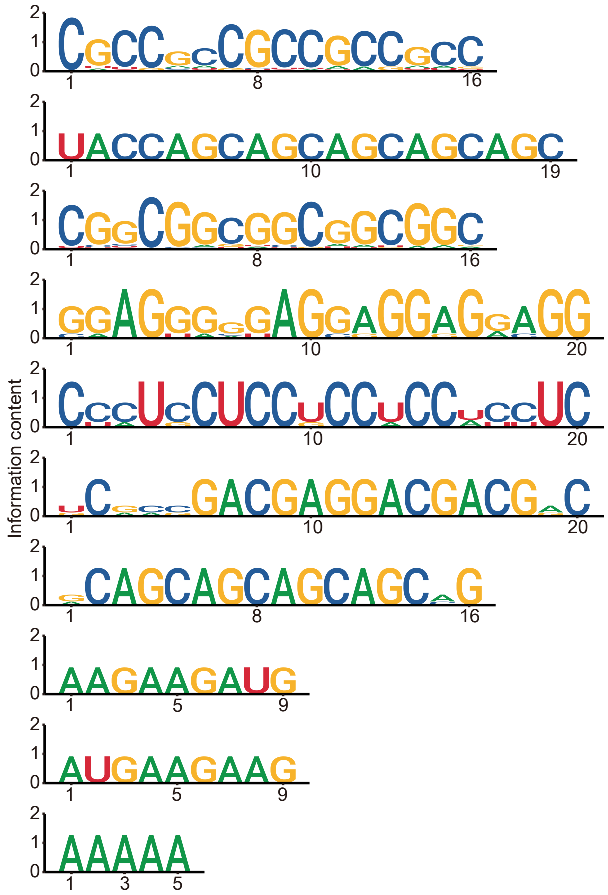

Motif sequence	*P* value	Sequence count	Motif length
CGCCGCCGCCGCCGCC	2.30E-05	61	16
TACCAGCAGCAGCAGCAGC	5.70E+00	2	19
CGGCGGCGGCGGCGGC	1.60E+01	43	16
GGAGGGGGAGGAGGAGGAGG	5.80E+04	7	20
CCCTCCTCCTCCTCCTCCTC	7.10E+04	5	20
TCGCCGACGAGGACGACGAC	6.10E+05	3	20
GCAGCAGCAGCAGCAG	9.20E+05	3	16
AAGAAGATG	4.50E+06	2	9
ATGAAGAAG	4.50E+06	2	9
AAAAA	1.90E+07	3	5

Figure S11 Possible motifs in the 3′ region of ribosome-pausing sites

Sequence logos showing possible motifs in the region downstream of ribosome-pausing sites. The height of each letter indicates the probability of occurrence. The numbers along the x-axis refer to the length of the motifs. E-value, expectant value, statistical significance of the motif.
