# Supplementary material for: Ribosome Pausing Negatively Regulates Protein Translation in Maize Seedlings during Dark-to-Light Transitions
Source: Int J Mol Sci. 2024 Jul 22;25(14):7985. doi: 10.3390/ijms25147985 (PMC11277263; doi:10.3390/ijms25147985)
Supplement: Supplementary file 1 [file ijms-25-07985-s001.zip › FigureS2.pdf]

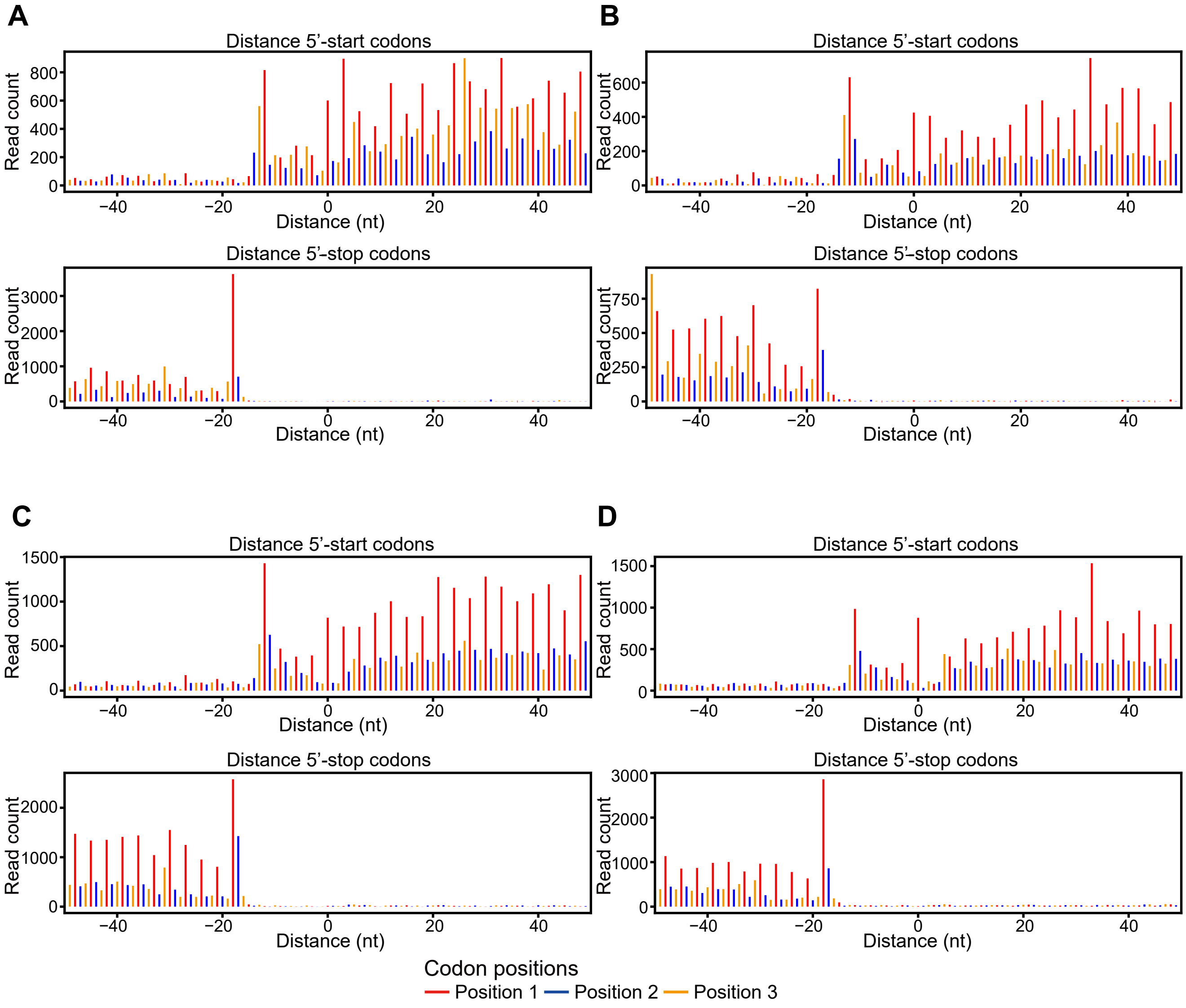

Figure S2 3-nt Periodicity and distribution of RPFs on transcripts in different samples
A–D Number of reads centered at each position along transcripts for the RPFs identified at the 0-h (A), 0.5-h (B), 1-h (C), and 4-h (D) time points. The numbers along the x-axis indicate the distance from the first nucleotide of the RPF to the start or stop codons. The three open reading frames are represented in different colors.
