# Supplementary material for: Ribosome Pausing Negatively Regulates Protein Translation in Maize Seedlings during Dark-to-Light Transitions
Source: Int J Mol Sci. 2024 Jul 22;25(14):7985. doi: 10.3390/ijms25147985 (PMC11277263; doi:10.3390/ijms25147985)
Supplement: Supplementary file 1 [file ijms-25-07985-s001.zip › FigureS3.pdf]

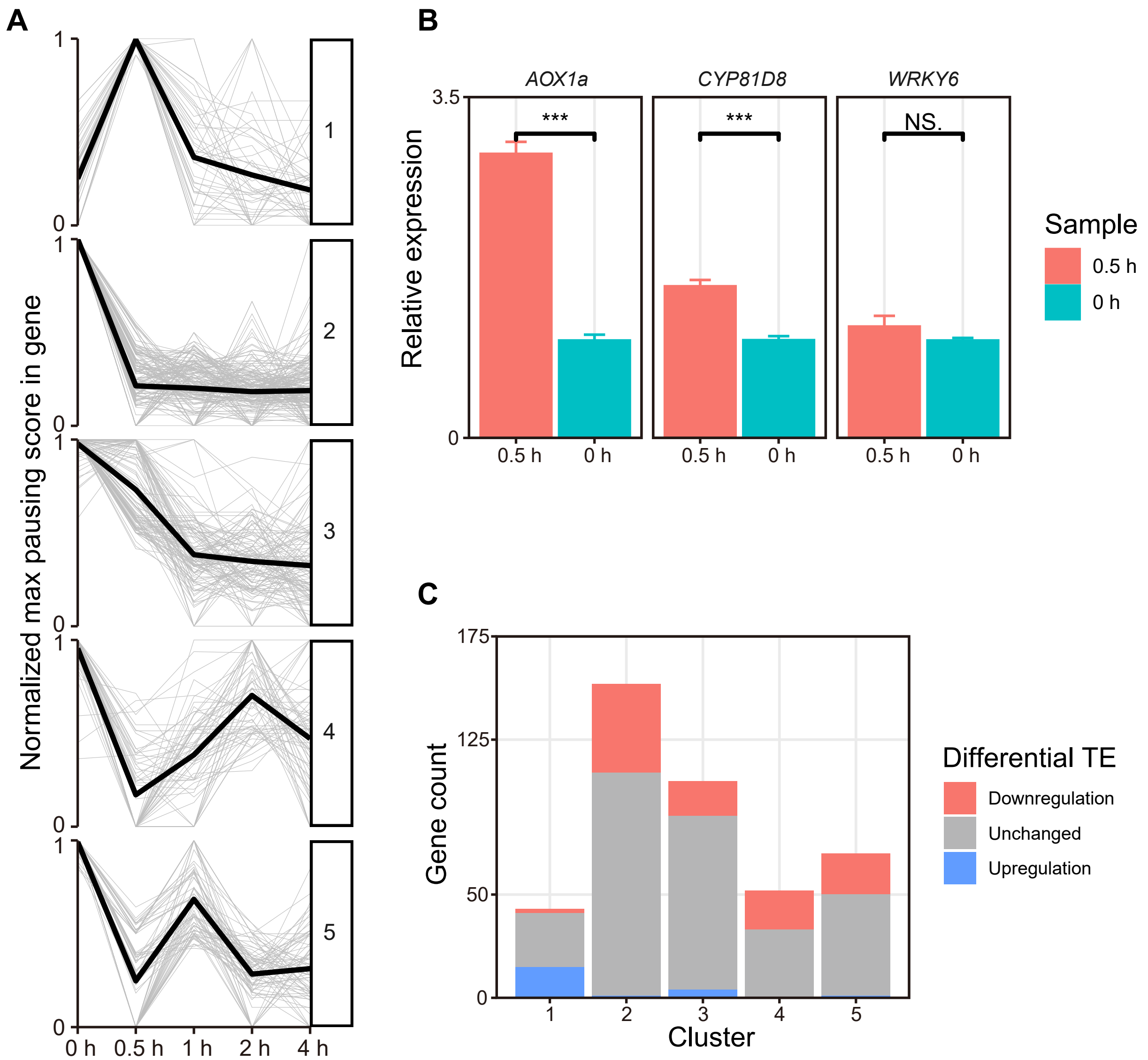

Figure S3 Transcripts with ribosome pausing define five clusters

A. Clustering analysis of ribosome pausing scores defining five distinct clusters with different patterns of pausing scores as a function of duration of light exposure. Transcripts with similar trends in their pausing score pattern (gray) were classified into one cluster; a regression curve (black) was fitted to the maximus pausing score at each time point and cluster. **B.** Relative expression levels of genes involved in reactive oxygen species (ROS), acting as markers for photomorphogenesis, as determined by RT-qPCR. For each gene, the relative expression at the 0-h time point was normalized to 1.0. Three biological replicates were performed for each gene. **C.** Number of transcripts with differential translation efficiency (TE) for each of the five clusters.
