# Supplementary material for: Ribosome Pausing Negatively Regulates Protein Translation in Maize Seedlings during Dark-to-Light Transitions
Source: Int J Mol Sci. 2024 Jul 22;25(14):7985. doi: 10.3390/ijms25147985 (PMC11277263; doi:10.3390/ijms25147985)
Supplement: Supplementary file 1 [file ijms-25-07985-s001.zip › FigureS4.pdf]

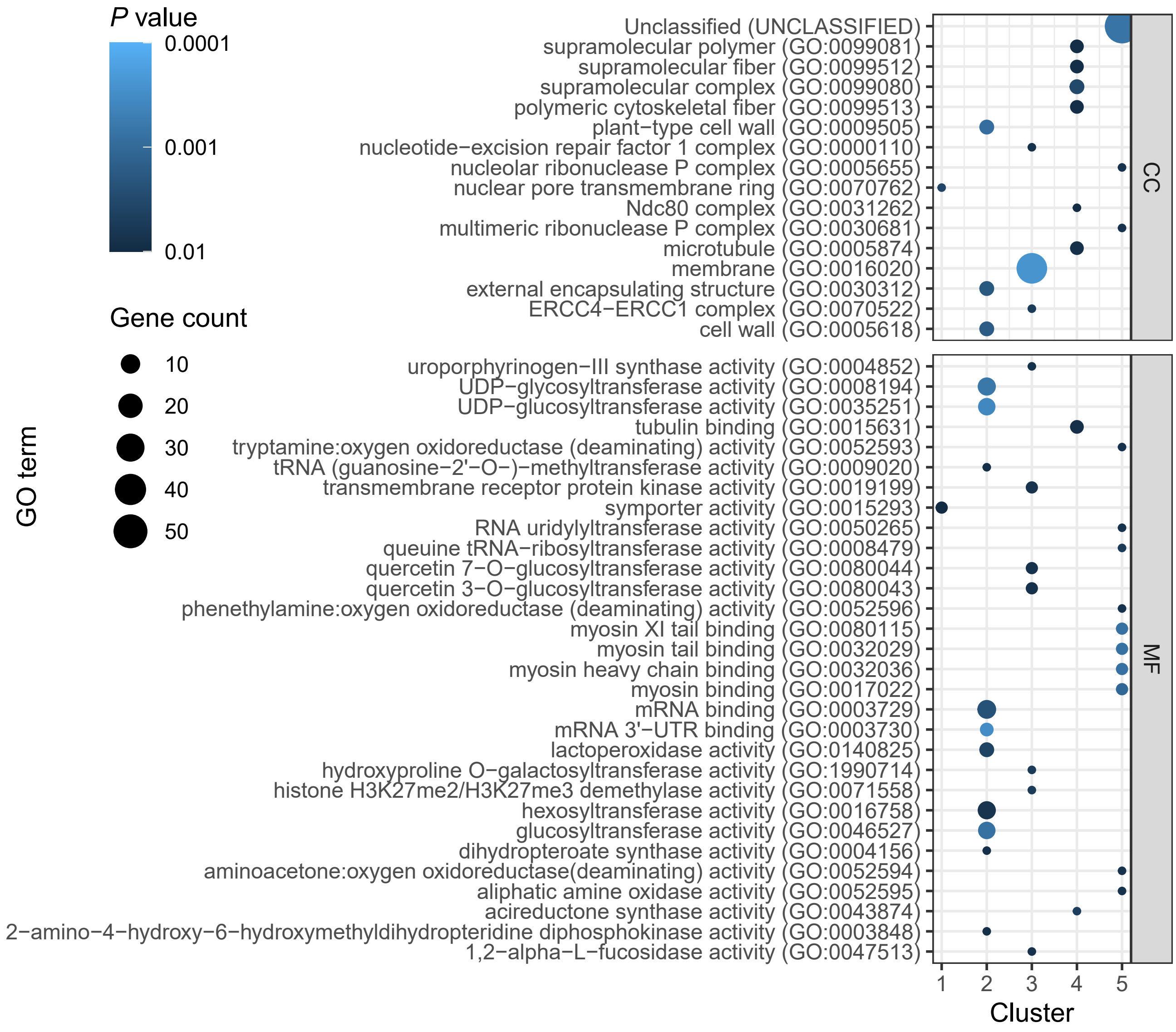


**Figure S4  Gene Ontology term enrichment analysis of the transcripts with ribosome pausing**
CC, cellular component; MF, molecular function. The size of the circle indicates the number of transcripts; the color of the circle indicates the *P* value.
