# Supplementary material for: Ribosome Pausing Negatively Regulates Protein Translation in Maize Seedlings during Dark-to-Light Transitions
Source: Int J Mol Sci. 2024 Jul 22;25(14):7985. doi: 10.3390/ijms25147985 (PMC11277263; doi:10.3390/ijms25147985)
Supplement: Supplementary file 1 [file ijms-25-07985-s001.zip › FigureS5.pdf]

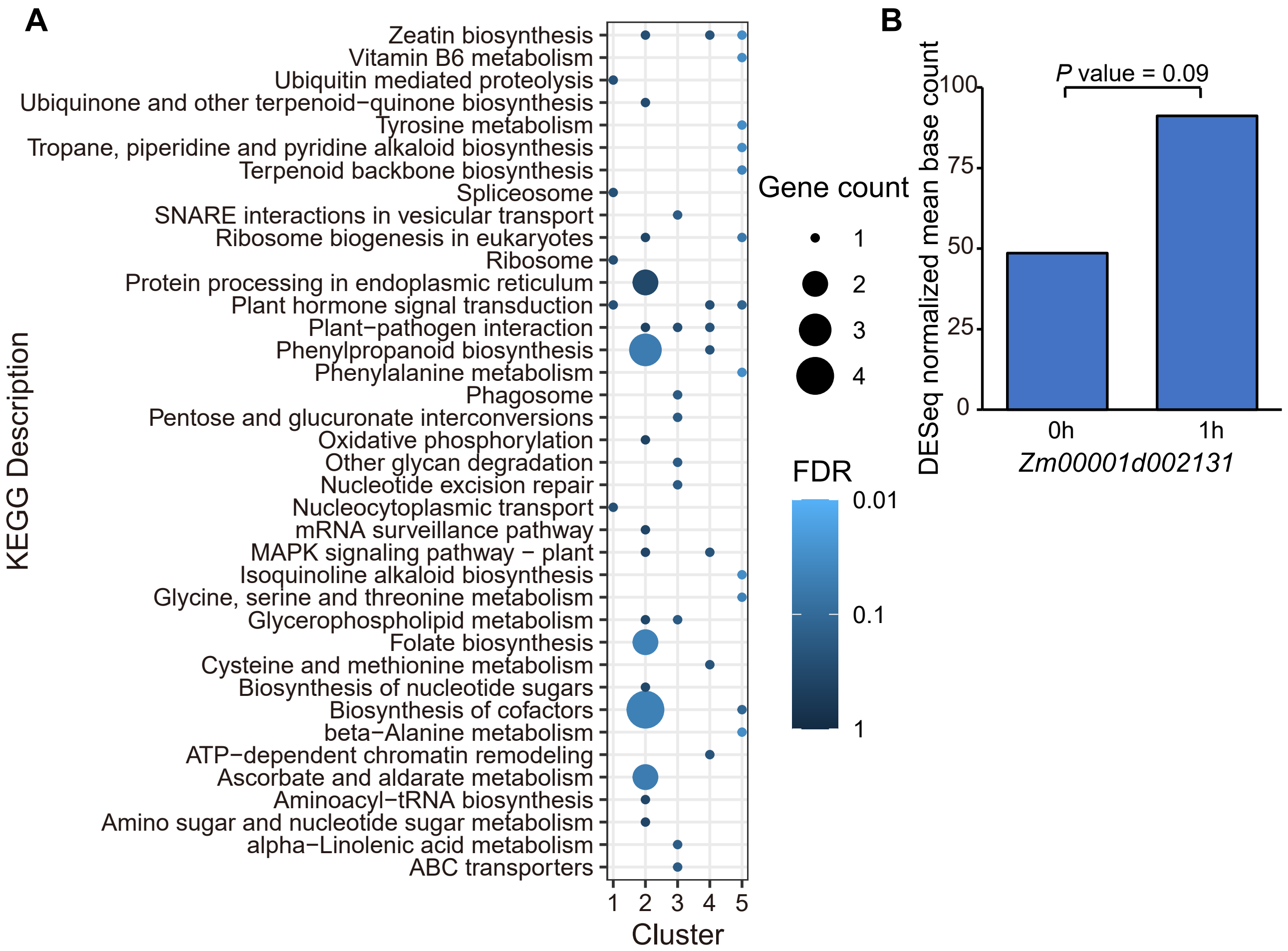

Figure S5 Kyoto Encyclopedia of Genes and Genomes pathway enrichment analysis of transcripts with ribosome pausing

A. Kyoto Encyclopedia of Genes and Genomes pathway analysis on the genes whose transcript belonged to one of the five cluster of ribosome-paused transcripts. The size of the circle indicates the number of transcripts; the color of the circle indicates the false discovery rate (FDR). B. Normalized expression of *Zm00001d002131* at the 0- and 1-h time points, as measured by RNA-seq.
