# Supplementary material for: Ribosome Pausing Negatively Regulates Protein Translation in Maize Seedlings during Dark-to-Light Transitions
Source: Int J Mol Sci. 2024 Jul 22;25(14):7985. doi: 10.3390/ijms25147985 (PMC11277263; doi:10.3390/ijms25147985)
Supplement: Supplementary file 1 [file ijms-25-07985-s001.zip › FigureS6.pdf]

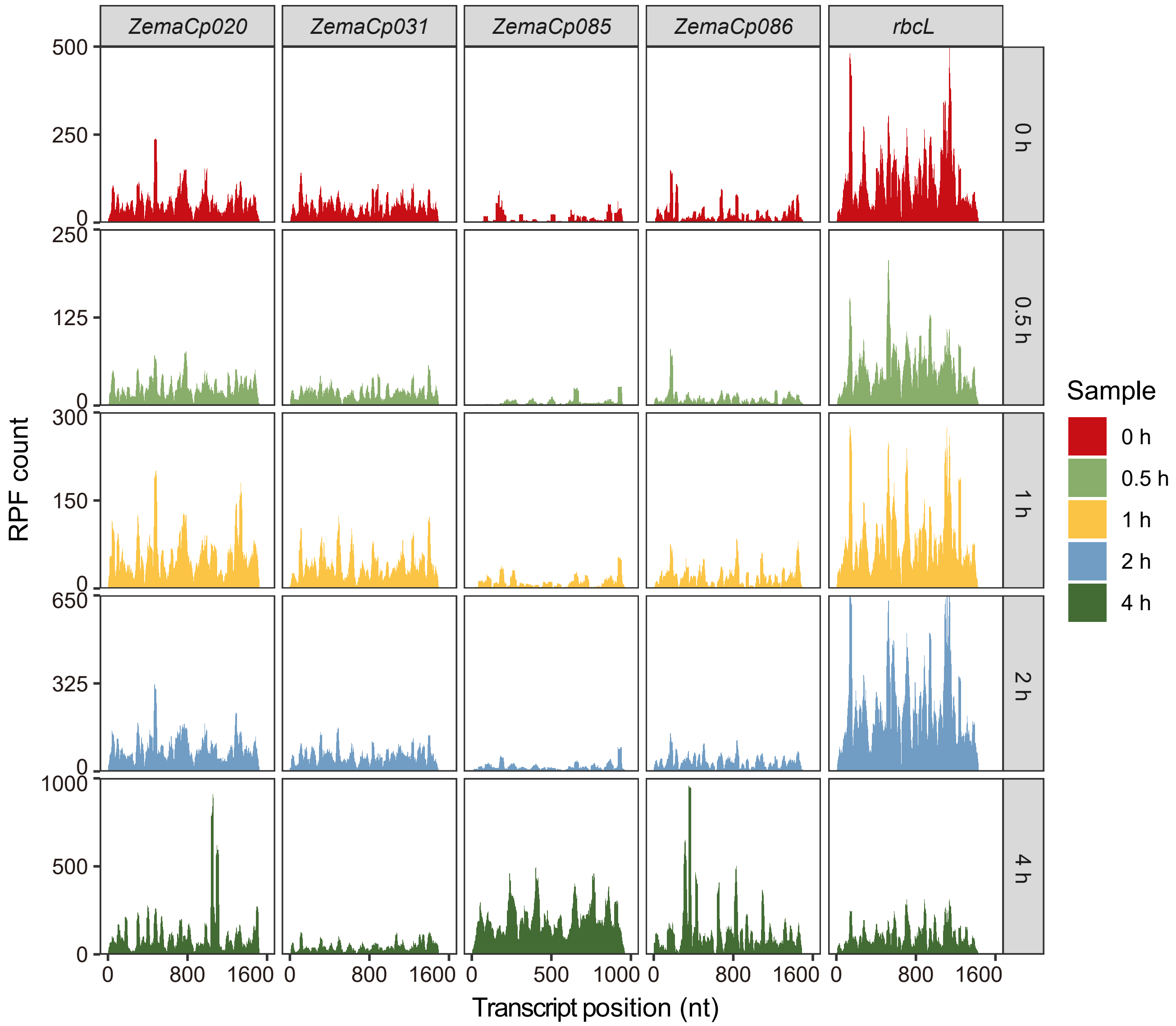

Figure S6 Examples of ribosome-paused chloroplast transcripts
Distribution of RPF counts along five chloroplast ribosome-paused transcripts. *Ribulose-1,5-bisphosphate carboxylase/oxygenase large subunit* (*rbcL*) is a non-pausing control. The colors indicate different time points.
