# Supplementary material for: Ribosome Pausing Negatively Regulates Protein Translation in Maize Seedlings during Dark-to-Light Transitions
Source: Int J Mol Sci. 2024 Jul 22;25(14):7985. doi: 10.3390/ijms25147985 (PMC11277263; doi:10.3390/ijms25147985)
Supplement: Supplementary file 1 [file ijms-25-07985-s001.zip › FigureS7.pdf]

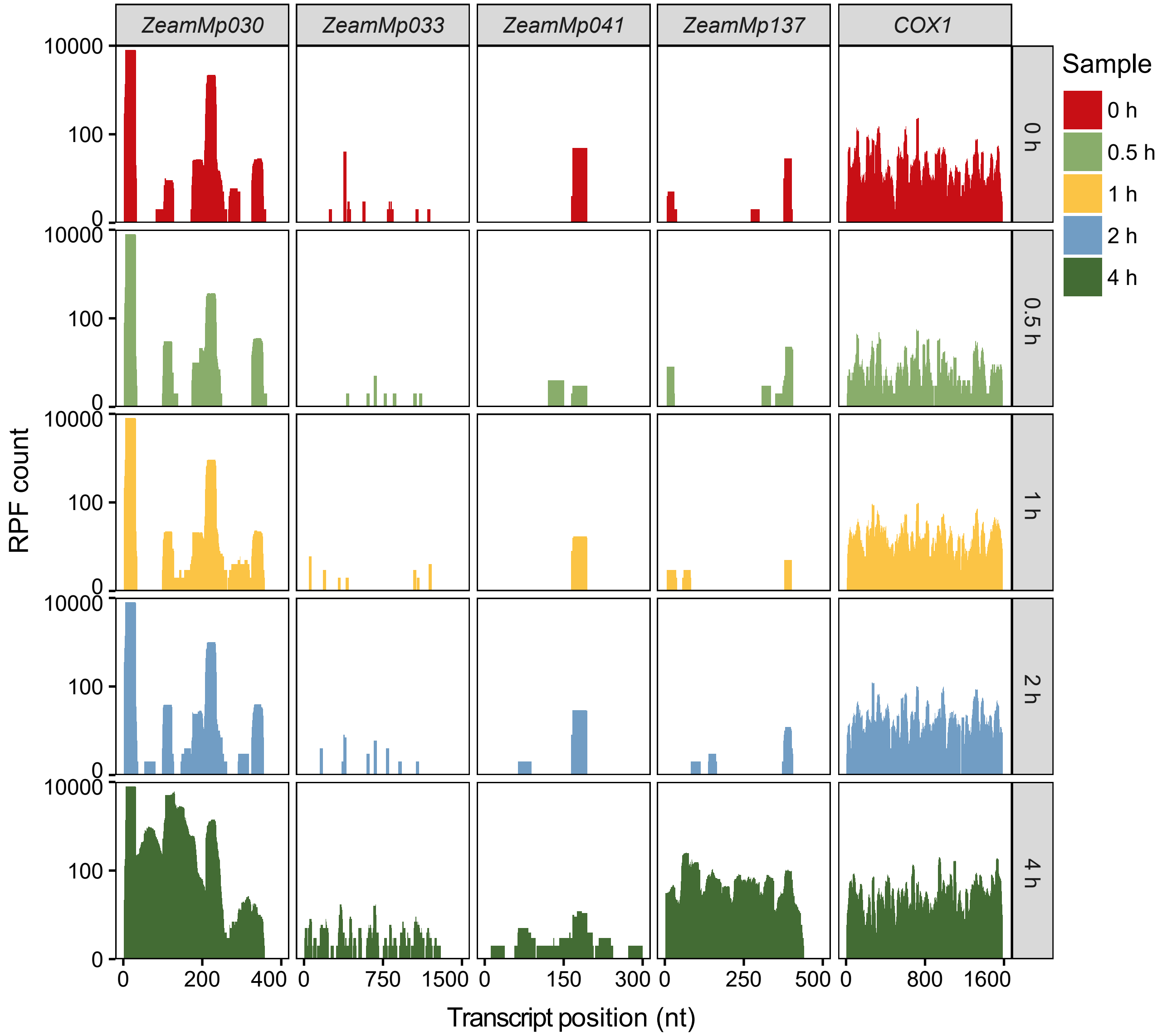

Figure S7 Examples of ribosome-paused mitochondrion transcripts
Distribution of RPF counts along five mitochondrion ribosome-paused transcripts. *Cytochrome C oxidase subunit 1* (*cox1*) is a non-paused control. The colors indicate different time points.
