# Supplementary material for: Ribosome Pausing Negatively Regulates Protein Translation in Maize Seedlings during Dark-to-Light Transitions
Source: Int J Mol Sci. 2024 Jul 22;25(14):7985. doi: 10.3390/ijms25147985 (PMC11277263; doi:10.3390/ijms25147985)
Supplement: Supplementary file 1 [file ijms-25-07985-s001.zip › FigureS8.pdf]

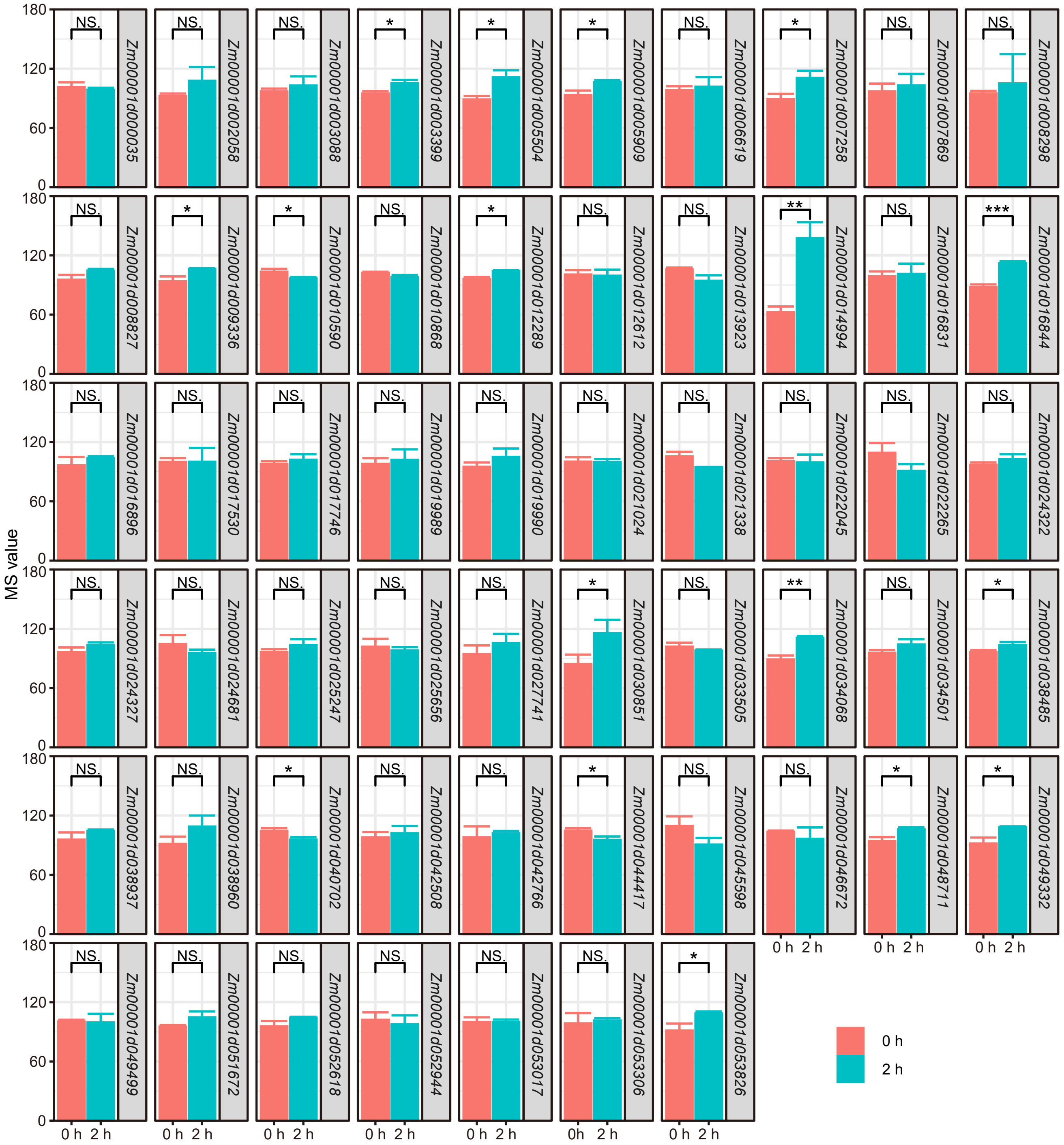

Figure S8 TMT labeling proteomics analysis of proteins encoded by transcripts with ribosome pausing

Protein abundance encoded by transcripts with ribosome pausing, based on mass spectrometry analysis, is shown for the 0- and 2-h time points.
