# Supplementary material for: Ribosome Pausing Negatively Regulates Protein Translation in Maize Seedlings during Dark-to-Light Transitions
Source: Int J Mol Sci. 2024 Jul 22;25(14):7985. doi: 10.3390/ijms25147985 (PMC11277263; doi:10.3390/ijms25147985)
Supplement: Supplementary file 1 [file ijms-25-07985-s001.zip › FigureS9.pdf]

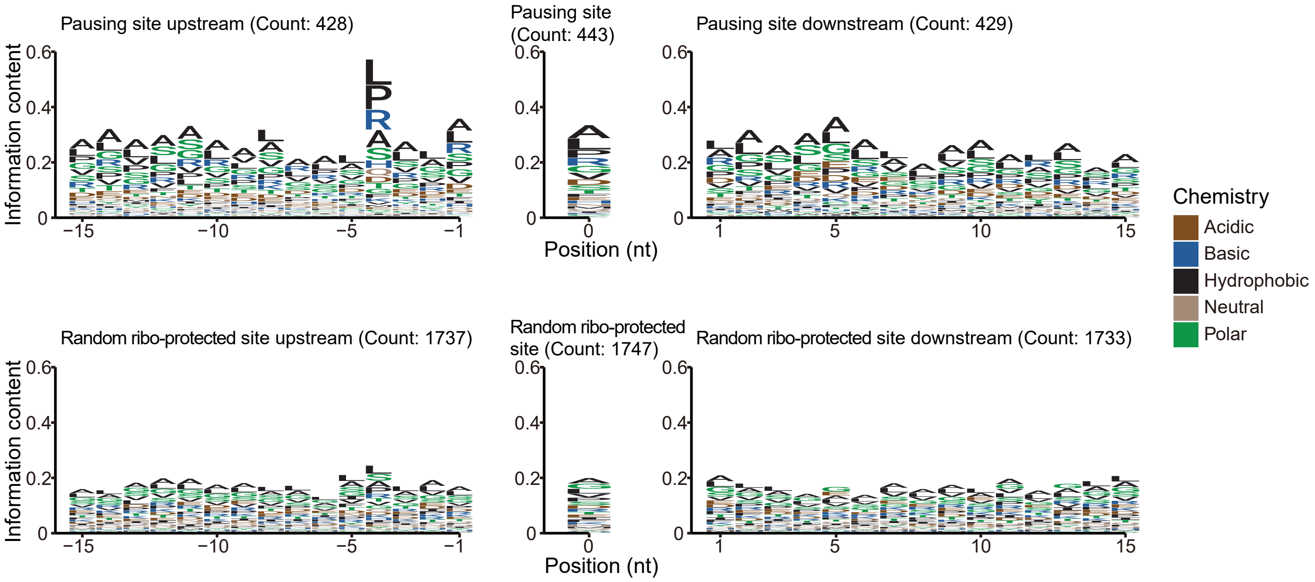

Figure S9 Features of amino acid residues encoded by the fragments around the ribosome-pausing sites

Sequence logo showing the amino acid residues near ribosome-pausing sites in various pausing site contexts. Upper panels, statistics from paused transcripts; lower panels, statistics from randomly chosen transcripts. The properties of amino acid residues are shown in different colors: acidic, red; basic, blue; hydrophobic, black; neutral, purple; polar, green. The height of each letter indicates the probability at that position. The numbers along the x-axis refer to the distance to the codons that underwent ribosome pausing.
