# Supplementary material for: Ribosome Pausing Negatively Regulates Protein Translation in Maize Seedlings during Dark-to-Light Transitions
Source: Int J Mol Sci. 2024 Jul 22;25(14):7985. doi: 10.3390/ijms25147985 (PMC11277263; doi:10.3390/ijms25147985)
Supplement: Supplementary file 1 [file ijms-25-07985-s001.zip › Table S1.pdf]

Table S1 Statistics of the Ribo-seq raw data and filtering results

Sample	Raw reads	23-36nt clean	Rm rRNA
B73-0h-1	56,415,269	39,667,823	5,670,620
B73-0h-2	73,316,731	67,546,829	5,855,543
B73-0h-3	30,338,709	22,458,596	2,181,717
B73-0.5h-1	28,091,621	16,864,985	3,470,310
B73-0.5h-2	54,398,534	47,277,098	3,381,581
B73-0.5h-3	16,310,515	11,342,033	1,092,438
B73-1h-1	41,036,537	27,638,709	4,044,049
B73-1h-2	62,259,406	53,709,865	4,499,810
B73-1h-3	15,408,708	11,220,511	1,128,603
B73-2h-1	73,367,862	43,512,789	6,795,028
B73-2h-2	68,173,386	55,182,889	6,284,671
B73-2h-3	12,389,556	8,895,740	1,049,712
B73-4h-1	27,370,759	18,070,252	5,352,387
B73-4h-2	19,958,180	17,284,270	1,494,560
B73-4h-3	19,073,102	17,009,908	1,844,178

Note : Rm rRNA means 23-36nt reads and through bioinformatics methods removing rRNA.
