# Supplementary material for: Ribosome Pausing Negatively Regulates Protein Translation in Maize Seedlings during Dark-to-Light Transitions
Source: Int J Mol Sci. 2024 Jul 22;25(14):7985. doi: 10.3390/ijms25147985 (PMC11277263; doi:10.3390/ijms25147985)
Supplement: Supplementary file 1 [file ijms-25-07985-s001.zip › Table S2.pdf]

Table S2 Five clusters of ribosome paused transcripts

Transcripts	Cluster	Sample	Normalized max pausing score
ZeamMp030	3	0h	0.578
ZeamMp033	5	0h	1.000
ZeamMp034	4	0h	0.450
ZeamMp041	4	0h	1.000
ZeamMp137	3	0h	1.000
Zm00001d000035	3	0h	1.000
Zm00001d000390	2	0h	1.000
Zm00001d002058	5	0h	1.000
Zm00001d002086	1	0h	0.417
Zm00001d002131	2	0h	1.000
Zm00001d002542	3	0h	1.000
Zm00001d002684	1	0h	0.488
Zm00001d002757	3	0h	1.000
Zm00001d002782	3	0h	1.000
Zm00001d002899	2	0h	1.000
Zm00001d003088	5	0h	1.000
Zm00001d003183	1	0h	0.000
Zm00001d003281	5	0h	1.000
Zm00001d003399	3	0h	1.000
Zm00001d003400	4	0h	1.000
Zm00001d003427	2	0h	1.000
Zm00001d003435	5	0h	1.000
Zm00001d003463	2	0h	1.000
Zm00001d003516	4	0h	1.000
Zm00001d003538	2	0h	1.000
Zm00001d003593	2	0h	1.000
Zm00001d003743	2	0h	1.000
Zm00001d003763	4	0h	1.000
Zm00001d004301	1	0h	0.352
Zm00001d004310	1	0h	0.348
Zm00001d004910	4	0h	1.000
Zm00001d005109	5	0h	1.000
Zm00001d005480	3	0h	0.995
Zm00001d005504	3	0h	0.829
Zm00001d005612	2	0h	1.000
Zm00001d005680	4	0h	1.000
Zm00001d005909	3	0h	1.000
Zm00001d005936	2	0h	1.000
Zm00001d005962	2	0h	1.000
Zm00001d005989	3	0h	1.000
Zm00001d006000	2	0h	1.000
Zm00001d006011	3	0h	1.000
Zm00001d006045	3	0h	1.000
Zm00001d006132	3	0h	1.000
Zm00001d006193	4	0h	1.000
Zm00001d006321	2	0h	1.000
Zm00001d006619	3	0h	1.000
Zm00001d006631	1	0h	0.250
Zm00001d006638	5	0h	0.875
Zm00001d006894	5	0h	1.000

Transcripts	Cluster	Sample	Normalized max pausing score
Zm00001d006947	4	0h	1.000
Zm00001d006950	1	0h	0.339
Zm00001d007015	1	0h	0.188
Zm00001d007050	2	0h	1.000
Zm00001d007162	3	0h	1.000
Zm00001d007197	2	0h	1.000
Zm00001d007258	2	0h	1.000
Zm00001d007259	5	0h	1.000
Zm00001d007294	3	0h	1.000
Zm00001d007478	4	0h	1.000
Zm00001d007503	5	0h	1.000
Zm00001d007518	1	0h	0.000
Zm00001d007606	4	0h	1.000
Zm00001d007839	2	0h	1.000
Zm00001d007869	2	0h	1.000
Zm00001d008187	4	0h	1.000
Zm00001d008219	2	0h	1.000
Zm00001d008297	2	0h	1.000
Zm00001d008298	5	0h	1.000
Zm00001d008329	2	0h	1.000
Zm00001d008409	5	0h	1.000
Zm00001d008764	2	0h	1.000
Zm00001d008827	3	0h	1.000
Zm00001d008859	3	0h	1.000
Zm00001d009008	4	0h	1.000
Zm00001d009108	2	0h	1.000
Zm00001d009138	4	0h	0.938
Zm00001d009336	3	0h	0.926
Zm00001d009568	2	0h	1.000
Zm00001d009747	4	0h	1.000
Zm00001d009787	2	0h	1.000
Zm00001d010044	2	0h	1.000
Zm00001d010222	3	0h	0.917
Zm00001d010325	2	0h	1.000
Zm00001d010388	3	0h	1.000
Zm00001d010564	2	0h	1.000
Zm00001d010590	2	0h	1.000
Zm00001d010594	2	0h	1.000
Zm00001d010610	3	0h	1.000
Zm00001d010621	2	0h	1.000
Zm00001d010785	5	0h	0.920
Zm00001d010788	3	0h	1.000
Zm00001d010868	2	0h	1.000
Zm00001d010872	2	0h	1.000
Zm00001d010925	2	0h	1.000
Zm00001d011068	4	0h	1.000
Zm00001d011620	1	0h	0.000
Zm00001d011881	3	0h	1.000
Zm00001d011890	5	0h	1.000
Zm00001d011964	1	0h	0.172
Zm00001d012041	2	0h	1.000

Transcripts	Cluster	Sample	Normalized max pausing score
Zm00001d012237	2	0h	1.000
Zm00001d012275	3	0h	1.000
Zm00001d012289	2	0h	1.000
Zm00001d012387	1	0h	0.000
Zm00001d012612	2	0h	1.000
Zm00001d012626	2	0h	1.000
Zm00001d012785	2	0h	1.000
Zm00001d012934	5	0h	1.000
Zm00001d013069	1	0h	0.369
Zm00001d013162	2	0h	1.000
Zm00001d013311	4	0h	1.000
Zm00001d013339	5	0h	1.000
Zm00001d013342	2	0h	1.000
Zm00001d013399	2	0h	1.000
Zm00001d013794	3	0h	0.973
Zm00001d013923	2	0h	1.000
Zm00001d014196	4	0h	1.000
Zm00001d014253	1	0h	0.000
Zm00001d014414	5	0h	1.000
Zm00001d014463	2	0h	1.000
Zm00001d014704	2	0h	1.000
Zm00001d014820	2	0h	1.000
Zm00001d014994	2	0h	1.000
Zm00001d015059	5	0h	1.000
Zm00001d015129	3	0h	1.000
Zm00001d015202	2	0h	1.000
Zm00001d015215	2	0h	1.000
Zm00001d015407	4	0h	1.000
Zm00001d015412	2	0h	1.000
Zm00001d015744	1	0h	0.400
Zm00001d015779	4	0h	0.364
Zm00001d015884	3	0h	1.000
Zm00001d015990	4	0h	1.000
Zm00001d016154	5	0h	1.000
Zm00001d016262	2	0h	1.000
Zm00001d016301	5	0h	1.000
Zm00001d016322	3	0h	1.000
Zm00001d016417	2	0h	1.000
Zm00001d016648	5	0h	1.000
Zm00001d016806	5	0h	1.000
Zm00001d016831	4	0h	1.000
Zm00001d016844	5	0h	1.000
Zm00001d016896	3	0h	1.000
Zm00001d017351	3	0h	1.000
Zm00001d017353	1	0h	0.183
Zm00001d017462	4	0h	1.000
Zm00001d017530	2	0h	1.000
Zm00001d017696	2	0h	1.000
Zm00001d017746	2	0h	1.000
Zm00001d017851	3	0h	0.974
Zm00001d017991	2	0h	1.000

Transcripts	Cluster	Sample	Normalized max pausing score
Zm00001d018058	3	0h	1.000
Zm00001d018081	2	0h	1.000
Zm00001d018117	1	0h	0.000
Zm00001d018133	4	0h	0.837
Zm00001d018191	1	0h	0.346
Zm00001d018475	4	0h	1.000
Zm00001d018696	3	0h	1.000
Zm00001d018806	2	0h	1.000
Zm00001d019002	2	0h	1.000
Zm00001d019400	3	0h	1.000
Zm00001d019422	5	0h	1.000
Zm00001d019582	5	0h	1.000
Zm00001d019669	1	0h	0.600
Zm00001d019925	5	0h	0.955
Zm00001d019989	3	0h	1.000
Zm00001d019990	2	0h	1.000
Zm00001d020176	5	0h	1.000
Zm00001d020277	4	0h	0.594
Zm00001d020403	2	0h	1.000
Zm00001d020425	3	0h	1.000
Zm00001d020497	1	0h	0.271
Zm00001d020610	2	0h	1.000
Zm00001d020620	2	0h	1.000
Zm00001d020651	5	0h	1.000
Zm00001d020909	3	0h	1.000
Zm00001d021024	5	0h	1.000
Zm00001d021216	2	0h	1.000
Zm00001d021294	2	0h	1.000
Zm00001d021338	2	0h	1.000
Zm00001d021439	3	0h	1.000
Zm00001d021576	4	0h	1.000
Zm00001d021744	3	0h	0.991
Zm00001d021778	2	0h	1.000
Zm00001d021967	2	0h	1.000
Zm00001d021995	5	0h	1.000
Zm00001d022040	2	0h	1.000
Zm00001d022045	2	0h	1.000
Zm00001d022067	2	0h	1.000
Zm00001d022265	5	0h	1.000
Zm00001d022350	2	0h	1.000
Zm00001d022474	2	0h	1.000
Zm00001d022529	5	0h	1.000
Zm00001d023240	1	0h	0.273
Zm00001d023253	4	0h	0.967
Zm00001d023291	2	0h	1.000
Zm00001d023300	2	0h	1.000
Zm00001d023312	2	0h	1.000
Zm00001d023396	3	0h	1.000
Zm00001d023455	3	0h	1.000
Zm00001d023654	5	0h	1.000
Zm00001d023700	1	0h	0.000

Transcripts	Cluster	Sample	Normalized max pausing score
Zm00001d023767	5	0h	1.000
Zm00001d024088	2	0h	1.000
Zm00001d024253	5	0h	1.000
Zm00001d024322	2	0h	1.000
Zm00001d024324	1	0h	0.000
Zm00001d024327	3	0h	1.000
Zm00001d024647	2	0h	1.000
Zm00001d024681	5	0h	0.862
Zm00001d024687	2	0h	1.000
Zm00001d024717	4	0h	1.000
Zm00001d024768	2	0h	1.000
Zm00001d024823	5	0h	1.000
Zm00001d024873	3	0h	1.000
Zm00001d025027	1	0h	0.279
Zm00001d025040	4	0h	0.926
Zm00001d025247	3	0h	1.000
Zm00001d025656	3	0h	1.000
Zm00001d025746	3	0h	0.852
Zm00001d025804	2	0h	1.000
Zm00001d025807	3	0h	1.000
Zm00001d025842	2	0h	1.000
Zm00001d026032	2	0h	1.000
Zm00001d026397	1	0h	0.224
Zm00001d026406	3	0h	1.000
Zm00001d026592	1	0h	0.000
Zm00001d027292	2	0h	1.000
Zm00001d027308	4	0h	1.000
Zm00001d027338	4	0h	1.000
Zm00001d027472	1	0h	0.234
Zm00001d027530	5	0h	1.000
Zm00001d027622	3	0h	1.000
Zm00001d027673	3	0h	1.000
Zm00001d027741	2	0h	1.000
Zm00001d027751	3	0h	1.000
Zm00001d027946	5	0h	1.000
Zm00001d028004	3	0h	1.000
Zm00001d028025	2	0h	1.000
Zm00001d028073	2	0h	1.000
Zm00001d028362	4	0h	1.000
Zm00001d028427	2	0h	1.000
Zm00001d028447	5	0h	1.000
Zm00001d028615	2	0h	1.000
Zm00001d028697	4	0h	1.000
Zm00001d028714	2	0h	1.000
Zm00001d028835	5	0h	1.000
Zm00001d028899	2	0h	1.000
Zm00001d028925	2	0h	1.000
Zm00001d029059	3	0h	1.000
Zm00001d029241	3	0h	1.000
Zm00001d029257	4	0h	0.833
Zm00001d029402	2	0h	1.000

Transcripts	Cluster	Sample	Normalized max pausing score
Zm00001d029427	2	0h	1.000
Zm00001d029579	2	0h	1.000
Zm00001d029676	5	0h	1.000
Zm00001d029921	4	0h	1.000
Zm00001d029950	4	0h	1.000
Zm00001d029969	2	0h	1.000
Zm00001d030016	4	0h	1.000
Zm00001d030299	5	0h	1.000
Zm00001d030305	2	0h	1.000
Zm00001d030470	3	0h	1.000
Zm00001d030661	3	0h	0.734
Zm00001d030775	1	0h	0.108
Zm00001d030851	2	0h	1.000
Zm00001d030877	2	0h	1.000
Zm00001d030942	3	0h	1.000
Zm00001d031189	1	0h	0.500
Zm00001d031230	3	0h	1.000
Zm00001d031454	5	0h	1.000
Zm00001d031465	1	0h	0.667
Zm00001d031533	5	0h	1.000
Zm00001d031717	3	0h	1.000
Zm00001d031730	3	0h	0.917
Zm00001d031782	5	0h	1.000
Zm00001d031858	4	0h	1.000
Zm00001d032274	5	0h	0.885
Zm00001d032324	5	0h	1.000
Zm00001d032433	4	0h	1.000
Zm00001d032439	3	0h	1.000
Zm00001d032496	2	0h	1.000
Zm00001d032587	2	0h	1.000
Zm00001d032616	1	0h	0.149
Zm00001d032858	5	0h	1.000
Zm00001d032870	3	0h	1.000
Zm00001d032978	3	0h	1.000
Zm00001d033068	3	0h	0.906
Zm00001d033139	2	0h	1.000
Zm00001d033374	4	0h	1.000
Zm00001d033505	2	0h	1.000
Zm00001d033583	2	0h	1.000
Zm00001d033595	5	0h	1.000
Zm00001d033836	2	0h	1.000
Zm00001d033879	3	0h	1.000
Zm00001d034064	3	0h	1.000
Zm00001d034068	2	0h	1.000
Zm00001d034501	2	0h	1.000
Zm00001d034738	2	0h	1.000
Zm00001d034888	1	0h	0.333
Zm00001d035020	2	0h	1.000
Zm00001d035163	2	0h	1.000
Zm00001d035322	4	0h	1.000
Zm00001d035383	5	0h	1.000

Transcripts	Cluster	Sample	Normalized max pausing score
Zm00001d035457	3	0h	0.800
Zm00001d035592	5	0h	1.000
Zm00001d035963	4	0h	1.000
Zm00001d036152	5	0h	1.000
Zm00001d036532	5	0h	1.000
Zm00001d036550	1	0h	0.109
Zm00001d036615	4	0h	0.793
Zm00001d036690	4	0h	0.778
Zm00001d036946	4	0h	1.000
Zm00001d037017	3	0h	1.000
Zm00001d037182	3	0h	1.000
Zm00001d037197	5	0h	1.000
Zm00001d037666	2	0h	1.000
Zm00001d037799	3	0h	1.000
Zm00001d037958	3	0h	1.000
Zm00001d038067	4	0h	1.000
Zm00001d038117	1	0h	0.272
Zm00001d038281	2	0h	1.000
Zm00001d038342	2	0h	1.000
Zm00001d038485	3	0h	1.000
Zm00001d038626	1	0h	0.298
Zm00001d038645	5	0h	1.000
Zm00001d038793	2	0h	1.000
Zm00001d038850	3	0h	1.000
Zm00001d038937	2	0h	1.000
Zm00001d038960	2	0h	1.000
Zm00001d039057	2	0h	1.000
Zm00001d039101	2	0h	1.000
Zm00001d039103	4	0h	1.000
Zm00001d039132	2	0h	1.000
Zm00001d039219	4	0h	1.000
Zm00001d039240	2	0h	1.000
Zm00001d039310	2	0h	1.000
Zm00001d039510	2	0h	1.000
Zm00001d039579	4	0h	1.000
Zm00001d039637	1	0h	0.429
Zm00001d039642	2	0h	1.000
Zm00001d039694	2	0h	1.000
Zm00001d039946	1	0h	0.249
Zm00001d040047	1	0h	0.520
Zm00001d040112	2	0h	1.000
Zm00001d040220	4	0h	1.000
Zm00001d040541	5	0h	1.000
Zm00001d040544	3	0h	1.000
Zm00001d040628	3	0h	0.917
Zm00001d040702	2	0h	1.000
Zm00001d040724	3	0h	1.000
Zm00001d040743	4	0h	1.000
Zm00001d041774	1	0h	0.269
Zm00001d042169	5	0h	1.000
Zm00001d042508	2	0h	1.000

Transcripts	Cluster	Sample	Normalized max pausing score
Zm00001d042676	1	0h	0.465
Zm00001d042765	5	0h	1.000
Zm00001d042766	5	0h	1.000
Zm00001d042801	3	0h	1.000
Zm00001d042909	4	0h	1.000
Zm00001d042936	2	0h	1.000
Zm00001d043145	2	0h	1.000
Zm00001d043234	4	0h	1.000
Zm00001d043293	4	0h	1.000
Zm00001d043339	2	0h	1.000
Zm00001d043391	4	0h	0.966
Zm00001d043400	2	0h	1.000
Zm00001d043558	4	0h	1.000
Zm00001d043596	2	0h	1.000
Zm00001d043702	3	0h	1.000
Zm00001d043751	2	0h	1.000
Zm00001d043870	3	0h	1.000
Zm00001d044052	5	0h	1.000
Zm00001d044059	3	0h	1.000
Zm00001d044202	3	0h	0.962
Zm00001d044417	2	0h	1.000
Zm00001d044515	5	0h	1.000
Zm00001d044597	5	0h	1.000
Zm00001d044802	3	0h	0.744
Zm00001d044874	2	0h	1.000
Zm00001d044895	3	0h	1.000
Zm00001d044911	2	0h	1.000
Zm00001d044918	3	0h	1.000
Zm00001d045370	2	0h	1.000
Zm00001d045495	4	0h	1.000
Zm00001d045515	3	0h	1.000
Zm00001d045598	5	0h	1.000
Zm00001d045755	3	0h	1.000
Zm00001d045788	2	0h	1.000
Zm00001d045888	5	0h	1.000
Zm00001d045913	3	0h	0.882
Zm00001d046383	2	0h	1.000
Zm00001d046501	5	0h	1.000
Zm00001d046621	5	0h	1.000
Zm00001d046672	2	0h	1.000
Zm00001d046696	4	0h	1.000
Zm00001d046729	5	0h	1.000
Zm00001d046743	3	0h	1.000
Zm00001d046759	2	0h	1.000
Zm00001d046882	5	0h	1.000
Zm00001d046947	3	0h	1.000
Zm00001d046979	2	0h	1.000
Zm00001d047013	1	0h	0.000
Zm00001d047069	2	0h	1.000
Zm00001d047202	3	0h	1.000
Zm00001d047499	4	0h	0.889

Transcripts	Cluster	Sample	Normalized max pausing score
Zm00001d047637	3	0h	1.000
Zm00001d047708	2	0h	1.000
Zm00001d047753	2	0h	1.000
Zm00001d047921	4	0h	0.909
Zm00001d047937	2	0h	1.000
Zm00001d048032	1	0h	0.000
Zm00001d048113	5	0h	1.000
Zm00001d048131	2	0h	1.000
Zm00001d048192	5	0h	1.000
Zm00001d048234	1	0h	0.333
Zm00001d048451	2	0h	1.000
Zm00001d048595	3	0h	1.000
Zm00001d048635	5	0h	1.000
Zm00001d048695	3	0h	0.929
Zm00001d048711	2	0h	1.000
Zm00001d048991	3	0h	1.000
Zm00001d049145	5	0h	1.000
Zm00001d049228	3	0h	1.000
Zm00001d049332	2	0h	1.000
Zm00001d049400	1	0h	0.328
Zm00001d049499	2	0h	1.000
Zm00001d049585	1	0h	0.250
Zm00001d049595	5	0h	0.848
Zm00001d050092	2	0h	1.000
Zm00001d050141	2	0h	1.000
Zm00001d050172	2	0h	1.000
Zm00001d050294	2	0h	1.000
Zm00001d050308	4	0h	1.000
Zm00001d050335	3	0h	0.789
Zm00001d050350	3	0h	1.000
Zm00001d050498	1	0h	0.000
Zm00001d050558	5	0h	1.000
Zm00001d050830	3	0h	1.000
Zm00001d051140	1	0h	0.333
Zm00001d051424	2	0h	1.000
Zm00001d051474	1	0h	0.667
Zm00001d051660	2	0h	1.000
Zm00001d051672	3	0h	1.000
Zm00001d051788	4	0h	1.000
Zm00001d052209	3	0h	1.000
Zm00001d052212	2	0h	1.000
Zm00001d052239	2	0h	1.000
Zm00001d052385	2	0h	1.000
Zm00001d052471	3	0h	1.000
Zm00001d052618	2	0h	1.000
Zm00001d052701	2	0h	1.000
Zm00001d052847	3	0h	1.000
Zm00001d052933	3	0h	1.000
Zm00001d052944	2	0h	1.000
Zm00001d052981	4	0h	0.862
Zm00001d053017	2	0h	1.000

Transcripts	Cluster	Sample	Normalized max pausing score
Zm00001d053156	3	0h	1.000
Zm00001d053244	3	0h	0.958
Zm00001d053306	3	0h	1.000
Zm00001d053346	5	0h	1.000
Zm00001d053695	2	0h	1.000
Zm00001d053826	2	0h	1.000
Zm00001d054009	2	0h	1.000
Zm00001d054071	2	0h	1.000
ZeamMp030	3	0.5h	1.000
ZeamMp033	5	0.5h	0.289
ZeamMp034	4	0.5h	0.675
ZeamMp041	4	0.5h	0.600
ZeamMp137	3	0.5h	0.557
Zm00001d000035	3	0.5h	0.569
Zm00001d000390	2	0.5h	0.395
Zm00001d002058	5	0.5h	0.376
Zm00001d002086	1	0.5h	1.000
Zm00001d002131	2	0.5h	0.114
Zm00001d002542	3	0.5h	0.580
Zm00001d002684	1	0.5h	1.000
Zm00001d002757	3	0.5h	0.583
Zm00001d002782	3	0.5h	0.640
Zm00001d002899	2	0.5h	0.175
Zm00001d003088	5	0.5h	0.571
Zm00001d003183	1	0.5h	1.000
Zm00001d003281	5	0.5h	0.370
Zm00001d003399	3	0.5h	0.525
Zm00001d003400	4	0.5h	0.000
Zm00001d003427	2	0.5h	0.402
Zm00001d003435	5	0.5h	0.000
Zm00001d003463	2	0.5h	0.175
Zm00001d003516	4	0.5h	0.347
Zm00001d003538	2	0.5h	0.250
Zm00001d003593	2	0.5h	0.294
Zm00001d003743	2	0.5h	0.171
Zm00001d003763	4	0.5h	0.360
Zm00001d004301	1	0.5h	1.000
Zm00001d004310	1	0.5h	1.000
Zm00001d004910	4	0.5h	0.000
Zm00001d005109	5	0.5h	0.556
Zm00001d005480	3	0.5h	1.000
Zm00001d005504	3	0.5h	1.000
Zm00001d005612	2	0.5h	0.383
Zm00001d005680	4	0.5h	0.500
Zm00001d005909	3	0.5h	0.893
Zm00001d005936	2	0.5h	0.139
Zm00001d005962	2	0.5h	0.500
Zm00001d005989	3	0.5h	0.983
Zm00001d006000	2	0.5h	0.000
Zm00001d006011	3	0.5h	0.954
Zm00001d006045	3	0.5h	0.571

Transcripts	Cluster	Sample	Normalized max pausing score
Zm00001d006132	3	0.5h	0.500
Zm00001d006193	4	0.5h	0.000
Zm00001d006321	2	0.5h	0.278
Zm00001d006619	3	0.5h	0.864
Zm00001d006631	1	0.5h	1.000
Zm00001d006638	5	0.5h	0.250
Zm00001d006894	5	0.5h	0.500
Zm00001d006947	4	0.5h	0.000
Zm00001d006950	1	0.5h	1.000
Zm00001d007015	1	0.5h	1.000
Zm00001d007050	2	0.5h	0.324
Zm00001d007162	3	0.5h	0.638
Zm00001d007197	2	0.5h	0.244
Zm00001d007258	2	0.5h	0.226
Zm00001d007259	5	0.5h	0.252
Zm00001d007294	3	0.5h	0.545
Zm00001d007478	4	0.5h	0.000
Zm00001d007503	5	0.5h	0.500
Zm00001d007518	1	0.5h	1.000
Zm00001d007606	4	0.5h	0.000
Zm00001d007839	2	0.5h	0.108
Zm00001d007869	2	0.5h	0.349
Zm00001d008187	4	0.5h	0.000
Zm00001d008219	2	0.5h	0.000
Zm00001d008297	2	0.5h	0.219
Zm00001d008298	5	0.5h	0.000
Zm00001d008329	2	0.5h	0.117
Zm00001d008409	5	0.5h	0.377
Zm00001d008764	2	0.5h	0.393
Zm00001d008827	3	0.5h	0.692
Zm00001d008859	3	0.5h	0.577
Zm00001d009008	4	0.5h	0.000
Zm00001d009108	2	0.5h	0.134
Zm00001d009138	4	0.5h	0.333
Zm00001d009336	3	0.5h	1.000
Zm00001d009568	2	0.5h	0.295
Zm00001d009747	4	0.5h	0.241
Zm00001d009787	2	0.5h	0.239
Zm00001d010044	2	0.5h	0.284
Zm00001d010222	3	0.5h	0.765
Zm00001d010325	2	0.5h	0.000
Zm00001d010388	3	0.5h	0.906
Zm00001d010564	2	0.5h	0.213
Zm00001d010590	2	0.5h	0.312
Zm00001d010594	2	0.5h	0.167
Zm00001d010610	3	0.5h	0.500
Zm00001d010621	2	0.5h	0.500
Zm00001d010785	5	0.5h	0.500
Zm00001d010788	3	0.5h	0.660
Zm00001d010868	2	0.5h	0.344
Zm00001d010872	2	0.5h	0.300

Transcripts	Cluster	Sample	Normalized max pausing score
Zm00001d010925	2	0.5h	0.000
Zm00001d011068	4	0.5h	0.223
Zm00001d011620	1	0.5h	1.000
Zm00001d011881	3	0.5h	0.759
Zm00001d011890	5	0.5h	0.298
Zm00001d011964	1	0.5h	1.000
Zm00001d012041	2	0.5h	0.000
Zm00001d012237	2	0.5h	0.000
Zm00001d012275	3	0.5h	0.605
Zm00001d012289	2	0.5h	0.297
Zm00001d012387	1	0.5h	1.000
Zm00001d012612	2	0.5h	0.427
Zm00001d012626	2	0.5h	0.000
Zm00001d012785	2	0.5h	0.250
Zm00001d012934	5	0.5h	0.386
Zm00001d013069	1	0.5h	1.000
Zm00001d013162	2	0.5h	0.212
Zm00001d013311	4	0.5h	0.000
Zm00001d013339	5	0.5h	0.199
Zm00001d013342	2	0.5h	0.250
Zm00001d013399	2	0.5h	0.395
Zm00001d013794	3	0.5h	1.000
Zm00001d013923	2	0.5h	0.290
Zm00001d014196	4	0.5h	0.333
Zm00001d014253	1	0.5h	0.963
Zm00001d014414	5	0.5h	0.000
Zm00001d014463	2	0.5h	0.213
Zm00001d014704	2	0.5h	0.218
Zm00001d014820	2	0.5h	0.059
Zm00001d014994	2	0.5h	0.148
Zm00001d015059	5	0.5h	0.000
Zm00001d015129	3	0.5h	0.593
Zm00001d015202	2	0.5h	0.000
Zm00001d015215	2	0.5h	0.260
Zm00001d015407	4	0.5h	0.310
Zm00001d015412	2	0.5h	0.305
Zm00001d015744	1	0.5h	1.000
Zm00001d015779	4	0.5h	0.385
Zm00001d015884	3	0.5h	0.500
Zm00001d015990	4	0.5h	0.000
Zm00001d016154	5	0.5h	0.000
Zm00001d016262	2	0.5h	0.000
Zm00001d016301	5	0.5h	0.000
Zm00001d016322	3	0.5h	1.000
Zm00001d016417	2	0.5h	0.000
Zm00001d016648	5	0.5h	0.000
Zm00001d016806	5	0.5h	0.000
Zm00001d016831	4	0.5h	0.149
Zm00001d016844	5	0.5h	0.000
Zm00001d016896	3	0.5h	0.586
Zm00001d017351	3	0.5h	0.990

Transcripts	Cluster	Sample	Normalized max pausing score
Zm00001d017353	1	0.5h	1.000
Zm00001d017462	4	0.5h	0.396
Zm00001d017530	2	0.5h	0.387
Zm00001d017696	2	0.5h	0.309
Zm00001d017746	2	0.5h	0.105
Zm00001d017851	3	0.5h	1.000
Zm00001d017991	2	0.5h	0.000
Zm00001d018058	3	0.5h	1.000
Zm00001d018081	2	0.5h	0.556
Zm00001d018117	1	0.5h	1.000
Zm00001d018133	4	0.5h	0.394
Zm00001d018191	1	0.5h	1.000
Zm00001d018475	4	0.5h	0.000
Zm00001d018696	3	0.5h	0.721
Zm00001d018806	2	0.5h	0.175
Zm00001d019002	2	0.5h	0.193
Zm00001d019400	3	0.5h	0.537
Zm00001d019422	5	0.5h	0.556
Zm00001d019582	5	0.5h	0.000
Zm00001d019669	1	0.5h	1.000
Zm00001d019925	5	0.5h	0.000
Zm00001d019989	3	0.5h	0.513
Zm00001d019990	2	0.5h	0.000
Zm00001d020176	5	0.5h	0.000
Zm00001d020277	4	0.5h	0.616
Zm00001d020403	2	0.5h	0.225
Zm00001d020425	3	0.5h	0.429
Zm00001d020497	1	0.5h	1.000
Zm00001d020610	2	0.5h	0.160
Zm00001d020620	2	0.5h	0.226
Zm00001d020651	5	0.5h	0.000
Zm00001d020909	3	0.5h	1.000
Zm00001d021024	5	0.5h	0.225
Zm00001d021216	2	0.5h	0.167
Zm00001d021294	2	0.5h	0.000
Zm00001d021338	2	0.5h	0.221
Zm00001d021439	3	0.5h	1.000
Zm00001d021576	4	0.5h	0.000
Zm00001d021744	3	0.5h	1.000
Zm00001d021778	2	0.5h	0.000
Zm00001d021967	2	0.5h	0.000
Zm00001d021995	5	0.5h	0.393
Zm00001d022040	2	0.5h	0.435
Zm00001d022045	2	0.5h	0.233
Zm00001d022067	2	0.5h	0.000
Zm00001d022265	5	0.5h	0.261
Zm00001d022350	2	0.5h	0.000
Zm00001d022474	2	0.5h	0.354
Zm00001d022529	5	0.5h	0.000
Zm00001d023240	1	0.5h	1.000
Zm00001d023253	4	0.5h	0.241

Transcripts	Cluster	Sample	Normalized max pausing score
Zm00001d023291	2	0.5h	0.216
Zm00001d023300	2	0.5h	0.333
Zm00001d023312	2	0.5h	0.360
Zm00001d023396	3	0.5h	0.747
Zm00001d023455	3	0.5h	0.571
Zm00001d023654	5	0.5h	0.279
Zm00001d023700	1	0.5h	1.000
Zm00001d023767	5	0.5h	0.252
Zm00001d024088	2	0.5h	0.341
Zm00001d024253	5	0.5h	0.500
Zm00001d024322	2	0.5h	0.307
Zm00001d024324	1	0.5h	1.000
Zm00001d024327	3	0.5h	0.918
Zm00001d024647	2	0.5h	0.232
Zm00001d024681	5	0.5h	0.500
Zm00001d024687	2	0.5h	0.400
Zm00001d024717	4	0.5h	0.000
Zm00001d024768	2	0.5h	0.457
Zm00001d024823	5	0.5h	0.397
Zm00001d024873	3	0.5h	0.500
Zm00001d025027	1	0.5h	1.000
Zm00001d025040	4	0.5h	0.000
Zm00001d025247	3	0.5h	0.822
Zm00001d025656	3	0.5h	0.667
Zm00001d025746	3	0.5h	1.000
Zm00001d025804	2	0.5h	0.538
Zm00001d025807	3	0.5h	0.528
Zm00001d025842	2	0.5h	0.528
Zm00001d026032	2	0.5h	0.000
Zm00001d026397	1	0.5h	1.000
Zm00001d026406	3	0.5h	0.667
Zm00001d026592	1	0.5h	0.913
Zm00001d027292	2	0.5h	0.391
Zm00001d027308	4	0.5h	0.000
Zm00001d027338	4	0.5h	0.000
Zm00001d027472	1	0.5h	1.000
Zm00001d027530	5	0.5h	0.355
Zm00001d027622	3	0.5h	0.533
Zm00001d027673	3	0.5h	0.944
Zm00001d027741	2	0.5h	0.523
Zm00001d027751	3	0.5h	0.500
Zm00001d027946	5	0.5h	0.353
Zm00001d028004	3	0.5h	0.517
Zm00001d028025	2	0.5h	0.200
Zm00001d028073	2	0.5h	0.000
Zm00001d028362	4	0.5h	0.278
Zm00001d028427	2	0.5h	0.336
Zm00001d028447	5	0.5h	0.518
Zm00001d028615	2	0.5h	0.000
Zm00001d028697	4	0.5h	0.545
Zm00001d028714	2	0.5h	0.200

Transcripts	Cluster	Sample	Normalized max pausing score
Zm00001d028835	5	0.5h	0.524
Zm00001d028899	2	0.5h	0.000
Zm00001d028925	2	0.5h	0.000
Zm00001d029059	3	0.5h	0.500
Zm00001d029241	3	0.5h	0.713
Zm00001d029257	4	0.5h	0.000
Zm00001d029402	2	0.5h	0.206
Zm00001d029427	2	0.5h	0.000
Zm00001d029579	2	0.5h	0.384
Zm00001d029676	5	0.5h	0.000
Zm00001d029921	4	0.5h	0.000
Zm00001d029950	4	0.5h	0.000
Zm00001d029969	2	0.5h	0.400
Zm00001d030016	4	0.5h	0.417
Zm00001d030299	5	0.5h	0.000
Zm00001d030305	2	0.5h	0.000
Zm00001d030470	3	0.5h	0.870
Zm00001d030661	3	0.5h	1.000
Zm00001d030775	1	0.5h	1.000
Zm00001d030851	2	0.5h	0.413
Zm00001d030877	2	0.5h	0.000
Zm00001d030942	3	0.5h	0.920
Zm00001d031189	1	0.5h	1.000
Zm00001d031230	3	0.5h	0.957
Zm00001d031454	5	0.5h	0.345
Zm00001d031465	1	0.5h	1.000
Zm00001d031533	5	0.5h	0.343
Zm00001d031717	3	0.5h	0.765
Zm00001d031730	3	0.5h	1.000
Zm00001d031782	5	0.5h	0.000
Zm00001d031858	4	0.5h	0.258
Zm00001d032274	5	0.5h	0.509
Zm00001d032324	5	0.5h	0.282
Zm00001d032433	4	0.5h	0.000
Zm00001d032439	3	0.5h	0.562
Zm00001d032496	2	0.5h	0.132
Zm00001d032587	2	0.5h	0.166
Zm00001d032616	1	0.5h	1.000
Zm00001d032858	5	0.5h	0.230
Zm00001d032870	3	0.5h	0.586
Zm00001d032978	3	0.5h	0.531
Zm00001d033068	3	0.5h	1.000
Zm00001d033139	2	0.5h	0.000
Zm00001d033374	4	0.5h	0.000
Zm00001d033505	2	0.5h	0.302
Zm00001d033583	2	0.5h	0.000
Zm00001d033595	5	0.5h	0.000
Zm00001d033836	2	0.5h	0.134
Zm00001d033879	3	0.5h	0.993
Zm00001d034064	3	0.5h	0.749
Zm00001d034068	2	0.5h	0.412

Transcripts	Cluster	Sample	Normalized max pausing score
Zm00001d034501	2	0.5h	0.356
Zm00001d034738	2	0.5h	0.436
Zm00001d034888	1	0.5h	0.960
Zm00001d035020	2	0.5h	0.000
Zm00001d035163	2	0.5h	0.193
Zm00001d035322	4	0.5h	0.000
Zm00001d035383	5	0.5h	0.280
Zm00001d035457	3	0.5h	0.920
Zm00001d035592	5	0.5h	0.000
Zm00001d035963	4	0.5h	0.384
Zm00001d036152	5	0.5h	0.560
Zm00001d036532	5	0.5h	0.182
Zm00001d036550	1	0.5h	1.000
Zm00001d036615	4	0.5h	0.714
Zm00001d036690	4	0.5h	0.000
Zm00001d036946	4	0.5h	0.000
Zm00001d037017	3	0.5h	0.560
Zm00001d037182	3	0.5h	0.519
Zm00001d037197	5	0.5h	0.521
Zm00001d037666	2	0.5h	0.424
Zm00001d037799	3	0.5h	0.455
Zm00001d037958	3	0.5h	0.714
Zm00001d038067	4	0.5h	0.518
Zm00001d038117	1	0.5h	1.000
Zm00001d038281	2	0.5h	0.345
Zm00001d038342	2	0.5h	0.543
Zm00001d038485	3	0.5h	0.512
Zm00001d038626	1	0.5h	1.000
Zm00001d038645	5	0.5h	0.000
Zm00001d038793	2	0.5h	0.250
Zm00001d038850	3	0.5h	0.603
Zm00001d038937	2	0.5h	0.114
Zm00001d038960	2	0.5h	0.162
Zm00001d039057	2	0.5h	0.000
Zm00001d039101	2	0.5h	0.000
Zm00001d039103	4	0.5h	0.000
Zm00001d039132	2	0.5h	0.000
Zm00001d039219	4	0.5h	0.000
Zm00001d039240	2	0.5h	0.000
Zm00001d039310	2	0.5h	0.201
Zm00001d039510	2	0.5h	0.000
Zm00001d039579	4	0.5h	0.000
Zm00001d039637	1	0.5h	1.000
Zm00001d039642	2	0.5h	0.140
Zm00001d039694	2	0.5h	0.000
Zm00001d039946	1	0.5h	1.000
Zm00001d040047	1	0.5h	1.000
Zm00001d040112	2	0.5h	0.302
Zm00001d040220	4	0.5h	0.000
Zm00001d040541	5	0.5h	0.583
Zm00001d040544	3	0.5h	0.629

Transcripts	Cluster	Sample	Normalized max pausing score
Zm00001d040628	3	0.5h	1.000
Zm00001d040702	2	0.5h	0.205
Zm00001d040724	3	0.5h	0.583
Zm00001d040743	4	0.5h	0.514
Zm00001d041774	1	0.5h	1.000
Zm00001d042169	5	0.5h	0.643
Zm00001d042508	2	0.5h	0.297
Zm00001d042676	1	0.5h	1.000
Zm00001d042765	5	0.5h	0.000
Zm00001d042766	5	0.5h	0.556
Zm00001d042801	3	0.5h	0.812
Zm00001d042909	4	0.5h	0.233
Zm00001d042936	2	0.5h	0.000
Zm00001d043145	2	0.5h	0.153
Zm00001d043234	4	0.5h	0.000
Zm00001d043293	4	0.5h	0.420
Zm00001d043339	2	0.5h	0.248
Zm00001d043391	4	0.5h	0.250
Zm00001d043400	2	0.5h	0.381
Zm00001d043558	4	0.5h	0.000
Zm00001d043596	2	0.5h	0.000
Zm00001d043702	3	0.5h	0.975
Zm00001d043751	2	0.5h	0.000
Zm00001d043870	3	0.5h	0.713
Zm00001d044052	5	0.5h	0.000
Zm00001d044059	3	0.5h	0.524
Zm00001d044202	3	0.5h	1.000
Zm00001d044417	2	0.5h	0.229
Zm00001d044515	5	0.5h	0.387
Zm00001d044597	5	0.5h	0.304
Zm00001d044802	3	0.5h	1.000
Zm00001d044874	2	0.5h	0.500
Zm00001d044895	3	0.5h	0.524
Zm00001d044911	2	0.5h	0.000
Zm00001d044918	3	0.5h	0.831
Zm00001d045370	2	0.5h	0.429
Zm00001d045495	4	0.5h	0.000
Zm00001d045515	3	0.5h	0.568
Zm00001d045598	5	0.5h	0.548
Zm00001d045755	3	0.5h	0.500
Zm00001d045788	2	0.5h	0.175
Zm00001d045888	5	0.5h	0.000
Zm00001d045913	3	0.5h	1.000
Zm00001d046383	2	0.5h	0.000
Zm00001d046501	5	0.5h	0.000
Zm00001d046621	5	0.5h	0.000
Zm00001d046672	2	0.5h	0.171
Zm00001d046696	4	0.5h	0.000
Zm00001d046729	5	0.5h	0.000
Zm00001d046743	3	0.5h	0.643
Zm00001d046759	2	0.5h	0.144

Transcripts	Cluster	Sample	Normalized max pausing score
Zm00001d046882	5	0.5h	0.356
Zm00001d046947	3	0.5h	0.643
Zm00001d046979	2	0.5h	0.280
Zm00001d047013	1	0.5h	1.000
Zm00001d047069	2	0.5h	0.000
Zm00001d047202	3	0.5h	0.638
Zm00001d047499	4	0.5h	0.000
Zm00001d047637	3	0.5h	1.000
Zm00001d047708	2	0.5h	0.000
Zm00001d047753	2	0.5h	0.500
Zm00001d047921	4	0.5h	0.000
Zm00001d047937	2	0.5h	0.504
Zm00001d048032	1	0.5h	1.000
Zm00001d048113	5	0.5h	0.400
Zm00001d048131	2	0.5h	0.362
Zm00001d048192	5	0.5h	0.000
Zm00001d048234	1	0.5h	0.913
Zm00001d048451	2	0.5h	0.387
Zm00001d048595	3	0.5h	0.960
Zm00001d048635	5	0.5h	0.000
Zm00001d048695	3	0.5h	1.000
Zm00001d048711	2	0.5h	0.099
Zm00001d048991	3	0.5h	0.611
Zm00001d049145	5	0.5h	0.254
Zm00001d049228	3	0.5h	0.415
Zm00001d049332	2	0.5h	0.248
Zm00001d049400	1	0.5h	1.000
Zm00001d049499	2	0.5h	0.320
Zm00001d049585	1	0.5h	1.000
Zm00001d049595	5	0.5h	0.500
Zm00001d050092	2	0.5h	0.409
Zm00001d050141	2	0.5h	0.000
Zm00001d050172	2	0.5h	0.191
Zm00001d050294	2	0.5h	0.000
Zm00001d050308	4	0.5h	0.000
Zm00001d050335	3	0.5h	1.000
Zm00001d050350	3	0.5h	0.523
Zm00001d050498	1	0.5h	1.000
Zm00001d050558	5	0.5h	0.504
Zm00001d050830	3	0.5h	0.777
Zm00001d051140	1	0.5h	1.000
Zm00001d051424	2	0.5h	0.000
Zm00001d051474	1	0.5h	1.000
Zm00001d051660	2	0.5h	0.429
Zm00001d051672	3	0.5h	0.561
Zm00001d051788	4	0.5h	0.000
Zm00001d052209	3	0.5h	0.577
Zm00001d052212	2	0.5h	0.533
Zm00001d052239	2	0.5h	0.355
Zm00001d052385	2	0.5h	0.321
Zm00001d052471	3	0.5h	0.668

Transcripts	Cluster	Sample	Normalized max pausing score
Zm00001d052618	2	0.5h	0.337
Zm00001d052701	2	0.5h	0.424
Zm00001d052847	3	0.5h	0.738
Zm00001d052933	3	0.5h	0.500
Zm00001d052944	2	0.5h	0.390
Zm00001d052981	4	0.5h	0.286
Zm00001d053017	2	0.5h	0.321
Zm00001d053156	3	0.5h	0.595
Zm00001d053244	3	0.5h	0.900
Zm00001d053306	3	0.5h	0.504
Zm00001d053346	5	0.5h	0.000
Zm00001d053695	2	0.5h	0.160
Zm00001d053826	2	0.5h	0.127
Zm00001d054009	2	0.5h	0.294
Zm00001d054071	2	0.5h	0.000
ZeamMp030	3	1h	0.797
ZeamMp033	5	1h	0.799
ZeamMp034	4	1h	0.703
ZeamMp041	4	1h	0.938
ZeamMp137	3	1h	0.527
Zm00001d000035	3	1h	0.171
Zm00001d000390	2	1h	0.307
Zm00001d002058	5	1h	0.677
Zm00001d002086	1	1h	0.522
Zm00001d002131	2	1h	0.062
Zm00001d002542	3	1h	0.387
Zm00001d002684	1	1h	0.786
Zm00001d002757	3	1h	0.000
Zm00001d002782	3	1h	0.290
Zm00001d002899	2	1h	0.299
Zm00001d003088	5	1h	0.857
Zm00001d003183	1	1h	0.000
Zm00001d003281	5	1h	0.593
Zm00001d003399	3	1h	0.529
Zm00001d003400	4	1h	0.527
Zm00001d003427	2	1h	0.402
Zm00001d003435	5	1h	0.519
Zm00001d003463	2	1h	0.306
Zm00001d003516	4	1h	0.260
Zm00001d003538	2	1h	0.167
Zm00001d003593	2	1h	0.353
Zm00001d003743	2	1h	0.180
Zm00001d003763	4	1h	0.432
Zm00001d004301	1	1h	0.264
Zm00001d004310	1	1h	0.696
Zm00001d004910	4	1h	0.000
Zm00001d005109	5	1h	0.833
Zm00001d005480	3	1h	0.283
Zm00001d005504	3	1h	0.111
Zm00001d005612	2	1h	0.096
Zm00001d005680	4	1h	0.333

Transcripts	Cluster	Sample	Normalized max pausing score
Zm00001d005909	3	1h	0.239
Zm00001d005936	2	1h	0.182
Zm00001d005962	2	1h	0.000
Zm00001d005989	3	1h	0.365
Zm00001d006000	2	1h	0.000
Zm00001d006011	3	1h	0.236
Zm00001d006045	3	1h	0.381
Zm00001d006132	3	1h	0.500
Zm00001d006193	4	1h	0.697
Zm00001d006321	2	1h	0.327
Zm00001d006619	3	1h	0.348
Zm00001d006631	1	1h	0.583
Zm00001d006638	5	1h	1.000
Zm00001d006894	5	1h	1.000
Zm00001d006947	4	1h	0.348
Zm00001d006950	1	1h	0.337
Zm00001d007015	1	1h	0.375
Zm00001d007050	2	1h	0.241
Zm00001d007162	3	1h	0.318
Zm00001d007197	2	1h	0.306
Zm00001d007258	2	1h	0.151
Zm00001d007259	5	1h	0.540
Zm00001d007294	3	1h	0.242
Zm00001d007478	4	1h	0.000
Zm00001d007503	5	1h	1.000
Zm00001d007518	1	1h	1.000
Zm00001d007606	4	1h	0.788
Zm00001d007839	2	1h	0.142
Zm00001d007869	2	1h	0.403
Zm00001d008187	4	1h	0.551
Zm00001d008219	2	1h	0.000
Zm00001d008297	2	1h	0.284
Zm00001d008298	5	1h	1.000
Zm00001d008329	2	1h	0.151
Zm00001d008409	5	1h	0.471
Zm00001d008764	2	1h	0.077
Zm00001d008827	3	1h	0.415
Zm00001d008859	3	1h	0.216
Zm00001d009008	4	1h	0.537
Zm00001d009108	2	1h	0.269
Zm00001d009138	4	1h	0.000
Zm00001d009336	3	1h	0.161
Zm00001d009568	2	1h	0.184
Zm00001d009747	4	1h	0.361
Zm00001d009787	2	1h	0.153
Zm00001d010044	2	1h	0.406
Zm00001d010222	3	1h	0.500
Zm00001d010325	2	1h	0.000
Zm00001d010388	3	1h	0.592
Zm00001d010564	2	1h	0.064
Zm00001d010590	2	1h	0.451

Transcripts	Cluster	Sample	Normalized max pausing score
Zm00001d010594	2	1h	0.333
Zm00001d010610	3	1h	0.444
Zm00001d010621	2	1h	0.000
Zm00001d010785	5	1h	1.000
Zm00001d010788	3	1h	0.187
Zm00001d010868	2	1h	0.147
Zm00001d010872	2	1h	0.180
Zm00001d010925	2	1h	0.500
Zm00001d011068	4	1h	0.200
Zm00001d011620	1	1h	0.655
Zm00001d011881	3	1h	0.379
Zm00001d011890	5	1h	0.593
Zm00001d011964	1	1h	0.064
Zm00001d012041	2	1h	0.000
Zm00001d012237	2	1h	0.000
Zm00001d012275	3	1h	0.101
Zm00001d012289	2	1h	0.260
Zm00001d012387	1	1h	0.000
Zm00001d012612	2	1h	0.301
Zm00001d012626	2	1h	0.000
Zm00001d012785	2	1h	0.308
Zm00001d012934	5	1h	0.514
Zm00001d013069	1	1h	0.443
Zm00001d013162	2	1h	0.188
Zm00001d013311	4	1h	0.000
Zm00001d013339	5	1h	0.477
Zm00001d013342	2	1h	0.333
Zm00001d013399	2	1h	0.339
Zm00001d013794	3	1h	0.500
Zm00001d013923	2	1h	0.232
Zm00001d014196	4	1h	0.444
Zm00001d014253	1	1h	0.101
Zm00001d014414	5	1h	0.444
Zm00001d014463	2	1h	0.148
Zm00001d014704	2	1h	0.172
Zm00001d014820	2	1h	0.058
Zm00001d014994	2	1h	0.143
Zm00001d015059	5	1h	0.543
Zm00001d015129	3	1h	0.593
Zm00001d015202	2	1h	0.385
Zm00001d015215	2	1h	0.260
Zm00001d015407	4	1h	0.251
Zm00001d015412	2	1h	0.295
Zm00001d015744	1	1h	0.400
Zm00001d015779	4	1h	0.617
Zm00001d015884	3	1h	0.417
Zm00001d015990	4	1h	0.333
Zm00001d016154	5	1h	0.494
Zm00001d016262	2	1h	0.000
Zm00001d016301	5	1h	0.664
Zm00001d016322	3	1h	0.400

Transcripts	Cluster	Sample	Normalized max pausing score
Zm00001d016417	2	1h	0.379
Zm00001d016648	5	1h	0.545
Zm00001d016806	5	1h	0.593
Zm00001d016831	4	1h	0.184
Zm00001d016844	5	1h	0.500
Zm00001d016896	3	1h	0.521
Zm00001d017351	3	1h	0.323
Zm00001d017353	1	1h	0.548
Zm00001d017462	4	1h	0.616
Zm00001d017530	2	1h	0.362
Zm00001d017696	2	1h	0.187
Zm00001d017746	2	1h	0.241
Zm00001d017851	3	1h	0.464
Zm00001d017991	2	1h	0.257
Zm00001d018058	3	1h	0.334
Zm00001d018081	2	1h	0.197
Zm00001d018117	1	1h	0.000
Zm00001d018133	4	1h	0.452
Zm00001d018191	1	1h	0.208
Zm00001d018475	4	1h	0.350
Zm00001d018696	3	1h	0.271
Zm00001d018806	2	1h	0.314
Zm00001d019002	2	1h	0.347
Zm00001d019400	3	1h	0.537
Zm00001d019422	5	1h	0.694
Zm00001d019582	5	1h	0.667
Zm00001d019669	1	1h	0.182
Zm00001d019925	5	1h	1.000
Zm00001d019989	3	1h	0.570
Zm00001d019990	2	1h	0.000
Zm00001d020176	5	1h	0.692
Zm00001d020277	4	1h	0.828
Zm00001d020403	2	1h	0.333
Zm00001d020425	3	1h	0.236
Zm00001d020497	1	1h	0.431
Zm00001d020610	2	1h	0.078
Zm00001d020620	2	1h	0.355
Zm00001d020651	5	1h	0.744
Zm00001d020909	3	1h	0.000
Zm00001d021024	5	1h	0.675
Zm00001d021216	2	1h	0.071
Zm00001d021294	2	1h	0.500
Zm00001d021338	2	1h	0.352
Zm00001d021439	3	1h	0.600
Zm00001d021576	4	1h	0.527
Zm00001d021744	3	1h	0.383
Zm00001d021778	2	1h	0.347
Zm00001d021967	2	1h	0.347
Zm00001d021995	5	1h	0.589
Zm00001d022040	2	1h	0.303
Zm00001d022045	2	1h	0.318

Transcripts	Cluster	Sample	Normalized max pausing score
Zm00001d022067	2	1h	0.333
Zm00001d022265	5	1h	0.696
Zm00001d022350	2	1h	0.000
Zm00001d022474	2	1h	0.310
Zm00001d022529	5	1h	0.741
Zm00001d023240	1	1h	0.333
Zm00001d023253	4	1h	0.417
Zm00001d023291	2	1h	0.226
Zm00001d023300	2	1h	0.000
Zm00001d023312	2	1h	0.000
Zm00001d023396	3	1h	0.320
Zm00001d023455	3	1h	0.286
Zm00001d023654	5	1h	0.446
Zm00001d023700	1	1h	0.000
Zm00001d023767	5	1h	0.504
Zm00001d024088	2	1h	0.248
Zm00001d024253	5	1h	0.714
Zm00001d024322	2	1h	0.273
Zm00001d024324	1	1h	0.348
Zm00001d024327	3	1h	0.351
Zm00001d024647	2	1h	0.238
Zm00001d024681	5	1h	1.000
Zm00001d024687	2	1h	0.083
Zm00001d024717	4	1h	0.000
Zm00001d024768	2	1h	0.381
Zm00001d024823	5	1h	0.433
Zm00001d024873	3	1h	0.400
Zm00001d025027	1	1h	0.034
Zm00001d025040	4	1h	1.000
Zm00001d025247	3	1h	0.130
Zm00001d025656	3	1h	0.489
Zm00001d025746	3	1h	0.554
Zm00001d025804	2	1h	0.190
Zm00001d025807	3	1h	0.545
Zm00001d025842	2	1h	0.220
Zm00001d026032	2	1h	0.000
Zm00001d026397	1	1h	0.626
Zm00001d026406	3	1h	0.400
Zm00001d026592	1	1h	0.500
Zm00001d027292	2	1h	0.391
Zm00001d027308	4	1h	0.000
Zm00001d027338	4	1h	0.235
Zm00001d027472	1	1h	0.226
Zm00001d027530	5	1h	0.518
Zm00001d027622	3	1h	0.427
Zm00001d027673	3	1h	0.750
Zm00001d027741	2	1h	0.000
Zm00001d027751	3	1h	0.333
Zm00001d027946	5	1h	0.847
Zm00001d028004	3	1h	0.326
Zm00001d028025	2	1h	0.000

Transcripts	Cluster	Sample	Normalized max pausing score
Zm00001d028073	2	1h	0.277
Zm00001d028362	4	1h	0.000
Zm00001d028427	2	1h	0.145
Zm00001d028447	5	1h	0.829
Zm00001d028615	2	1h	0.000
Zm00001d028697	4	1h	0.545
Zm00001d028714	2	1h	0.182
Zm00001d028835	5	1h	0.698
Zm00001d028899	2	1h	0.000
Zm00001d028925	2	1h	0.000
Zm00001d029059	3	1h	0.348
Zm00001d029241	3	1h	0.230
Zm00001d029257	4	1h	0.429
Zm00001d029402	2	1h	0.281
Zm00001d029427	2	1h	0.346
Zm00001d029579	2	1h	0.314
Zm00001d029676	5	1h	0.500
Zm00001d029921	4	1h	0.643
Zm00001d029950	4	1h	0.129
Zm00001d029969	2	1h	0.286
Zm00001d030016	4	1h	0.500
Zm00001d030299	5	1h	0.523
Zm00001d030305	2	1h	0.393
Zm00001d030470	3	1h	0.580
Zm00001d030661	3	1h	0.522
Zm00001d030775	1	1h	0.114
Zm00001d030851	2	1h	0.240
Zm00001d030877	2	1h	0.000
Zm00001d030942	3	1h	0.400
Zm00001d031189	1	1h	0.500
Zm00001d031230	3	1h	0.611
Zm00001d031454	5	1h	0.690
Zm00001d031465	1	1h	0.350
Zm00001d031533	5	1h	0.686
Zm00001d031717	3	1h	0.188
Zm00001d031730	3	1h	0.200
Zm00001d031782	5	1h	0.833
Zm00001d031858	4	1h	0.517
Zm00001d032274	5	1h	1.000
Zm00001d032324	5	1h	0.494
Zm00001d032433	4	1h	0.522
Zm00001d032439	3	1h	0.562
Zm00001d032496	2	1h	0.207
Zm00001d032587	2	1h	0.435
Zm00001d032616	1	1h	0.208
Zm00001d032858	5	1h	0.590
Zm00001d032870	3	1h	0.410
Zm00001d032978	3	1h	0.354
Zm00001d033068	3	1h	0.416
Zm00001d033139	2	1h	0.000
Zm00001d033374	4	1h	0.500

Transcripts	Cluster	Sample	Normalized max pausing score
Zm00001d033505	2	1h	0.325
Zm00001d033583	2	1h	0.500
Zm00001d033595	5	1h	0.714
Zm00001d033836	2	1h	0.260
Zm00001d033879	3	1h	0.388
Zm00001d034064	3	1h	0.127
Zm00001d034068	2	1h	0.237
Zm00001d034501	2	1h	0.114
Zm00001d034738	2	1h	0.312
Zm00001d034888	1	1h	1.000
Zm00001d035020	2	1h	0.383
Zm00001d035163	2	1h	0.211
Zm00001d035322	4	1h	0.000
Zm00001d035383	5	1h	0.560
Zm00001d035457	3	1h	1.000
Zm00001d035592	5	1h	0.480
Zm00001d035963	4	1h	0.354
Zm00001d036152	5	1h	0.747
Zm00001d036532	5	1h	0.545
Zm00001d036550	1	1h	0.364
Zm00001d036615	4	1h	0.250
Zm00001d036690	4	1h	0.000
Zm00001d036946	4	1h	0.348
Zm00001d037017	3	1h	0.480
Zm00001d037182	3	1h	0.519
Zm00001d037197	5	1h	0.694
Zm00001d037666	2	1h	0.382
Zm00001d037799	3	1h	0.189
Zm00001d037958	3	1h	0.286
Zm00001d038067	4	1h	0.414
Zm00001d038117	1	1h	0.181
Zm00001d038281	2	1h	0.230
Zm00001d038342	2	1h	0.181
Zm00001d038485	3	1h	0.420
Zm00001d038626	1	1h	0.783
Zm00001d038645	5	1h	1.000
Zm00001d038793	2	1h	0.214
Zm00001d038850	3	1h	0.334
Zm00001d038937	2	1h	0.180
Zm00001d038960	2	1h	0.142
Zm00001d039057	2	1h	0.000
Zm00001d039101	2	1h	0.000
Zm00001d039103	4	1h	0.596
Zm00001d039132	2	1h	0.000
Zm00001d039219	4	1h	0.446
Zm00001d039240	2	1h	0.425
Zm00001d039310	2	1h	0.453
Zm00001d039510	2	1h	0.000
Zm00001d039579	4	1h	0.524
Zm00001d039637	1	1h	0.642
Zm00001d039642	2	1h	0.182

Transcripts	Cluster	Sample	Normalized max pausing score
Zm00001d039694	2	1h	0.000
Zm00001d039946	1	1h	0.111
Zm00001d040047	1	1h	0.175
Zm00001d040112	2	1h	0.426
Zm00001d040220	4	1h	0.428
Zm00001d040541	5	1h	0.875
Zm00001d040544	3	1h	0.328
Zm00001d040628	3	1h	0.000
Zm00001d040702	2	1h	0.180
Zm00001d040724	3	1h	0.583
Zm00001d040743	4	1h	0.514
Zm00001d041774	1	1h	0.000
Zm00001d042169	5	1h	0.804
Zm00001d042508	2	1h	0.170
Zm00001d042676	1	1h	0.233
Zm00001d042765	5	1h	0.750
Zm00001d042766	5	1h	0.889
Zm00001d042801	3	1h	0.284
Zm00001d042909	4	1h	0.250
Zm00001d042936	2	1h	0.000
Zm00001d043145	2	1h	0.409
Zm00001d043234	4	1h	0.667
Zm00001d043293	4	1h	0.159
Zm00001d043339	2	1h	0.177
Zm00001d043391	4	1h	0.250
Zm00001d043400	2	1h	0.343
Zm00001d043558	4	1h	0.529
Zm00001d043596	2	1h	0.000
Zm00001d043702	3	1h	0.379
Zm00001d043751	2	1h	0.333
Zm00001d043870	3	1h	0.693
Zm00001d044052	5	1h	0.950
Zm00001d044059	3	1h	0.263
Zm00001d044202	3	1h	0.000
Zm00001d044417	2	1h	0.193
Zm00001d044515	5	1h	0.423
Zm00001d044597	5	1h	0.609
Zm00001d044802	3	1h	0.497
Zm00001d044874	2	1h	0.000
Zm00001d044895	3	1h	0.476
Zm00001d044911	2	1h	0.000
Zm00001d044918	3	1h	0.325
Zm00001d045370	2	1h	0.257
Zm00001d045495	4	1h	0.286
Zm00001d045515	3	1h	0.568
Zm00001d045598	5	1h	0.821
Zm00001d045755	3	1h	0.250
Zm00001d045788	2	1h	0.165
Zm00001d045888	5	1h	0.646
Zm00001d045913	3	1h	0.333
Zm00001d046383	2	1h	0.430

Transcripts	Cluster	Sample	Normalized max pausing score
Zm00001d046501	5	1h	0.487
Zm00001d046621	5	1h	0.562
Zm00001d046672	2	1h	0.206
Zm00001d046696	4	1h	0.000
Zm00001d046729	5	1h	0.606
Zm00001d046743	3	1h	0.286
Zm00001d046759	2	1h	0.000
Zm00001d046882	5	1h	0.486
Zm00001d046947	3	1h	0.321
Zm00001d046979	2	1h	0.280
Zm00001d047013	1	1h	0.750
Zm00001d047069	2	1h	0.000
Zm00001d047202	3	1h	0.510
Zm00001d047499	4	1h	0.600
Zm00001d047637	3	1h	1.000
Zm00001d047708	2	1h	0.000
Zm00001d047753	2	1h	0.000
Zm00001d047921	4	1h	0.000
Zm00001d047937	2	1h	0.146
Zm00001d048032	1	1h	0.000
Zm00001d048113	5	1h	0.600
Zm00001d048131	2	1h	0.188
Zm00001d048192	5	1h	0.625
Zm00001d048234	1	1h	0.364
Zm00001d048451	2	1h	0.298
Zm00001d048595	3	1h	0.000
Zm00001d048635	5	1h	0.691
Zm00001d048695	3	1h	0.667
Zm00001d048711	2	1h	0.071
Zm00001d048991	3	1h	0.349
Zm00001d049145	5	1h	0.667
Zm00001d049228	3	1h	0.366
Zm00001d049332	2	1h	0.414
Zm00001d049400	1	1h	0.469
Zm00001d049499	2	1h	0.285
Zm00001d049585	1	1h	0.400
Zm00001d049595	5	1h	1.000
Zm00001d050092	2	1h	0.230
Zm00001d050141	2	1h	0.000
Zm00001d050172	2	1h	0.150
Zm00001d050294	2	1h	0.000
Zm00001d050308	4	1h	0.500
Zm00001d050335	3	1h	0.362
Zm00001d050350	3	1h	0.348
Zm00001d050498	1	1h	0.543
Zm00001d050558	5	1h	0.841
Zm00001d050830	3	1h	0.518
Zm00001d051140	1	1h	0.000
Zm00001d051424	2	1h	0.000
Zm00001d051474	1	1h	0.667
Zm00001d051660	2	1h	0.224

Transcripts	Cluster	Sample	Normalized max pausing score
Zm00001d051672	3	1h	0.237
Zm00001d051788	4	1h	0.383
Zm00001d052209	3	1h	0.135
Zm00001d052212	2	1h	0.163
Zm00001d052239	2	1h	0.121
Zm00001d052385	2	1h	0.214
Zm00001d052471	3	1h	0.371
Zm00001d052618	2	1h	0.295
Zm00001d052701	2	1h	0.166
Zm00001d052847	3	1h	0.000
Zm00001d052933	3	1h	0.500
Zm00001d052944	2	1h	0.231
Zm00001d052981	4	1h	0.417
Zm00001d053017	2	1h	0.257
Zm00001d053156	3	1h	0.397
Zm00001d053244	3	1h	1.000
Zm00001d053306	3	1h	0.308
Zm00001d053346	5	1h	0.500
Zm00001d053695	2	1h	0.122
Zm00001d053826	2	1h	0.116
Zm00001d054009	2	1h	0.195
Zm00001d054071	2	1h	0.283
ZeamMp030	3	2h	0.906
ZeamMp033	5	2h	0.365
ZeamMp034	4	2h	0.630
ZeamMp041	4	2h	0.931
ZeamMp137	3	2h	0.205
Zm00001d000035	3	2h	0.346
Zm00001d000390	2	2h	0.119
Zm00001d002058	5	2h	0.189
Zm00001d002086	1	2h	0.522
Zm00001d002131	2	2h	0.222
Zm00001d002542	3	2h	0.490
Zm00001d002684	1	2h	0.314
Zm00001d002757	3	2h	0.365
Zm00001d002782	3	2h	0.410
Zm00001d002899	2	2h	0.349
Zm00001d003088	5	2h	0.317
Zm00001d003183	1	2h	0.349
Zm00001d003281	5	2h	0.476
Zm00001d003399	3	2h	0.233
Zm00001d003400	4	2h	0.571
Zm00001d003427	2	2h	0.268
Zm00001d003435	5	2h	0.415
Zm00001d003463	2	2h	0.245
Zm00001d003516	4	2h	0.781
Zm00001d003538	2	2h	0.333
Zm00001d003593	2	2h	0.094
Zm00001d003743	2	2h	0.248
Zm00001d003763	4	2h	0.540
Zm00001d004301	1	2h	0.167

Transcripts	Cluster	Sample	Normalized max pausing score
Zm00001d004310	1	2h	0.596
Zm00001d004910	4	2h	0.664
Zm00001d005109	5	2h	0.556
Zm00001d005480	3	2h	0.565
Zm00001d005504	3	2h	0.273
Zm00001d005612	2	2h	0.109
Zm00001d005680	4	2h	1.000
Zm00001d005909	3	2h	0.082
Zm00001d005936	2	2h	0.250
Zm00001d005962	2	2h	0.667
Zm00001d005989	3	2h	0.176
Zm00001d006000	2	2h	0.000
Zm00001d006011	3	2h	0.629
Zm00001d006045	3	2h	0.381
Zm00001d006132	3	2h	0.519
Zm00001d006193	4	2h	0.896
Zm00001d006321	2	2h	0.159
Zm00001d006619	3	2h	0.144
Zm00001d006631	1	2h	0.667
Zm00001d006638	5	2h	0.171
Zm00001d006894	5	2h	0.225
Zm00001d006947	4	2h	0.470
Zm00001d006950	1	2h	0.150
Zm00001d007015	1	2h	0.562
Zm00001d007050	2	2h	0.131
Zm00001d007162	3	2h	0.424
Zm00001d007197	2	2h	0.175
Zm00001d007258	2	2h	0.075
Zm00001d007259	5	2h	0.157
Zm00001d007294	3	2h	0.545
Zm00001d007478	4	2h	0.667
Zm00001d007503	5	2h	0.000
Zm00001d007518	1	2h	0.000
Zm00001d007606	4	2h	0.591
Zm00001d007839	2	2h	0.102
Zm00001d007869	2	2h	0.210
Zm00001d008187	4	2h	0.735
Zm00001d008219	2	2h	0.667
Zm00001d008297	2	2h	0.183
Zm00001d008298	5	2h	0.500
Zm00001d008329	2	2h	0.095
Zm00001d008409	5	2h	0.269
Zm00001d008764	2	2h	0.046
Zm00001d008827	3	2h	0.173
Zm00001d008859	3	2h	0.315
Zm00001d009008	4	2h	0.534
Zm00001d009108	2	2h	0.232
Zm00001d009138	4	2h	1.000
Zm00001d009336	3	2h	0.375
Zm00001d009568	2	2h	0.124
Zm00001d009747	4	2h	0.542

Transcripts	Cluster	Sample	Normalized max pausing score
Zm00001d009787	2	2h	0.228
Zm00001d010044	2	2h	0.152
Zm00001d010222	3	2h	0.500
Zm00001d010325	2	2h	0.253
Zm00001d010388	3	2h	0.000
Zm00001d010564	2	2h	0.069
Zm00001d010590	2	2h	0.174
Zm00001d010594	2	2h	0.333
Zm00001d010610	3	2h	0.600
Zm00001d010621	2	2h	0.000
Zm00001d010785	5	2h	0.500
Zm00001d010788	3	2h	0.297
Zm00001d010868	2	2h	0.229
Zm00001d010872	2	2h	0.257
Zm00001d010925	2	2h	0.000
Zm00001d011068	4	2h	0.425
Zm00001d011620	1	2h	0.000
Zm00001d011881	3	2h	0.185
Zm00001d011890	5	2h	0.395
Zm00001d011964	1	2h	0.058
Zm00001d012041	2	2h	0.000
Zm00001d012237	2	2h	0.284
Zm00001d012275	3	2h	0.303
Zm00001d012289	2	2h	0.189
Zm00001d012387	1	2h	0.000
Zm00001d012612	2	2h	0.220
Zm00001d012626	2	2h	0.000
Zm00001d012785	2	2h	0.333
Zm00001d012934	5	2h	0.482
Zm00001d013069	1	2h	0.237
Zm00001d013162	2	2h	0.155
Zm00001d013311	4	2h	0.828
Zm00001d013339	5	2h	0.229
Zm00001d013342	2	2h	0.222
Zm00001d013399	2	2h	0.222
Zm00001d013794	3	2h	0.182
Zm00001d013923	2	2h	0.277
Zm00001d014196	4	2h	0.667
Zm00001d014253	1	2h	1.000
Zm00001d014414	5	2h	0.263
Zm00001d014463	2	2h	0.160
Zm00001d014704	2	2h	0.128
Zm00001d014820	2	2h	0.035
Zm00001d014994	2	2h	0.131
Zm00001d015059	5	2h	0.362
Zm00001d015129	3	2h	0.593
Zm00001d015202	2	2h	0.000
Zm00001d015215	2	2h	0.135
Zm00001d015407	4	2h	0.645
Zm00001d015412	2	2h	0.188
Zm00001d015744	1	2h	0.333

Transcripts	Cluster	Sample	Normalized max pausing score
Zm00001d015779	4	2h	0.556
Zm00001d015884	3	2h	0.400
Zm00001d015990	4	2h	0.500
Zm00001d016154	5	2h	0.342
Zm00001d016262	2	2h	0.000
Zm00001d016301	5	2h	0.266
Zm00001d016322	3	2h	0.375
Zm00001d016417	2	2h	0.207
Zm00001d016648	5	2h	0.375
Zm00001d016806	5	2h	0.431
Zm00001d016831	4	2h	0.796
Zm00001d016844	5	2h	0.200
Zm00001d016896	3	2h	0.361
Zm00001d017351	3	2h	0.195
Zm00001d017353	1	2h	0.469
Zm00001d017462	4	2h	0.679
Zm00001d017530	2	2h	0.290
Zm00001d017696	2	2h	0.176
Zm00001d017746	2	2h	0.124
Zm00001d017851	3	2h	0.212
Zm00001d017991	2	2h	0.000
Zm00001d018058	3	2h	0.200
Zm00001d018081	2	2h	0.209
Zm00001d018117	1	2h	0.000
Zm00001d018133	4	2h	0.774
Zm00001d018191	1	2h	0.000
Zm00001d018475	4	2h	0.523
Zm00001d018696	3	2h	0.412
Zm00001d018806	2	2h	0.322
Zm00001d019002	2	2h	0.116
Zm00001d019400	3	2h	0.403
Zm00001d019422	5	2h	0.370
Zm00001d019582	5	2h	0.000
Zm00001d019669	1	2h	0.086
Zm00001d019925	5	2h	0.000
Zm00001d019989	3	2h	0.428
Zm00001d019990	2	2h	0.365
Zm00001d020176	5	2h	0.000
Zm00001d020277	4	2h	1.000
Zm00001d020403	2	2h	0.286
Zm00001d020425	3	2h	0.310
Zm00001d020497	1	2h	0.204
Zm00001d020610	2	2h	0.078
Zm00001d020620	2	2h	0.266
Zm00001d020651	5	2h	0.372
Zm00001d020909	3	2h	0.000
Zm00001d021024	5	2h	0.256
Zm00001d021216	2	2h	0.085
Zm00001d021294	2	2h	0.200
Zm00001d021338	2	2h	0.114
Zm00001d021439	3	2h	0.000

Transcripts	Cluster	Sample	Normalized max pausing score
Zm00001d021576	4	2h	0.738
Zm00001d021744	3	2h	0.493
Zm00001d021778	2	2h	0.347
Zm00001d021967	2	2h	0.130
Zm00001d021995	5	2h	0.236
Zm00001d022040	2	2h	0.199
Zm00001d022045	2	2h	0.294
Zm00001d022067	2	2h	0.000
Zm00001d022265	5	2h	0.369
Zm00001d022350	2	2h	0.000
Zm00001d022474	2	2h	0.206
Zm00001d022529	5	2h	0.225
Zm00001d023240	1	2h	0.374
Zm00001d023253	4	2h	1.000
Zm00001d023291	2	2h	0.175
Zm00001d023300	2	2h	0.083
Zm00001d023312	2	2h	0.405
Zm00001d023396	3	2h	0.373
Zm00001d023455	3	2h	0.343
Zm00001d023654	5	2h	0.334
Zm00001d023700	1	2h	0.260
Zm00001d023767	5	2h	0.315
Zm00001d024088	2	2h	0.477
Zm00001d024253	5	2h	0.333
Zm00001d024322	2	2h	0.154
Zm00001d024324	1	2h	0.523
Zm00001d024327	3	2h	0.090
Zm00001d024647	2	2h	0.177
Zm00001d024681	5	2h	0.500
Zm00001d024687	2	2h	0.185
Zm00001d024717	4	2h	1.000
Zm00001d024768	2	2h	0.000
Zm00001d024823	5	2h	0.510
Zm00001d024873	3	2h	0.000
Zm00001d025027	1	2h	0.031
Zm00001d025040	4	2h	1.000
Zm00001d025247	3	2h	0.293
Zm00001d025656	3	2h	0.232
Zm00001d025746	3	2h	0.462
Zm00001d025804	2	2h	0.113
Zm00001d025807	3	2h	0.189
Zm00001d025842	2	2h	0.132
Zm00001d026032	2	2h	0.000
Zm00001d026397	1	2h	0.184
Zm00001d026406	3	2h	0.500
Zm00001d026592	1	2h	1.000
Zm00001d027292	2	2h	0.261
Zm00001d027308	4	2h	0.847
Zm00001d027338	4	2h	0.352
Zm00001d027472	1	2h	0.174
Zm00001d027530	5	2h	0.000

Transcripts	Cluster	Sample	Normalized max pausing score
Zm00001d027622	3	2h	0.444
Zm00001d027673	3	2h	0.286
Zm00001d027741	2	2h	0.295
Zm00001d027751	3	2h	0.174
Zm00001d027946	5	2h	0.106
Zm00001d028004	3	2h	0.353
Zm00001d028025	2	2h	0.375
Zm00001d028073	2	2h	0.000
Zm00001d028362	4	2h	0.494
Zm00001d028427	2	2h	0.164
Zm00001d028447	5	2h	0.207
Zm00001d028615	2	2h	0.000
Zm00001d028697	4	2h	0.873
Zm00001d028714	2	2h	0.261
Zm00001d028835	5	2h	0.190
Zm00001d028899	2	2h	0.000
Zm00001d028925	2	2h	0.000
Zm00001d029059	3	2h	0.308
Zm00001d029241	3	2h	0.134
Zm00001d029257	4	2h	1.000
Zm00001d029402	2	2h	0.286
Zm00001d029427	2	2h	0.000
Zm00001d029579	2	2h	0.384
Zm00001d029676	5	2h	0.429
Zm00001d029921	4	2h	0.429
Zm00001d029950	4	2h	0.517
Zm00001d029969	2	2h	0.267
Zm00001d030016	4	2h	0.875
Zm00001d030299	5	2h	0.347
Zm00001d030305	2	2h	0.286
Zm00001d030470	3	2h	0.258
Zm00001d030661	3	2h	0.348
Zm00001d030775	1	2h	0.046
Zm00001d030851	2	2h	0.186
Zm00001d030877	2	2h	0.000
Zm00001d030942	3	2h	0.500
Zm00001d031189	1	2h	0.000
Zm00001d031230	3	2h	0.612
Zm00001d031454	5	2h	0.345
Zm00001d031465	1	2h	0.429
Zm00001d031533	5	2h	0.324
Zm00001d031717	3	2h	0.219
Zm00001d031730	3	2h	0.130
Zm00001d031782	5	2h	0.000
Zm00001d031858	4	2h	0.775
Zm00001d032274	5	2h	0.500
Zm00001d032324	5	2h	0.212
Zm00001d032433	4	2h	0.523
Zm00001d032439	3	2h	0.375
Zm00001d032496	2	2h	0.216
Zm00001d032587	2	2h	0.145

Transcripts	Cluster	Sample	Normalized max pausing score
Zm00001d032616	1	2h	0.000
Zm00001d032858	5	2h	0.000
Zm00001d032870	3	2h	0.456
Zm00001d032978	3	2h	0.354
Zm00001d033068	3	2h	0.652
Zm00001d033139	2	2h	0.500
Zm00001d033374	4	2h	0.500
Zm00001d033505	2	2h	0.213
Zm00001d033583	2	2h	0.000
Zm00001d033595	5	2h	0.357
Zm00001d033836	2	2h	0.190
Zm00001d033879	3	2h	0.194
Zm00001d034064	3	2h	0.205
Zm00001d034068	2	2h	0.263
Zm00001d034501	2	2h	0.163
Zm00001d034738	2	2h	0.182
Zm00001d034888	1	2h	0.000
Zm00001d035020	2	2h	0.209
Zm00001d035163	2	2h	0.211
Zm00001d035322	4	2h	0.744
Zm00001d035383	5	2h	0.373
Zm00001d035457	3	2h	0.000
Zm00001d035592	5	2h	0.270
Zm00001d035963	4	2h	0.576
Zm00001d036152	5	2h	0.373
Zm00001d036532	5	2h	0.390
Zm00001d036550	1	2h	0.182
Zm00001d036615	4	2h	1.000
Zm00001d036690	4	2h	1.000
Zm00001d036946	4	2h	0.697
Zm00001d037017	3	2h	0.373
Zm00001d037182	3	2h	0.415
Zm00001d037197	5	2h	0.000
Zm00001d037666	2	2h	0.233
Zm00001d037799	3	2h	0.325
Zm00001d037958	3	2h	0.429
Zm00001d038067	4	2h	0.690
Zm00001d038117	1	2h	0.163
Zm00001d038281	2	2h	0.250
Zm00001d038342	2	2h	0.181
Zm00001d038485	3	2h	0.512
Zm00001d038626	1	2h	0.522
Zm00001d038645	5	2h	0.500
Zm00001d038793	2	2h	0.000
Zm00001d038850	3	2h	0.275
Zm00001d038937	2	2h	0.189
Zm00001d038960	2	2h	0.085
Zm00001d039057	2	2h	0.000
Zm00001d039101	2	2h	0.000
Zm00001d039103	4	2h	0.937
Zm00001d039132	2	2h	0.600

Transcripts	Cluster	Sample	Normalized max pausing score
Zm00001d039219	4	2h	0.744
Zm00001d039240	2	2h	0.181
Zm00001d039310	2	2h	0.302
Zm00001d039510	2	2h	0.000
Zm00001d039579	4	2h	0.838
Zm00001d039637	1	2h	0.474
Zm00001d039642	2	2h	0.333
Zm00001d039694	2	2h	0.000
Zm00001d039946	1	2h	0.070
Zm00001d040047	1	2h	0.130
Zm00001d040112	2	2h	0.213
Zm00001d040220	4	2h	0.534
Zm00001d040541	5	2h	0.194
Zm00001d040544	3	2h	0.377
Zm00001d040628	3	2h	0.000
Zm00001d040702	2	2h	0.225
Zm00001d040724	3	2h	0.292
Zm00001d040743	4	2h	0.685
Zm00001d041774	1	2h	0.716
Zm00001d042169	5	2h	0.321
Zm00001d042508	2	2h	0.140
Zm00001d042676	1	2h	0.121
Zm00001d042765	5	2h	0.222
Zm00001d042766	5	2h	0.556
Zm00001d042801	3	2h	0.568
Zm00001d042909	4	2h	0.389
Zm00001d042936	2	2h	0.000
Zm00001d043145	2	2h	0.160
Zm00001d043234	4	2h	0.667
Zm00001d043293	4	2h	0.420
Zm00001d043339	2	2h	0.138
Zm00001d043391	4	2h	0.500
Zm00001d043400	2	2h	0.381
Zm00001d043558	4	2h	0.423
Zm00001d043596	2	2h	0.520
Zm00001d043702	3	2h	0.542
Zm00001d043751	2	2h	0.400
Zm00001d043870	3	2h	0.389
Zm00001d044052	5	2h	0.452
Zm00001d044059	3	2h	0.269
Zm00001d044202	3	2h	0.429
Zm00001d044417	2	2h	0.100
Zm00001d044515	5	2h	0.387
Zm00001d044597	5	2h	0.187
Zm00001d044802	3	2h	0.239
Zm00001d044874	2	2h	0.000
Zm00001d044895	3	2h	0.236
Zm00001d044911	2	2h	0.000
Zm00001d044918	3	2h	0.346
Zm00001d045370	2	2h	0.271
Zm00001d045495	4	2h	0.300

Transcripts	Cluster	Sample	Normalized max pausing score
Zm00001d045515	3	2h	0.459
Zm00001d045598	5	2h	0.274
Zm00001d045755	3	2h	0.294
Zm00001d045788	2	2h	0.187
Zm00001d045888	5	2h	0.316
Zm00001d045913	3	2h	0.625
Zm00001d046383	2	2h	0.135
Zm00001d046501	5	2h	0.295
Zm00001d046621	5	2h	0.147
Zm00001d046672	2	2h	0.283
Zm00001d046696	4	2h	0.500
Zm00001d046729	5	2h	0.227
Zm00001d046743	3	2h	0.085
Zm00001d046759	2	2h	0.211
Zm00001d046882	5	2h	0.458
Zm00001d046947	3	2h	0.250
Zm00001d046979	2	2h	0.407
Zm00001d047013	1	2h	0.000
Zm00001d047069	2	2h	0.000
Zm00001d047202	3	2h	0.213
Zm00001d047499	4	2h	1.000
Zm00001d047637	3	2h	0.750
Zm00001d047708	2	2h	0.000
Zm00001d047753	2	2h	0.000
Zm00001d047921	4	2h	1.000
Zm00001d047937	2	2h	0.129
Zm00001d048032	1	2h	0.000
Zm00001d048113	5	2h	0.185
Zm00001d048131	2	2h	0.000
Zm00001d048192	5	2h	0.000
Zm00001d048234	1	2h	1.000
Zm00001d048451	2	2h	0.126
Zm00001d048595	3	2h	0.600
Zm00001d048635	5	2h	0.519
Zm00001d048695	3	2h	0.500
Zm00001d048711	2	2h	0.023
Zm00001d048991	3	2h	0.611
Zm00001d049145	5	2h	0.308
Zm00001d049228	3	2h	0.389
Zm00001d049332	2	2h	0.233
Zm00001d049400	1	2h	0.321
Zm00001d049499	2	2h	0.305
Zm00001d049585	1	2h	0.333
Zm00001d049595	5	2h	0.294
Zm00001d050092	2	2h	0.168
Zm00001d050141	2	2h	0.000
Zm00001d050172	2	2h	0.287
Zm00001d050294	2	2h	0.571
Zm00001d050308	4	2h	1.000
Zm00001d050335	3	2h	0.337
Zm00001d050350	3	2h	0.348

Transcripts	Cluster	Sample	Normalized max pausing score
Zm00001d050498	1	2h	0.000
Zm00001d050558	5	2h	0.000
Zm00001d050830	3	2h	0.740
Zm00001d051140	1	2h	0.000
Zm00001d051424	2	2h	0.000
Zm00001d051474	1	2h	0.200
Zm00001d051660	2	2h	0.139
Zm00001d051672	3	2h	0.200
Zm00001d051788	4	2h	0.502
Zm00001d052209	3	2h	0.387
Zm00001d052212	2	2h	0.104
Zm00001d052239	2	2h	0.151
Zm00001d052385	2	2h	0.321
Zm00001d052471	3	2h	0.278
Zm00001d052618	2	2h	0.187
Zm00001d052701	2	2h	0.132
Zm00001d052847	3	2h	0.554
Zm00001d052933	3	2h	0.359
Zm00001d052944	2	2h	0.433
Zm00001d052981	4	2h	1.000
Zm00001d053017	2	2h	0.154
Zm00001d053156	3	2h	0.156
Zm00001d053244	3	2h	0.700
Zm00001d053306	3	2h	0.187
Zm00001d053346	5	2h	0.000
Zm00001d053695	2	2h	0.174
Zm00001d053826	2	2h	0.152
Zm00001d054009	2	2h	0.293
Zm00001d054071	2	2h	0.438
ZeamMp030	3	4h	0.865
ZeamMp033	5	4h	0.069
ZeamMp034	4	4h	1.000
ZeamMp041	4	4h	0.368
ZeamMp137	3	4h	0.059
Zm00001d000035	3	4h	0.310
Zm00001d000390	2	4h	0.135
Zm00001d002058	5	4h	0.103
Zm00001d002086	1	4h	0.348
Zm00001d002131	2	4h	0.370
Zm00001d002542	3	4h	0.387
Zm00001d002684	1	4h	0.248
Zm00001d002757	3	4h	0.167
Zm00001d002782	3	4h	0.154
Zm00001d002899	2	4h	0.349
Zm00001d003088	5	4h	0.490
Zm00001d003183	1	4h	0.000
Zm00001d003281	5	4h	0.159
Zm00001d003399	3	4h	0.300
Zm00001d003400	4	4h	0.190
Zm00001d003427	2	4h	0.127
Zm00001d003435	5	4h	0.207

Transcripts	Cluster	Sample	Normalized max pausing score
Zm00001d003463	2	4h	0.000
Zm00001d003516	4	4h	0.149
Zm00001d003538	2	4h	0.500
Zm00001d003593	2	4h	0.168
Zm00001d003743	2	4h	0.267
Zm00001d003763	4	4h	0.540
Zm00001d004301	1	4h	0.176
Zm00001d004310	1	4h	0.000
Zm00001d004910	4	4h	0.609
Zm00001d005109	5	4h	0.278
Zm00001d005480	3	4h	0.283
Zm00001d005504	3	4h	0.333
Zm00001d005612	2	4h	0.191
Zm00001d005680	4	4h	1.000
Zm00001d005909	3	4h	0.171
Zm00001d005936	2	4h	0.200
Zm00001d005962	2	4h	0.000
Zm00001d005989	3	4h	0.192
Zm00001d006000	2	4h	0.000
Zm00001d006011	3	4h	0.509
Zm00001d006045	3	4h	0.327
Zm00001d006132	3	4h	0.502
Zm00001d006193	4	4h	0.000
Zm00001d006321	2	4h	0.148
Zm00001d006619	3	4h	0.377
Zm00001d006631	1	4h	0.667
Zm00001d006638	5	4h	0.182
Zm00001d006894	5	4h	0.268
Zm00001d006947	4	4h	0.448
Zm00001d006950	1	4h	0.086
Zm00001d007015	1	4h	0.562
Zm00001d007050	2	4h	0.261
Zm00001d007162	3	4h	0.848
Zm00001d007197	2	4h	0.306
Zm00001d007258	2	4h	0.103
Zm00001d007259	5	4h	0.168
Zm00001d007294	3	4h	0.156
Zm00001d007478	4	4h	0.250
Zm00001d007503	5	4h	0.000
Zm00001d007518	1	4h	0.500
Zm00001d007606	4	4h	0.788
Zm00001d007839	2	4h	0.199
Zm00001d007869	2	4h	0.262
Zm00001d008187	4	4h	0.735
Zm00001d008219	2	4h	0.000
Zm00001d008297	2	4h	0.127
Zm00001d008298	5	4h	0.500
Zm00001d008329	2	4h	0.111
Zm00001d008409	5	4h	0.251
Zm00001d008764	2	4h	0.052
Zm00001d008827	3	4h	0.115

Transcripts	Cluster	Sample	Normalized max pausing score
Zm00001d008859	3	4h	0.192
Zm00001d009008	4	4h	0.322
Zm00001d009108	2	4h	0.358
Zm00001d009138	4	4h	0.500
Zm00001d009336	3	4h	0.188
Zm00001d009568	2	4h	0.158
Zm00001d009747	4	4h	0.271
Zm00001d009787	2	4h	0.293
Zm00001d010044	2	4h	0.155
Zm00001d010222	3	4h	1.000
Zm00001d010325	2	4h	0.207
Zm00001d010388	3	4h	0.000
Zm00001d010564	2	4h	0.060
Zm00001d010590	2	4h	0.110
Zm00001d010594	2	4h	0.333
Zm00001d010610	3	4h	0.325
Zm00001d010621	2	4h	0.000
Zm00001d010785	5	4h	0.333
Zm00001d010788	3	4h	0.594
Zm00001d010868	2	4h	0.121
Zm00001d010872	2	4h	0.240
Zm00001d010925	2	4h	0.000
Zm00001d011068	4	4h	0.600
Zm00001d011620	1	4h	0.545
Zm00001d011881	3	4h	0.683
Zm00001d011890	5	4h	0.593
Zm00001d011964	1	4h	0.044
Zm00001d012041	2	4h	0.000
Zm00001d012237	2	4h	0.455
Zm00001d012275	3	4h	0.145
Zm00001d012289	2	4h	0.347
Zm00001d012387	1	4h	0.000
Zm00001d012612	2	4h	0.151
Zm00001d012626	2	4h	0.000
Zm00001d012785	2	4h	0.000
Zm00001d012934	5	4h	0.321
Zm00001d013069	1	4h	0.369
Zm00001d013162	2	4h	0.128
Zm00001d013311	4	4h	0.000
Zm00001d013339	5	4h	0.265
Zm00001d013342	2	4h	0.200
Zm00001d013399	2	4h	0.198
Zm00001d013794	3	4h	0.500
Zm00001d013923	2	4h	0.145
Zm00001d014196	4	4h	0.444
Zm00001d014253	1	4h	0.000
Zm00001d014414	5	4h	0.519
Zm00001d014463	2	4h	0.194
Zm00001d014704	2	4h	0.364
Zm00001d014820	2	4h	0.022
Zm00001d014994	2	4h	0.148

Transcripts	Cluster	Sample	Normalized max pausing score
Zm00001d015059	5	4h	0.363
Zm00001d015129	3	4h	0.444
Zm00001d015202	2	4h	0.400
Zm00001d015215	2	4h	0.149
Zm00001d015407	4	4h	0.414
Zm00001d015412	2	4h	0.174
Zm00001d015744	1	4h	0.256
Zm00001d015779	4	4h	1.000
Zm00001d015884	3	4h	0.143
Zm00001d015990	4	4h	0.700
Zm00001d016154	5	4h	0.185
Zm00001d016262	2	4h	0.000
Zm00001d016301	5	4h	0.163
Zm00001d016322	3	4h	0.500
Zm00001d016417	2	4h	0.103
Zm00001d016648	5	4h	0.500
Zm00001d016806	5	4h	0.296
Zm00001d016831	4	4h	0.398
Zm00001d016844	5	4h	0.286
Zm00001d016896	3	4h	0.293
Zm00001d017351	3	4h	0.354
Zm00001d017353	1	4h	0.235
Zm00001d017462	4	4h	0.594
Zm00001d017530	2	4h	0.145
Zm00001d017696	2	4h	0.137
Zm00001d017746	2	4h	0.080
Zm00001d017851	3	4h	0.000
Zm00001d017991	2	4h	0.000
Zm00001d018058	3	4h	0.286
Zm00001d018081	2	4h	0.039
Zm00001d018117	1	4h	0.000
Zm00001d018133	4	4h	1.000
Zm00001d018191	1	4h	0.260
Zm00001d018475	4	4h	0.523
Zm00001d018696	3	4h	0.147
Zm00001d018806	2	4h	0.190
Zm00001d019002	2	4h	0.463
Zm00001d019400	3	4h	0.537
Zm00001d019422	5	4h	0.278
Zm00001d019582	5	4h	0.333
Zm00001d019669	1	4h	0.084
Zm00001d019925	5	4h	0.000
Zm00001d019989	3	4h	0.440
Zm00001d019990	2	4h	0.274
Zm00001d020176	5	4h	0.506
Zm00001d020277	4	4h	0.478
Zm00001d020403	2	4h	0.222
Zm00001d020425	3	4h	0.495
Zm00001d020497	1	4h	0.070
Zm00001d020610	2	4h	0.040
Zm00001d020620	2	4h	0.207

Transcripts	Cluster	Sample	Normalized max pausing score
Zm00001d020651	5	4h	0.279
Zm00001d020909	3	4h	0.000
Zm00001d021024	5	4h	0.205
Zm00001d021216	2	4h	0.079
Zm00001d021294	2	4h	0.000
Zm00001d021338	2	4h	0.230
Zm00001d021439	3	4h	0.500
Zm00001d021576	4	4h	0.410
Zm00001d021744	3	4h	0.192
Zm00001d021778	2	4h	0.304
Zm00001d021967	2	4h	0.142
Zm00001d021995	5	4h	0.393
Zm00001d022040	2	4h	0.259
Zm00001d022045	2	4h	0.239
Zm00001d022067	2	4h	0.500
Zm00001d022265	5	4h	0.209
Zm00001d022350	2	4h	0.694
Zm00001d022474	2	4h	0.291
Zm00001d022529	5	4h	0.196
Zm00001d023240	1	4h	0.500
Zm00001d023253	4	4h	0.250
Zm00001d023291	2	4h	0.244
Zm00001d023300	2	4h	0.099
Zm00001d023312	2	4h	0.360
Zm00001d023396	3	4h	0.373
Zm00001d023455	3	4h	0.381
Zm00001d023654	5	4h	0.371
Zm00001d023700	1	4h	0.000
Zm00001d023767	5	4h	0.630
Zm00001d024088	2	4h	0.298
Zm00001d024253	5	4h	0.143
Zm00001d024322	2	4h	0.217
Zm00001d024324	1	4h	0.348
Zm00001d024327	3	4h	0.143
Zm00001d024647	2	4h	0.379
Zm00001d024681	5	4h	0.236
Zm00001d024687	2	4h	0.167
Zm00001d024717	4	4h	0.000
Zm00001d024768	2	4h	0.381
Zm00001d024823	5	4h	0.191
Zm00001d024873	3	4h	0.667
Zm00001d025027	1	4h	0.651
Zm00001d025040	4	4h	0.000
Zm00001d025247	3	4h	0.138
Zm00001d025656	3	4h	0.317
Zm00001d025746	3	4h	0.222
Zm00001d025804	2	4h	0.112
Zm00001d025807	3	4h	0.195
Zm00001d025842	2	4h	0.192
Zm00001d026032	2	4h	0.206
Zm00001d026397	1	4h	0.099

Transcripts	Cluster	Sample	Normalized max pausing score
Zm00001d026406	3	4h	0.500
Zm00001d026592	1	4h	0.000
Zm00001d027292	2	4h	0.352
Zm00001d027308	4	4h	0.415
Zm00001d027338	4	4h	0.528
Zm00001d027472	1	4h	0.246
Zm00001d027530	5	4h	0.414
Zm00001d027622	3	4h	0.000
Zm00001d027673	3	4h	1.000
Zm00001d027741	2	4h	0.314
Zm00001d027751	3	4h	0.286
Zm00001d027946	5	4h	0.106
Zm00001d028004	3	4h	0.219
Zm00001d028025	2	4h	0.118
Zm00001d028073	2	4h	0.369
Zm00001d028362	4	4h	0.556
Zm00001d028427	2	4h	0.094
Zm00001d028447	5	4h	0.148
Zm00001d028615	2	4h	0.000
Zm00001d028697	4	4h	0.727
Zm00001d028714	2	4h	0.143
Zm00001d028835	5	4h	0.000
Zm00001d028899	2	4h	0.000
Zm00001d028925	2	4h	0.000
Zm00001d029059	3	4h	0.204
Zm00001d029241	3	4h	0.113
Zm00001d029257	4	4h	0.150
Zm00001d029402	2	4h	0.103
Zm00001d029427	2	4h	0.000
Zm00001d029579	2	4h	0.329
Zm00001d029676	5	4h	0.333
Zm00001d029921	4	4h	0.918
Zm00001d029950	4	4h	0.414
Zm00001d029969	2	4h	0.250
Zm00001d030016	4	4h	0.600
Zm00001d030299	5	4h	0.434
Zm00001d030305	2	4h	0.175
Zm00001d030470	3	4h	0.387
Zm00001d030661	3	4h	0.190
Zm00001d030775	1	4h	0.051
Zm00001d030851	2	4h	0.248
Zm00001d030877	2	4h	0.000
Zm00001d030942	3	4h	0.500
Zm00001d031189	1	4h	0.000
Zm00001d031230	3	4h	0.306
Zm00001d031454	5	4h	0.518
Zm00001d031465	1	4h	0.000
Zm00001d031533	5	4h	0.515
Zm00001d031717	3	4h	0.113
Zm00001d031730	3	4h	0.081
Zm00001d031782	5	4h	0.667

Transcripts	Cluster	Sample	Normalized max pausing score
Zm00001d031858	4	4h	0.564
Zm00001d032274	5	4h	0.714
Zm00001d032324	5	4h	0.846
Zm00001d032433	4	4h	0.524
Zm00001d032439	3	4h	0.221
Zm00001d032496	2	4h	0.140
Zm00001d032587	2	4h	0.258
Zm00001d032616	1	4h	0.000
Zm00001d032858	5	4h	0.000
Zm00001d032870	3	4h	0.547
Zm00001d032978	3	4h	0.354
Zm00001d033068	3	4h	0.000
Zm00001d033139	2	4h	0.000
Zm00001d033374	4	4h	0.333
Zm00001d033505	2	4h	0.302
Zm00001d033583	2	4h	0.000
Zm00001d033595	5	4h	0.278
Zm00001d033836	2	4h	0.257
Zm00001d033879	3	4h	0.420
Zm00001d034064	3	4h	0.123
Zm00001d034068	2	4h	0.356
Zm00001d034501	2	4h	0.184
Zm00001d034738	2	4h	0.312
Zm00001d034888	1	4h	0.500
Zm00001d035020	2	4h	0.287
Zm00001d035163	2	4h	0.290
Zm00001d035322	4	4h	0.372
Zm00001d035383	5	4h	0.000
Zm00001d035457	3	4h	0.000
Zm00001d035592	5	4h	0.648
Zm00001d035963	4	4h	0.128
Zm00001d036152	5	4h	0.280
Zm00001d036532	5	4h	0.182
Zm00001d036550	1	4h	0.273
Zm00001d036615	4	4h	0.333
Zm00001d036690	4	4h	0.500
Zm00001d036946	4	4h	0.000
Zm00001d037017	3	4h	0.299
Zm00001d037182	3	4h	0.000
Zm00001d037197	5	4h	0.000
Zm00001d037666	2	4h	0.212
Zm00001d037799	3	4h	0.505
Zm00001d037958	3	4h	0.762
Zm00001d038067	4	4h	0.518
Zm00001d038117	1	4h	0.272
Zm00001d038281	2	4h	0.172
Zm00001d038342	2	4h	0.085
Zm00001d038485	3	4h	0.262
Zm00001d038626	1	4h	0.232
Zm00001d038645	5	4h	0.000
Zm00001d038793	2	4h	1.000

Transcripts	Cluster	Sample	Normalized max pausing score
Zm00001d038850	3	4h	0.234
Zm00001d038937	2	4h	0.286
Zm00001d038960	2	4h	0.185
Zm00001d039057	2	4h	0.000
Zm00001d039101	2	4h	0.571
Zm00001d039103	4	4h	0.170
Zm00001d039132	2	4h	0.000
Zm00001d039219	4	4h	0.558
Zm00001d039240	2	4h	0.103
Zm00001d039310	2	4h	0.101
Zm00001d039510	2	4h	0.500
Zm00001d039579	4	4h	0.299
Zm00001d039637	1	4h	0.274
Zm00001d039642	2	4h	0.000
Zm00001d039694	2	4h	0.000
Zm00001d039946	1	4h	0.091
Zm00001d040047	1	4h	0.260
Zm00001d040112	2	4h	0.201
Zm00001d040220	4	4h	0.214
Zm00001d040541	5	4h	0.389
Zm00001d040544	3	4h	0.419
Zm00001d040628	3	4h	0.429
Zm00001d040702	2	4h	0.163
Zm00001d040724	3	4h	0.233
Zm00001d040743	4	4h	0.514
Zm00001d041774	1	4h	0.000
Zm00001d042169	5	4h	0.000
Zm00001d042508	2	4h	0.099
Zm00001d042676	1	4h	0.217
Zm00001d042765	5	4h	0.500
Zm00001d042766	5	4h	0.185
Zm00001d042801	3	4h	0.284
Zm00001d042909	4	4h	0.583
Zm00001d042936	2	4h	0.000
Zm00001d043145	2	4h	0.245
Zm00001d043234	4	4h	0.667
Zm00001d043293	4	4h	0.630
Zm00001d043339	2	4h	0.124
Zm00001d043391	4	4h	1.000
Zm00001d043400	2	4h	0.000
Zm00001d043558	4	4h	0.705
Zm00001d043596	2	4h	0.000
Zm00001d043702	3	4h	0.722
Zm00001d043751	2	4h	0.000
Zm00001d043870	3	4h	0.587
Zm00001d044052	5	4h	0.362
Zm00001d044059	3	4h	0.698
Zm00001d044202	3	4h	0.182
Zm00001d044417	2	4h	0.148
Zm00001d044515	5	4h	0.291
Zm00001d044597	5	4h	0.609

Transcripts	Cluster	Sample	Normalized max pausing score
Zm00001d044802	3	4h	0.135
Zm00001d044874	2	4h	0.000
Zm00001d044895	3	4h	0.698
Zm00001d044911	2	4h	0.000
Zm00001d044918	3	4h	0.000
Zm00001d045370	2	4h	0.000
Zm00001d045495	4	4h	0.667
Zm00001d045515	3	4h	0.331
Zm00001d045598	5	4h	0.365
Zm00001d045755	3	4h	0.333
Zm00001d045788	2	4h	0.231
Zm00001d045888	5	4h	0.554
Zm00001d045913	3	4h	0.500
Zm00001d046383	2	4h	0.175
Zm00001d046501	5	4h	0.177
Zm00001d046621	5	4h	0.450
Zm00001d046672	2	4h	0.104
Zm00001d046696	4	4h	0.500
Zm00001d046729	5	4h	0.568
Zm00001d046743	3	4h	0.343
Zm00001d046759	2	4h	0.182
Zm00001d046882	5	4h	0.214
Zm00001d046947	3	4h	0.214
Zm00001d046979	2	4h	0.373
Zm00001d047013	1	4h	0.000
Zm00001d047069	2	4h	0.262
Zm00001d047202	3	4h	0.255
Zm00001d047499	4	4h	0.500
Zm00001d047637	3	4h	0.500
Zm00001d047708	2	4h	0.333
Zm00001d047753	2	4h	0.667
Zm00001d047921	4	4h	0.333
Zm00001d047937	2	4h	0.208
Zm00001d048032	1	4h	0.133
Zm00001d048113	5	4h	0.150
Zm00001d048131	2	4h	0.000
Zm00001d048192	5	4h	0.333
Zm00001d048234	1	4h	0.333
Zm00001d048451	2	4h	0.129
Zm00001d048595	3	4h	0.000
Zm00001d048635	5	4h	0.440
Zm00001d048695	3	4h	0.333
Zm00001d048711	2	4h	0.038
Zm00001d048991	3	4h	0.000
Zm00001d049145	5	4h	0.342
Zm00001d049228	3	4h	0.346
Zm00001d049332	2	4h	0.310
Zm00001d049400	1	4h	0.161
Zm00001d049499	2	4h	0.184
Zm00001d049585	1	4h	0.000
Zm00001d049595	5	4h	0.500

Transcripts	Cluster	Sample	Normalized max pausing score
Zm00001d050092	2	4h	0.160
Zm00001d050141	2	4h	0.000
Zm00001d050172	2	4h	0.246
Zm00001d050294	2	4h	0.000
Zm00001d050308	4	4h	0.517
Zm00001d050335	3	4h	0.259
Zm00001d050350	3	4h	0.523
Zm00001d050498	1	4h	0.000
Zm00001d050558	5	4h	0.420
Zm00001d050830	3	4h	0.000
Zm00001d051140	1	4h	0.000
Zm00001d051424	2	4h	0.000
Zm00001d051474	1	4h	0.000
Zm00001d051660	2	4h	0.171
Zm00001d051672	3	4h	0.206
Zm00001d051788	4	4h	0.574
Zm00001d052209	3	4h	0.290
Zm00001d052212	2	4h	0.000
Zm00001d052239	2	4h	0.282
Zm00001d052385	2	4h	0.321
Zm00001d052471	3	4h	0.223
Zm00001d052618	2	4h	0.132
Zm00001d052701	2	4h	0.273
Zm00001d052847	3	4h	0.443
Zm00001d052933	3	4h	0.500
Zm00001d052944	2	4h	0.257
Zm00001d052981	4	4h	0.200
Zm00001d053017	2	4h	0.343
Zm00001d053156	3	4h	0.238
Zm00001d053244	3	4h	0.334
Zm00001d053306	3	4h	0.473
Zm00001d053346	5	4h	0.400
Zm00001d053695	2	4h	0.260
Zm00001d053826	2	4h	0.286
Zm00001d054009	2	4h	0.521
Zm00001d054071	2	4h	0.282
