# Supplementary material for: Ribosome Pausing Negatively Regulates Protein Translation in Maize Seedlings during Dark-to-Light Transitions
Source: Int J Mol Sci. 2024 Jul 22;25(14):7985. doi: 10.3390/ijms25147985 (PMC11277263; doi:10.3390/ijms25147985)
Supplement: Supplementary file 1 [file ijms-25-07985-s001.zip › Table S3.pdf]

Table S3 Gene ontology analysis of ribosome paused transcripts

GO Term	Genome	Count	Expected	Fold Enrichment	P-value	FDR	Term	Cluster
negative regulation of long-day photoperiodism, flowering (GO:0048579)	3	1	0	> 100	4.86E-03	1.00E+00	BP	1
histone H3-K36 methylation (GO:0010452)	4	1	0	> 100	6.07E-03	1.00E+00	BP	1
regulation of stomatal opening (GO:1902456)	5	1	0.01	> 100	7.28E-03	1.00E+00	BP	1
nuclear pore organization (GO:0006999)	6	1	0.01	> 100	8.49E-03	1.00E+00	BP	1
meiotic spindle organization (GO:0000212)	7	1	0.01	> 100	9.70E-03	1.00E+00	BP	1
positive regulation of organ growth (GO:0046622)	8	1	0.01	> 100	1.09E-02	1.00E+00	BP	1
positive regulation of developmental growth (GO:0048639)	11	1	0.01	74.6	1.45E-02	1.00E+00	BP	1
negative regulation of reproductive process (GO:2000242)	12	1	0.01	68.38	1.57E-02	1.00E+00	BP	1
meiotic DNA double-strand break formation (GO:0042138)	13	1	0.02	63.12	1.69E-02	1.00E+00	BP	1
negative regulation of post-embryonic development (GO:0048581)	13	1	0.02	63.12	1.69E-02	1.00E+00	BP	1
regulation of organ growth (GO:0046620)	16	1	0.02	51.29	2.05E-02	1.00E+00	BP	1
gibberellic acid mediated signaling pathway (GO:0009740)	17	1	0.02	48.27	2.17E-02	1.00E+00	BP	1
(1->3)-beta-D-glucan biosynthetic process (GO:0006075)	18	1	0.02	45.59	2.29E-02	1.00E+00	BP	1
(1->3)-beta-D-glucan metabolic process (GO:0006074)	18	1	0.02	45.59	2.29E-02	1.00E+00	BP	1
regulation of long-day photoperiodism, flowering (GO:0048586)	19	1	0.02	43.19	2.41E-02	1.00E+00	BP	1
regulation of stomatal movement (GO:0010119)	20	1	0.02	41.03	2.53E-02	1.00E+00	BP	1
gibberellin mediated signaling pathway (GO:0010476)	20	1	0.02	41.03	2.53E-02	1.00E+00	BP	1
male meiotic nuclear division (GO:0007140)	21	1	0.03	39.08	2.64E-02	1.00E+00	BP	1
negative regulation of multicellular organismal process (GO:0051241)	22	1	0.03	37.3	2.76E-02	1.00E+00	BP	1
cellular response to gibberellin stimulus (GO:0071370)	22	1	0.03	37.3	2.76E-02	1.00E+00	BP	1
regulation of cell shape (GO:0008360)	23	1	0.03	35.68	2.88E-02	1.00E+00	BP	1
negative regulation of developmental process (GO:0051093)	26	1	0.03	31.56	3.24E-02	1.00E+00	BP	1
phosphate ion transport (GO:0006817)	26	1	0.03	31.56	3.24E-02	1.00E+00	BP	1
male gamete generation (GO:0048232)	27	1	0.03	30.39	3.35E-02	1.00E+00	BP	1
cellular process involved in reproduction in multicellular organism (GO:0022412)	27	1	0.03	30.39	3.35E-02	1.00E+00	BP	1
nucleus organization (GO:0006997)	28	1	0.03	29.31	3.47E-02	1.00E+00	BP	1
gamete generation (GO:0007276)	28	1	0.03	29.31	3.47E-02	1.00E+00	BP	1
glucuronoxylan biosynthetic process (GO:0010417)	28	1	0.03	29.31	3.47E-02	1.00E+00	BP	1
glucuronoxylan metabolic process (GO:0010413)	28	1	0.03	29.31	3.47E-02	1.00E+00	BP	1
regulation of photoperiodism, flowering (GO:2000028)	29	1	0.04	28.3	3.59E-02	1.00E+00	BP	1
positive regulation of multicellular organismal process (GO:0051240)	33	1	0.04	24.87	4.06E-02	1.00E+00	BP	1
regulation of cell morphogenesis (GO:0022604)	34	1	0.04	24.14	4.17E-02	1.00E+00	BP	1
positive regulation of developmental process (GO:0051094)	37	1	0.05	22.18	4.52E-02	1.00E+00	BP	1
histone lysine methylation (GO:0034968)	40	1	0.05	20.52	4.87E-02	1.00E+00	BP	1
positive regulation of growth (GO:0045927)	41	1	0.05	20.01	4.99E-02	1.00E+00	BP	1
multicellular organism reproduction (GO:0032504)	45	1	0.05	18.24	5.45E-02	1.00E+00	BP	1
regulation of developmental growth (GO:0048638)	45	1	0.05	18.24	5.45E-02	1.00E+00	BP	1
multicellular organismal reproductive process (GO:0048609)	45	1	0.05	18.24	5.45E-02	1.00E+00	BP	1
regulation of jasmonic acid mediated signaling pathway (GO:2000022)	46	1	0.06	17.84	5.57E-02	1.00E+00	BP	1
response to gibberellin (GO:0009739)	47	1	0.06	17.46	5.68E-02	1.00E+00	BP	1
peptidyl-lysine methylation (GO:0018022)	54	1	0.07	15.2	6.48E-02	1.00E+00	BP	1
histone methylation (GO:0016571)	55	1	0.07	14.92	6.60E-02	1.00E+00	BP	1
mRNA transport (GO:0051028)	58	1	0.07	14.15	6.94E-02	1.00E+00	BP	1
negative regulation of response to stimulus (GO:0048585)	58	1	0.07	14.15	6.94E-02	1.00E+00	BP	1
xylan biosynthetic process (GO:0045492)	59	1	0.07	13.91	7.05E-02	1.00E+00	BP	1
plant-type secondary cell wall biogenesis (GO:0009834)	66	1	0.08	12.43	7.84E-02	1.00E+00	BP	1
meiosis I cell cycle process (GO:0061982)	71	1	0.09	11.56	8.40E-02	1.00E+00	BP	1
histone modification (GO:0016570)	143	2	0.17	11.48	1.36E-02	1.00E+00	BP	1
regulation of anatomical structure morphogenesis (GO:0022603)	73	1	0.09	11.24	8.63E-02	1.00E+00	BP	1
establishment of RNA localization (GO:0051236)	74	1	0.09	11.09	8.74E-02	1.00E+00	BP	1

GO Term	Genome	Count	Expected	Fold Enrichment	P-value	FDR	Term	Cluster
RNA transport (GO:0050658)	74	1	0.09	11.09	8.74E-02	1.00E+00	BP	1
nucleic acid transport (GO:0050657)	74	1	0.09	11.09	8.74E-02	1.00E+00	BP	1
regulation of multicellular organismal process (GO:0051239)	150	2	0.18	10.94	1.48E-02	1.00E+00	BP	1
RNA localization (GO:0006403)	76	1	0.09	10.8	8.96E-02	1.00E+00	BP	1
beta-glucan biosynthetic process (GO:0051274)	80	1	0.1	10.26	9.40E-02	1.00E+00	BP	1
spindle organization (GO:0007051)	80	1	0.1	10.26	9.40E-02	1.00E+00	BP	1
regulation of developmental process (GO:0050793)	245	3	0.3	10.05	3.45E-03	1.00E+00	BP	1
protein methylation (GO:0006479)	85	1	0.1	9.65	9.95E-02	1.00E+00	BP	1
response to wounding (GO:0009611)	85	1	0.1	9.65	9.95E-02	1.00E+00	BP	1
cell surface receptor signaling pathway (GO:0007166)	85	1	0.1	9.65	9.95E-02	1.00E+00	BP	1
protein alkylation (GO:0008213)	85	1	0.1	9.65	9.95E-02	1.00E+00	BP	1
meiotic nuclear division (GO:0140013)	86	1	0.1	9.54	1.01E-01	1.00E+00	BP	1
regulation of growth (GO:0040008)	92	1	0.11	8.92	1.07E-01	1.00E+00	BP	1
inorganic anion transport (GO:0015698)	97	1	0.12	8.46	1.13E-01	1.00E+00	BP	1
regulation of multicellular organismal development (GO:2000026)	106	1	0.13	7.74	1.22E-01	1.00E+00	BP	1
regulation of post-embryonic development (GO:0048580)	106	1	0.13	7.74	1.22E-01	1.00E+00	BP	1
protein deubiquitination (GO:0016579)	108	1	0.13	7.6	1.24E-01	1.00E+00	BP	1
beta-glucan metabolic process (GO:0051273)	111	1	0.14	7.39	1.28E-01	1.00E+00	BP	1
meiotic cell cycle process (GO:1903046)	111	1	0.14	7.39	1.28E-01	1.00E+00	BP	1
regulation of defense response (GO:0031347)	113	1	0.14	7.26	1.30E-01	1.00E+00	BP	1
regulation of reproductive process (GO:2000241)	113	1	0.14	7.26	1.30E-01	1.00E+00	BP	1
nuclear division (GO:0000280)	121	1	0.15	6.78	1.38E-01	1.00E+00	BP	1
chromosome segregation (GO:0007059)	126	1	0.15	6.51	1.44E-01	1.00E+00	BP	1
plant-type cell wall biogenesis (GO:0009832)	128	1	0.16	6.41	1.46E-01	1.00E+00	BP	1
xylan metabolic process (GO:0045491)	131	1	0.16	6.26	1.49E-01	1.00E+00	BP	1
polysaccharide biosynthetic process (GO:0000271)	281	2	0.34	5.84	4.66E-02	1.00E+00	BP	1
cell wall polysaccharide biosynthetic process (GO:0070592)	146	1	0.18	5.62	1.64E-01	1.00E+00	BP	1
organelle fission (GO:0048285)	150	1	0.18	5.47	1.68E-01	1.00E+00	BP	1
RNA modification (GO:0009451)	453	3	0.55	5.43	1.81E-02	1.00E+00	BP	1
regulation of response to stimulus (GO:0048583)	310	2	0.38	5.29	5.55E-02	1.00E+00	BP	1
regulation of response to stress (GO:0080134)	158	1	0.19	5.19	1.76E-01	1.00E+00	BP	1
cellular response to lipid (GO:0071396)	162	1	0.2	5.07	1.80E-01	1.00E+00	BP	1
regulation of signal transduction (GO:0009966)	162	1	0.2	5.07	1.80E-01	1.00E+00	BP	1
regulation of signaling (GO:0023051)	163	1	0.2	5.03	1.81E-01	1.00E+00	BP	1
regulation of cell communication (GO:0010646)	166	1	0.2	4.94	1.84E-01	1.00E+00	BP	1
nucleobase-containing compound transport (GO:0015931)	174	1	0.21	4.72	1.92E-01	1.00E+00	BP	1
nucleic acid phosphodiester bond hydrolysis (GO:0090305)	176	1	0.21	4.66	1.94E-01	1.00E+00	BP	1
polysaccharide catabolic process (GO:0000272)	176	1	0.21	4.66	1.94E-01	1.00E+00	BP	1
microtubule cytoskeleton organization (GO:0000226)	181	1	0.22	4.53	1.99E-01	1.00E+00	BP	1
plant-type cell wall organization or biogenesis (GO:0071669)	181	1	0.22	4.53	1.99E-01	1.00E+00	BP	1
protein glycosylation (GO:0006486)	185	1	0.23	4.44	2.03E-01	1.00E+00	BP	1
macromolecule glycosylation (GO:0043413)	185	1	0.23	4.44	2.03E-01	1.00E+00	BP	1
glycoprotein biosynthetic process (GO:0009101)	187	1	0.23	4.39	2.05E-01	1.00E+00	BP	1
polysaccharide metabolic process (GO:0005976)	562	3	0.68	4.38	3.14E-02	1.00E+00	BP	1
glycosylation (GO:0070085)	191	1	0.23	4.3	2.09E-01	1.00E+00	BP	1
meiotic cell cycle (GO:0051321)	193	1	0.24	4.25	2.11E-01	1.00E+00	BP	1
glucan biosynthetic process (GO:0009250)	195	1	0.24	4.21	2.13E-01	1.00E+00	BP	1
macromolecule methylation (GO:0043414)	198	1	0.24	4.14	2.16E-01	1.00E+00	BP	1
carbohydrate biosynthetic process (GO:0016051)	396	2	0.48	4.14	8.46E-02	1.00E+00	BP	1
response to temperature stimulus (GO:0009266)	202	1	0.25	4.06	2.20E-01	1.00E+00	BP	1
glycoprotein metabolic process (GO:0009100)	205	1	0.25	4	2.22E-01	1.00E+00	BP	1

GO Term	Genome	Count	Expected	Fold Enrichment	P-value	FDR	Term	Cluster
cell wall biogenesis (GO:0042546)	210	1	0.26	3.91	2.27E-01	1.00E+00	BP	1
protein modification by small protein removal (GO:0070646)	230	1	0.28	3.57	2.46E-01	1.00E+00	BP	1
cellular response to oxygen-containing compound (GO:1901701)	239	1	0.29	3.43	2.54E-01	1.00E+00	BP	1
peptidyl-lysine modification (GO:0018205)	240	1	0.29	3.42	2.55E-01	1.00E+00	BP	1
hemicellulose metabolic process (GO:0010410)	240	1	0.29	3.42	2.55E-01	1.00E+00	BP	1
cell wall polysaccharide metabolic process (GO:0010383)	250	1	0.3	3.28	2.64E-01	1.00E+00	BP	1
sexual reproduction (GO:0019953)	258	1	0.31	3.18	2.71E-01	1.00E+00	BP	1
response to lipid (GO:0033993)	271	1	0.33	3.03	2.83E-01	1.00E+00	BP	1
cell wall macromolecule metabolic process (GO:0044036)	274	1	0.33	2.99	2.85E-01	1.00E+00	BP	1
cell wall organization or biogenesis (GO:0071554)	586	2	0.71	2.8	1.60E-01	1.00E+00	BP	1
microtubule-based process (GO:0007017)	293	1	0.36	2.8	3.02E-01	1.00E+00	BP	1
glucan metabolic process (GO:0044042)	294	1	0.36	2.79	3.03E-01	1.00E+00	BP	1
modification-dependent protein catabolic process (GO:0019941)	614	2	0.75	2.67	1.73E-01	1.00E+00	BP	1
macromolecule modification (GO:0043412)	3738	12	4.56	2.63	1.44E-03	1.00E+00	BP	1
modification-dependent macromolecule catabolic process (GO:0043632)	636	2	0.78	2.58	1.82E-01	1.00E+00	BP	1
cytoskeleton organization (GO:0007010)	340	1	0.41	2.41	3.41E-01	1.00E+00	BP	1
cell wall organization (GO:0071555)	343	1	0.42	2.39	3.43E-01	1.00E+00	BP	1
carbohydrate catabolic process (GO:0016052)	346	1	0.42	2.37	3.46E-01	1.00E+00	BP	1
protein modification process (GO:0036211)	3194	9	3.89	2.31	1.40E-02	1.00E+00	BP	1
macromolecule catabolic process (GO:0009057)	1069	3	1.3	2.3	1.42E-01	1.00E+00	BP	1
external encapsulating structure organization (GO:0045229)	357	1	0.44	2.3	3.55E-01	1.00E+00	BP	1
proteolysis involved in protein catabolic process (GO:0051603)	726	2	0.88	2.26	2.22E-01	1.00E+00	BP	1
protein catabolic process (GO:0030163)	738	2	0.9	2.22	2.27E-01	1.00E+00	BP	1
response to hormone (GO:0009725)	756	2	0.92	2.17	2.35E-01	1.00E+00	BP	1
response to endogenous stimulus (GO:0009719)	762	2	0.93	2.15	2.38E-01	1.00E+00	BP	1
protein phosphorylation (GO:0006468)	1543	4	1.88	2.13	1.18E-01	1.00E+00	BP	1
DNA-templated transcription (GO:0006351)	824	2	1	1.99	2.66E-01	1.00E+00	BP	1
chromatin organization (GO:0006325)	417	1	0.51	1.97	4.01E-01	1.00E+00	BP	1
signal transduction (GO:0007165)	1258	3	1.53	1.96	1.98E-01	1.00E+00	BP	1
RNA biosynthetic process (GO:0032774)	840	2	1.02	1.95	2.73E-01	1.00E+00	BP	1
protein modification by small protein conjugation or removal (GO:0070647)	847	2	1.03	1.94	2.77E-01	1.00E+00	BP	1
cell cycle process (GO:0022402)	425	1	0.52	1.93	4.06E-01	1.00E+00	BP	1
signaling (GO:0023052)	1277	3	1.56	1.93	2.04E-01	1.00E+00	BP	1
regulation of biological quality (GO:0065008)	433	1	0.53	1.9	4.12E-01	1.00E+00	BP	1
cell communication (GO:0007154)	1326	3	1.62	1.86	2.19E-01	1.00E+00	BP	1
carbohydrate metabolic process (GO:0005975)	1347	3	1.64	1.83	2.26E-01	1.00E+00	BP	1
RNA metabolic process (GO:0016070)	2384	5	2.91	1.72	2.13E-01	1.00E+00	BP	1
organonitrogen compound catabolic process (GO:1901565)	955	2	1.16	1.72	3.25E-01	1.00E+00	BP	1
protein-DNA complex organization (GO:0071824)	481	1	0.59	1.71	4.46E-01	1.00E+00	BP	1
regulation of biological process (GO:0050789)	5297	11	6.46	1.7	8.55E-02	1.00E+00	BP	1
macromolecule biosynthetic process (GO:0009059)	2436	5	2.97	1.68	2.21E-01	1.00E+00	BP	1
response to oxygen-containing compound (GO:1901700)	492	1	0.6	1.67	4.53E-01	1.00E+00	BP	1
hormone-mediated signaling pathway (GO:0009755)	497	1	0.61	1.65	4.57E-01	1.00E+00	BP	1
response to organic substance (GO:0010033)	1000	2	1.22	1.64	3.45E-01	1.00E+00	BP	1
reproductive process (GO:0022414)	503	1	0.61	1.63	4.61E-01	1.00E+00	BP	1
cellular response to hormone stimulus (GO:0032870)	504	1	0.61	1.63	4.62E-01	1.00E+00	BP	1
protein-containing complex organization (GO:0043933)	1011	2	1.23	1.62	3.50E-01	1.00E+00	BP	1
cellular response to endogenous stimulus (GO:0071495)	510	1	0.62	1.61	4.65E-01	1.00E+00	BP	1
biological regulation (GO:0065007)	5626	11	6.86	1.6	9.72E-02	1.00E+00	BP	1
carbohydrate derivative biosynthetic process (GO:1901137)	512	1	0.62	1.6	4.67E-01	1.00E+00	BP	1
regulation of cellular process (GO:0050794)	4625	9	5.64	1.6	1.72E-01	1.00E+00	BP	1

GO Term	Genome	Count	Expected	Fold Enrichment	P-value	FDR	Term	Cluster
macromolecule metabolic process (GO:0043170)	8871	17	10.81	1.57	3.82E-02	1.00E+00	BP	1
regulation of RNA biosynthetic process (GO:2001141)	2705	5	3.3	1.52	3.81E-01	1.00E+00	BP	1
regulation of DNA-templated transcription (GO:0006355)	2705	5	3.3	1.52	3.81E-01	1.00E+00	BP	1
methylation (GO:0032259)	541	1	0.66	1.52	4.86E-01	1.00E+00	BP	1
phosphorylation (GO:0016310)	2164	4	2.64	1.52	3.36E-01	1.00E+00	BP	1
peptidyl-amino acid modification (GO:0018193)	544	1	0.66	1.51	4.87E-01	1.00E+00	BP	1
reproduction (GO:0000003)	562	1	0.68	1.46	4.99E-01	1.00E+00	BP	1
nucleic acid metabolic process (GO:0090304)	3395	6	4.14	1.45	3.03E-01	1.00E+00	BP	1
regulation of RNA metabolic process (GO:0051252)	2843	5	3.46	1.44	3.94E-01	1.00E+00	BP	1
protein ubiquitination (GO:0016567)	573	1	0.7	1.43	5.05E-01	1.00E+00	BP	1
cellular response to organic substance (GO:0071310)	578	1	0.7	1.42	5.09E-01	1.00E+00	BP	1
regulation of nucleobase-containing compound metabolic process (GO:0019219)	2913	5	3.55	1.41	4.01E-01	1.00E+00	BP	1
protein metabolic process (GO:0019538)	5254	9	6.4	1.41	2.85E-01	1.00E+00	BP	1
cell cycle (GO:0007049)	586	1	0.71	1.4	5.13E-01	1.00E+00	BP	1
regulation of macromolecule biosynthetic process (GO:0010556)	2942	5	3.59	1.39	4.05E-01	1.00E+00	BP	1
regulation of cellular biosynthetic process (GO:0031326)	2959	5	3.61	1.39	4.07E-01	1.00E+00	BP	1
regulation of biosynthetic process (GO:0009889)	2969	5	3.62	1.38	4.08E-01	1.00E+00	BP	1
ubiquitin-dependent protein catabolic process (GO:0006511)	601	1	0.73	1.37	5.22E-01	1.00E+00	BP	1
response to abiotic stimulus (GO:0009628)	611	1	0.74	1.34	5.28E-01	1.00E+00	BP	1
protein modification by small protein conjugation (GO:0032446)	618	1	0.75	1.33	5.32E-01	1.00E+00	BP	1
organic substance catabolic process (GO:1901575)	1863	3	2.27	1.32	4.96E-01	1.00E+00	BP	1
nucleobase-containing compound biosynthetic process (GO:0034654)	1266	2	1.54	1.3	6.68E-01	1.00E+00	BP	1
nitrogen compound metabolic process (GO:0006807)	9609	15	11.71	1.28	3.12E-01	1.00E+00	BP	1
nucleobase-containing compound metabolic process (GO:0006139)	3868	6	4.71	1.27	4.68E-01	1.00E+00	BP	1
catabolic process (GO:0009056)	1947	3	2.37	1.26	5.13E-01	1.00E+00	BP	1
regulation of cellular metabolic process (GO:0031323)	3270	5	3.98	1.25	5.96E-01	1.00E+00	BP	1
regulation of gene expression (GO:0010468)	3303	5	4.03	1.24	5.98E-01	1.00E+00	BP	1
regulation of nitrogen compound metabolic process (GO:0051171)	3361	5	4.1	1.22	6.02E-01	1.00E+00	BP	1
primary metabolic process (GO:0044238)	11484	17	13.99	1.21	3.43E-01	1.00E+00	BP	1
regulation of primary metabolic process (GO:0080090)	3400	5	4.14	1.21	6.05E-01	1.00E+00	BP	1
multicellular organismal process (GO:0032501)	686	1	0.84	1.2	5.70E-01	1.00E+00	BP	1
cellular component organization or biogenesis (GO:0071840)	3437	5	4.19	1.19	6.08E-01	1.00E+00	BP	1
heterocycle metabolic process (GO:0046483)	4164	6	5.07	1.18	6.37E-01	1.00E+00	BP	1
vesicle-mediated transport (GO:0016192)	699	1	0.85	1.17	5.77E-01	1.00E+00	BP	1
cellular nitrogen compound metabolic process (GO:0034641)	4908	7	5.98	1.17	6.60E-01	1.00E+00	BP	1
organonitrogen compound metabolic process (GO:1901564)	6443	9	7.85	1.15	6.95E-01	1.00E+00	BP	1
cellular aromatic compound metabolic process (GO:0006725)	4327	6	5.27	1.14	6.47E-01	1.00E+00	BP	1
organic substance metabolic process (GO:0071704)	12450	17	15.17	1.12	6.41E-01	1.00E+00	BP	1
cellular component organization (GO:0016043)	2935	4	3.58	1.12	7.80E-01	1.00E+00	BP	1
response to stimulus (GO:0050896)	4415	6	5.38	1.12	8.17E-01	1.00E+00	BP	1
regulation of macromolecule metabolic process (GO:0060255)	3684	5	4.49	1.11	8.02E-01	1.00E+00	BP	1
organic cyclic compound metabolic process (GO:1901360)	4430	6	5.4	1.11	8.18E-01	1.00E+00	BP	1
heterocycle biosynthetic process (GO:0018130)	1487	2	1.81	1.1	7.03E-01	1.00E+00	BP	1
phosphate-containing compound metabolic process (GO:0006796)	2984	4	3.64	1.1	7.83E-01	1.00E+00	BP	1
positive regulation of biological process (GO:0048518)	749	1	0.91	1.1	6.02E-01	1.00E+00	BP	1
regulation of metabolic process (GO:0019222)	3766	5	4.59	1.09	8.05E-01	1.00E+00	BP	1
response to chemical (GO:0042221)	1513	2	1.84	1.08	7.07E-01	1.00E+00	BP	1
Unclassified (UNCLASSIFIED)	17419	23	21.23	1.08	6.64E-01	1.00E+00	BP	1
phosphorus metabolic process (GO:0006793)	3030	4	3.69	1.08	7.85E-01	1.00E+00	BP	1
carbohydrate derivative metabolic process (GO:1901135)	763	1	0.93	1.08	6.09E-01	1.00E+00	BP	1
proteolysis (GO:0006508)	1544	2	1.88	1.06	7.13E-01	1.00E+00	BP	1

GO Term	Genome	Count	Expected	Fold Enrichment	P-value	FDR	Term	Cluster
aromatic compound biosynthetic process (GO:0019438)	1547	2	1.89	1.06	7.13E-01	1.00E+00	BP	1
transport (GO:0006810)	3186	4	3.88	1.03	7.94E-01	1.00E+00	BP	1
metabolic process (GO:0008152)	13613	17	16.59	1.02	8.80E-01	1.00E+00	BP	1
negative regulation of biological process (GO:0048519)	801	1	0.98	1.02	6.27E-01	1.00E+00	BP	1
establishment of localization (GO:0051234)	3225	4	3.93	1.02	1.00E+00	1.00E+00	BP	1
cellular nitrogen compound biosynthetic process (GO:0044271)	2423	3	2.95	1.02	1.00E+00	1.00E+00	BP	1
organic substance biosynthetic process (GO:1901576)	4099	5	5	1	1.00E+00	1.00E+00	BP	1
localization (GO:0051179)	3340	4	4.07	0.98	1.00E+00	1.00E+00	BP	1
organic cyclic compound biosynthetic process (GO:1901362)	1677	2	2.04	0.98	1.00E+00	1.00E+00	BP	1
organelle organization (GO:0006996)	1700	2	2.07	0.97	1.00E+00	1.00E+00	BP	1
biosynthetic process (GO:0009058)	4259	5	5.19	0.96	1.00E+00	1.00E+00	BP	1
cellular process (GO:0009987)	15372	18	18.73	0.96	8.83E-01	1.00E+00	BP	1
cellular metabolic process (GO:0044237)	9471	11	11.54	0.95	1.00E+00	1.00E+00	BP	1
transmembrane transport (GO:0055085)	1724	2	2.1	0.95	1.00E+00	1.00E+00	BP	1
biological_process (GO:0008150)	21970	25	26.77	0.93	6.64E-01	1.00E+00	BP	1
cellular response to stimulus (GO:0051716)	2710	3	3.3	0.91	1.00E+00	1.00E+00	BP	1
cellular response to chemical stimulus (GO:0070887)	934	1	1.14	0.88	1.00E+00	1.00E+00	BP	1
translation (GO:0006412)	947	1	1.15	0.87	1.00E+00	1.00E+00	BP	1
protein transport (GO:0015031)	950	1	1.16	0.86	1.00E+00	1.00E+00	BP	1
establishment of protein localization (GO:0045184)	962	1	1.17	0.85	1.00E+00	1.00E+00	BP	1
peptide biosynthetic process (GO:0043043)	965	1	1.18	0.85	1.00E+00	1.00E+00	BP	1
organonitrogen compound biosynthetic process (GO:1901566)	2013	2	2.45	0.82	1.00E+00	1.00E+00	BP	1
peptide metabolic process (GO:0006518)	1013	1	1.23	0.81	1.00E+00	1.00E+00	BP	1
protein localization (GO:0008104)	1049	1	1.28	0.78	1.00E+00	1.00E+00	BP	1
cellular macromolecule localization (GO:0070727)	1050	1	1.28	0.78	1.00E+00	1.00E+00	BP	1
amide biosynthetic process (GO:0043604)	1059	1	1.29	0.77	1.00E+00	1.00E+00	BP	1
cellular biosynthetic process (GO:0044249)	3461	3	4.22	0.71	7.97E-01	1.00E+00	BP	1
amide metabolic process (GO:0043603)	1168	1	1.42	0.7	1.00E+00	1.00E+00	BP	1
macromolecule localization (GO:0033036)	1264	1	1.54	0.65	1.00E+00	1.00E+00	BP	1
cellular localization (GO:0051641)	1297	1	1.58	0.63	1.00E+00	1.00E+00	BP	1
nitrogen compound transport (GO:0071705)	1381	1	1.68	0.59	1.00E+00	1.00E+00	BP	1
cellular component biogenesis (GO:0044085)	1401	1	1.71	0.59	1.00E+00	1.00E+00	BP	1
organic substance transport (GO:0071702)	1759	1	2.14	0.47	7.25E-01	1.00E+00	BP	1
gene expression (GO:0010467)	2102	1	2.56	0.39	5.19E-01	1.00E+00	BP	1
response to stress (GO:0006950)	2296	1	2.8	0.36	5.28E-01	1.00E+00	BP	1
endo-1,4-beta-xylanase activity (GO:0031176)	11	1	0.01	74.6	1.45E-02	1.00E+00	MF	1
protein tag (GO:0031386)	13	1	0.02	63.12	1.69E-02	1.00E+00	MF	1
1,3-beta-D-glucan synthase activity (GO:0003843)	18	1	0.02	45.59	2.29E-02	1.00E+00	MF	1
xylanase activity (GO:0097599)	21	1	0.03	39.08	2.64E-02	1.00E+00	MF	1
protein serine/threonine kinase activator activity (GO:0043539)	27	1	0.03	30.39	3.35E-02	1.00E+00	MF	1
mRNA 3'-UTR binding (GO:0003730)	28	1	0.03	29.31	3.47E-02	1.00E+00	MF	1
inorganic phosphate transmembrane transporter activity (GO:0005315)	34	1	0.04	24.14	4.17E-02	1.00E+00	MF	1
glucuronosyltransferase activity (GO:0015020)	38	1	0.05	21.59	4.64E-02	1.00E+00	MF	1
ubiquitin protein ligase binding (GO:0031625)	40	1	0.05	20.52	4.87E-02	1.00E+00	MF	1
ubiquitin-like protein ligase binding (GO:0044389)	42	1	0.05	19.54	5.10E-02	1.00E+00	MF	1
protein kinase activator activity (GO:0030295)	45	1	0.05	18.24	5.45E-02	1.00E+00	MF	1
kinase activator activity (GO:0019209)	45	1	0.05	18.24	5.45E-02	1.00E+00	MF	1
symporter activity (GO:0015293)	121	2	0.15	13.56	9.91E-03	1.00E+00	MF	1
cysteine-type deubiquitinase activity (GO:0004843)	87	1	0.11	9.43	1.02E-01	1.00E+00	MF	1
deubiquitinase activity (GO:0101005)	102	1	0.12	8.05	1.18E-01	1.00E+00	MF	1
cysteine-type endopeptidase activity (GO:0004197)	106	1	0.13	7.74	1.22E-01	1.00E+00	MF	1

GO Term	Genome	Count	Expected	Fold Enrichment	P-value	FDR	Term	Cluster
protein-macromolecule adaptor activity (GO:0030674)	116	1	0.14	7.07	1.33E-01	1.00E+00	MF	1
molecular adaptor activity (GO:0060090)	124	1	0.15	6.62	1.41E-01	1.00E+00	MF	1
protein kinase regulator activity (GO:0019887)	127	1	0.15	6.46	1.45E-01	1.00E+00	MF	1
kinase regulator activity (GO:0019207)	137	1	0.17	5.99	1.55E-01	1.00E+00	MF	1
secondary active transmembrane transporter activity (GO:0015291)	347	2	0.42	4.73	6.75E-02	1.00E+00	MF	1
enzyme activator activity (GO:0008047)	184	1	0.22	4.46	2.02E-01	1.00E+00	MF	1
molecular function activator activity (GO:0140677)	203	1	0.25	4.04	2.20E-01	1.00E+00	MF	1
UDP-glycosyltransferase activity (GO:0008194)	428	2	0.52	3.83	9.65E-02	1.00E+00	MF	1
zinc ion binding (GO:0008270)	651	3	0.79	3.78	4.53E-02	1.00E+00	MF	1
ubiquitin-like protein peptidase activity (GO:0019783)	220	1	0.27	3.73	2.37E-01	1.00E+00	MF	1
flavin adenine dinucleotide binding (GO:0050660)	230	1	0.28	3.57	2.46E-01	1.00E+00	MF	1
oxidoreductase activity, acting on the CH-OH group of donors, NAD or NADP as acceptor (GO:0016616)	255	1	0.31	3.22	2.69E-01	1.00E+00	MF	1
enzyme binding (GO:0019899)	271	1	0.33	3.03	2.83E-01	1.00E+00	MF	1
oxidoreductase activity, acting on CH-OH group of donors (GO:0016614)	290	1	0.35	2.83	2.99E-01	1.00E+00	MF	1
UDP-glucosyltransferase activity (GO:0035251)	298	1	0.36	2.75	3.06E-01	1.00E+00	MF	1
protein serine/threonine kinase activity (GO:0004674)	923	3	1.12	2.67	1.03E-01	1.00E+00	MF	1
hexosyltransferase activity (GO:0016758)	637	2	0.78	2.58	1.83E-01	1.00E+00	MF	1
active transmembrane transporter activity (GO:0022804)	645	2	0.79	2.54	1.86E-01	1.00E+00	MF	1
glycosyltransferase activity (GO:0016757)	972	3	1.18	2.53	1.15E-01	1.00E+00	MF	1
mRNA binding (GO:0003729)	651	2	0.79	2.52	1.89E-01	1.00E+00	MF	1
glucosyltransferase activity (GO:0046527)	340	1	0.41	2.41	3.41E-01	1.00E+00	MF	1
ubiquitin protein ligase activity (GO:0061630)	354	1	0.43	2.32	3.52E-01	1.00E+00	MF	1
calcium ion binding (GO:0005509)	355	1	0.43	2.31	3.53E-01	1.00E+00	MF	1
ubiquitin-like protein ligase activity (GO:0061659)	360	1	0.44	2.28	3.57E-01	1.00E+00	MF	1
cysteine-type peptidase activity (GO:0008234)	385	1	0.47	2.13	3.77E-01	1.00E+00	MF	1
DNA-binding transcription factor activity (GO:0003700)	1586	4	1.93	2.07	1.27E-01	1.00E+00	MF	1
protein kinase activity (GO:0004672)	1604	4	1.95	2.05	1.31E-01	1.00E+00	MF	1
RNA binding (GO:0003723)	2087	5	2.54	1.97	1.09E-01	1.00E+00	MF	1
transition metal ion binding (GO:0046914)	1290	3	1.57	1.91	2.08E-01	1.00E+00	MF	1
transcription regulator activity (GO:0140110)	1753	4	2.14	1.87	1.65E-01	1.00E+00	MF	1
phosphotransferase activity, alcohol group as acceptor (GO:0016773)	1823	4	2.22	1.8	2.84E-01	1.00E+00	MF	1
hydrolase activity, hydrolyzing O-glycosyl compounds (GO:0004553)	462	1	0.56	1.78	4.33E-01	1.00E+00	MF	1
endopeptidase activity (GO:0004175)	467	1	0.57	1.76	4.36E-01	1.00E+00	MF	1
hydrolase activity, acting on glycosyl bonds (GO:0016798)	525	1	0.64	1.56	4.75E-01	1.00E+00	MF	1
ligase activity (GO:0016874)	529	1	0.64	1.55	4.78E-01	1.00E+00	MF	1
kinase activity (GO:0016301)	2226	4	2.71	1.47	3.48E-01	1.00E+00	MF	1
ubiquitin-protein transferase activity (GO:0004842)	558	1	0.68	1.47	4.96E-01	1.00E+00	MF	1
inorganic molecular entity transmembrane transporter activity (GO:0015318)	562	1	0.68	1.46	4.99E-01	1.00E+00	MF	1
nucleic acid binding (GO:0003676)	5685	10	6.93	1.44	2.16E-01	1.00E+00	MF	1
ubiquitin-like protein transferase activity (GO:0019787)	578	1	0.7	1.42	5.09E-01	1.00E+00	MF	1
aminoacyltransferase activity (GO:0016755)	583	1	0.71	1.41	5.12E-01	1.00E+00	MF	1
transcription regulatory region nucleic acid binding (GO:0001067)	589	1	0.72	1.39	5.15E-01	1.00E+00	MF	1
transcription cis-regulatory region binding (GO:0000976)	589	1	0.72	1.39	5.15E-01	1.00E+00	MF	1
structural constituent of ribosome (GO:0003735)	593	1	0.72	1.38	5.18E-01	1.00E+00	MF	1
enzyme regulator activity (GO:0030234)	604	1	0.74	1.36	5.24E-01	1.00E+00	MF	1
sequence-specific double-stranded DNA binding (GO:1990837)	608	1	0.74	1.35	5.26E-01	1.00E+00	MF	1
transferase activity, transferring phosphorus-containing groups (GO:0016772)	2537	4	3.09	1.29	5.50E-01	1.00E+00	MF	1
DNA binding (GO:0003677)	3182	5	3.88	1.29	5.90E-01	1.00E+00	MF	1
molecular function regulator activity (GO:0098772)	652	1	0.79	1.26	5.52E-01	1.00E+00	MF	1
catalytic activity, acting on a protein (GO:0140096)	3926	6	4.78	1.25	4.74E-01	1.00E+00	MF	1
heterocyclic compound binding (GO:1901363)	10349	15	12.61	1.19	4.16E-01	1.00E+00	MF	1

GO Term	Genome	Count	Expected	Fold Enrichment	P-value	FDR	Term	Cluster
organic cyclic compound binding (GO:0097159)	10373	15	12.64	1.19	4.17E-01	1.00E+00	MF	1
transferase activity (GO:0016740)	5764	8	7.02	1.14	6.82E-01	1.00E+00	MF	1
double-stranded DNA binding (GO:0003690)	723	1	0.88	1.13	5.89E-01	1.00E+00	MF	1
binding (GO:0005488)	14290	19	17.41	1.09	6.54E-01	1.00E+00	MF	1
metal ion binding (GO:0046872)	3774	5	4.6	1.09	8.05E-01	1.00E+00	MF	1
cation binding (GO:0043169)	3802	5	4.63	1.08	8.06E-01	1.00E+00	MF	1
Unclassified (UNCLASSIFIED)	16329	21	19.9	1.06	7.71E-01	1.00E+00	MF	1
ion binding (GO:0043167)	7841	10	9.56	1.05	8.57E-01	1.00E+00	MF	1
structural molecule activity (GO:0005198)	803	1	0.98	1.02	1.00E+00	1.00E+00	MF	1
transmembrane transporter activity (GO:0022857)	1612	2	1.96	1.02	1.00E+00	1.00E+00	MF	1
transporter activity (GO:0005215)	1678	2	2.04	0.98	1.00E+00	1.00E+00	MF	1
ATP binding (GO:0005524)	3362	4	4.1	0.98	1.00E+00	1.00E+00	MF	1
molecular_function (GO:0003674)	23060	27	28.1	0.96	7.71E-01	1.00E+00	MF	1
nucleotide binding (GO:0000166)	4340	5	5.29	0.95	1.00E+00	1.00E+00	MF	1
nucleoside phosphate binding (GO:1901265)	4340	5	5.29	0.95	1.00E+00	1.00E+00	MF	1
adenyl ribonucleotide binding (GO:0032559)	3497	4	4.26	0.94	1.00E+00	1.00E+00	MF	1
anion binding (GO:0043168)	4484	5	5.46	0.92	1.00E+00	1.00E+00	MF	1
small molecule binding (GO:0036094)	4599	5	5.6	0.89	1.00E+00	1.00E+00	MF	1
adenyl nucleotide binding (GO:0030554)	3702	4	4.51	0.89	1.00E+00	1.00E+00	MF	1
purine ribonucleoside triphosphate binding (GO:0035639)	3719	4	4.53	0.88	1.00E+00	1.00E+00	MF	1
purine ribonucleotide binding (GO:0032555)	3854	4	4.7	0.85	1.00E+00	1.00E+00	MF	1
ribonucleotide binding (GO:0032553)	3896	4	4.75	0.84	1.00E+00	1.00E+00	MF	1
carbohydrate derivative binding (GO:0097367)	3928	4	4.79	0.84	1.00E+00	1.00E+00	MF	1
peptidase activity (GO:0008233)	1007	1	1.23	0.81	1.00E+00	1.00E+00	MF	1
purine nucleotide binding (GO:0017076)	4059	4	4.95	0.81	8.15E-01	1.00E+00	MF	1
oxidoreductase activity (GO:0016491)	2048	2	2.5	0.8	1.00E+00	1.00E+00	MF	1
catalytic activity (GO:0003824)	13387	12	16.31	0.74	2.23E-01	1.00E+00	MF	1
acyltransferase activity (GO:0016746)	1155	1	1.41	0.71	1.00E+00	1.00E+00	MF	1
sequence-specific DNA binding (GO:0043565)	1176	1	1.43	0.7	1.00E+00	1.00E+00	MF	1
hydrolase activity (GO:0016787)	4566	2	5.56	0.36	1.70E-01	1.00E+00	MF	1
protein binding (GO:0005515)	2323	1	2.83	0.35	5.30E-01	1.00E+00	MF	1
nuclear pore transmembrane ring (GO:0070762)	2	1	0	> 100	3.65E-03	1.00E+00	CC	1
1,3-beta-D-glucan synthase complex (GO:0000148)	18	1	0.02	45.59	2.29E-02	1.00E+00	CC	1
nuclear membrane (GO:0031965)	22	1	0.03	37.3	2.76E-02	1.00E+00	CC	1
plasma membrane protein complex (GO:0098797)	45	1	0.05	18.24	5.45E-02	1.00E+00	CC	1
nuclear pore (GO:0005643)	65	1	0.08	12.62	7.73E-02	1.00E+00	CC	1
nuclear envelope (GO:0005635)	103	1	0.13	7.97	1.19E-01	1.00E+00	CC	1
ribosome (GO:0005840)	694	2	0.85	2.36	2.08E-01	1.00E+00	CC	1
Golgi apparatus (GO:0005794)	959	2	1.17	1.71	3.27E-01	1.00E+00	CC	1
nucleus (GO:0005634)	6118	11	7.46	1.48	1.62E-01	1.00E+00	CC	1
membrane (GO:0016020)	8074	12	9.84	1.22	4.73E-01	1.00E+00	CC	1
cytosol (GO:0005829)	1383	2	1.69	1.19	6.86E-01	1.00E+00	CC	1
endomembrane system (GO:0012505)	2115	3	2.58	1.16	7.43E-01	1.00E+00	CC	1
plasma membrane (GO:0005886)	1449	2	1.77	1.13	6.97E-01	1.00E+00	CC	1
membrane protein complex (GO:0098796)	763	1	0.93	1.08	6.09E-01	1.00E+00	CC	1
transferase complex (GO:1990234)	772	1	0.94	1.06	6.14E-01	1.00E+00	CC	1
intracellular membrane-bounded organelle (GO:0043231)	10826	14	13.19	1.06	7.49E-01	1.00E+00	CC	1
membrane-bounded organelle (GO:0043227)	10848	14	13.22	1.06	8.72E-01	1.00E+00	CC	1
intracellular organelle (GO:0043229)	11979	15	14.6	1.03	8.76E-01	1.00E+00	CC	1
organelle (GO:0043226)	11985	15	14.61	1.03	8.76E-01	1.00E+00	CC	1
Unclassified (UNCLASSIFIED)	19561	24	23.84	1.01	1.00E+00	1.00E+00	CC	1

GO Term	Genome	Count	Expected	Fold Enrichment	P-value	FDR	Term	Cluster
cellular anatomical entity (GO:0110165)	19592	24	23.88	1.01	1.00E+00	1.00E+00	CC	1
cellular_component (GO:0005575)	19828	24	24.16	0.99	1.00E+00	1.00E+00	CC	1
cell periphery (GO:0071944)	1669	2	2.03	0.98	1.00E+00	1.00E+00	CC	1
envelope (GO:0031975)	844	1	1.03	0.97	1.00E+00	1.00E+00	CC	1
organelle envelope (GO:0031967)	844	1	1.03	0.97	1.00E+00	1.00E+00	CC	1
intracellular anatomical structure (GO:0005622)	14114	16	17.2	0.93	7.65E-01	1.00E+00	CC	1
endoplasmic reticulum (GO:0005783)	954	1	1.16	0.86	1.00E+00	1.00E+00	CC	1
nuclear protein-containing complex (GO:0140513)	991	1	1.21	0.83	1.00E+00	1.00E+00	CC	1
cytoplasm (GO:0005737)	8535	8	10.4	0.77	4.85E-01	1.00E+00	CC	1
ribonucleoprotein complex (GO:1990904)	1113	1	1.36	0.74	1.00E+00	1.00E+00	CC	1
mitochondrion (GO:0005739)	1131	1	1.38	0.73	1.00E+00	1.00E+00	CC	1
intracellular non-membrane-bounded organelle (GO:0043232)	2611	2	3.18	0.63	7.70E-01	1.00E+00	CC	1
non-membrane-bounded organelle (GO:0043228)	2611	2	3.18	0.63	7.70E-01	1.00E+00	CC	1
protein-containing complex (GO:0032991)	4069	3	4.96	0.61	4.79E-01	1.00E+00	CC	1
catalytic complex (GO:1902494)	1505	1	1.83	0.55	1.00E+00	1.00E+00	CC	1
organelle membrane (GO:0031090)	1851	1	2.26	0.44	7.28E-01	1.00E+00	CC	1
tRNA guanine ribose methylation (GO:0002938)	1	1	0	> 100	8.58E-03	1.00E+00	BP	2
cellular response to UV-A (GO:0071492)	2	1	0.01	> 100	1.28E-02	1.00E+00	BP	2
[2Fe-2S] cluster assembly (GO:0044571)	2	1	0.01	> 100	1.28E-02	1.00E+00	BP	2
response to UV-A (GO:0070141)	2	1	0.01	> 100	1.28E-02	1.00E+00	BP	2
regulation of ER to Golgi vesicle-mediated transport (GO:0060628)	2	1	0.01	> 100	1.28E-02	1.00E+00	BP	2
tRNA nucleoside ribose methylation (GO:0002128)	3	1	0.01	77.23	1.71E-02	1.00E+00	BP	2
histidyl-tRNA aminoacylation (GO:0006427)	3	1	0.01	77.23	1.71E-02	1.00E+00	BP	2
positive gravitropism (GO:0009958)	3	1	0.01	77.23	1.71E-02	1.00E+00	BP	2
cellular response to high light intensity (GO:0071486)	4	1	0.02	57.92	2.13E-02	1.00E+00	BP	2
cellular response to light intensity (GO:0071484)	4	1	0.02	57.92	2.13E-02	1.00E+00	BP	2
cytosolic ribosome assembly (GO:0042256)	4	1	0.02	57.92	2.13E-02	1.00E+00	BP	2
response to mechanical stimulus (GO:0009612)	5	1	0.02	46.34	2.55E-02	1.00E+00	BP	2
folic acid biosynthetic process (GO:0046656)	5	1	0.02	46.34	2.55E-02	1.00E+00	BP	2
mitochondrial RNA 3'-end processing (GO:0000965)	7	1	0.03	33.1	3.39E-02	1.00E+00	BP	2
regulation of intracellular transport (GO:0032386)	7	1	0.03	33.1	3.39E-02	1.00E+00	BP	2
protein deneddylation (GO:0000338)	9	1	0.04	25.74	4.22E-02	1.00E+00	BP	2
folic acid metabolic process (GO:0046655)	9	1	0.04	25.74	4.22E-02	1.00E+00	BP	2
cellular response to UV (GO:0034644)	10	1	0.04	23.17	4.63E-02	1.00E+00	BP	2
regulation of vesicle-mediated transport (GO:0060627)	10	1	0.04	23.17	4.63E-02	1.00E+00	BP	2
tetrahydrofolate biosynthetic process (GO:0046654)	11	1	0.05	21.06	5.04E-02	1.00E+00	BP	2
ER-associated misfolded protein catabolic process (GO:0071712)	25	2	0.11	18.54	6.00E-03	1.00E+00	BP	2
Mo-molybdopterin cofactor biosynthetic process (GO:0006777)	13	1	0.06	17.82	5.85E-02	1.00E+00	BP	2
Mo-molybdopterin cofactor metabolic process (GO:0019720)	13	1	0.06	17.82	5.85E-02	1.00E+00	BP	2
prosthetic group metabolic process (GO:0051189)	14	1	0.06	16.55	6.26E-02	1.00E+00	BP	2
molybdopterin cofactor metabolic process (GO:0043545)	14	1	0.06	16.55	6.26E-02	1.00E+00	BP	2
molybdopterin cofactor biosynthetic process (GO:0032324)	14	1	0.06	16.55	6.26E-02	1.00E+00	BP	2
response to misfolded protein (GO:0051788)	30	2	0.13	15.45	8.37E-03	1.00E+00	BP	2
cellular response to misfolded protein (GO:0071218)	30	2	0.13	15.45	8.37E-03	1.00E+00	BP	2
regulation of cellular localization (GO:0060341)	15	1	0.06	15.45	6.66E-02	1.00E+00	BP	2
gene silencing by RNA-directed DNA methylation (GO:0080188)	16	1	0.07	14.48	7.06E-02	1.00E+00	BP	2
DNA methylation-dependent heterochromatin formation (GO:0006346)	16	1	0.07	14.48	7.06E-02	1.00E+00	BP	2
L-ascorbic acid biosynthetic process (GO:0019853)	16	1	0.07	14.48	7.06E-02	1.00E+00	BP	2
facultative heterochromatin formation (GO:0140718)	16	1	0.07	14.48	7.06E-02	1.00E+00	BP	2
sucrose catabolic process (GO:0005987)	17	1	0.07	13.63	7.46E-02	1.00E+00	BP	2
megagametogenesis (GO:0009561)	17	1	0.07	13.63	7.46E-02	1.00E+00	BP	2

GO Term	Genome	Count	Expected	Fold Enrichment	P-value	FDR	Term	Cluster
histone H3-K4 methylation (GO:0051568)	17	1	0.07	13.63	7.46E-02	1.00E+00	BP	2
small ncRNA-mediated heterochromatin formation (GO:0031048)	18	1	0.08	12.87	7.86E-02	1.00E+00	BP	2
mitochondrial RNA processing (GO:0000963)	19	1	0.08	12.19	8.25E-02	1.00E+00	BP	2
folic acid-containing compound biosynthetic process (GO:0009396)	19	1	0.08	12.19	8.25E-02	1.00E+00	BP	2
intermembrane lipid transfer (GO:0120009)	19	1	0.08	12.19	8.25E-02	1.00E+00	BP	2
L-ascorbic acid metabolic process (GO:0019852)	20	1	0.09	11.59	8.65E-02	1.00E+00	BP	2
auxin polar transport (GO:0009926)	20	1	0.09	11.59	8.65E-02	1.00E+00	BP	2
disaccharide catabolic process (GO:0046352)	20	1	0.09	11.59	8.65E-02	1.00E+00	BP	2
pteridine-containing compound biosynthetic process (GO:0042559)	21	1	0.09	11.03	9.04E-02	1.00E+00	BP	2
response to high light intensity (GO:0009644)	21	1	0.09	11.03	9.04E-02	1.00E+00	BP	2
lactone biosynthetic process (GO:1901336)	21	1	0.09	11.03	9.04E-02	1.00E+00	BP	2
tetrahydrofolate metabolic process (GO:0046653)	23	1	0.1	10.07	9.82E-02	1.00E+00	BP	2
activation of GTPase activity (GO:0090630)	23	1	0.1	10.07	9.82E-02	1.00E+00	BP	2
regulation of secondary shoot formation (GO:2000032)	24	1	0.1	9.65	1.02E-01	1.00E+00	BP	2
oligosaccharide catabolic process (GO:0009313)	24	1	0.1	9.65	1.02E-01	1.00E+00	BP	2
regulation of morphogenesis of a branching structure (GO:0060688)	24	1	0.1	9.65	1.02E-01	1.00E+00	BP	2
protein quality control for misfolded or incompletely synthesized proteins (GO:0006515)	49	2	0.21	9.46	2.04E-02	1.00E+00	BP	2
lactone metabolic process (GO:1901334)	25	1	0.11	9.27	1.06E-01	1.00E+00	BP	2
spindle assembly (GO:0051225)	51	2	0.22	9.09	2.19E-02	1.00E+00	BP	2
regulation of plant organ formation (GO:1905428)	26	1	0.11	8.91	1.10E-01	1.00E+00	BP	2
gravitropism (GO:0009630)	26	1	0.11	8.91	1.10E-01	1.00E+00	BP	2
positive regulation of GTPase activity (GO:0043547)	27	1	0.12	8.58	1.14E-01	1.00E+00	BP	2
transcription by RNA polymerase I (GO:0006360)	27	1	0.12	8.58	1.14E-01	1.00E+00	BP	2
response to gravity (GO:0009629)	27	1	0.12	8.58	1.14E-01	1.00E+00	BP	2
alditol metabolic process (GO:0019400)	28	1	0.12	8.28	1.17E-01	1.00E+00	BP	2
glycerol metabolic process (GO:0006071)	28	1	0.12	8.28	1.17E-01	1.00E+00	BP	2
cellular response to topologically incorrect protein (GO:0035967)	59	2	0.25	7.85	2.85E-02	1.00E+00	BP	2
response to topologically incorrect protein (GO:0035966)	59	2	0.25	7.85	2.85E-02	1.00E+00	BP	2
embryo sac development (GO:0009553)	30	1	0.13	7.72	1.25E-01	1.00E+00	BP	2
auxin transport (GO:0060918)	30	1	0.13	7.72	1.25E-01	1.00E+00	BP	2
mitochondrial gene expression (GO:0140053)	61	2	0.26	7.6	3.02E-02	1.00E+00	BP	2
peptidyl-threonine dephosphorylation (GO:0035970)	61	2	0.26	7.6	3.02E-02	1.00E+00	BP	2
response to UV (GO:0009411)	31	1	0.13	7.47	1.29E-01	1.00E+00	BP	2
regulation of GTPase activity (GO:0043087)	31	1	0.13	7.47	1.29E-01	1.00E+00	BP	2
hormone transport (GO:0009914)	32	1	0.14	7.24	1.33E-01	1.00E+00	BP	2
protein targeting to vacuole (GO:0006623)	32	1	0.14	7.24	1.33E-01	1.00E+00	BP	2
positive regulation of hydrolase activity (GO:0051345)	34	1	0.15	6.81	1.40E-01	1.00E+00	BP	2
phospholipid transport (GO:0015914)	34	1	0.15	6.81	1.40E-01	1.00E+00	BP	2
folic acid-containing compound metabolic process (GO:0006760)	35	1	0.15	6.62	1.44E-01	1.00E+00	BP	2
water-soluble vitamin biosynthetic process (GO:0042364)	73	2	0.32	6.35	4.15E-02	1.00E+00	BP	2
cellular response to light stimulus (GO:0071482)	37	1	0.16	6.26	1.51E-01	1.00E+00	BP	2
pteridine-containing compound metabolic process (GO:0042558)	37	1	0.16	6.26	1.51E-01	1.00E+00	BP	2
cellular modified amino acid biosynthetic process (GO:0042398)	37	1	0.16	6.26	1.51E-01	1.00E+00	BP	2
mitochondrial translation (GO:0032543)	37	1	0.16	6.26	1.51E-01	1.00E+00	BP	2
response to light intensity (GO:0009642)	38	1	0.16	6.1	1.55E-01	1.00E+00	BP	2
protein complex oligomerization (GO:0051259)	40	1	0.17	5.79	1.62E-01	1.00E+00	BP	2
histone lysine methylation (GO:0034968)	40	1	0.17	5.79	1.62E-01	1.00E+00	BP	2
spindle organization (GO:0007051)	80	2	0.35	5.79	4.87E-02	1.00E+00	BP	2
ERAD pathway (GO:0036503)	81	2	0.35	5.72	4.98E-02	1.00E+00	BP	2
cellular response to radiation (GO:0071478)	41	1	0.18	5.65	1.66E-01	1.00E+00	BP	2
water-soluble vitamin metabolic process (GO:0006767)	83	2	0.36	5.58	5.20E-02	1.00E+00	BP	2

GO Term	Genome	Count	Expected	Fold Enrichment	P-value	FDR	Term	Cluster
iron-sulfur cluster assembly (GO:0016226)	42	1	0.18	5.52	1.69E-01	1.00E+00	BP	2
tRNA methylation (GO:0030488)	42	1	0.18	5.52	1.69E-01	1.00E+00	BP	2
xenobiotic export from cell (GO:0046618)	42	1	0.18	5.52	1.69E-01	1.00E+00	BP	2
xenobiotic detoxification by transmembrane export across the plasma membrane (GO:1990961)	42	1	0.18	5.52	1.69E-01	1.00E+00	BP	2
metallo-sulfur cluster assembly (GO:0031163)	42	1	0.18	5.52	1.69E-01	1.00E+00	BP	2
hydrogen peroxide catabolic process (GO:0042744)	171	4	0.74	5.42	7.04E-03	1.00E+00	BP	2
dicarboxylic acid biosynthetic process (GO:0043650)	43	1	0.19	5.39	1.73E-01	1.00E+00	BP	2
hydrogen peroxide metabolic process (GO:0042743)	172	4	0.74	5.39	7.18E-03	1.00E+00	BP	2
vitamin biosynthetic process (GO:0009110)	87	2	0.38	5.33	5.64E-02	1.00E+00	BP	2
response to organonitrogen compound (GO:0010243)	90	2	0.39	5.15	5.98E-02	1.00E+00	BP	2
mitochondrial RNA metabolic process (GO:0000959)	45	1	0.19	5.15	1.80E-01	1.00E+00	BP	2
sister chromatid cohesion (GO:0007062)	46	1	0.2	5.04	1.83E-01	1.00E+00	BP	2
protein dephosphorylation (GO:0006470)	186	4	0.8	4.98	9.33E-03	1.00E+00	BP	2
vitamin metabolic process (GO:0006766)	97	2	0.42	4.78	6.80E-02	1.00E+00	BP	2
monosaccharide biosynthetic process (GO:0046364)	49	1	0.21	4.73	1.94E-01	1.00E+00	BP	2
non-membrane-bounded organelle assembly (GO:0140694)	149	3	0.64	4.67	2.81E-02	1.00E+00	BP	2
reactive oxygen species metabolic process (GO:0072593)	200	4	0.86	4.63	1.19E-02	1.00E+00	BP	2
heterochromatin formation (GO:0031507)	51	1	0.22	4.54	2.01E-01	1.00E+00	BP	2
heterochromatin organization (GO:0070828)	51	1	0.22	4.54	2.01E-01	1.00E+00	BP	2
water transport (GO:0006833)	51	1	0.22	4.54	2.01E-01	1.00E+00	BP	2
fluid transport (GO:0042044)	51	1	0.22	4.54	2.01E-01	1.00E+00	BP	2
cellular response to environmental stimulus (GO:0104004)	52	1	0.22	4.46	2.04E-01	1.00E+00	BP	2
cellular response to abiotic stimulus (GO:0071214)	52	1	0.22	4.46	2.04E-01	1.00E+00	BP	2
tropism (GO:0009606)	53	1	0.23	4.37	2.08E-01	1.00E+00	BP	2
regulation of transport (GO:0051049)	53	1	0.23	4.37	2.08E-01	1.00E+00	BP	2
peptidyl-lysine methylation (GO:0018022)	54	1	0.23	4.29	2.11E-01	1.00E+00	BP	2
negative regulation of gene expression, epigenetic (GO:0045814)	55	1	0.24	4.21	2.14E-01	1.00E+00	BP	2
histone methylation (GO:0016571)	55	1	0.24	4.21	2.14E-01	1.00E+00	BP	2
cellular modified amino acid metabolic process (GO:0006575)	55	1	0.24	4.21	2.14E-01	1.00E+00	BP	2
response to nitrogen compound (GO:1901698)	112	2	0.48	4.14	8.66E-02	1.00E+00	BP	2
xenobiotic transport (GO:0042908)	58	1	0.25	3.99	2.25E-01	1.00E+00	BP	2
establishment of protein localization to vacuole (GO:0072666)	58	1	0.25	3.99	2.25E-01	1.00E+00	BP	2
protein localization to vacuole (GO:0072665)	58	1	0.25	3.99	2.25E-01	1.00E+00	BP	2
response to endoplasmic reticulum stress (GO:0034976)	119	2	0.51	3.89	9.58E-02	1.00E+00	BP	2
sucrose metabolic process (GO:0005985)	60	1	0.26	3.86	2.31E-01	1.00E+00	BP	2
dephosphorylation (GO:0016311)	248	4	1.07	3.74	2.38E-02	1.00E+00	BP	2
retrograde vesicle-mediated transport, Golgi to endoplasmic reticulum (GO:0006890)	63	1	0.27	3.68	2.41E-01	1.00E+00	BP	2
chromosome segregation (GO:0007059)	126	2	0.54	3.68	1.05E-01	1.00E+00	BP	2
organelle assembly (GO:0070925)	195	3	0.84	3.56	5.42E-02	1.00E+00	BP	2
cellular oxidant detoxification (GO:0098869)	266	4	1.15	3.48	2.96E-02	1.00E+00	BP	2
detoxification (GO:0098754)	334	5	1.44	3.47	1.59E-02	1.00E+00	BP	2
regulation of localization (GO:0032879)	67	1	0.29	3.46	2.54E-01	1.00E+00	BP	2
export across plasma membrane (GO:0140115)	67	1	0.29	3.46	2.54E-01	1.00E+00	BP	2
cellular response to toxic substance (GO:0097237)	283	4	1.22	3.27	3.58E-02	1.00E+00	BP	2
cellular detoxification (GO:1990748)	283	4	1.22	3.27	3.58E-02	1.00E+00	BP	2
response to toxic substance (GO:0009636)	358	5	1.55	3.24	2.07E-02	1.00E+00	BP	2
ribosome assembly (GO:0042255)	72	1	0.31	3.22	2.70E-01	1.00E+00	BP	2
epigenetic regulation of gene expression (GO:0040029)	72	1	0.31	3.22	2.70E-01	1.00E+00	BP	2
regulation of anatomical structure morphogenesis (GO:0022603)	73	1	0.32	3.17	2.73E-01	1.00E+00	BP	2
response to oxidative stress (GO:0006979)	319	4	1.38	2.91	5.13E-02	1.00E+00	BP	2
gametophyte development (GO:0048229)	81	1	0.35	2.86	2.98E-01	1.00E+00	BP	2

GO Term	Genome	Count	Expected	Fold Enrichment	P-value	FDR	Term	Cluster
protein methylation (GO:0006479)	85	1	0.37	2.73	3.10E-01	1.00E+00	BP	2
response to wounding (GO:0009611)	85	1	0.37	2.73	3.10E-01	1.00E+00	BP	2
protein alkylation (GO:0008213)	85	1	0.37	2.73	3.10E-01	1.00E+00	BP	2
polyol metabolic process (GO:0019751)	87	1	0.38	2.66	3.16E-01	1.00E+00	BP	2
tRNA aminoacylation for protein translation (GO:0006418)	88	1	0.38	2.63	3.19E-01	1.00E+00	BP	2
microtubule cytoskeleton organization (GO:0000226)	181	2	0.78	2.56	1.86E-01	1.00E+00	BP	2
regulation of growth (GO:0040008)	92	1	0.4	2.52	3.30E-01	1.00E+00	BP	2
positive regulation of catalytic activity (GO:0043085)	93	1	0.4	2.49	3.33E-01	1.00E+00	BP	2
protein autophosphorylation (GO:0046777)	94	1	0.41	2.46	3.36E-01	1.00E+00	BP	2
tRNA aminoacylation (GO:0043039)	94	1	0.41	2.46	3.36E-01	1.00E+00	BP	2
amino acid activation (GO:0043038)	95	1	0.41	2.44	3.39E-01	1.00E+00	BP	2
translational elongation (GO:0006414)	96	1	0.41	2.41	3.42E-01	1.00E+00	BP	2
amino acid transmembrane transport (GO:0003333)	97	1	0.42	2.39	3.45E-01	1.00E+00	BP	2
RNA methylation (GO:0001510)	98	1	0.42	2.36	3.47E-01	1.00E+00	BP	2
macromolecule methylation (GO:0043414)	198	2	0.85	2.34	2.12E-01	1.00E+00	BP	2
proteasomal protein catabolic process (GO:0010498)	305	3	1.32	2.28	1.47E-01	1.00E+00	BP	2
positive regulation of molecular function (GO:0044093)	102	1	0.44	2.27	3.59E-01	1.00E+00	BP	2
disaccharide metabolic process (GO:0005984)	103	1	0.44	2.25	3.61E-01	1.00E+00	BP	2
amino acid transport (GO:0006865)	105	1	0.45	2.21	3.67E-01	1.00E+00	BP	2
tRNA modification (GO:0006400)	106	1	0.46	2.19	3.70E-01	1.00E+00	BP	2
vacuolar transport (GO:0007034)	107	1	0.46	2.17	3.72E-01	1.00E+00	BP	2
oligosaccharide metabolic process (GO:0009311)	111	1	0.48	2.09	3.83E-01	1.00E+00	BP	2
RNA 3'-end processing (GO:0031123)	111	1	0.48	2.09	3.83E-01	1.00E+00	BP	2
RNA-mediated gene silencing (GO:0031047)	117	1	0.5	1.98	3.99E-01	1.00E+00	BP	2
dicarboxylic acid metabolic process (GO:0043648)	117	1	0.5	1.98	3.99E-01	1.00E+00	BP	2
organic acid transmembrane transport (GO:1903825)	127	1	0.55	1.82	4.24E-01	1.00E+00	BP	2
carboxylic acid transmembrane transport (GO:1905039)	127	1	0.55	1.82	4.24E-01	1.00E+00	BP	2
negative regulation of catalytic activity (GO:0043086)	128	1	0.55	1.81	4.27E-01	1.00E+00	BP	2
negative regulation of molecular function (GO:0044092)	131	1	0.57	1.77	4.34E-01	1.00E+00	BP	2
regulation of hormone levels (GO:0010817)	133	1	0.57	1.74	4.39E-01	1.00E+00	BP	2
cellular response to chemical stimulus (GO:0070887)	934	7	4.03	1.74	1.29E-01	1.00E+00	BP	2
methylation (GO:0032259)	541	4	2.33	1.71	3.02E-01	1.00E+00	BP	2
tRNA metabolic process (GO:0006399)	273	2	1.18	1.7	3.31E-01	1.00E+00	BP	2
peptidyl-serine modification (GO:0018209)	139	1	0.6	1.67	4.53E-01	1.00E+00	BP	2
peptidyl-serine phosphorylation (GO:0018105)	139	1	0.6	1.67	4.53E-01	1.00E+00	BP	2
organophosphate ester transport (GO:0015748)	141	1	0.61	1.64	4.58E-01	1.00E+00	BP	2
cell cycle process (GO:0022402)	425	3	1.83	1.64	4.36E-01	1.00E+00	BP	2
endoplasmic reticulum to Golgi vesicle-mediated transport (GO:0006888)	142	1	0.61	1.63	4.60E-01	1.00E+00	BP	2
histone modification (GO:0016570)	143	1	0.62	1.62	4.63E-01	1.00E+00	BP	2
regulation of hydrolase activity (GO:0051336)	143	1	0.62	1.62	4.63E-01	1.00E+00	BP	2
RNA catabolic process (GO:0006401)	146	1	0.63	1.59	4.70E-01	1.00E+00	BP	2
microtubule-based process (GO:0007017)	293	2	1.26	1.58	3.62E-01	1.00E+00	BP	2
tRNA processing (GO:0008033)	148	1	0.64	1.57	4.74E-01	1.00E+00	BP	2
regulation of multicellular organismal process (GO:0051239)	150	1	0.65	1.54	4.79E-01	1.00E+00	BP	2
lipid transport (GO:0006869)	150	1	0.65	1.54	4.79E-01	1.00E+00	BP	2
alcohol metabolic process (GO:0006066)	156	1	0.67	1.49	4.92E-01	1.00E+00	BP	2
cellular catabolic process (GO:0044248)	783	5	3.38	1.48	3.97E-01	1.00E+00	BP	2
export from cell (GO:0140352)	159	1	0.69	1.46	4.99E-01	1.00E+00	BP	2
lipid localization (GO:0010876)	163	1	0.7	1.42	5.07E-01	1.00E+00	BP	2
double-strand break repair via homologous recombination (GO:0000724)	164	1	0.71	1.41	5.09E-01	1.00E+00	BP	2
regulation of catalytic activity (GO:0050790)	330	2	1.42	1.4	6.56E-01	1.00E+00	BP	2

GO Term	Genome	Count	Expected	Fold Enrichment	P-value	FDR	Term	Cluster
response to chemical (GO:0042221)	1513	9	6.53	1.38	3.13E-01	1.00E+00	BP	2
cytoskeleton organization (GO:0007010)	340	2	1.47	1.36	6.61E-01	1.00E+00	BP	2
recombinational repair (GO:0000725)	171	1	0.74	1.35	5.24E-01	1.00E+00	BP	2
regulation of molecular function (GO:0065009)	344	2	1.48	1.35	6.63E-01	1.00E+00	BP	2
carboxylic acid transport (GO:0046942)	173	1	0.75	1.34	5.28E-01	1.00E+00	BP	2
organic acid transport (GO:0015849)	173	1	0.75	1.34	5.28E-01	1.00E+00	BP	2
protein glycosylation (GO:0006486)	185	1	0.8	1.25	5.52E-01	1.00E+00	BP	2
macromolecule glycosylation (GO:0043413)	185	1	0.8	1.25	5.52E-01	1.00E+00	BP	2
glycoprotein biosynthetic process (GO:0009101)	187	1	0.81	1.24	5.56E-01	1.00E+00	BP	2
protein modification process (GO:0036211)	3194	17	13.79	1.23	3.96E-01	1.00E+00	BP	2
glycosylation (GO:0070085)	191	1	0.82	1.21	5.63E-01	1.00E+00	BP	2
protein ubiquitination (GO:0016567)	573	3	2.47	1.21	7.40E-01	1.00E+00	BP	2
Unclassified (UNCLASSIFIED)	17419	91	75.18	1.21	1.63E-02	1.00E+00	BP	2
cellular response to organic substance (GO:0071310)	578	3	2.49	1.2	7.42E-01	1.00E+00	BP	2
cellular component assembly (GO:0022607)	774	4	3.34	1.2	5.80E-01	1.00E+00	BP	2
cell cycle (GO:0007049)	586	3	2.53	1.19	7.43E-01	1.00E+00	BP	2
macromolecule modification (GO:0043412)	3738	19	16.13	1.18	4.31E-01	1.00E+00	BP	2
nucleobase-containing compound catabolic process (GO:0034655)	198	1	0.85	1.17	5.76E-01	1.00E+00	BP	2
phosphate-containing compound metabolic process (GO:0006796)	2984	15	12.88	1.16	5.59E-01	1.00E+00	BP	2
ubiquitin-dependent protein catabolic process (GO:0006511)	601	3	2.59	1.16	7.47E-01	1.00E+00	BP	2
phosphorus metabolic process (GO:0006793)	3030	15	13.08	1.15	5.63E-01	1.00E+00	BP	2
response to abiotic stimulus (GO:0009628)	611	3	2.64	1.14	7.50E-01	1.00E+00	BP	2
modification-dependent protein catabolic process (GO:0019941)	614	3	2.65	1.13	7.51E-01	1.00E+00	BP	2
glycoprotein metabolic process (GO:0009100)	205	1	0.88	1.13	5.89E-01	1.00E+00	BP	2
double-strand break repair (GO:0006302)	205	1	0.88	1.13	5.89E-01	1.00E+00	BP	2
DNA-templated transcription (GO:0006351)	824	4	3.56	1.12	7.84E-01	1.00E+00	BP	2
protein modification by small protein conjugation (GO:0032446)	618	3	2.67	1.12	7.52E-01	1.00E+00	BP	2
organic substance catabolic process (GO:1901575)	1863	9	8.04	1.12	7.15E-01	1.00E+00	BP	2
chromatin organization (GO:0006325)	417	2	1.8	1.11	7.02E-01	1.00E+00	BP	2
RNA biosynthetic process (GO:0032774)	840	4	3.63	1.1	7.87E-01	1.00E+00	BP	2
protein modification by small protein conjugation or removal (GO:0070647)	847	4	3.66	1.09	7.88E-01	1.00E+00	BP	2
modification-dependent macromolecule catabolic process (GO:0043632)	636	3	2.74	1.09	7.57E-01	1.00E+00	BP	2
heterocycle biosynthetic process (GO:0018130)	1487	7	6.42	1.09	6.89E-01	1.00E+00	BP	2
catabolic process (GO:0009056)	1947	9	8.4	1.07	7.25E-01	1.00E+00	BP	2
phosphorylation (GO:0016310)	2164	10	9.34	1.07	7.37E-01	1.00E+00	BP	2
protein phosphorylation (GO:0006468)	1543	7	6.66	1.05	8.42E-01	1.00E+00	BP	2
regulation of RNA biosynthetic process (GO:2001141)	2705	12	11.67	1.03	8.79E-01	1.00E+00	BP	2
regulation of DNA-templated transcription (GO:0006355)	2705	12	11.67	1.03	8.79E-01	1.00E+00	BP	2
RNA modification (GO:0009451)	453	2	1.96	1.02	7.23E-01	1.00E+00	BP	2
response to stress (GO:0006950)	2296	10	9.91	1.01	8.70E-01	1.00E+00	BP	2
mRNA processing (GO:0006397)	460	2	1.99	1.01	1.00E+00	1.00E+00	BP	2
protein modification by small protein removal (GO:0070646)	230	1	0.99	1.01	1.00E+00	1.00E+00	BP	2
sulfur compound metabolic process (GO:0006790)	231	1	1	1	1.00E+00	1.00E+00	BP	2
intracellular signal transduction (GO:0035556)	463	2	2	1	1.00E+00	1.00E+00	BP	2
heterocycle catabolic process (GO:0046700)	233	1	1.01	0.99	1.00E+00	1.00E+00	BP	2
cellular nitrogen compound catabolic process (GO:0044270)	235	1	1.01	0.99	1.00E+00	1.00E+00	BP	2
regulation of RNA metabolic process (GO:0051252)	2843	12	12.27	0.98	1.00E+00	1.00E+00	BP	2
monosaccharide metabolic process (GO:0005996)	238	1	1.03	0.97	1.00E+00	1.00E+00	BP	2
RNA metabolic process (GO:0016070)	2384	10	10.29	0.97	1.00E+00	1.00E+00	BP	2
protein metabolic process (GO:0019538)	5254	22	22.68	0.97	1.00E+00	1.00E+00	BP	2
organic cyclic compound biosynthetic process (GO:1901362)	1677	7	7.24	0.97	1.00E+00	1.00E+00	BP	2

GO Term	Genome	Count	Expected	Fold Enrichment	P-value	FDR	Term	Cluster
peptidyl-lysine modification (GO:0018205)	240	1	1.04	0.97	1.00E+00	1.00E+00	BP	2
response to light stimulus (GO:0009416)	240	1	1.04	0.97	1.00E+00	1.00E+00	BP	2
protein-DNA complex organization (GO:0071824)	481	2	2.08	0.96	1.00E+00	1.00E+00	BP	2
proteolysis involved in protein catabolic process (GO:0051603)	726	3	3.13	0.96	1.00E+00	1.00E+00	BP	2
regulation of nucleobase-containing compound metabolic process (GO:0019219)	2913	12	12.57	0.95	1.00E+00	1.00E+00	BP	2
regulation of developmental process (GO:0050793)	245	1	1.06	0.95	1.00E+00	1.00E+00	BP	2
regulation of macromolecule biosynthetic process (GO:0010556)	2942	12	12.7	0.95	1.00E+00	1.00E+00	BP	2
protein catabolic process (GO:0030163)	738	3	3.19	0.94	1.00E+00	1.00E+00	BP	2
cellular response to stimulus (GO:0051716)	2710	11	11.7	0.94	1.00E+00	1.00E+00	BP	2
regulation of cellular biosynthetic process (GO:0031326)	2959	12	12.77	0.94	1.00E+00	1.00E+00	BP	2
regulation of biosynthetic process (GO:0009889)	2969	12	12.81	0.94	1.00E+00	1.00E+00	BP	2
response to organic substance (GO:0010033)	1000	4	4.32	0.93	1.00E+00	1.00E+00	BP	2
protein targeting (GO:0006605)	252	1	1.09	0.92	1.00E+00	1.00E+00	BP	2
Golgi vesicle transport (GO:0048193)	253	1	1.09	0.92	1.00E+00	1.00E+00	BP	2
regulation of gene expression (GO:0010468)	3303	13	14.26	0.91	8.89E-01	1.00E+00	BP	2
biological regulation (GO:0065007)	5626	22	24.28	0.91	7.41E-01	1.00E+00	BP	2
response to radiation (GO:0009314)	259	1	1.12	0.89	1.00E+00	1.00E+00	BP	2
proteasome-mediated ubiquitin-dependent protein catabolic process (GO:0043161)	259	1	1.12	0.89	1.00E+00	1.00E+00	BP	2
response to stimulus (GO:0050896)	4415	17	19.05	0.89	7.15E-01	1.00E+00	BP	2
RNA processing (GO:0006396)	1041	4	4.49	0.89	1.00E+00	1.00E+00	BP	2
organic acid biosynthetic process (GO:0016053)	521	2	2.25	0.89	1.00E+00	1.00E+00	BP	2
carboxylic acid biosynthetic process (GO:0046394)	521	2	2.25	0.89	1.00E+00	1.00E+00	BP	2
macromolecule catabolic process (GO:0009057)	1069	4	4.61	0.87	1.00E+00	1.00E+00	BP	2
electron transport chain (GO:0022900)	269	1	1.16	0.86	1.00E+00	1.00E+00	BP	2
peptidyl-amino acid modification (GO:0018193)	544	2	2.35	0.85	1.00E+00	1.00E+00	BP	2
regulation of cellular process (GO:0050794)	4625	17	19.96	0.85	5.51E-01	1.00E+00	BP	2
regulation of cellular metabolic process (GO:0031323)	3270	12	14.11	0.85	6.76E-01	1.00E+00	BP	2
macromolecule metabolic process (GO:0043170)	8871	32	38.29	0.84	2.70E-01	1.00E+00	BP	2
metabolic process (GO:0008152)	13613	49	58.75	0.83	1.25E-01	1.00E+00	BP	2
biological_process (GO:0008150)	21970	79	94.82	0.83	1.63E-02	1.00E+00	BP	2
cellular metabolic process (GO:0044237)	9471	34	40.88	0.83	2.42E-01	1.00E+00	BP	2
regulation of biological process (GO:0050789)	5297	19	22.86	0.83	4.32E-01	1.00E+00	BP	2
carboxylic acid metabolic process (GO:0019752)	1117	4	4.82	0.83	1.00E+00	1.00E+00	BP	2
regulation of nitrogen compound metabolic process (GO:0051171)	3361	12	14.51	0.83	5.83E-01	1.00E+00	BP	2
organonitrogen compound metabolic process (GO:1901564)	6443	23	27.81	0.83	3.51E-01	1.00E+00	BP	2
oxoacid metabolic process (GO:0043436)	1130	4	4.88	0.82	1.00E+00	1.00E+00	BP	2
organic acid metabolic process (GO:0006082)	1131	4	4.88	0.82	1.00E+00	1.00E+00	BP	2
response to auxin (GO:0009733)	283	1	1.22	0.82	1.00E+00	1.00E+00	BP	2
regulation of primary metabolic process (GO:0080090)	3400	12	14.67	0.82	5.83E-01	1.00E+00	BP	2
regulation of macromolecule metabolic process (GO:0060255)	3684	13	15.9	0.82	5.11E-01	1.00E+00	BP	2
regulation of cell cycle (GO:0051726)	284	1	1.23	0.82	1.00E+00	1.00E+00	BP	2
aromatic compound catabolic process (GO:0019439)	285	1	1.23	0.81	1.00E+00	1.00E+00	BP	2
membrane organization (GO:0061024)	287	1	1.24	0.81	1.00E+00	1.00E+00	BP	2
transmembrane transport (GO:0055085)	1724	6	7.44	0.81	8.50E-01	1.00E+00	BP	2
organic substance metabolic process (GO:0071704)	12450	43	53.73	0.8	8.25E-02	1.00E+00	BP	2
regulation of metabolic process (GO:0019222)	3766	13	16.25	0.8	5.12E-01	1.00E+00	BP	2
organic cyclic compound catabolic process (GO:1901361)	290	1	1.25	0.8	1.00E+00	1.00E+00	BP	2
nitrogen compound metabolic process (GO:0006807)	9609	33	41.47	0.8	1.52E-01	1.00E+00	BP	2
response to external stimulus (GO:0009605)	583	2	2.52	0.79	1.00E+00	1.00E+00	BP	2
establishment of protein localization to organelle (GO:0072594)	295	1	1.27	0.79	1.00E+00	1.00E+00	BP	2
organic hydroxy compound metabolic process (GO:1901615)	296	1	1.28	0.78	1.00E+00	1.00E+00	BP	2

GO Term	Genome	Count	Expected	Fold Enrichment	P-value	FDR	Term	Cluster
heterocycle metabolic process (GO:0046483)	4164	14	17.97	0.78	3.81E-01	1.00E+00	BP	2
organic anion transport (GO:0015711)	300	1	1.29	0.77	1.00E+00	1.00E+00	BP	2
cellular process (GO:0009987)	15372	51	66.34	0.77	1.78E-02	1.00E+00	BP	2
mRNA metabolic process (GO:0016071)	615	2	2.65	0.75	1.00E+00	1.00E+00	BP	2
nucleic acid metabolic process (GO:0090304)	3395	11	14.65	0.75	4.10E-01	1.00E+00	BP	2
proteolysis (GO:0006508)	1544	5	6.66	0.75	6.91E-01	1.00E+00	BP	2
aromatic compound biosynthetic process (GO:0019438)	1547	5	6.68	0.75	6.91E-01	1.00E+00	BP	2
organic cyclic compound metabolic process (GO:1901360)	4430	14	19.12	0.73	2.72E-01	1.00E+00	BP	2
nucleobase-containing compound biosynthetic process (GO:0034654)	1266	4	5.46	0.73	6.66E-01	1.00E+00	BP	2
organonitrogen compound catabolic process (GO:1901565)	955	3	4.12	0.73	8.02E-01	1.00E+00	BP	2
ncRNA metabolic process (GO:0034660)	637	2	2.75	0.73	1.00E+00	1.00E+00	BP	2
transport (GO:0006810)	3186	10	13.75	0.73	3.95E-01	1.00E+00	BP	2
establishment of localization (GO:0051234)	3225	10	13.92	0.72	3.27E-01	1.00E+00	BP	2
primary metabolic process (GO:0044238)	11484	35	49.56	0.71	1.40E-02	1.00E+00	BP	2
regulation of transcription by RNA polymerase II (GO:0006357)	659	2	2.84	0.7	1.00E+00	1.00E+00	BP	2
localization (GO:0051179)	3340	10	14.42	0.69	2.69E-01	1.00E+00	BP	2
protein-containing complex organization (GO:0043933)	1011	3	4.36	0.69	8.05E-01	1.00E+00	BP	2
negative regulation of gene expression (GO:0010629)	342	1	1.48	0.68	1.00E+00	1.00E+00	BP	2
chromatin remodeling (GO:0006338)	346	1	1.49	0.67	1.00E+00	1.00E+00	BP	2
carbohydrate catabolic process (GO:0016052)	346	1	1.49	0.67	1.00E+00	1.00E+00	BP	2
protein localization to organelle (GO:0033365)	346	1	1.49	0.67	1.00E+00	1.00E+00	BP	2
cellular nitrogen compound biosynthetic process (GO:0044271)	2423	7	10.46	0.67	3.37E-01	1.00E+00	BP	2
macromolecule biosynthetic process (GO:0009059)	2436	7	10.51	0.67	3.37E-01	1.00E+00	BP	2
vesicle-mediated transport (GO:0016192)	699	2	3.02	0.66	7.73E-01	1.00E+00	BP	2
cellular component biogenesis (GO:0044085)	1401	4	6.05	0.66	5.33E-01	1.00E+00	BP	2
gene expression (GO:0010467)	2102	6	9.07	0.66	3.90E-01	1.00E+00	BP	2
nucleobase-containing compound metabolic process (GO:0006139)	3868	11	16.69	0.66	1.56E-01	1.00E+00	BP	2
amide biosynthetic process (GO:0043604)	1059	3	4.57	0.66	6.34E-01	1.00E+00	BP	2
small molecule biosynthetic process (GO:0044283)	708	2	3.06	0.65	7.74E-01	1.00E+00	BP	2
cellular aromatic compound metabolic process (GO:0006725)	4327	12	18.68	0.64	1.10E-01	1.00E+00	BP	2
cellular component organization (GO:0016043)	2935	8	12.67	0.63	2.38E-01	1.00E+00	BP	2
positive regulation of DNA-templated transcription (GO:0045893)	376	1	1.62	0.62	1.00E+00	1.00E+00	BP	2
positive regulation of RNA biosynthetic process (GO:1902680)	376	1	1.62	0.62	1.00E+00	1.00E+00	BP	2
cellular nitrogen compound metabolic process (GO:0034641)	4908	13	21.18	0.61	6.17E-02	1.00E+00	BP	2
response to hormone (GO:0009725)	756	2	3.26	0.61	7.76E-01	1.00E+00	BP	2
small molecule metabolic process (GO:0044281)	1894	5	8.17	0.61	3.65E-01	1.00E+00	BP	2
response to endogenous stimulus (GO:0009719)	762	2	3.29	0.61	7.76E-01	1.00E+00	BP	2
cellular biosynthetic process (GO:0044249)	3461	9	14.94	0.6	1.33E-01	1.00E+00	BP	2
amide metabolic process (GO:0043603)	1168	3	5.04	0.6	4.96E-01	1.00E+00	BP	2
positive regulation of macromolecule biosynthetic process (GO:0010557)	394	1	1.7	0.59	1.00E+00	1.00E+00	BP	2
carbohydrate biosynthetic process (GO:0016051)	396	1	1.71	0.59	1.00E+00	1.00E+00	BP	2
positive regulation of cellular biosynthetic process (GO:0031328)	399	1	1.72	0.58	1.00E+00	1.00E+00	BP	2
organonitrogen compound biosynthetic process (GO:1901566)	2013	5	8.69	0.58	2.90E-01	1.00E+00	BP	2
positive regulation of biosynthetic process (GO:0009891)	403	1	1.74	0.57	1.00E+00	1.00E+00	BP	2
cellular response to stress (GO:0033554)	1229	3	5.3	0.57	5.01E-01	1.00E+00	BP	2
organic substance biosynthetic process (GO:1901576)	4099	10	17.69	0.57	5.77E-02	1.00E+00	BP	2
signal transduction (GO:0007165)	1258	3	5.43	0.55	3.83E-01	1.00E+00	BP	2
organelle organization (GO:0006996)	1700	4	7.34	0.55	2.57E-01	1.00E+00	BP	2
signaling (GO:0023052)	1277	3	5.51	0.54	3.83E-01	1.00E+00	BP	2
biosynthetic process (GO:0009058)	4259	10	18.38	0.54	3.51E-02	1.00E+00	BP	2
cellular component organization or biogenesis (GO:0071840)	3437	8	14.83	0.54	7.45E-02	1.00E+00	BP	2

GO Term	Genome	Count	Expected	Fold Enrichment	P-value	FDR	Term	Cluster
regulation of biological quality (GO:0065008)	433	1	1.87	0.54	1.00E+00	1.00E+00	BP	2
organic substance transport (GO:0071702)	1759	4	7.59	0.53	2.59E-01	1.00E+00	BP	2
cell communication (GO:0007154)	1326	3	5.72	0.52	3.88E-01	1.00E+00	BP	2
carbohydrate metabolic process (GO:0005975)	1347	3	5.81	0.52	2.93E-01	1.00E+00	BP	2
positive regulation of RNA metabolic process (GO:0051254)	469	1	2.02	0.49	7.27E-01	1.00E+00	BP	2
monocarboxylic acid metabolic process (GO:0032787)	472	1	2.04	0.49	7.27E-01	1.00E+00	BP	2
translation (GO:0006412)	947	2	4.09	0.49	4.48E-01	1.00E+00	BP	2
generation of precursor metabolites and energy (GO:0006091)	480	1	2.07	0.48	7.27E-01	1.00E+00	BP	2
peptide biosynthetic process (GO:0043043)	965	2	4.16	0.48	4.50E-01	1.00E+00	BP	2
positive regulation of nucleobase-containing compound metabolic process (GO:0045935)	488	1	2.11	0.47	7.28E-01	1.00E+00	BP	2
hormone-mediated signaling pathway (GO:0009755)	497	1	2.15	0.47	7.28E-01	1.00E+00	BP	2
ribosome biogenesis (GO:0042254)	499	1	2.15	0.46	7.29E-01	1.00E+00	BP	2
cellular response to hormone stimulus (GO:0032870)	504	1	2.18	0.46	7.29E-01	1.00E+00	BP	2
peptide metabolic process (GO:0006518)	1013	2	4.37	0.46	3.34E-01	1.00E+00	BP	2
cellular response to endogenous stimulus (GO:0071495)	510	1	2.2	0.45	7.30E-01	1.00E+00	BP	2
carbohydrate derivative biosynthetic process (GO:1901137)	512	1	2.21	0.45	7.30E-01	1.00E+00	BP	2
ncRNA processing (GO:0034470)	521	1	2.25	0.44	7.31E-01	1.00E+00	BP	2
positive regulation of cellular metabolic process (GO:0031325)	535	1	2.31	0.43	7.33E-01	1.00E+00	BP	2
organophosphate biosynthetic process (GO:0090407)	541	1	2.33	0.43	7.34E-01	1.00E+00	BP	2
protein-containing complex assembly (GO:0065003)	554	1	2.39	0.42	7.36E-01	1.00E+00	BP	2
amino acid metabolic process (GO:0006520)	555	1	2.4	0.42	7.36E-01	1.00E+00	BP	2
positive regulation of nitrogen compound metabolic process (GO:0051173)	593	1	2.56	0.39	5.26E-01	1.00E+00	BP	2
positive regulation of cellular process (GO:0048522)	597	1	2.58	0.39	5.27E-01	1.00E+00	BP	2
negative regulation of macromolecule metabolic process (GO:0010605)	602	1	2.6	0.38	5.27E-01	1.00E+00	BP	2
chromosome organization (GO:0051276)	603	1	2.6	0.38	5.27E-01	1.00E+00	BP	2
positive regulation of macromolecule metabolic process (GO:0010604)	603	1	2.6	0.38	5.27E-01	1.00E+00	BP	2
ribonucleoprotein complex biogenesis (GO:0022613)	613	1	2.65	0.38	5.29E-01	1.00E+00	BP	2
negative regulation of metabolic process (GO:0009892)	615	1	2.65	0.38	5.29E-01	1.00E+00	BP	2
intracellular protein transport (GO:0006886)	617	1	2.66	0.38	5.29E-01	1.00E+00	BP	2
positive regulation of metabolic process (GO:0009893)	620	1	2.68	0.37	5.30E-01	1.00E+00	BP	2
multicellular organism development (GO:0007275)	627	1	2.71	0.37	5.31E-01	1.00E+00	BP	2
macromolecule localization (GO:0033036)	1264	2	5.46	0.37	1.85E-01	1.00E+00	BP	2
DNA recombination (GO:0006310)	648	1	2.8	0.36	5.35E-01	1.00E+00	BP	2
multicellular organismal process (GO:0032501)	686	1	2.96	0.34	3.78E-01	1.00E+00	BP	2
nitrogen compound transport (GO:0071705)	1381	2	5.96	0.34	1.37E-01	1.00E+00	BP	2
positive regulation of biological process (GO:0048518)	749	1	3.23	0.31	3.87E-01	1.00E+00	BP	2
carbohydrate derivative metabolic process (GO:1901135)	763	1	3.29	0.3	3.90E-01	1.00E+00	BP	2
negative regulation of biological process (GO:0048519)	801	1	3.46	0.29	2.71E-01	1.00E+00	BP	2
organophosphate metabolic process (GO:0019637)	808	1	3.49	0.29	2.72E-01	1.00E+00	BP	2
anatomical structure development (GO:0048856)	825	1	3.56	0.28	2.74E-01	1.00E+00	BP	2
DNA repair (GO:0006281)	872	1	3.76	0.27	1.92E-01	1.00E+00	BP	2
intracellular transport (GO:0046907)	896	1	3.87	0.26	1.93E-01	1.00E+00	BP	2
DNA damage response (GO:0006974)	901	1	3.89	0.26	1.93E-01	1.00E+00	BP	2
establishment of localization in cell (GO:0051649)	932	1	4.02	0.25	1.97E-01	1.00E+00	BP	2
protein transport (GO:0015031)	950	1	4.1	0.24	1.99E-01	1.00E+00	BP	2
establishment of protein localization (GO:0045184)	962	1	4.15	0.24	1.36E-01	1.00E+00	BP	2
developmental process (GO:0032502)	965	1	4.16	0.24	1.36E-01	1.00E+00	BP	2
protein localization (GO:0008104)	1049	1	4.53	0.22	1.42E-01	1.00E+00	BP	2
cellular macromolecule localization (GO:0070727)	1050	1	4.53	0.22	1.42E-01	1.00E+00	BP	2
DNA metabolic process (GO:0006259)	1050	1	4.53	0.22	1.42E-01	1.00E+00	BP	2
lipid metabolic process (GO:0006629)	1209	1	5.22	0.19	6.90E-02	1.00E+00	BP	2

GO Term	Genome	Count	Expected	Fold Enrichment	P-value	FDR	Term	Cluster
cellular localization (GO:0051641)	1297	1	5.6	0.18	4.82E-02	1.00E+00	BP	2
2-amino-4-hydroxy-6-hydroxymethyldihydropteridine diphosphokinase activity (GO:0003848)	1	1	0	> 100	8.58E-03	1.00E+00	MF	2
tRNA (guanosine-2'-O-)-methyltransferase activity (GO:0009020)	1	1	0	> 100	8.58E-03	1.00E+00	MF	2
dihydropteroate synthase activity (GO:0004156)	1	1	0	> 100	8.58E-03	1.00E+00	MF	2
tRNA 2'-O-methyltransferase activity (GO:0106050)	2	1	0.01	> 100	1.28E-02	1.00E+00	MF	2
molybdenum cofactor sulfurtransferase activity (GO:0102867)	2	1	0.01	> 100	1.28E-02	1.00E+00	MF	2
Mo-molybdopterin cofactor sulfurase activity (GO:0008265)	2	1	0.01	> 100	1.28E-02	1.00E+00	MF	2
deNEDDylase activity (GO:0019784)	2	1	0.01	> 100	1.28E-02	1.00E+00	MF	2
histidine-tRNA ligase activity (GO:0004821)	3	1	0.01	77.23	1.71E-02	1.00E+00	MF	2
CoA pyrophosphatase activity (GO:0010945)	4	1	0.02	57.92	2.13E-02	1.00E+00	MF	2
RNA 2'-O-methyltransferase activity (GO:0062105)	5	1	0.02	46.34	2.55E-02	1.00E+00	MF	2
L-gulonolactone oxidase activity (GO:0050105)	5	1	0.02	46.34	2.55E-02	1.00E+00	MF	2
phosphatidylinositol transfer activity (GO:0008526)	7	1	0.03	33.1	3.39E-02	1.00E+00	MF	2
microtubule minus-end binding (GO:0051011)	8	1	0.03	28.96	3.80E-02	1.00E+00	MF	2
ATPase activator activity (GO:0001671)	9	1	0.04	25.74	4.22E-02	1.00E+00	MF	2
ribonucleoside binding (GO:0032549)	9	1	0.04	25.74	4.22E-02	1.00E+00	MF	2
nucleoside binding (GO:0001882)	9	1	0.04	25.74	4.22E-02	1.00E+00	MF	2
mRNA 3'-UTR binding (GO:0003730)	28	3	0.12	24.82	3.21E-04	8.07E-01	MF	2
sucrose alpha-glucosidase activity (GO:0004575)	11	1	0.05	21.06	5.04E-02	1.00E+00	MF	2
glycopeptide alpha-N-acetylgalactosaminidase activity (GO:0033926)	11	1	0.05	21.06	5.04E-02	1.00E+00	MF	2
alpha-glucosidase activity (GO:0090599)	13	1	0.06	17.82	5.85E-02	1.00E+00	MF	2
D-arabinono-1,4-lactone oxidase activity (GO:0003885)	13	1	0.06	17.82	5.85E-02	1.00E+00	MF	2
molybdenum ion binding (GO:0030151)	14	1	0.06	16.55	6.26E-02	1.00E+00	MF	2
glycerophosphodiester phosphodiesterase activity (GO:0008889)	14	1	0.06	16.55	6.26E-02	1.00E+00	MF	2
RNA polymerase I activity (GO:0001054)	15	1	0.06	15.45	6.66E-02	1.00E+00	MF	2
proteasome binding (GO:0070628)	15	1	0.06	15.45	6.66E-02	1.00E+00	MF	2
phospholipid transfer activity (GO:0120014)	15	1	0.06	15.45	6.66E-02	1.00E+00	MF	2
tRNA (guanine) methyltransferase activity (GO:0016423)	16	1	0.07	14.48	7.06E-02	1.00E+00	MF	2
beta-fructofuranosidase activity (GO:0004564)	17	1	0.07	13.63	7.46E-02	1.00E+00	MF	2
diphosphotransferase activity (GO:0016778)	18	1	0.08	12.87	7.86E-02	1.00E+00	MF	2
catalytic activity, acting on a glycoprotein (GO:0140103)	18	1	0.08	12.87	7.86E-02	1.00E+00	MF	2
lipid transfer activity (GO:0120013)	19	1	0.08	12.19	8.25E-02	1.00E+00	MF	2
oxidoreductase activity, acting on the CH-OH group of donors, oxygen as acceptor (GO:0016899)	20	1	0.09	11.59	8.65E-02	1.00E+00	MF	2
phospholipid transporter activity (GO:0005548)	20	1	0.09	11.59	8.65E-02	1.00E+00	MF	2
acetylglucosaminyltransferase activity (GO:0008375)	24	1	0.1	9.65	1.02E-01	1.00E+00	MF	2
tRNA methyltransferase activity (GO:0008175)	25	1	0.11	9.27	1.06E-01	1.00E+00	MF	2
protein serine/threonine kinase activator activity (GO:0043539)	27	1	0.12	8.58	1.14E-01	1.00E+00	MF	2
ubiquitin-like protein conjugating enzyme binding (GO:0044390)	55	2	0.24	8.43	2.51E-02	1.00E+00	MF	2
sulfurtransferase activity (GO:0016783)	28	1	0.12	8.28	1.17E-01	1.00E+00	MF	2
polyubiquitin modification-dependent protein binding (GO:0031593)	33	1	0.14	7.02	1.36E-01	1.00E+00	MF	2
lactoperoxidase activity (GO:0140825)	145	4	0.63	6.39	4.00E-03	1.00E+00	MF	2
ATPase regulator activity (GO:0060590)	38	1	0.16	6.1	1.55E-01	1.00E+00	MF	2
modification-dependent protein binding (GO:0140030)	38	1	0.16	6.1	1.55E-01	1.00E+00	MF	2
UDP-glucosyltransferase activity (GO:0035251)	298	7	1.29	5.44	3.72E-04	4.69E-01	MF	2
transferase activity, transferring sulphur-containing groups (GO:0016782)	44	1	0.19	5.27	1.76E-01	1.00E+00	MF	2
protein kinase activator activity (GO:0030295)	45	1	0.19	5.15	1.80E-01	1.00E+00	MF	2
kinase activator activity (GO:0019209)	45	1	0.19	5.15	1.80E-01	1.00E+00	MF	2
quercetin 7-O-glucosyltransferase activity (GO:0080044)	47	1	0.2	4.93	1.87E-01	1.00E+00	MF	2
quercetin 3-O-glucosyltransferase activity (GO:0080043)	48	1	0.21	4.83	1.90E-01	1.00E+00	MF	2
glucosyltransferase activity (GO:0046527)	340	7	1.47	4.77	7.98E-04	5.02E-01	MF	2

GO Term	Genome	Count	Expected	Fold Enrichment	P-value	FDR	Term	Cluster
water transmembrane transporter activity (GO:0005372)	49	1	0.21	4.73	1.94E-01	1.00E+00	MF	2
tRNA binding (GO:0000049)	49	1	0.21	4.73	1.94E-01	1.00E+00	MF	2
water channel activity (GO:0015250)	49	1	0.21	4.73	1.94E-01	1.00E+00	MF	2
UDP-glycosyltransferase activity (GO:0008194)	428	8	1.85	4.33	6.33E-04	5.31E-01	MF	2
oxidoreductase activity, acting on peroxide as acceptor (GO:0016684)	224	4	0.97	4.14	1.72E-02	1.00E+00	MF	2
peroxidase activity (GO:0004601)	224	4	0.97	4.14	1.72E-02	1.00E+00	MF	2
xenobiotic transmembrane transporter activity (GO:0042910)	58	1	0.25	3.99	2.25E-01	1.00E+00	MF	2
myosin phosphatase activity (GO:0017018)	243	4	1.05	3.81	2.23E-02	1.00E+00	MF	2
O-methyltransferase activity (GO:0008171)	62	1	0.27	3.74	2.38E-01	1.00E+00	MF	2
protein serine/threonine phosphatase activity (GO:0004722)	249	4	1.07	3.72	2.41E-02	1.00E+00	MF	2
calcium-dependent protein kinase activity (GO:0010857)	64	1	0.28	3.62	2.44E-01	1.00E+00	MF	2
calmodulin-dependent protein kinase activity (GO:0004683)	64	1	0.28	3.62	2.44E-01	1.00E+00	MF	2
calcium-dependent protein serine/threonine kinase activity (GO:0009931)	64	1	0.28	3.62	2.44E-01	1.00E+00	MF	2
antioxidant activity (GO:0016209)	261	4	1.13	3.55	2.79E-02	1.00E+00	MF	2
molecular function activator activity (GO:0140677)	203	3	0.88	3.42	5.96E-02	1.00E+00	MF	2
phosphoric diester hydrolase activity (GO:0008081)	68	1	0.29	3.41	2.57E-01	1.00E+00	MF	2
ribosome binding (GO:0043022)	70	1	0.3	3.31	2.64E-01	1.00E+00	MF	2
translation elongation factor activity (GO:0003746)	72	1	0.31	3.22	2.70E-01	1.00E+00	MF	2
mRNA binding (GO:0003729)	651	9	2.81	3.2	2.33E-03	1.00E+00	MF	2
DNA-directed 5'-3' RNA polymerase activity (GO:0003899)	73	1	0.32	3.17	2.73E-01	1.00E+00	MF	2
phosphoprotein phosphatase activity (GO:0004721)	294	4	1.27	3.15	4.02E-02	1.00E+00	MF	2
RNA methyltransferase activity (GO:0008173)	78	1	0.34	2.97	2.89E-01	1.00E+00	MF	2
hexosyltransferase activity (GO:0016758)	637	8	2.75	2.91	7.02E-03	1.00E+00	MF	2
heme binding (GO:0020037)	565	7	2.44	2.87	1.22E-02	1.00E+00	MF	2
5'-3' RNA polymerase activity (GO:0034062)	84	1	0.36	2.76	3.07E-01	1.00E+00	MF	2
manganese ion binding (GO:0030145)	84	1	0.36	2.76	3.07E-01	1.00E+00	MF	2
RNA polymerase activity (GO:0097747)	84	1	0.36	2.76	3.07E-01	1.00E+00	MF	2
lipid transporter activity (GO:0005319)	86	1	0.37	2.69	3.13E-01	1.00E+00	MF	2
tetrapyrrole binding (GO:0046906)	609	7	2.63	2.66	1.76E-02	1.00E+00	MF	2
calmodulin binding (GO:0005516)	177	2	0.76	2.62	1.80E-01	1.00E+00	MF	2
FAD binding (GO:0071949)	89	1	0.38	2.6	3.22E-01	1.00E+00	MF	2
ubiquitin binding (GO:0043130)	89	1	0.38	2.6	3.22E-01	1.00E+00	MF	2
antiporter activity (GO:0015297)	181	2	0.78	2.56	1.86E-01	1.00E+00	MF	2
enzyme activator activity (GO:0008047)	184	2	0.79	2.52	1.91E-01	1.00E+00	MF	2
RNA helicase activity (GO:0003724)	93	1	0.4	2.49	3.33E-01	1.00E+00	MF	2
ATP-dependent activity, acting on RNA (GO:0008186)	93	1	0.4	2.49	3.33E-01	1.00E+00	MF	2
glucosidase activity (GO:0015926)	94	1	0.41	2.46	3.36E-01	1.00E+00	MF	2
catalytic activity, acting on a tRNA (GO:0140101)	191	2	0.82	2.43	2.02E-01	1.00E+00	MF	2
amino acid transmembrane transporter activity (GO:0015171)	96	1	0.41	2.41	3.42E-01	1.00E+00	MF	2
ribonucleoprotein complex binding (GO:0043021)	96	1	0.41	2.41	3.42E-01	1.00E+00	MF	2
ligase activity, forming carbon-oxygen bonds (GO:0016875)	101	1	0.44	2.29	3.56E-01	1.00E+00	MF	2
aminoacyl-tRNA ligase activity (GO:0004812)	101	1	0.44	2.29	3.56E-01	1.00E+00	MF	2
ubiquitin-like protein binding (GO:0032182)	102	1	0.44	2.27	3.59E-01	1.00E+00	MF	2
glycosyltransferase activity (GO:0016757)	972	9	4.2	2.15	4.04E-02	1.00E+00	MF	2
GTPase activator activity (GO:0005096)	110	1	0.47	2.11	3.80E-01	1.00E+00	MF	2
protein-folding chaperone binding (GO:0051087)	113	1	0.49	2.05	3.88E-01	1.00E+00	MF	2
flavin adenine dinucleotide binding (GO:0050660)	230	2	0.99	2.01	2.63E-01	1.00E+00	MF	2
phosphoric ester hydrolase activity (GO:0042578)	576	5	2.49	2.01	1.06E-01	1.00E+00	MF	2
microtubule binding (GO:0008017)	248	2	1.07	1.87	2.92E-01	1.00E+00	MF	2
vitamin B6 binding (GO:0070279)	124	1	0.54	1.87	4.17E-01	1.00E+00	MF	2
pyridoxal phosphate binding (GO:0030170)	124	1	0.54	1.87	4.17E-01	1.00E+00	MF	2

GO Term	Genome	Count	Expected	Fold Enrichment	P-value	FDR	Term	Cluster
phosphatase activity (GO:0016791)	505	4	2.18	1.84	1.77E-01	1.00E+00	MF	2
protein kinase regulator activity (GO:0019887)	127	1	0.55	1.82	4.24E-01	1.00E+00	MF	2
tubulin binding (GO:0015631)	259	2	1.12	1.79	3.09E-01	1.00E+00	MF	2
enzyme binding (GO:0019899)	271	2	1.17	1.71	3.28E-01	1.00E+00	MF	2
kinase regulator activity (GO:0019207)	137	1	0.59	1.69	4.49E-01	1.00E+00	MF	2
carboxylic acid transmembrane transporter activity (GO:0046943)	140	1	0.6	1.66	4.56E-01	1.00E+00	MF	2
organic acid transmembrane transporter activity (GO:0005342)	140	1	0.6	1.66	4.56E-01	1.00E+00	MF	2
GTPase activity (GO:0003924)	280	2	1.21	1.66	3.42E-01	1.00E+00	MF	2
monooxygenase activity (GO:0004497)	441	3	1.9	1.58	4.44E-01	1.00E+00	MF	2
catalytic activity, acting on RNA (GO:0140098)	595	4	2.57	1.56	3.32E-01	1.00E+00	MF	2
serine-type endopeptidase activity (GO:0004252)	152	1	0.66	1.52	4.83E-01	1.00E+00	MF	2
iron ion binding (GO:0005506)	465	3	2.01	1.49	4.58E-01	1.00E+00	MF	2
metal ion binding (GO:0046872)	3774	24	16.29	1.47	5.01E-02	1.00E+00	MF	2
cation binding (GO:0043169)	3802	24	16.41	1.46	6.68E-02	1.00E+00	MF	2
RNA binding (GO:0003723)	2087	13	9.01	1.44	1.69E-01	1.00E+00	MF	2
transition metal ion binding (GO:0046914)	1290	8	5.57	1.44	2.77E-01	1.00E+00	MF	2
molecular function regulator activity (GO:0098772)	652	4	2.81	1.42	3.70E-01	1.00E+00	MF	2
vitamin binding (GO:0019842)	165	1	0.71	1.4	5.11E-01	1.00E+00	MF	2
signaling receptor activity (GO:0038023)	171	1	0.74	1.35	5.24E-01	1.00E+00	MF	2
oxidoreductase activity, acting on paired donors, with incorporation or reduction of molecular oxygen (GO:0016705)	513	3	2.21	1.35	4.89E-01	1.00E+00	MF	2
secondary active transmembrane transporter activity (GO:0015291)	347	2	1.5	1.34	6.64E-01	1.00E+00	MF	2
hydrolase activity, acting on acid anhydrides (GO:0016817)	875	5	3.78	1.32	4.34E-01	1.00E+00	MF	2
DNA-binding transcription factor activity (GO:0003700)	1586	9	6.85	1.31	4.29E-01	1.00E+00	MF	2
ubiquitin protein ligase activity (GO:0061630)	354	2	1.53	1.31	6.68E-01	1.00E+00	MF	2
methyltransferase activity (GO:0008168)	532	3	2.3	1.31	5.03E-01	1.00E+00	MF	2
molecular transducer activity (GO:0060089)	179	1	0.77	1.29	5.40E-01	1.00E+00	MF	2
ubiquitin-like protein ligase activity (GO:0061659)	360	2	1.55	1.29	6.71E-01	1.00E+00	MF	2
transferase activity, transferring one-carbon groups (GO:0016741)	549	3	2.37	1.27	5.15E-01	1.00E+00	MF	2
organic anion transmembrane transporter activity (GO:0008514)	184	1	0.79	1.26	5.50E-01	1.00E+00	MF	2
ubiquitin-protein transferase activity (GO:0004842)	558	3	2.41	1.25	5.22E-01	1.00E+00	MF	2
guanyl ribonucleotide binding (GO:0032561)	375	2	1.62	1.24	6.79E-01	1.00E+00	MF	2
GTP binding (GO:0005525)	375	2	1.62	1.24	6.79E-01	1.00E+00	MF	2
guanyl nucleotide binding (GO:0019001)	375	2	1.62	1.24	6.79E-01	1.00E+00	MF	2
nucleoside-triphosphatase regulator activity (GO:0060589)	191	1	0.82	1.21	5.63E-01	1.00E+00	MF	2
GTPase regulator activity (GO:0030695)	191	1	0.82	1.21	5.63E-01	1.00E+00	MF	2
ubiquitin-like protein transferase activity (GO:0019787)	578	3	2.49	1.2	7.42E-01	1.00E+00	MF	2
aminoacyltransferase activity (GO:0016755)	583	3	2.52	1.19	7.43E-01	1.00E+00	MF	2
transcription regulator activity (GO:0140110)	1753	9	7.57	1.19	5.73E-01	1.00E+00	MF	2
sequence-specific DNA binding (GO:0043565)	1176	6	5.08	1.18	6.47E-01	1.00E+00	MF	2
transferase activity (GO:0016740)	5764	29	24.88	1.17	3.84E-01	1.00E+00	MF	2
enzyme regulator activity (GO:0030234)	604	3	2.61	1.15	7.48E-01	1.00E+00	MF	2
electron transfer activity (GO:0009055)	203	1	0.88	1.14	5.86E-01	1.00E+00	MF	2
oxidoreductase activity (GO:0016491)	2048	10	8.84	1.13	6.05E-01	1.00E+00	MF	2
ion binding (GO:0043167)	7841	38	33.84	1.12	4.41E-01	1.00E+00	MF	2
enzyme inhibitor activity (GO:0004857)	207	1	0.89	1.12	5.93E-01	1.00E+00	MF	2
molecular function inhibitor activity (GO:0140678)	209	1	0.9	1.11	5.96E-01	1.00E+00	MF	2
hydrolase activity, acting on ester bonds (GO:0016788)	1254	6	5.41	1.11	6.64E-01	1.00E+00	MF	2
pyrophosphatase activity (GO:0016462)	851	4	3.67	1.09	7.88E-01	1.00E+00	MF	2
protein-containing complex binding (GO:0044877)	431	2	1.86	1.08	7.10E-01	1.00E+00	MF	2
heterocyclic compound binding (GO:1901363)	10349	48	44.67	1.07	5.42E-01	1.00E+00	MF	2

GO Term	Genome	Count	Expected	Fold Enrichment	P-value	FDR	Term	Cluster
hydrolase activity, acting on acid anhydrides, in phosphorus-containing anhydrides (GO:0016818)	864	4	3.73	1.07	7.91E-01	1.00E+00	MF	2
organic cyclic compound binding (GO:0097159)	10373	48	44.77	1.07	6.01E-01	1.00E+00	MF	2
zinc ion binding (GO:0008270)	651	3	2.81	1.07	7.61E-01	1.00E+00	MF	2
nucleic acid binding (GO:0003676)	5685	26	24.54	1.06	7.43E-01	1.00E+00	MF	2
binding (GO:0005488)	14290	65	61.67	1.05	6.32E-01	1.00E+00	MF	2
cytoskeletal protein binding (GO:0008092)	440	2	1.9	1.05	7.16E-01	1.00E+00	MF	2
ubiquitin-like protein peptidase activity (GO:0019783)	220	1	0.95	1.05	6.15E-01	1.00E+00	MF	2
transferase activity, transferring alkyl or aryl (other than methyl) groups (GO:0016765)	222	1	0.96	1.04	6.18E-01	1.00E+00	MF	2
S-adenosylmethionine-dependent methyltransferase activity (GO:0008757)	222	1	0.96	1.04	6.18E-01	1.00E+00	MF	2
kinase activity (GO:0016301)	2226	10	9.61	1.04	8.67E-01	1.00E+00	MF	2
molecular_function (GO:0003674)	23060	101	99.53	1.01	8.76E-01	1.00E+00	MF	2
protein kinase activity (GO:0004672)	1604	7	6.92	1.01	8.47E-01	1.00E+00	MF	2
transferase activity, transferring phosphorus-containing groups (GO:0016772)	2537	11	10.95	1	1.00E+00	1.00E+00	MF	2
catalytic activity, acting on a protein (GO:0140096)	3926	17	16.94	1	1.00E+00	1.00E+00	MF	2
protein binding (GO:0005515)	2323	10	10.03	1	1.00E+00	1.00E+00	MF	2
catalytic activity (GO:0003824)	13387	57	57.78	0.99	9.35E-01	1.00E+00	MF	2
Unclassified (UNCLASSIFIED)	16329	69	70.47	0.98	8.76E-01	1.00E+00	MF	2
transporter activity (GO:0005215)	1678	7	7.24	0.97	1.00E+00	1.00E+00	MF	2
serine hydrolase activity (GO:0017171)	247	1	1.07	0.94	1.00E+00	1.00E+00	MF	2
serine-type peptidase activity (GO:0008236)	247	1	1.07	0.94	1.00E+00	1.00E+00	MF	2
ribonucleoside triphosphate phosphatase activity (GO:0017111)	755	3	3.26	0.92	1.00E+00	1.00E+00	MF	2
passive transmembrane transporter activity (GO:0022803)	252	1	1.09	0.92	1.00E+00	1.00E+00	MF	2
channel activity (GO:0015267)	252	1	1.09	0.92	1.00E+00	1.00E+00	MF	2
phosphotransferase activity, alcohol group as acceptor (GO:0016773)	1823	7	7.87	0.89	1.00E+00	1.00E+00	MF	2
translation regulator activity, nucleic acid binding (GO:0090079)	261	1	1.13	0.89	1.00E+00	1.00E+00	MF	2
translation factor activity, RNA binding (GO:0008135)	261	1	1.13	0.89	1.00E+00	1.00E+00	MF	2
ligase activity (GO:0016874)	529	2	2.28	0.88	1.00E+00	1.00E+00	MF	2
DNA binding (GO:0003677)	3182	12	13.73	0.87	7.77E-01	1.00E+00	MF	2
transmembrane transporter activity (GO:0022857)	1612	6	6.96	0.86	1.00E+00	1.00E+00	MF	2
small molecule binding (GO:0036094)	4599	17	19.85	0.86	5.51E-01	1.00E+00	MF	2
translation regulator activity (GO:0045182)	271	1	1.17	0.85	1.00E+00	1.00E+00	MF	2
RNA polymerase II cis-regulatory region sequence-specific DNA binding (GO:0000978)	273	1	1.18	0.85	1.00E+00	1.00E+00	MF	2
nucleotidyltransferase activity (GO:0016779)	279	1	1.2	0.83	1.00E+00	1.00E+00	MF	2
anion binding (GO:0043168)	4484	16	19.35	0.83	4.69E-01	1.00E+00	MF	2
carbohydrate derivative binding (GO:0097367)	3928	14	16.95	0.83	5.22E-01	1.00E+00	MF	2
hydrolase activity (GO:0016787)	4566	16	19.71	0.81	4.70E-01	1.00E+00	MF	2
purine ribonucleoside triphosphate binding (GO:0035639)	3719	13	16.05	0.81	5.11E-01	1.00E+00	MF	2
acyltransferase activity (GO:0016746)	1155	4	4.98	0.8	8.22E-01	1.00E+00	MF	2
nucleotide binding (GO:0000166)	4340	15	18.73	0.8	4.60E-01	1.00E+00	MF	2
nucleoside phosphate binding (GO:1901265)	4340	15	18.73	0.8	4.60E-01	1.00E+00	MF	2
oxidoreductase activity, acting on CH-OH group of donors (GO:0016614)	290	1	1.25	0.8	1.00E+00	1.00E+00	MF	2
purine ribonucleotide binding (GO:0032555)	3854	13	16.63	0.78	4.36E-01	1.00E+00	MF	2
cis-regulatory region sequence-specific DNA binding (GO:0000987)	299	1	1.29	0.77	1.00E+00	1.00E+00	MF	2
ribonucleotide binding (GO:0032553)	3896	13	16.81	0.77	3.69E-01	1.00E+00	MF	2
salt transmembrane transporter activity (GO:1901702)	300	1	1.29	0.77	1.00E+00	1.00E+00	MF	2
ATP binding (GO:0005524)	3362	11	14.51	0.76	4.09E-01	1.00E+00	MF	2
RNA polymerase II transcription regulatory region sequence-specific DNA binding (GO:0000977)	306	1	1.32	0.76	1.00E+00	1.00E+00	MF	2
purine nucleotide binding (GO:0017076)	4059	13	17.52	0.74	3.11E-01	1.00E+00	MF	2
adenyl ribonucleotide binding (GO:0032559)	3497	11	15.09	0.73	3.43E-01	1.00E+00	MF	2
active transmembrane transporter activity (GO:0022804)	645	2	2.78	0.72	1.00E+00	1.00E+00	MF	2

GO Term	Genome	Count	Expected	Fold Enrichment	P-value	FDR	Term	Cluster
catalytic activity, acting on a nucleic acid (GO:0140640)	1293	4	5.58	0.72	6.66E-01	1.00E+00	MF	2
protein dimerization activity (GO:0046983)	649	2	2.8	0.71	1.00E+00	1.00E+00	MF	2
adenyl nucleotide binding (GO:0030554)	3702	11	15.98	0.69	2.34E-01	1.00E+00	MF	2
DNA-binding transcription factor activity, RNA polymerase II-specific (GO:0000981)	351	1	1.51	0.66	1.00E+00	1.00E+00	MF	2
calcium ion binding (GO:0005509)	355	1	1.53	0.65	1.00E+00	1.00E+00	MF	2
cysteine-type peptidase activity (GO:0008234)	385	1	1.66	0.6	1.00E+00	1.00E+00	MF	2
hydrolase activity, hydrolyzing O-glycosyl compounds (GO:0004553)	462	1	1.99	0.5	7.27E-01	1.00E+00	MF	2
endopeptidase activity (GO:0004175)	467	1	2.02	0.5	7.27E-01	1.00E+00	MF	2
ATP hydrolysis activity (GO:0016887)	477	1	2.06	0.49	7.27E-01	1.00E+00	MF	2
peptidase activity (GO:0008233)	1007	2	4.35	0.46	3.34E-01	1.00E+00	MF	2
hydrolase activity, acting on glycosyl bonds (GO:0016798)	525	1	2.27	0.44	7.32E-01	1.00E+00	MF	2
acyltransferase activity, transferring groups other than amino-acyl groups (GO:0016747)	531	1	2.29	0.44	7.32E-01	1.00E+00	MF	2
helicase activity (GO:0004386)	541	1	2.33	0.43	7.34E-01	1.00E+00	MF	2
inorganic molecular entity transmembrane transporter activity (GO:0015318)	562	1	2.43	0.41	7.38E-01	1.00E+00	MF	2
lyase activity (GO:0016829)	572	1	2.47	0.41	5.26E-01	1.00E+00	MF	2
transcription regulatory region nucleic acid binding (GO:0001067)	589	1	2.54	0.39	5.26E-01	1.00E+00	MF	2
transcription cis-regulatory region binding (GO:0000976)	589	1	2.54	0.39	5.26E-01	1.00E+00	MF	2
sequence-specific double-stranded DNA binding (GO:1990837)	608	1	2.62	0.38	5.28E-01	1.00E+00	MF	2
ATP-dependent activity (GO:0140657)	1364	2	5.89	0.34	1.36E-01	1.00E+00	MF	2
double-stranded DNA binding (GO:0003690)	723	1	3.12	0.32	3.82E-01	1.00E+00	MF	2
protein serine/threonine kinase activity (GO:0004674)	923	1	3.98	0.25	1.95E-01	1.00E+00	MF	2
extrinsic component of endoplasmic reticulum membrane (GO:0042406)	2	1	0.01	> 100	1.28E-02	1.00E+00	CC	2
Dsl1/NZR complex (GO:0070939)	3	1	0.01	77.23	1.71E-02	1.00E+00	CC	2
mitochondrial degradosome (GO:0045025)	4	1	0.02	57.92	2.13E-02	1.00E+00	CC	2
phragmoplast (GO:0009524)	5	1	0.02	46.34	2.55E-02	1.00E+00	CC	2
cohesin complex (GO:0008278)	7	1	0.03	33.1	3.39E-02	1.00E+00	CC	2
Piccolo NuA4 histone acetyltransferase complex (GO:0032777)	9	1	0.04	25.74	4.22E-02	1.00E+00	CC	2
extrinsic component of organelle membrane (GO:0031312)	9	1	0.04	25.74	4.22E-02	1.00E+00	CC	2
HAUS complex (GO:0070652)	10	1	0.04	23.17	4.63E-02	1.00E+00	CC	2
mitochondrial nucleoid (GO:0042645)	10	1	0.04	23.17	4.63E-02	1.00E+00	CC	2
RNA polymerase I complex (GO:0005736)	17	1	0.07	13.63	7.46E-02	1.00E+00	CC	2
nuclear microtubule (GO:0005880)	21	1	0.09	11.03	9.04E-02	1.00E+00	CC	2
cytoplasmic exosome (RNase complex) (GO:0000177)	22	1	0.09	10.53	9.43E-02	1.00E+00	CC	2
spindle microtubule (GO:0005876)	22	1	0.09	10.53	9.43E-02	1.00E+00	CC	2
H4/H2A histone acetyltransferase complex (GO:0043189)	23	1	0.1	10.07	9.82E-02	1.00E+00	CC	2
NuA4 histone acetyltransferase complex (GO:0035267)	23	1	0.1	10.07	9.82E-02	1.00E+00	CC	2
plant-type cell wall (GO:0009505)	94	4	0.41	9.86	8.68E-04	7.56E-01	CC	2
cell wall (GO:0005618)	112	4	0.48	8.28	1.62E-03	7.06E-01	CC	2
H4 histone acetyltransferase complex (GO:1902562)	28	1	0.12	8.28	1.17E-01	1.00E+00	CC	2
external encapsulating structure (GO:0030312)	116	4	0.5	7.99	1.84E-03	5.33E-01	CC	2
nucleoid (GO:0009295)	31	1	0.13	7.47	1.29E-01	1.00E+00	CC	2
exosome (RNase complex) (GO:0000178)	34	1	0.15	6.81	1.40E-01	1.00E+00	CC	2
exoribonuclease complex (GO:1905354)	35	1	0.15	6.62	1.44E-01	1.00E+00	CC	2
plastid outer membrane (GO:0009527)	53	1	0.23	4.37	2.08E-01	1.00E+00	CC	2
histone acetyltransferase complex (GO:0000123)	60	1	0.26	3.86	2.31E-01	1.00E+00	CC	2
microtubule associated complex (GO:0005875)	66	1	0.28	3.51	2.51E-01	1.00E+00	CC	2
acetyltransferase complex (GO:1902493)	72	1	0.31	3.22	2.70E-01	1.00E+00	CC	2
protein acetyltransferase complex (GO:0031248)	72	1	0.31	3.22	2.70E-01	1.00E+00	CC	2
extrinsic component of membrane (GO:0019898)	77	1	0.33	3.01	2.86E-01	1.00E+00	CC	2
spindle (GO:0005819)	82	1	0.35	2.83	3.01E-01	1.00E+00	CC	2
plasmodesma (GO:0009506)	99	1	0.43	2.34	3.50E-01	1.00E+00	CC	2

GO Term	Genome	Count	Expected	Fold Enrichment	P-value	FDR	Term	Cluster
cell-cell junction (GO:0005911)	99	1	0.43	2.34	3.50E-01	1.00E+00	CC	2
symplast (GO:0055044)	99	1	0.43	2.34	3.50E-01	1.00E+00	CC	2
anchoring junction (GO:0070161)	99	1	0.43	2.34	3.50E-01	1.00E+00	CC	2
cell junction (GO:0030054)	99	1	0.43	2.34	3.50E-01	1.00E+00	CC	2
vesicle tethering complex (GO:0099023)	113	1	0.49	2.05	3.88E-01	1.00E+00	CC	2
microtubule (GO:0005874)	259	2	1.12	1.79	3.09E-01	1.00E+00	CC	2
endoplasmic reticulum protein-containing complex (GO:0140534)	132	1	0.57	1.76	4.37E-01	1.00E+00	CC	2
nuclear DNA-directed RNA polymerase complex (GO:0055029)	133	1	0.57	1.74	4.39E-01	1.00E+00	CC	2
polymeric cytoskeletal fiber (GO:0099513)	267	2	1.15	1.74	3.22E-01	1.00E+00	CC	2
supramolecular polymer (GO:0099081)	268	2	1.16	1.73	3.23E-01	1.00E+00	CC	2
supramolecular fiber (GO:0099512)	268	2	1.16	1.73	3.23E-01	1.00E+00	CC	2
extracellular region (GO:0005576)	698	5	3.01	1.66	2.34E-01	1.00E+00	CC	2
mitochondrial matrix (GO:0005759)	143	1	0.62	1.62	4.63E-01	1.00E+00	CC	2
outer membrane (GO:0019867)	288	2	1.24	1.61	3.55E-01	1.00E+00	CC	2
organelle outer membrane (GO:0031968)	288	2	1.24	1.61	3.55E-01	1.00E+00	CC	2
endoplasmic reticulum membrane (GO:0005789)	446	3	1.92	1.56	4.47E-01	1.00E+00	CC	2
endoplasmic reticulum subcompartment (GO:0098827)	449	3	1.94	1.55	4.49E-01	1.00E+00	CC	2
nuclear outer membrane-endoplasmic reticulum membrane network (GO:0042175)	452	3	1.95	1.54	4.50E-01	1.00E+00	CC	2
cell periphery (GO:0071944)	1669	11	7.2	1.53	1.76E-01	1.00E+00	CC	2
microtubule cytoskeleton (GO:0015630)	304	2	1.31	1.52	3.79E-01	1.00E+00	CC	2
endoplasmic reticulum (GO:0005783)	954	6	4.12	1.46	3.13E-01	1.00E+00	CC	2
plastid membrane (GO:0042170)	334	2	1.44	1.39	6.58E-01	1.00E+00	CC	2
apoplast (GO:0048046)	175	1	0.76	1.32	5.32E-01	1.00E+00	CC	2
chloroplast thylakoid membrane (GO:0009535)	178	1	0.77	1.3	5.38E-01	1.00E+00	CC	2
DNA-directed RNA polymerase complex (GO:0000428)	178	1	0.77	1.3	5.38E-01	1.00E+00	CC	2
plastid thylakoid membrane (GO:0055035)	178	1	0.77	1.3	5.38E-01	1.00E+00	CC	2
RNA polymerase complex (GO:0030880)	184	1	0.79	1.26	5.50E-01	1.00E+00	CC	2
organelle subcompartment (GO:0031984)	563	3	2.43	1.23	7.38E-01	1.00E+00	CC	2
supramolecular complex (GO:0099080)	387	2	1.67	1.2	6.85E-01	1.00E+00	CC	2
plastid envelope (GO:0009526)	390	2	1.68	1.19	6.87E-01	1.00E+00	CC	2
plasma membrane (GO:0005886)	1449	7	6.25	1.12	6.82E-01	1.00E+00	CC	2
Unclassified (UNCLASSIFIED)	19561	94	84.42	1.11	1.45E-01	1.00E+00	CC	2
membrane (GO:0016020)	8074	38	34.85	1.09	5.68E-01	1.00E+00	CC	2
chloroplast thylakoid (GO:0009534)	214	1	0.92	1.08	6.05E-01	1.00E+00	CC	2
plastid thylakoid (GO:0031976)	214	1	0.92	1.08	6.05E-01	1.00E+00	CC	2
thylakoid membrane (GO:0042651)	221	1	0.95	1.05	6.17E-01	1.00E+00	CC	2
mitochondrion (GO:0005739)	1131	5	4.88	1.02	8.19E-01	1.00E+00	CC	2
ribosome (GO:0005840)	694	3	3	1	1.00E+00	1.00E+00	CC	2
cytoskeleton (GO:0005856)	478	2	2.06	0.97	1.00E+00	1.00E+00	CC	2
photosynthetic membrane (GO:0034357)	241	1	1.04	0.96	1.00E+00	1.00E+00	CC	2
mitochondrial protein-containing complex (GO:0098798)	242	1	1.04	0.96	1.00E+00	1.00E+00	CC	2
chromatin (GO:0000785)	246	1	1.06	0.94	1.00E+00	1.00E+00	CC	2
cellular_component (GO:0005575)	19828	76	85.58	0.89	1.45E-01	1.00E+00	CC	2
intracellular non-membrane-bounded organelle (GO:0043232)	2611	10	11.27	0.89	8.77E-01	1.00E+00	CC	2
non-membrane-bounded organelle (GO:0043228)	2611	10	11.27	0.89	8.77E-01	1.00E+00	CC	2
cellular anatomical entity (GO:0110165)	19592	75	84.56	0.89	1.45E-01	1.00E+00	CC	2
endomembrane system (GO:0012505)	2115	8	9.13	0.88	8.64E-01	1.00E+00	CC	2
cytosol (GO:0005829)	1383	5	5.97	0.84	8.36E-01	1.00E+00	CC	2
nucleus (GO:0005634)	6118	22	26.4	0.83	3.96E-01	1.00E+00	CC	2
chromosome (GO:0005694)	570	2	2.46	0.81	1.00E+00	1.00E+00	CC	2
intracellular membrane-bounded organelle (GO:0043231)	10826	36	46.72	0.77	7.04E-02	1.00E+00	CC	2

GO Term	Genome	Count	Expected	Fold Enrichment	P-value	FDR	Term	Cluster
membrane-bounded organelle (GO:0043227)	10848	36	46.82	0.77	7.04E-02	1.00E+00	CC	2
intracellular organelle (GO:0043229)	11979	39	51.7	0.75	3.65E-02	1.00E+00	CC	2
organelle (GO:0043226)	11985	39	51.73	0.75	3.65E-02	1.00E+00	CC	2
nucleoplasm (GO:0005654)	326	1	1.41	0.71	1.00E+00	1.00E+00	CC	2
intracellular anatomical structure (GO:0005622)	14114	42	60.91	0.69	2.25E-03	4.90E-01	CC	2
nucleolus (GO:0005730)	338	1	1.46	0.69	1.00E+00	1.00E+00	CC	2
intracellular organelle lumen (GO:0070013)	1025	3	4.42	0.68	8.06E-01	1.00E+00	CC	2
membrane-enclosed lumen (GO:0031974)	1025	3	4.42	0.68	8.06E-01	1.00E+00	CC	2
organelle lumen (GO:0043233)	1025	3	4.42	0.68	8.06E-01	1.00E+00	CC	2
organelle membrane (GO:0031090)	1851	5	7.99	0.63	3.63E-01	1.00E+00	CC	2
ribonucleoprotein complex (GO:1990904)	1113	3	4.8	0.62	6.38E-01	1.00E+00	CC	2
transferase complex, transferring phosphorus-containing groups (GO:0061695)	372	1	1.61	0.62	1.00E+00	1.00E+00	CC	2
protein-DNA complex (GO:0032993)	378	1	1.63	0.61	1.00E+00	1.00E+00	CC	2
transferase complex (GO:1990234)	772	2	3.33	0.6	7.77E-01	1.00E+00	CC	2
cytoplasm (GO:0005737)	8535	22	36.84	0.6	4.92E-03	8.56E-01	CC	2
intracellular protein-containing complex (GO:0140535)	792	2	3.42	0.59	7.79E-01	1.00E+00	CC	2
nuclear lumen (GO:0031981)	823	2	3.55	0.56	5.91E-01	1.00E+00	CC	2
thylakoid (GO:0009579)	417	1	1.8	0.56	1.00E+00	1.00E+00	CC	2
envelope (GO:0031975)	844	2	3.64	0.55	5.92E-01	1.00E+00	CC	2
organelle envelope (GO:0031967)	844	2	3.64	0.55	5.92E-01	1.00E+00	CC	2
protein-containing complex (GO:0032991)	4069	9	17.56	0.51	3.07E-02	1.00E+00	CC	2
Golgi apparatus (GO:0005794)	959	2	4.14	0.48	4.49E-01	1.00E+00	CC	2
catalytic complex (GO:1902494)	1505	3	6.5	0.46	2.24E-01	1.00E+00	CC	2
bounding membrane of organelle (GO:0098588)	1033	2	4.46	0.45	3.34E-01	1.00E+00	CC	2
plastid (GO:0009536)	1326	2	5.72	0.35	1.34E-01	1.00E+00	CC	2
nuclear protein-containing complex (GO:0140513)	991	1	4.28	0.23	1.37E-01	1.00E+00	CC	2
chloroplast (GO:0009507)	1128	1	4.87	0.21	9.91E-02	1.00E+00	CC	2
non-photoreactive DNA repair (GO:0010213)	1	1	0	> 100	5.36E-03	1.00E+00	BP	3
protein O-linked glycosylation via hydroxyproline (GO:0018258)	1	1	0	> 100	5.36E-03	1.00E+00	BP	3
cholesterol transport (GO:0030301)	2	1	0.01	> 100	8.03E-03	1.00E+00	BP	3
intracellular cholesterol transport (GO:0032367)	2	1	0.01	> 100	8.03E-03	1.00E+00	BP	3
arabinogalactan protein metabolic process (GO:0010405)	2	1	0.01	> 100	8.03E-03	1.00E+00	BP	3
cell wall proteoglycan metabolic process (GO:0010384)	3	1	0.01	> 100	1.07E-02	1.00E+00	BP	3
nucleotide-excision repair, preincision complex assembly (GO:0006294)	3	1	0.01	> 100	1.07E-02	1.00E+00	BP	3
positive regulation of seed germination (GO:0010030)	3	1	0.01	> 100	1.07E-02	1.00E+00	BP	3
histone H4-R3 methylation (GO:0043985)	3	1	0.01	> 100	1.07E-02	1.00E+00	BP	3
histone H4-K16 acetylation (GO:0043984)	3	1	0.01	> 100	1.07E-02	1.00E+00	BP	3
histone H4-K8 acetylation (GO:0043982)	3	1	0.01	> 100	1.07E-02	1.00E+00	BP	3
histone H4-K5 acetylation (GO:0043981)	3	1	0.01	> 100	1.07E-02	1.00E+00	BP	3
cell wall hydroxyproline-rich glycoprotein metabolic process (GO:0010404)	3	1	0.01	> 100	1.07E-02	1.00E+00	BP	3
response to gamma radiation (GO:0010332)	4	1	0.01	92.9	1.33E-02	1.00E+00	BP	3
primary root development (GO:0080022)	4	1	0.01	92.9	1.33E-02	1.00E+00	BP	3
UV-damage excision repair (GO:0070914)	5	1	0.01	74.32	1.60E-02	1.00E+00	BP	3
proteoglycan metabolic process (GO:0006029)	5	1	0.01	74.32	1.60E-02	1.00E+00	BP	3
gamma-aminobutyric acid transport (GO:0015812)	6	1	0.02	61.93	1.86E-02	1.00E+00	BP	3
mitochondrial ADP transmembrane transport (GO:0140021)	6	1	0.02	61.93	1.86E-02	1.00E+00	BP	3
protein O-linked glycosylation (GO:0006493)	6	1	0.02	61.93	1.86E-02	1.00E+00	BP	3
mitochondrial ATP transmembrane transport (GO:1990544)	6	1	0.02	61.93	1.86E-02	1.00E+00	BP	3
regulation of brassinosteroid mediated signaling pathway (GO:1900457)	6	1	0.02	61.93	1.86E-02	1.00E+00	BP	3
intracellular zinc ion homeostasis (GO:0006882)	6	1	0.02	61.93	1.86E-02	1.00E+00	BP	3
intracellular sterol transport (GO:0032366)	6	1	0.02	61.93	1.86E-02	1.00E+00	BP	3

GO Term	Genome	Count	Expected	Fold Enrichment	P-value	FDR	Term	Cluster
histone arginine methylation (GO:0034969)	7	1	0.02	53.08	2.13E-02	1.00E+00	BP	3
mucilage biosynthetic process involved in seed coat development (GO:0048354)	7	1	0.02	53.08	2.13E-02	1.00E+00	BP	3
histone H4 acetylation (GO:0043967)	7	1	0.02	53.08	2.13E-02	1.00E+00	BP	3
positive regulation of cell population proliferation (GO:0008284)	7	1	0.02	53.08	2.13E-02	1.00E+00	BP	3
regulation of seedling development (GO:1900140)	14	2	0.04	53.08	8.35E-04	1.00E+00	BP	3
mucilage metabolic process involved in seed coat development (GO:0048359)	8	1	0.02	46.45	2.39E-02	1.00E+00	BP	3
protein insertion into ER membrane (GO:0045048)	8	1	0.02	46.45	2.39E-02	1.00E+00	BP	3
mucilage biosynthetic process (GO:0010192)	8	1	0.02	46.45	2.39E-02	1.00E+00	BP	3
mucilage metabolic process (GO:0010191)	8	1	0.02	46.45	2.39E-02	1.00E+00	BP	3
intracellular lipid transport (GO:0032365)	8	1	0.02	46.45	2.39E-02	1.00E+00	BP	3
seed coat development (GO:0010214)	10	1	0.03	37.16	2.91E-02	1.00E+00	BP	3
response to ionizing radiation (GO:0010212)	10	1	0.03	37.16	2.91E-02	1.00E+00	BP	3
cellular response to UV (GO:0034644)	10	1	0.03	37.16	2.91E-02	1.00E+00	BP	3
root hair cell development (GO:0080147)	11	1	0.03	33.78	3.17E-02	1.00E+00	BP	3
cell maturation (GO:0048469)	11	1	0.03	33.78	3.17E-02	1.00E+00	BP	3
trichoblast differentiation (GO:0010054)	11	1	0.03	33.78	3.17E-02	1.00E+00	BP	3
root epidermal cell differentiation (GO:0010053)	11	1	0.03	33.78	3.17E-02	1.00E+00	BP	3
root hair cell differentiation (GO:0048765)	11	1	0.03	33.78	3.17E-02	1.00E+00	BP	3
trichoblast maturation (GO:0048764)	11	1	0.03	33.78	3.17E-02	1.00E+00	BP	3
centromere complex assembly (GO:0034508)	11	1	0.03	33.78	3.17E-02	1.00E+00	BP	3
response to UV-B (GO:0010224)	12	1	0.03	30.97	3.43E-02	1.00E+00	BP	3
cell fate determination (GO:0001709)	12	1	0.03	30.97	3.43E-02	1.00E+00	BP	3
leaf vascular tissue pattern formation (GO:0010305)	13	1	0.03	28.58	3.69E-02	1.00E+00	BP	3
nucleotide transmembrane transport (GO:1901679)	13	1	0.03	28.58	3.69E-02	1.00E+00	BP	3
regulation of seed germination (GO:0010029)	13	1	0.03	28.58	3.69E-02	1.00E+00	BP	3
CDP-choline pathway (GO:0006657)	13	1	0.03	28.58	3.69E-02	1.00E+00	BP	3
response to red light (GO:0010114)	15	1	0.04	24.77	4.21E-02	1.00E+00	BP	3
chloroplast RNA processing (GO:0031425)	15	1	0.04	24.77	4.21E-02	1.00E+00	BP	3
protoporphyrinogen IX biosynthetic process (GO:0006782)	15	1	0.04	24.77	4.21E-02	1.00E+00	BP	3
protoporphyrinogen IX metabolic process (GO:0046501)	15	1	0.04	24.77	4.21E-02	1.00E+00	BP	3
mitotic recombination (GO:0006312)	16	1	0.04	23.22	4.47E-02	1.00E+00	BP	3
peptidyl-arginine methylation (GO:0018216)	16	1	0.04	23.22	4.47E-02	1.00E+00	BP	3
xylem and phloem pattern formation (GO:0010051)	16	1	0.04	23.22	4.47E-02	1.00E+00	BP	3
ADP transport (GO:0015866)	17	1	0.05	21.86	4.72E-02	1.00E+00	BP	3
regulation of photomorphogenesis (GO:0010099)	17	1	0.05	21.86	4.72E-02	1.00E+00	BP	3
peptidyl-arginine modification (GO:0018195)	17	1	0.05	21.86	4.72E-02	1.00E+00	BP	3
establishment or maintenance of cell polarity (GO:0007163)	17	1	0.05	21.86	4.72E-02	1.00E+00	BP	3
ATP transport (GO:0015867)	18	1	0.05	20.64	4.98E-02	1.00E+00	BP	3
regulation of response to red or far red light (GO:2000030)	18	1	0.05	20.64	4.98E-02	1.00E+00	BP	3
phosphatidylcholine biosynthetic process (GO:0006656)	19	1	0.05	19.56	5.23E-02	1.00E+00	BP	3
chloroplast accumulation movement (GO:0009904)	19	1	0.05	19.56	5.23E-02	1.00E+00	BP	3
chloroplast avoidance movement (GO:0009903)	19	1	0.05	19.56	5.23E-02	1.00E+00	BP	3
regulation of abscisic acid-activated signaling pathway (GO:0009787)	20	1	0.05	18.58	5.49E-02	1.00E+00	BP	3
developmental maturation (GO:0021700)	20	1	0.05	18.58	5.49E-02	1.00E+00	BP	3
establishment of plastid localization (GO:0051667)	20	1	0.05	18.58	5.49E-02	1.00E+00	BP	3
regulation of cellular response to alcohol (GO:1905957)	20	1	0.05	18.58	5.49E-02	1.00E+00	BP	3
phloem or xylem histogenesis (GO:0010087)	20	1	0.05	18.58	5.49E-02	1.00E+00	BP	3
regulation of response to alcohol (GO:1901419)	20	1	0.05	18.58	5.49E-02	1.00E+00	BP	3
anatomical structure maturation (GO:0071695)	20	1	0.05	18.58	5.49E-02	1.00E+00	BP	3
anther development (GO:0048653)	20	1	0.05	18.58	5.49E-02	1.00E+00	BP	3
phosphatidylethanolamine biosynthetic process (GO:0006646)	20	1	0.05	18.58	5.49E-02	1.00E+00	BP	3

GO Term	Genome	Count	Expected	Fold Enrichment	P-value	FDR	Term	Cluster
chloroplast relocation (GO:0009902)	20	1	0.05	18.58	5.49E-02	1.00E+00	BP	3
zinc ion transmembrane transport (GO:0071577)	20	1	0.05	18.58	5.49E-02	1.00E+00	BP	3
gibberellin mediated signaling pathway (GO:0010476)	20	1	0.05	18.58	5.49E-02	1.00E+00	BP	3
phosphatidylethanolamine metabolic process (GO:0046337)	20	1	0.05	18.58	5.49E-02	1.00E+00	BP	3
androecium development (GO:0048466)	22	1	0.06	16.89	6.00E-02	1.00E+00	BP	3
stamen development (GO:0048443)	22	1	0.06	16.89	6.00E-02	1.00E+00	BP	3
cellular response to gibberellin stimulus (GO:0071370)	22	1	0.06	16.89	6.00E-02	1.00E+00	BP	3
oxylipin biosynthetic process (GO:0031408)	22	1	0.06	16.89	6.00E-02	1.00E+00	BP	3
oxylipin metabolic process (GO:0031407)	22	1	0.06	16.89	6.00E-02	1.00E+00	BP	3
zinc ion transport (GO:0006829)	22	1	0.06	16.89	6.00E-02	1.00E+00	BP	3
chloroplast localization (GO:0019750)	22	1	0.06	16.89	6.00E-02	1.00E+00	BP	3
systemic acquired resistance (GO:0009627)	23	1	0.06	16.16	6.25E-02	1.00E+00	BP	3
abaxial cell fate specification (GO:0010158)	23	1	0.06	16.16	6.25E-02	1.00E+00	BP	3
plastid localization (GO:0051644)	23	1	0.06	16.16	6.25E-02	1.00E+00	BP	3
regulation of cell shape (GO:0008360)	23	1	0.06	16.16	6.25E-02	1.00E+00	BP	3
positive regulation of post-embryonic development (GO:0048582)	23	1	0.06	16.16	6.25E-02	1.00E+00	BP	3
purine ribonucleotide transport (GO:0015868)	24	1	0.06	15.48	6.50E-02	1.00E+00	BP	3
heme biosynthetic process (GO:0006783)	26	1	0.07	14.29	7.00E-02	1.00E+00	BP	3
sterol transport (GO:0015918)	26	1	0.07	14.29	7.00E-02	1.00E+00	BP	3
purine nucleotide transport (GO:0015865)	27	1	0.07	13.76	7.25E-02	1.00E+00	BP	3
adenine nucleotide transport (GO:0051503)	27	1	0.07	13.76	7.25E-02	1.00E+00	BP	3
regulation of photoperiodism, flowering (GO:2000028)	29	1	0.08	12.81	7.75E-02	1.00E+00	BP	3
nucleotide transport (GO:0006862)	30	1	0.08	12.39	8.00E-02	1.00E+00	BP	3
regulation of cell population proliferation (GO:0042127)	30	1	0.08	12.39	8.00E-02	1.00E+00	BP	3
cell fate specification (GO:0001708)	30	1	0.08	12.39	8.00E-02	1.00E+00	BP	3
cortical cytoskeleton organization (GO:0030865)	31	1	0.08	11.99	8.24E-02	1.00E+00	BP	3
response to UV (GO:0009411)	31	1	0.08	11.99	8.24E-02	1.00E+00	BP	3
phosphatidylcholine metabolic process (GO:0046470)	31	1	0.08	11.99	8.24E-02	1.00E+00	BP	3
organic hydroxy compound transport (GO:0015850)	32	1	0.09	11.61	8.49E-02	1.00E+00	BP	3
purine-containing compound transmembrane transport (GO:0072530)	32	1	0.09	11.61	8.49E-02	1.00E+00	BP	3
cell fate commitment (GO:0045165)	64	2	0.17	11.61	1.37E-02	1.00E+00	BP	3
plant epidermal cell differentiation (GO:0090627)	32	1	0.09	11.61	8.49E-02	1.00E+00	BP	3
positive regulation of multicellular organismal process (GO:0051240)	33	1	0.09	11.26	8.74E-02	1.00E+00	BP	3
actin nucleation (GO:0045010)	34	1	0.09	10.93	8.98E-02	1.00E+00	BP	3
regulation of cell morphogenesis (GO:0022604)	34	1	0.09	10.93	8.98E-02	1.00E+00	BP	3
response to oomycetes (GO:0002239)	34	1	0.09	10.93	8.98E-02	1.00E+00	BP	3
defense response to oomycetes (GO:0002229)	34	1	0.09	10.93	8.98E-02	1.00E+00	BP	3
xylan acetylation (GO:1990937)	69	2	0.19	10.77	1.57E-02	1.00E+00	BP	3
floral whorl development (GO:0048438)	35	1	0.09	10.62	9.23E-02	1.00E+00	BP	3
regionalization (GO:0003002)	35	1	0.09	10.62	9.23E-02	1.00E+00	BP	3
protein insertion into membrane (GO:0051205)	36	1	0.1	10.32	9.47E-02	1.00E+00	BP	3
epigenetic regulation of gene expression (GO:0040029)	72	2	0.19	10.32	1.70E-02	1.00E+00	BP	3
heme metabolic process (GO:0042168)	36	1	0.1	10.32	9.47E-02	1.00E+00	BP	3
cellular response to light stimulus (GO:0071482)	37	1	0.1	10.04	9.71E-02	1.00E+00	BP	3
positive regulation of developmental process (GO:0051094)	37	1	0.1	10.04	9.71E-02	1.00E+00	BP	3
internal protein amino acid acetylation (GO:0006475)	38	1	0.1	9.78	9.96E-02	1.00E+00	BP	3
internal peptidyl-lysine acetylation (GO:0018393)	38	1	0.1	9.78	9.96E-02	1.00E+00	BP	3
histone acetylation (GO:0016573)	38	1	0.1	9.78	9.96E-02	1.00E+00	BP	3
cellular response to radiation (GO:0071478)	41	1	0.11	9.06	1.07E-01	1.00E+00	BP	3
floral organ development (GO:0048437)	41	1	0.11	9.06	1.07E-01	1.00E+00	BP	3
root morphogenesis (GO:0010015)	41	1	0.11	9.06	1.07E-01	1.00E+00	BP	3

GO Term	Genome	Count	Expected	Fold Enrichment	P-value	FDR	Term	Cluster
xenobiotic export from cell (GO:0046618)	42	1	0.11	8.85	1.09E-01	1.00E+00	BP	3
small GTPase mediated signal transduction (GO:0007264)	42	1	0.11	8.85	1.09E-01	1.00E+00	BP	3
xenobiotic detoxification by transmembrane export across the plasma membrane (GO:1990961)	42	1	0.11	8.85	1.09E-01	1.00E+00	BP	3
cell development (GO:0048468)	43	1	0.12	8.64	1.12E-01	1.00E+00	BP	3
peptidyl-lysine acetylation (GO:0018394)	43	1	0.12	8.64	1.12E-01	1.00E+00	BP	3
endoplasmic reticulum organization (GO:0007029)	43	1	0.12	8.64	1.12E-01	1.00E+00	BP	3
response to gibberellin (GO:0009739)	47	1	0.13	7.91	1.21E-01	1.00E+00	BP	3
amino acid transmembrane transport (GO:0003333)	97	2	0.26	7.66	2.92E-02	1.00E+00	BP	3
root development (GO:0048364)	100	2	0.27	7.43	3.09E-02	1.00E+00	BP	3
root system development (GO:0022622)	100	2	0.27	7.43	3.09E-02	1.00E+00	BP	3
pattern specification process (GO:0007389)	51	1	0.14	7.29	1.31E-01	1.00E+00	BP	3
cellular response to environmental stimulus (GO:0104004)	52	1	0.14	7.15	1.33E-01	1.00E+00	BP	3
protein acetylation (GO:0006473)	52	1	0.14	7.15	1.33E-01	1.00E+00	BP	3
cellular response to abiotic stimulus (GO:0071214)	52	1	0.14	7.15	1.33E-01	1.00E+00	BP	3
amino acid transport (GO:0006865)	105	2	0.28	7.08	3.37E-02	1.00E+00	BP	3
regulation of multicellular organismal development (GO:2000026)	106	2	0.29	7.01	3.43E-02	1.00E+00	BP	3
regulation of actin cytoskeleton organization (GO:0032956)	53	1	0.14	7.01	1.35E-01	1.00E+00	BP	3
regulation of post-embryonic development (GO:0048580)	106	2	0.29	7.01	3.43E-02	1.00E+00	BP	3
porphyrin-containing compound biosynthetic process (GO:0006779)	54	1	0.15	6.88	1.37E-01	1.00E+00	BP	3
regulation of actin filament-based process (GO:0032970)	54	1	0.15	6.88	1.37E-01	1.00E+00	BP	3
protein-DNA complex assembly (GO:0065004)	109	2	0.29	6.82	3.60E-02	1.00E+00	BP	3
negative regulation of gene expression, epigenetic (GO:0045814)	55	1	0.15	6.76	1.40E-01	1.00E+00	BP	3
histone methylation (GO:0016571)	55	1	0.15	6.76	1.40E-01	1.00E+00	BP	3
tetrapyrrole biosynthetic process (GO:0033014)	56	1	0.15	6.64	1.42E-01	1.00E+00	BP	3
plant epidermis development (GO:0090558)	58	1	0.16	6.41	1.47E-01	1.00E+00	BP	3
xenobiotic transport (GO:0042908)	58	1	0.16	6.41	1.47E-01	1.00E+00	BP	3
actin filament organization (GO:0007015)	116	2	0.31	6.41	4.03E-02	1.00E+00	BP	3
peptidyl-threonine dephosphorylation (GO:0035970)	61	1	0.16	6.09	1.54E-01	1.00E+00	BP	3
response to red or far red light (GO:0009639)	62	1	0.17	5.99	1.56E-01	1.00E+00	BP	3
organic acid transmembrane transport (GO:1903825)	127	2	0.34	5.85	4.73E-02	1.00E+00	BP	3
carboxylic acid transmembrane transport (GO:1905039)	127	2	0.34	5.85	4.73E-02	1.00E+00	BP	3
actin cytoskeleton organization (GO:0030036)	128	2	0.34	5.81	4.79E-02	1.00E+00	BP	3
tissue development (GO:0009888)	129	2	0.35	5.76	4.86E-02	1.00E+00	BP	3
actin filament-based process (GO:0030029)	130	2	0.35	5.72	4.92E-02	1.00E+00	BP	3
xylan metabolic process (GO:0045491)	131	2	0.35	5.67	4.99E-02	1.00E+00	BP	3
export across plasma membrane (GO:0140115)	67	1	0.18	5.55	1.67E-01	1.00E+00	BP	3
plant organ morphogenesis (GO:1905392)	68	1	0.18	5.46	1.69E-01	1.00E+00	BP	3
peptidyl-proline modification (GO:0018208)	68	1	0.18	5.46	1.69E-01	1.00E+00	BP	3
histone modification (GO:0016570)	143	2	0.38	5.2	5.82E-02	1.00E+00	BP	3
regulation of anatomical structure morphogenesis (GO:0022603)	73	1	0.2	5.09	1.80E-01	1.00E+00	BP	3
defense response to bacterium (GO:0042742)	73	1	0.2	5.09	1.80E-01	1.00E+00	BP	3
cell differentiation (GO:0030154)	223	3	0.6	5	2.32E-02	1.00E+00	BP	3
regulation of multicellular organismal process (GO:0051239)	150	2	0.4	4.95	6.32E-02	1.00E+00	BP	3
pigment biosynthetic process (GO:0046148)	77	1	0.21	4.83	1.89E-01	1.00E+00	BP	3
porphyrin-containing compound metabolic process (GO:0006778)	77	1	0.21	4.83	1.89E-01	1.00E+00	BP	3
plant organ development (GO:0099402)	231	3	0.62	4.83	2.54E-02	1.00E+00	BP	3
tetrapyrrole metabolic process (GO:0033013)	78	1	0.21	4.76	1.91E-01	1.00E+00	BP	3
regulation of cytoskeleton organization (GO:0051493)	78	1	0.21	4.76	1.91E-01	1.00E+00	BP	3
cellular developmental process (GO:0048869)	240	3	0.65	4.64	2.79E-02	1.00E+00	BP	3
flower development (GO:0009908)	81	1	0.22	4.59	1.98E-01	1.00E+00	BP	3
regulation of developmental process (GO:0050793)	245	3	0.66	4.55	2.94E-02	1.00E+00	BP	3

GO Term	Genome	Count	Expected	Fold Enrichment	P-value	FDR	Term	Cluster
response to bacterium (GO:0009617)	82	1	0.22	4.53	2.00E-01	1.00E+00	BP	3
supramolecular fiber organization (GO:0097435)	168	2	0.45	4.42	7.67E-02	1.00E+00	BP	3
membrane fusion (GO:0061025)	85	1	0.23	4.37	2.07E-01	1.00E+00	BP	3
protein methylation (GO:0006479)	85	1	0.23	4.37	2.07E-01	1.00E+00	BP	3
cell surface receptor signaling pathway (GO:0007166)	85	1	0.23	4.37	2.07E-01	1.00E+00	BP	3
protein alkylation (GO:0008213)	85	1	0.23	4.37	2.07E-01	1.00E+00	BP	3
recombinational repair (GO:0000725)	171	2	0.46	4.35	7.90E-02	1.00E+00	BP	3
reproductive shoot system development (GO:0090567)	86	1	0.23	4.32	2.09E-01	1.00E+00	BP	3
SCF-dependent proteasomal ubiquitin-dependent protein catabolic process (GO:0031146)	86	1	0.23	4.32	2.09E-01	1.00E+00	BP	3
carboxylic acid transport (GO:0046942)	173	2	0.47	4.3	8.06E-02	1.00E+00	BP	3
organic acid transport (GO:0015849)	173	2	0.47	4.3	8.06E-02	1.00E+00	BP	3
potassium ion transmembrane transport (GO:0071805)	87	1	0.23	4.27	2.11E-01	1.00E+00	BP	3
potassium ion transport (GO:0006813)	89	1	0.24	4.18	2.15E-01	1.00E+00	BP	3
transition metal ion transport (GO:0000041)	89	1	0.24	4.18	2.15E-01	1.00E+00	BP	3
cell wall macromolecule metabolic process (GO:0044036)	274	3	0.74	4.07	3.88E-02	1.00E+00	BP	3
protein autophosphorylation (GO:0046777)	94	1	0.25	3.95	2.26E-01	1.00E+00	BP	3
protein acylation (GO:0043543)	96	1	0.26	3.87	2.30E-01	1.00E+00	BP	3
pigment metabolic process (GO:0042440)	96	1	0.26	3.87	2.30E-01	1.00E+00	BP	3
establishment of organelle localization (GO:0051656)	96	1	0.26	3.87	2.30E-01	1.00E+00	BP	3
organic anion transport (GO:0015711)	300	3	0.81	3.72	4.84E-02	1.00E+00	BP	3
phyllome development (GO:0048827)	100	1	0.27	3.72	2.38E-01	1.00E+00	BP	3
carbohydrate derivative transport (GO:1901264)	101	1	0.27	3.68	2.40E-01	1.00E+00	BP	3
protein polyubiquitination (GO:0000209)	108	1	0.29	3.44	2.54E-01	1.00E+00	BP	3
regulation of reproductive process (GO:2000241)	113	1	0.3	3.29	2.64E-01	1.00E+00	BP	3
nucleotide-excision repair (GO:0006289)	117	1	0.31	3.18	2.72E-01	1.00E+00	BP	3
chloroplast organization (GO:0009658)	120	1	0.32	3.1	2.78E-01	1.00E+00	BP	3
response to light stimulus (GO:0009416)	240	2	0.65	3.1	1.38E-01	1.00E+00	BP	3
hemicellulose metabolic process (GO:0010410)	240	2	0.65	3.1	1.38E-01	1.00E+00	BP	3
protein-DNA complex organization (GO:0071824)	481	4	1.29	3.09	4.20E-02	1.00E+00	BP	3
inorganic ion homeostasis (GO:0098771)	121	1	0.33	3.07	2.80E-01	1.00E+00	BP	3
endomembrane system organization (GO:0010256)	123	1	0.33	3.02	2.84E-01	1.00E+00	BP	3
seed development (GO:0048316)	123	1	0.33	3.02	2.84E-01	1.00E+00	BP	3
cell wall polysaccharide metabolic process (GO:0010383)	250	2	0.67	2.97	1.47E-01	1.00E+00	BP	3
intracellular monoatomic cation homeostasis (GO:0030003)	129	1	0.35	2.88	2.95E-01	1.00E+00	BP	3
response to radiation (GO:0009314)	259	2	0.7	2.87	1.55E-01	1.00E+00	BP	3
fruit development (GO:0010154)	131	1	0.35	2.84	2.99E-01	1.00E+00	BP	3
reproductive structure development (GO:0048608)	270	2	0.73	2.75	1.66E-01	1.00E+00	BP	3
response to lipid (GO:0033993)	271	2	0.73	2.74	1.67E-01	1.00E+00	BP	3
reproductive system development (GO:0061458)	273	2	0.73	2.72	1.69E-01	1.00E+00	BP	3
negative regulation of DNA-templated transcription (GO:0045892)	140	1	0.38	2.65	3.16E-01	1.00E+00	BP	3
negative regulation of RNA biosynthetic process (GO:1902679)	140	1	0.38	2.65	3.16E-01	1.00E+00	BP	3
intracellular monoatomic ion homeostasis (GO:0006873)	140	1	0.38	2.65	3.16E-01	1.00E+00	BP	3
organophosphate ester transport (GO:0015748)	141	1	0.38	2.64	3.18E-01	1.00E+00	BP	3
endoplasmic reticulum to Golgi vesicle-mediated transport (GO:0006888)	142	1	0.38	2.62	3.20E-01	1.00E+00	BP	3
membrane organization (GO:0061024)	287	2	0.77	2.59	1.82E-01	1.00E+00	BP	3
metal ion transport (GO:0030001)	287	2	0.77	2.59	1.82E-01	1.00E+00	BP	3
regulation of organelle organization (GO:0033043)	144	1	0.39	2.58	3.23E-01	1.00E+00	BP	3
response to other organism (GO:0051707)	437	3	1.18	2.55	1.15E-01	1.00E+00	BP	3
response to external biotic stimulus (GO:0043207)	437	3	1.18	2.55	1.15E-01	1.00E+00	BP	3
response to abscisic acid (GO:0009737)	148	1	0.4	2.51	3.30E-01	1.00E+00	BP	3
establishment of protein localization to membrane (GO:0090150)	148	1	0.4	2.51	3.30E-01	1.00E+00	BP	3

GO Term	Genome	Count	Expected	Fold Enrichment	P-value	FDR	Term	Cluster
negative regulation of RNA metabolic process (GO:0051253)	149	1	0.4	2.49	3.32E-01	1.00E+00	BP	3
response to alcohol (GO:0097305)	149	1	0.4	2.49	3.32E-01	1.00E+00	BP	3
fatty acid biosynthetic process (GO:0006633)	149	1	0.4	2.49	3.32E-01	1.00E+00	BP	3
lipid transport (GO:0006869)	150	1	0.4	2.48	3.34E-01	1.00E+00	BP	3
biological process involved in interspecies interaction between organisms (GO:0044419)	457	3	1.23	2.44	1.27E-01	1.00E+00	BP	3
developmental process involved in reproduction (GO:0003006)	307	2	0.83	2.42	2.01E-01	1.00E+00	BP	3
plastid organization (GO:0009657)	154	1	0.41	2.41	3.41E-01	1.00E+00	BP	3
regulation of response to stimulus (GO:0048583)	310	2	0.83	2.4	2.04E-01	1.00E+00	BP	3
response to biotic stimulus (GO:0009607)	470	3	1.26	2.37	1.35E-01	1.00E+00	BP	3
multicellular organism development (GO:0007275)	627	4	1.69	2.37	9.04E-02	1.00E+00	BP	3
intracellular chemical homeostasis (GO:0055082)	157	1	0.42	2.37	3.47E-01	1.00E+00	BP	3
export from cell (GO:0140352)	159	1	0.43	2.34	3.50E-01	1.00E+00	BP	3
system development (GO:0048731)	482	3	1.3	2.31	1.42E-01	1.00E+00	BP	3
developmental process (GO:0032502)	965	6	2.6	2.31	4.71E-02	1.00E+00	BP	3
auxin-activated signaling pathway (GO:0009734)	161	1	0.43	2.31	3.54E-01	1.00E+00	BP	3
cellular response to auxin stimulus (GO:0071365)	161	1	0.43	2.31	3.54E-01	1.00E+00	BP	3
monoatomic cation homeostasis (GO:0055080)	161	1	0.43	2.31	3.54E-01	1.00E+00	BP	3
cellular response to lipid (GO:0071396)	162	1	0.44	2.29	3.55E-01	1.00E+00	BP	3
regulation of signal transduction (GO:0009966)	162	1	0.44	2.29	3.55E-01	1.00E+00	BP	3
lipid localization (GO:0010876)	163	1	0.44	2.28	3.57E-01	1.00E+00	BP	3
regulation of signaling (GO:0023051)	163	1	0.44	2.28	3.57E-01	1.00E+00	BP	3
double-strand break repair via homologous recombination (GO:0000724)	164	1	0.44	2.27	3.59E-01	1.00E+00	BP	3
defense response to other organism (GO:0098542)	332	2	0.89	2.24	2.26E-01	1.00E+00	BP	3
organelle localization (GO:0051640)	166	1	0.45	2.24	3.62E-01	1.00E+00	BP	3
regulation of cell communication (GO:0010646)	166	1	0.45	2.24	3.62E-01	1.00E+00	BP	3
cytoskeleton organization (GO:0007010)	340	2	0.91	2.19	2.34E-01	1.00E+00	BP	3
protein phosphorylation (GO:0006468)	1543	9	4.15	2.17	3.81E-02	1.00E+00	BP	3
multicellular organismal process (GO:0032501)	686	4	1.85	2.17	1.15E-01	1.00E+00	BP	3
defense response (GO:0006952)	515	3	1.39	2.16	1.63E-01	1.00E+00	BP	3
chromatin remodeling (GO:0006338)	346	2	0.93	2.15	2.40E-01	1.00E+00	BP	3
post-embryonic development (GO:0009791)	348	2	0.94	2.14	2.42E-01	1.00E+00	BP	3
nucleobase-containing compound transport (GO:0015931)	174	1	0.47	2.14	3.76E-01	1.00E+00	BP	3
negative regulation of nucleobase-containing compound metabolic process (GO:0045934)	175	1	0.47	2.12	3.78E-01	1.00E+00	BP	3
regulation of cellular component organization (GO:0051128)	176	1	0.47	2.11	3.79E-01	1.00E+00	BP	3
monoatomic ion homeostasis (GO:0050801)	180	1	0.48	2.06	3.86E-01	1.00E+00	BP	3
peptidyl-amino acid modification (GO:0018193)	544	3	1.46	2.05	1.82E-01	1.00E+00	BP	3
protein localization to membrane (GO:0072657)	183	1	0.49	2.03	3.91E-01	1.00E+00	BP	3
protein glycosylation (GO:0006486)	185	1	0.5	2.01	3.94E-01	1.00E+00	BP	3
macromolecule glycosylation (GO:0043413)	185	1	0.5	2.01	3.94E-01	1.00E+00	BP	3
glycerophospholipid biosynthetic process (GO:0046474)	185	1	0.5	2.01	3.94E-01	1.00E+00	BP	3
protein dephosphorylation (GO:0006470)	186	1	0.5	2	3.96E-01	1.00E+00	BP	3
monocarboxylic acid biosynthetic process (GO:0072330)	186	1	0.5	2	3.96E-01	1.00E+00	BP	3
glycoprotein biosynthetic process (GO:0009101)	187	1	0.5	1.99	3.97E-01	1.00E+00	BP	3
glycosylation (GO:0070085)	191	1	0.51	1.95	4.04E-01	1.00E+00	BP	3
cellular homeostasis (GO:0019725)	193	1	0.52	1.93	4.07E-01	1.00E+00	BP	3
response to external stimulus (GO:0009605)	583	3	1.57	1.91	2.09E-01	1.00E+00	BP	3
cell wall organization or biogenesis (GO:0071554)	586	3	1.58	1.9	2.11E-01	1.00E+00	BP	3
glycerolipid biosynthetic process (GO:0045017)	196	1	0.53	1.9	4.12E-01	1.00E+00	BP	3
macromolecule methylation (GO:0043414)	198	1	0.53	1.88	4.15E-01	1.00E+00	BP	3
response to temperature stimulus (GO:0009266)	202	1	0.54	1.84	4.21E-01	1.00E+00	BP	3

GO Term	Genome	Count	Expected	Fold Enrichment	P-value	FDR	Term	Cluster
response to abiotic stimulus (GO:0009628)	611	3	1.64	1.82	2.28E-01	1.00E+00	BP	3
glycoprotein metabolic process (GO:0009100)	205	1	0.55	1.81	4.26E-01	1.00E+00	BP	3
double-strand break repair (GO:0006302)	205	1	0.55	1.81	4.26E-01	1.00E+00	BP	3
anatomical structure development (GO:0048856)	825	4	2.22	1.8	2.89E-01	1.00E+00	BP	3
macromolecule modification (GO:0043412)	3738	18	10.06	1.79	1.84E-02	1.00E+00	BP	3
chromatin organization (GO:0006325)	417	2	1.12	1.78	3.10E-01	1.00E+00	BP	3
inorganic cation transmembrane transport (GO:0098662)	420	2	1.13	1.77	3.13E-01	1.00E+00	BP	3
protein modification process (GO:0036211)	3194	15	8.6	1.75	3.11E-02	1.00E+00	BP	3
localization within membrane (GO:0051668)	216	1	0.58	1.72	4.43E-01	1.00E+00	BP	3
cellular localization (GO:0051641)	1297	6	3.49	1.72	1.67E-01	1.00E+00	BP	3
phosphorylation (GO:0016310)	2164	10	5.82	1.72	8.48E-02	1.00E+00	BP	3
shoot system development (GO:0048367)	221	1	0.59	1.68	4.50E-01	1.00E+00	BP	3
monoatomic cation transmembrane transport (GO:0098655)	447	2	1.2	1.66	3.40E-01	1.00E+00	BP	3
fatty acid metabolic process (GO:0006631)	235	1	0.63	1.58	4.71E-01	1.00E+00	BP	3
phospholipid biosynthetic process (GO:0008654)	239	1	0.64	1.55	4.76E-01	1.00E+00	BP	3
cellular response to oxygen-containing compound (GO:1901701)	239	1	0.64	1.55	4.76E-01	1.00E+00	BP	3
peptidyl-lysine modification (GO:0018205)	240	1	0.65	1.55	4.78E-01	1.00E+00	BP	3
glycerophospholipid metabolic process (GO:0006650)	241	1	0.65	1.54	4.79E-01	1.00E+00	BP	3
inorganic ion transmembrane transport (GO:0098660)	489	2	1.32	1.52	3.80E-01	1.00E+00	BP	3
response to oxygen-containing compound (GO:1901700)	492	2	1.32	1.51	3.83E-01	1.00E+00	BP	3
transmembrane transport (GO:0055085)	1724	7	4.64	1.51	2.34E-01	1.00E+00	BP	3
monoatomic cation transport (GO:0006812)	496	2	1.33	1.5	3.87E-01	1.00E+00	BP	3
dephosphorylation (GO:0016311)	248	1	0.67	1.5	4.89E-01	1.00E+00	BP	3
establishment of localization (GO:0051234)	3225	13	8.68	1.5	1.52E-01	1.00E+00	BP	3
hormone-mediated signaling pathway (GO:0009755)	497	2	1.34	1.5	3.88E-01	1.00E+00	BP	3
reproductive process (GO:0022414)	503	2	1.35	1.48	3.94E-01	1.00E+00	BP	3
signal transduction (GO:0007165)	1258	5	3.39	1.48	3.95E-01	1.00E+00	BP	3
response to hormone (GO:0009725)	756	3	2.03	1.47	4.61E-01	1.00E+00	BP	3
cellular response to hormone stimulus (GO:0032870)	504	2	1.36	1.47	3.95E-01	1.00E+00	BP	3
protein-containing complex organization (GO:0043933)	1011	4	2.72	1.47	3.53E-01	1.00E+00	BP	3
chemical homeostasis (GO:0048878)	253	1	0.68	1.47	4.96E-01	1.00E+00	BP	3
Golgi vesicle transport (GO:0048193)	253	1	0.68	1.47	4.96E-01	1.00E+00	BP	3
response to endogenous stimulus (GO:0009719)	762	3	2.05	1.46	4.63E-01	1.00E+00	BP	3
cellular response to endogenous stimulus (GO:0071495)	510	2	1.37	1.46	4.00E-01	1.00E+00	BP	3
glycerolipid metabolic process (GO:0046486)	255	1	0.69	1.46	4.99E-01	1.00E+00	BP	3
signaling (GO:0023052)	1277	5	3.44	1.45	3.99E-01	1.00E+00	BP	3
localization (GO:0051179)	3340	13	8.99	1.45	1.62E-01	1.00E+00	BP	3
proteasome-mediated ubiquitin-dependent protein catabolic process (GO:0043161)	259	1	0.7	1.43	5.04E-01	1.00E+00	BP	3
anatomical structure morphogenesis (GO:0009653)	262	1	0.71	1.42	5.08E-01	1.00E+00	BP	3
cell communication (GO:0007154)	1326	5	3.57	1.4	4.10E-01	1.00E+00	BP	3
methylation (GO:0032259)	541	2	1.46	1.37	6.59E-01	1.00E+00	BP	3
negative regulation of macromolecule biosynthetic process (GO:0010558)	271	1	0.73	1.37	5.20E-01	1.00E+00	BP	3
phosphate-containing compound metabolic process (GO:0006796)	2984	11	8.03	1.37	2.68E-01	1.00E+00	BP	3
monoatomic ion transmembrane transport (GO:0034220)	543	2	1.46	1.37	6.60E-01	1.00E+00	BP	3
phosphorus metabolic process (GO:0006793)	3030	11	8.15	1.35	2.74E-01	1.00E+00	BP	3
nitrogen compound transport (GO:0071705)	1381	5	3.72	1.35	4.25E-01	1.00E+00	BP	3
protein-containing complex assembly (GO:0065003)	554	2	1.49	1.34	6.63E-01	1.00E+00	BP	3
negative regulation of cellular biosynthetic process (GO:0031327)	279	1	0.75	1.33	5.30E-01	1.00E+00	BP	3
polysaccharide metabolic process (GO:0005976)	562	2	1.51	1.32	6.65E-01	1.00E+00	BP	3
reproduction (GO:0000003)	562	2	1.51	1.32	6.65E-01	1.00E+00	BP	3
negative regulation of biosynthetic process (GO:0009890)	281	1	0.76	1.32	5.33E-01	1.00E+00	BP	3

GO Term	Genome	Count	Expected	Fold Enrichment	P-value	FDR	Term	Cluster
response to auxin (GO:0009733)	283	1	0.76	1.31	5.35E-01	1.00E+00	BP	3
protein ubiquitination (GO:0016567)	573	2	1.54	1.3	6.69E-01	1.00E+00	BP	3
cellular response to organic substance (GO:0071310)	578	2	1.56	1.29	6.71E-01	1.00E+00	BP	3
transport (GO:0006810)	3186	11	8.57	1.28	3.71E-01	1.00E+00	BP	3
homeostatic process (GO:0042592)	291	1	0.78	1.28	5.45E-01	1.00E+00	BP	3
organic substance transport (GO:0071702)	1759	6	4.73	1.27	4.79E-01	1.00E+00	BP	3
negative regulation of macromolecule metabolic process (GO:0010605)	602	2	1.62	1.23	6.78E-01	1.00E+00	BP	3
monoatomic ion transport (GO:0006811)	609	2	1.64	1.22	6.81E-01	1.00E+00	BP	3
proteasomal protein catabolic process (GO:0010498)	305	1	0.82	1.22	5.62E-01	1.00E+00	BP	3
negative regulation of metabolic process (GO:0009892)	615	2	1.66	1.21	6.83E-01	1.00E+00	BP	3
regulation of cellular process (GO:0050794)	4625	15	12.45	1.21	4.49E-01	1.00E+00	BP	3
protein modification by small protein conjugation (GO:0032446)	618	2	1.66	1.2	6.84E-01	1.00E+00	BP	3
protein metabolic process (GO:0019538)	5254	17	14.14	1.2	3.92E-01	1.00E+00	BP	3
establishment of localization in cell (GO:0051649)	932	3	2.51	1.2	7.42E-01	1.00E+00	BP	3
macromolecule localization (GO:0033036)	1264	4	3.4	1.18	5.86E-01	1.00E+00	BP	3
establishment of protein localization (GO:0045184)	962	3	2.59	1.16	7.46E-01	1.00E+00	BP	3
DNA recombination (GO:0006310)	648	2	1.74	1.15	6.94E-01	1.00E+00	BP	3
cellular component organization (GO:0016043)	2935	9	7.9	1.14	7.09E-01	1.00E+00	BP	3
regulation of gene expression (GO:0010468)	3303	10	8.89	1.13	7.24E-01	1.00E+00	BP	3
phospholipid metabolic process (GO:0006644)	331	1	0.89	1.12	5.92E-01	1.00E+00	BP	3
negative regulation of cellular metabolic process (GO:0031324)	332	1	0.89	1.12	5.93E-01	1.00E+00	BP	3
response to organic substance (GO:0010033)	1000	3	2.69	1.11	7.53E-01	1.00E+00	BP	3
detoxification (GO:0098754)	334	1	0.9	1.11	5.95E-01	1.00E+00	BP	3
carbohydrate metabolic process (GO:0005975)	1347	4	3.62	1.1	7.86E-01	1.00E+00	BP	3
regulation of RNA biosynthetic process (GO:2001141)	2705	8	7.28	1.1	7.01E-01	1.00E+00	BP	3
regulation of DNA-templated transcription (GO:0006355)	2705	8	7.28	1.1	7.01E-01	1.00E+00	BP	3
organonitrogen compound metabolic process (GO:1901564)	6443	19	17.34	1.1	6.93E-01	1.00E+00	BP	3
response to stimulus (GO:0050896)	4415	13	11.88	1.09	7.57E-01	1.00E+00	BP	3
organelle organization (GO:0006996)	1700	5	4.57	1.09	8.08E-01	1.00E+00	BP	3
negative regulation of gene expression (GO:0010629)	342	1	0.92	1.09	6.04E-01	1.00E+00	BP	3
protein localization to organelle (GO:0033365)	346	1	0.93	1.07	6.08E-01	1.00E+00	BP	3
protein localization (GO:0008104)	1049	3	2.82	1.06	7.62E-01	1.00E+00	BP	3
cellular macromolecule localization (GO:0070727)	1050	3	2.83	1.06	7.62E-01	1.00E+00	BP	3
lipid biosynthetic process (GO:0008610)	706	2	1.9	1.05	7.16E-01	1.00E+00	BP	3
regulation of biological process (GO:0050789)	5297	15	14.25	1.05	7.76E-01	1.00E+00	BP	3
regulation of RNA metabolic process (GO:0051252)	2843	8	7.65	1.05	8.50E-01	1.00E+00	BP	3
cellular process (GO:0009987)	15372	43	41.37	1.04	7.65E-01	1.00E+00	BP	3
response to toxic substance (GO:0009636)	358	1	0.96	1.04	6.21E-01	1.00E+00	BP	3
biological_process (GO:0008150)	21970	61	59.12	1.03	7.69E-01	1.00E+00	BP	3
regulation of nucleobase-containing compound metabolic process (GO:0019219)	2913	8	7.84	1.02	8.53E-01	1.00E+00	BP	3
regulation of macromolecule biosynthetic process (GO:0010556)	2942	8	7.92	1.01	8.55E-01	1.00E+00	BP	3
protein folding (GO:0006457)	368	1	0.99	1.01	1.00E+00	1.00E+00	BP	3
regulation of macromolecule metabolic process (GO:0060255)	3684	10	9.91	1.01	1.00E+00	1.00E+00	BP	3
macromolecule metabolic process (GO:0043170)	8871	24	23.87	1.01	1.00E+00	1.00E+00	BP	3
regulation of cellular biosynthetic process (GO:0031326)	2959	8	7.96	1	1.00E+00	1.00E+00	BP	3
regulation of biosynthetic process (GO:0009889)	2969	8	7.99	1	1.00E+00	1.00E+00	BP	3
positive regulation of biological process (GO:0048518)	749	2	2.02	0.99	1.00E+00	1.00E+00	BP	3
biological regulation (GO:0065007)	5626	15	15.14	0.99	1.00E+00	1.00E+00	BP	3
regulation of metabolic process (GO:0019222)	3766	10	10.13	0.99	1.00E+00	1.00E+00	BP	3
response to chemical (GO:0042221)	1513	4	4.07	0.98	1.00E+00	1.00E+00	BP	3
cellular component organization or biogenesis (GO:0071840)	3437	9	9.25	0.97	1.00E+00	1.00E+00	BP	3

GO Term	Genome	Count	Expected	Fold Enrichment	P-value	FDR	Term	Cluster
cellular component assembly (GO:0022607)	774	2	2.08	0.96	1.00E+00	1.00E+00	BP	3
Unclassified (UNCLASSIFIED)	17419	45	46.88	0.96	7.69E-01	1.00E+00	BP	3
negative regulation of nitrogen compound metabolic process (GO:0051172)	393	1	1.06	0.95	1.00E+00	1.00E+00	BP	3
primary metabolic process (GO:0044238)	11484	29	30.9	0.94	7.49E-01	1.00E+00	BP	3
nitrogen compound metabolic process (GO:0006807)	9609	24	25.86	0.93	7.35E-01	1.00E+00	BP	3
negative regulation of biological process (GO:0048519)	801	2	2.16	0.93	1.00E+00	1.00E+00	BP	3
organic substance metabolic process (GO:0071704)	12450	31	33.5	0.93	6.76E-01	1.00E+00	BP	3
lipid metabolic process (GO:0006629)	1209	3	3.25	0.92	1.00E+00	1.00E+00	BP	3
regulation of cellular metabolic process (GO:0031323)	3270	8	8.8	0.91	1.00E+00	1.00E+00	BP	3
metabolic process (GO:0008152)	13613	33	36.63	0.9	5.40E-01	1.00E+00	BP	3
regulation of nitrogen compound metabolic process (GO:0051171)	3361	8	9.04	0.88	8.62E-01	1.00E+00	BP	3
protein modification by small protein conjugation or removal (GO:0070647)	847	2	2.28	0.88	1.00E+00	1.00E+00	BP	3
regulation of primary metabolic process (GO:0080090)	3400	8	9.15	0.87	8.62E-01	1.00E+00	BP	3
regulation of biological quality (GO:0065008)	433	1	1.17	0.86	1.00E+00	1.00E+00	BP	3
DNA repair (GO:0006281)	872	2	2.35	0.85	1.00E+00	1.00E+00	BP	3
intracellular transport (GO:0046907)	896	2	2.41	0.83	1.00E+00	1.00E+00	BP	3
DNA damage response (GO:0006974)	901	2	2.42	0.82	1.00E+00	1.00E+00	BP	3
cellular metabolic process (GO:0044237)	9471	21	25.49	0.82	3.63E-01	1.00E+00	BP	3
cellular response to stimulus (GO:0051716)	2710	6	7.29	0.82	8.47E-01	1.00E+00	BP	3
RNA modification (GO:0009451)	453	1	1.22	0.82	1.00E+00	1.00E+00	BP	3
cellular lipid metabolic process (GO:0044255)	907	2	2.44	0.82	1.00E+00	1.00E+00	BP	3
response to stress (GO:0006950)	2296	5	6.18	0.81	8.35E-01	1.00E+00	BP	3
mRNA processing (GO:0006397)	460	1	1.24	0.81	1.00E+00	1.00E+00	BP	3
intracellular signal transduction (GO:0035556)	463	1	1.25	0.8	1.00E+00	1.00E+00	BP	3
cellular response to chemical stimulus (GO:0070887)	934	2	2.51	0.8	1.00E+00	1.00E+00	BP	3
monocarboxylic acid metabolic process (GO:0032787)	472	1	1.27	0.79	1.00E+00	1.00E+00	BP	3
protein transport (GO:0015031)	950	2	2.56	0.78	1.00E+00	1.00E+00	BP	3
negative regulation of cellular process (GO:0048523)	484	1	1.3	0.77	1.00E+00	1.00E+00	BP	3
cellular biosynthetic process (GO:0044249)	3461	7	9.31	0.75	6.04E-01	1.00E+00	BP	3
organonitrogen compound biosynthetic process (GO:1901566)	2013	4	5.42	0.74	8.23E-01	1.00E+00	BP	3
carbohydrate derivative biosynthetic process (GO:1901137)	512	1	1.38	0.73	1.00E+00	1.00E+00	BP	3
aromatic compound biosynthetic process (GO:0019438)	1547	3	4.16	0.72	8.01E-01	1.00E+00	BP	3
RNA processing (GO:0006396)	1041	2	2.8	0.71	1.00E+00	1.00E+00	BP	3
organic acid biosynthetic process (GO:0016053)	521	1	1.4	0.71	1.00E+00	1.00E+00	BP	3
carboxylic acid biosynthetic process (GO:0046394)	521	1	1.4	0.71	1.00E+00	1.00E+00	BP	3
DNA metabolic process (GO:0006259)	1050	2	2.83	0.71	1.00E+00	1.00E+00	BP	3
biosynthetic process (GO:0009058)	4259	8	11.46	0.7	3.47E-01	1.00E+00	BP	3
organophosphate biosynthetic process (GO:0090407)	541	1	1.46	0.69	1.00E+00	1.00E+00	BP	3
positive regulation of cellular process (GO:0048522)	597	1	1.61	0.62	1.00E+00	1.00E+00	BP	3
ubiquitin-dependent protein catabolic process (GO:0006511)	601	1	1.62	0.62	1.00E+00	1.00E+00	BP	3
chromosome organization (GO:0051276)	603	1	1.62	0.62	1.00E+00	1.00E+00	BP	3
modification-dependent protein catabolic process (GO:0019941)	614	1	1.65	0.61	1.00E+00	1.00E+00	BP	3
cellular response to stress (GO:0033554)	1229	2	3.31	0.6	7.76E-01	1.00E+00	BP	3
mRNA metabolic process (GO:0016071)	615	1	1.66	0.6	1.00E+00	1.00E+00	BP	3
cellular aromatic compound metabolic process (GO:0006725)	4327	7	11.64	0.6	2.10E-01	1.00E+00	BP	3
modification-dependent macromolecule catabolic process (GO:0043632)	636	1	1.71	0.58	1.00E+00	1.00E+00	BP	3
nucleic acid metabolic process (GO:0090304)	3395	5	9.14	0.55	2.21E-01	1.00E+00	BP	3
organic substance biosynthetic process (GO:1901576)	4099	6	11.03	0.54	1.48E-01	1.00E+00	BP	3
heterocycle metabolic process (GO:0046483)	4164	6	11.21	0.54	1.13E-01	1.00E+00	BP	3
vesicle-mediated transport (GO:0016192)	699	1	1.88	0.53	1.00E+00	1.00E+00	BP	3
cellular component biogenesis (GO:0044085)	1401	2	3.77	0.53	5.93E-01	1.00E+00	BP	3

GO Term	Genome	Count	Expected	Fold Enrichment	P-value	FDR	Term	Cluster
gene expression (GO:0010467)	2102	3	5.66	0.53	3.82E-01	1.00E+00	BP	3
cellular nitrogen compound metabolic process (GO:0034641)	4908	7	13.21	0.53	7.58E-02	1.00E+00	BP	3
small molecule biosynthetic process (GO:0044283)	708	1	1.91	0.52	1.00E+00	1.00E+00	BP	3
proteolysis involved in protein catabolic process (GO:0051603)	726	1	1.95	0.51	1.00E+00	1.00E+00	BP	3
protein catabolic process (GO:0030163)	738	1	1.99	0.5	7.26E-01	1.00E+00	BP	3
organic cyclic compound metabolic process (GO:1901360)	4430	6	11.92	0.5	8.76E-02	1.00E+00	BP	3
heterocycle biosynthetic process (GO:0018130)	1487	2	4	0.5	4.44E-01	1.00E+00	BP	3
carbohydrate derivative metabolic process (GO:1901135)	763	1	2.05	0.49	7.27E-01	1.00E+00	BP	3
nucleobase-containing compound metabolic process (GO:0006139)	3868	5	10.41	0.48	9.90E-02	1.00E+00	BP	3
RNA metabolic process (GO:0016070)	2384	3	6.42	0.47	2.18E-01	1.00E+00	BP	3
cellular nitrogen compound biosynthetic process (GO:0044271)	2423	3	6.52	0.46	2.20E-01	1.00E+00	BP	3
organophosphate metabolic process (GO:0019637)	808	1	2.17	0.46	7.28E-01	1.00E+00	BP	3
macromolecule biosynthetic process (GO:0009059)	2436	3	6.56	0.46	2.20E-01	1.00E+00	BP	3
DNA-templated transcription (GO:0006351)	824	1	2.22	0.45	7.29E-01	1.00E+00	BP	3
organic cyclic compound biosynthetic process (GO:1901362)	1677	2	4.51	0.44	3.32E-01	1.00E+00	BP	3
RNA biosynthetic process (GO:0032774)	840	1	2.26	0.44	7.31E-01	1.00E+00	BP	3
translation (GO:0006412)	947	1	2.55	0.39	5.25E-01	1.00E+00	BP	3
organonitrogen compound catabolic process (GO:1901565)	955	1	2.57	0.39	5.25E-01	1.00E+00	BP	3
peptide biosynthetic process (GO:0043043)	965	1	2.6	0.39	5.26E-01	1.00E+00	BP	3
peptide metabolic process (GO:0006518)	1013	1	2.73	0.37	5.30E-01	1.00E+00	BP	3
amide biosynthetic process (GO:0043604)	1059	1	2.85	0.35	5.37E-01	1.00E+00	BP	3
macromolecule catabolic process (GO:0009057)	1069	1	2.88	0.35	3.75E-01	1.00E+00	BP	3
carboxylic acid metabolic process (GO:0019752)	1117	1	3.01	0.33	3.77E-01	1.00E+00	BP	3
oxoacid metabolic process (GO:0043436)	1130	1	3.04	0.33	3.77E-01	1.00E+00	BP	3
organic acid metabolic process (GO:0006082)	1131	1	3.04	0.33	3.77E-01	1.00E+00	BP	3
amide metabolic process (GO:0043603)	1168	1	3.14	0.32	3.81E-01	1.00E+00	BP	3
nucleobase-containing compound biosynthetic process (GO:0034654)	1266	1	3.41	0.29	2.68E-01	1.00E+00	BP	3
proteolysis (GO:0006508)	1544	1	4.16	0.24	1.33E-01	1.00E+00	BP	3
organic substance catabolic process (GO:1901575)	1863	1	5.01	0.2	6.53E-02	1.00E+00	BP	3
small molecule metabolic process (GO:0044281)	1894	1	5.1	0.2	6.56E-02	1.00E+00	BP	3
catabolic process (GO:0009056)	1947	1	5.24	0.19	6.70E-02	1.00E+00	BP	3
histone H3K27me2/H3K27me3 demethylase activity (GO:0071558)	1	1	0	> 100	5.36E-03	1.00E+00	MF	3
hydroxyproline O-galactosyltransferase activity (GO:1990714)	1	1	0	> 100	5.36E-03	1.00E+00	MF	3
uroporphyrinogen-III synthase activity (GO:0004852)	2	1	0.01	> 100	8.03E-03	1.00E+00	MF	3
1,2-alpha-L-fucosidase activity (GO:0047513)	2	1	0.01	> 100	8.03E-03	1.00E+00	MF	3
alpha-L-fucosidase activity (GO:0004560)	5	1	0.01	74.32	1.60E-02	1.00E+00	MF	3
gamma-aminobutyric acid transmembrane transporter activity (GO:0015185)	6	1	0.02	61.93	1.86E-02	1.00E+00	MF	3
fucosidase activity (GO:0015928)	6	1	0.02	61.93	1.86E-02	1.00E+00	MF	3
ethanolamine kinase activity (GO:0004305)	7	1	0.02	53.08	2.13E-02	1.00E+00	MF	3
choline kinase activity (GO:0004103)	7	1	0.02	53.08	2.13E-02	1.00E+00	MF	3
transmembrane receptor protein tyrosine kinase activity (GO:0004714)	12	1	0.03	30.97	3.43E-02	1.00E+00	MF	3
ATP:ADP antiporter activity (GO:0005471)	12	1	0.03	30.97	3.43E-02	1.00E+00	MF	3
ADP transmembrane transporter activity (GO:0015217)	14	1	0.04	26.54	3.95E-02	1.00E+00	MF	3
endodeoxyribonuclease activity, producing 5'-phosphomonoesters (GO:0016888)	15	1	0.04	24.77	4.21E-02	1.00E+00	MF	3
5'-flap endonuclease activity (GO:0017108)	15	1	0.04	24.77	4.21E-02	1.00E+00	MF	3
5S rRNA binding (GO:0008097)	16	1	0.04	23.22	4.47E-02	1.00E+00	MF	3
flap endonuclease activity (GO:0048256)	17	1	0.05	21.86	4.72E-02	1.00E+00	MF	3
ATP transmembrane transporter activity (GO:0005347)	18	1	0.05	20.64	4.98E-02	1.00E+00	MF	3
strictosidine synthase activity (GO:0016844)	18	1	0.05	20.64	4.98E-02	1.00E+00	MF	3
phospholipase A1 activity (GO:0008970)	19	1	0.05	19.56	5.23E-02	1.00E+00	MF	3
zinc ion transmembrane transporter activity (GO:0005385)	20	1	0.05	18.58	5.49E-02	1.00E+00	MF	3

GO Term	Genome	Count	Expected	Fold Enrichment	P-value	FDR	Term	Cluster
amine-lyase activity (GO:0016843)	21	1	0.06	17.69	5.74E-02	1.00E+00	MF	3
purine ribonucleotide transmembrane transporter activity (GO:0005346)	23	1	0.06	16.16	6.25E-02	1.00E+00	MF	3
transmembrane receptor protein kinase activity (GO:0019199)	46	2	0.12	16.16	7.43E-03	1.00E+00	MF	3
quercetin 7-O-glucosyltransferase activity (GO:0080044)	47	2	0.13	15.81	7.73E-03	1.00E+00	MF	3
quercetin 3-O-glucosyltransferase activity (GO:0080043)	48	2	0.13	15.48	8.04E-03	1.00E+00	MF	3
adenine nucleotide transmembrane transporter activity (GO:0000295)	24	1	0.06	15.48	6.50E-02	1.00E+00	MF	3
fatty acid binding (GO:0005504)	24	1	0.06	15.48	6.50E-02	1.00E+00	MF	3
purine nucleotide transmembrane transporter activity (GO:0015216)	24	1	0.06	15.48	6.50E-02	1.00E+00	MF	3
nucleotide transmembrane transporter activity (GO:0015215)	25	1	0.07	14.86	6.75E-02	1.00E+00	MF	3
transmembrane signaling receptor activity (GO:0004888)	54	2	0.15	13.76	1.00E-02	1.00E+00	MF	3
DNA endonuclease activity (GO:0004520)	28	1	0.08	13.27	7.50E-02	1.00E+00	MF	3
transmembrane receptor protein serine/threonine kinase activity (GO:0004675)	29	1	0.08	12.81	7.75E-02	1.00E+00	MF	3
monocarboxylic acid transmembrane transporter activity (GO:0008028)	30	1	0.08	12.39	8.00E-02	1.00E+00	MF	3
xylan O-acetyltransferase activity (GO:1990538)	61	2	0.16	12.18	1.25E-02	1.00E+00	MF	3
protein demethylase activity (GO:0140457)	31	1	0.08	11.99	8.24E-02	1.00E+00	MF	3
histone demethylase activity (GO:0032452)	31	1	0.08	11.99	8.24E-02	1.00E+00	MF	3
protein kinase binding (GO:0019901)	35	1	0.09	10.62	9.23E-02	1.00E+00	MF	3
O-acetyltransferase activity (GO:0016413)	75	2	0.2	9.91	1.83E-02	1.00E+00	MF	3
chromatin DNA binding (GO:0031490)	38	1	0.1	9.78	9.96E-02	1.00E+00	MF	3
organophosphate ester transmembrane transporter activity (GO:0015605)	38	1	0.1	9.78	9.96E-02	1.00E+00	MF	3
FMN binding (GO:0010181)	43	1	0.12	8.64	1.12E-01	1.00E+00	MF	3
demethylase activity (GO:0032451)	43	1	0.12	8.64	1.12E-01	1.00E+00	MF	3
monocarboxylic acid binding (GO:0033293)	45	1	0.12	8.26	1.16E-01	1.00E+00	MF	3
amino acid transmembrane transporter activity (GO:0015171)	96	2	0.26	7.74	2.87E-02	1.00E+00	MF	3
endonuclease activity, active with either ribo- or deoxyribonucleic acids and producing 5'-phosphomonoesters (GO:0016893)	50	1	0.13	7.43	1.28E-01	1.00E+00	MF	3
kinase binding (GO:0019900)	50	1	0.13	7.43	1.28E-01	1.00E+00	MF	3
polygalacturonase activity (GO:0004650)	56	1	0.15	6.64	1.42E-01	1.00E+00	MF	3
protein tyrosine kinase activity (GO:0004713)	57	1	0.15	6.52	1.44E-01	1.00E+00	MF	3
xenobiotic transmembrane transporter activity (GO:0042910)	58	1	0.16	6.41	1.47E-01	1.00E+00	MF	3
DNA nuclease activity (GO:0004536)	58	1	0.16	6.41	1.47E-01	1.00E+00	MF	3
galactosyltransferase activity (GO:0008378)	59	1	0.16	6.3	1.49E-01	1.00E+00	MF	3
organic anion transmembrane transporter activity (GO:0008514)	184	3	0.5	6.06	1.41E-02	1.00E+00	MF	3
O-methyltransferase activity (GO:0008171)	62	1	0.17	5.99	1.56E-01	1.00E+00	MF	3
SNAP receptor activity (GO:0005484)	63	1	0.17	5.9	1.58E-01	1.00E+00	MF	3
carbon-nitrogen lyase activity (GO:0016840)	64	1	0.17	5.81	1.60E-01	1.00E+00	MF	3
copper ion binding (GO:0005507)	69	1	0.19	5.39	1.72E-01	1.00E+00	MF	3
carboxylic acid transmembrane transporter activity (GO:0046943)	140	2	0.38	5.31	5.61E-02	1.00E+00	MF	3
organic acid transmembrane transporter activity (GO:0005342)	140	2	0.38	5.31	5.61E-02	1.00E+00	MF	3
transition metal ion transmembrane transporter activity (GO:0046915)	72	1	0.19	5.16	1.78E-01	1.00E+00	MF	3
O-acyltransferase activity (GO:0008374)	147	2	0.4	5.06	6.10E-02	1.00E+00	MF	3
acetyltransferase activity (GO:0016407)	148	2	0.4	5.02	6.17E-02	1.00E+00	MF	3
carbohydrate binding (GO:0030246)	314	4	0.85	4.73	1.08E-02	1.00E+00	MF	3
signaling receptor activity (GO:0038023)	171	2	0.46	4.35	7.90E-02	1.00E+00	MF	3
potassium ion transmembrane transporter activity (GO:0015079)	87	1	0.23	4.27	2.11E-01	1.00E+00	MF	3
polysaccharide binding (GO:0030247)	87	1	0.23	4.27	2.11E-01	1.00E+00	MF	3
phospholipase activity (GO:0004620)	89	1	0.24	4.18	2.15E-01	1.00E+00	MF	3
molecular transducer activity (GO:0060089)	179	2	0.48	4.15	8.54E-02	1.00E+00	MF	3
2-oxoglutarate-dependent dioxygenase activity (GO:0016706)	90	1	0.24	4.13	2.17E-01	1.00E+00	MF	3
antiporter activity (GO:0015297)	181	2	0.49	4.11	8.70E-02	1.00E+00	MF	3
nucleobase-containing compound transmembrane transporter activity (GO:0015932)	91	1	0.24	4.08	2.19E-01	1.00E+00	MF	3
carbohydrate derivative transmembrane transporter activity (GO:1901505)	95	1	0.26	3.91	2.28E-01	1.00E+00	MF	3

GO Term	Genome	Count	Expected	Fold Enrichment	P-value	FDR	Term	Cluster
salt transmembrane transporter activity (GO:1901702)	300	3	0.81	3.72	4.84E-02	1.00E+00	MF	3
carboxylic acid binding (GO:0031406)	101	1	0.27	3.68	2.40E-01	1.00E+00	MF	3
organic acid binding (GO:0043177)	101	1	0.27	3.68	2.40E-01	1.00E+00	MF	3
histone binding (GO:0042393)	104	1	0.28	3.57	2.46E-01	1.00E+00	MF	3
actin filament binding (GO:0051015)	104	1	0.28	3.57	2.46E-01	1.00E+00	MF	3
damaged DNA binding (GO:0003684)	109	1	0.29	3.41	2.56E-01	1.00E+00	MF	3
hydro-lyase activity (GO:0016836)	110	1	0.3	3.38	2.58E-01	1.00E+00	MF	3
protein-macromolecule adaptor activity (GO:0030674)	116	1	0.31	3.2	2.70E-01	1.00E+00	MF	3
histone modifying activity (GO:0140993)	119	1	0.32	3.12	2.76E-01	1.00E+00	MF	3
metal ion transmembrane transporter activity (GO:0046873)	245	2	0.66	3.03	1.43E-01	1.00E+00	MF	3
molecular adaptor activity (GO:0060090)	124	1	0.33	3	2.86E-01	1.00E+00	MF	3
ABC-type transporter activity (GO:0140359)	129	1	0.35	2.88	2.95E-01	1.00E+00	MF	3
ADP binding (GO:0043531)	134	1	0.36	2.77	3.05E-01	1.00E+00	MF	3
protein folding chaperone (GO:0044183)	138	1	0.37	2.69	3.12E-01	1.00E+00	MF	3
GTPase activity (GO:0003924)	280	2	0.75	2.65	1.75E-01	1.00E+00	MF	3
lipase activity (GO:0016298)	141	1	0.38	2.64	3.18E-01	1.00E+00	MF	3
transcription regulatory region nucleic acid binding (GO:0001067)	589	4	1.59	2.52	7.59E-02	1.00E+00	MF	3
transcription cis-regulatory region binding (GO:0000976)	589	4	1.59	2.52	7.59E-02	1.00E+00	MF	3
UDP-glucosyltransferase activity (GO:0035251)	298	2	0.8	2.49	1.93E-01	1.00E+00	MF	3
sequence-specific double-stranded DNA binding (GO:1990837)	608	4	1.64	2.44	8.30E-02	1.00E+00	MF	3
endonuclease activity (GO:0004519)	154	1	0.41	2.41	3.41E-01	1.00E+00	MF	3
actin binding (GO:0003779)	159	1	0.43	2.34	3.50E-01	1.00E+00	MF	3
single-stranded DNA binding (GO:0003697)	161	1	0.43	2.31	3.54E-01	1.00E+00	MF	3
rRNA binding (GO:0019843)	162	1	0.44	2.29	3.55E-01	1.00E+00	MF	3
carboxylic ester hydrolase activity (GO:0052689)	165	1	0.44	2.25	3.60E-01	1.00E+00	MF	3
glucosyltransferase activity (GO:0046527)	340	2	0.91	2.19	2.34E-01	1.00E+00	MF	3
dioxygenase activity (GO:0051213)	172	1	0.46	2.16	3.72E-01	1.00E+00	MF	3
secondary active transmembrane transporter activity (GO:0015291)	347	2	0.93	2.14	2.41E-01	1.00E+00	MF	3
chromatin binding (GO:0003682)	175	1	0.47	2.12	3.78E-01	1.00E+00	MF	3
protein kinase activity (GO:0004672)	1604	9	4.32	2.09	4.16E-02	1.00E+00	MF	3
double-stranded DNA binding (GO:0003690)	723	4	1.95	2.06	1.32E-01	1.00E+00	MF	3
phosphotransferase activity, alcohol group as acceptor (GO:0016773)	1823	10	4.91	2.04	3.21E-02	1.00E+00	MF	3
guanyl ribonucleotide binding (GO:0032561)	375	2	1.01	1.98	2.69E-01	1.00E+00	MF	3
GTP binding (GO:0005525)	375	2	1.01	1.98	2.69E-01	1.00E+00	MF	3
guanyl nucleotide binding (GO:0019001)	375	2	1.01	1.98	2.69E-01	1.00E+00	MF	3
lyase activity (GO:0016829)	572	3	1.54	1.95	2.01E-01	1.00E+00	MF	3
carbon-oxygen lyase activity (GO:0016835)	191	1	0.51	1.95	4.04E-01	1.00E+00	MF	3
sequence-specific DNA binding (GO:0043565)	1176	6	3.16	1.9	1.39E-01	1.00E+00	MF	3
inorganic cation transmembrane transporter activity (GO:0022890)	407	2	1.1	1.83	3.00E-01	1.00E+00	MF	3
hexosyltransferase activity (GO:0016758)	637	3	1.71	1.75	2.47E-01	1.00E+00	MF	3
UDP-glycosyltransferase activity (GO:0008194)	428	2	1.15	1.74	3.21E-01	1.00E+00	MF	3
active transmembrane transporter activity (GO:0022804)	645	3	1.74	1.73	2.52E-01	1.00E+00	MF	3
protein-containing complex binding (GO:0044877)	431	2	1.16	1.72	3.24E-01	1.00E+00	MF	3
monoatomic cation transmembrane transporter activity (GO:0008324)	437	2	1.18	1.7	3.30E-01	1.00E+00	MF	3
S-adenosylmethionine-dependent methyltransferase activity (GO:0008757)	222	1	0.6	1.67	4.52E-01	1.00E+00	MF	3
kinase activity (GO:0016301)	2226	10	5.99	1.67	9.29E-02	1.00E+00	MF	3
transmembrane transporter activity (GO:0022857)	1612	7	4.34	1.61	2.10E-01	1.00E+00	MF	3
hydrolase activity, hydrolyzing O-glycosyl compounds (GO:0004553)	462	2	1.24	1.61	3.54E-01	1.00E+00	MF	3
ATPase-coupled transmembrane transporter activity (GO:0042626)	231	1	0.62	1.61	4.65E-01	1.00E+00	MF	3
transporter activity (GO:0005215)	1678	7	4.52	1.55	2.23E-01	1.00E+00	MF	3
glycosyltransferase activity (GO:0016757)	972	4	2.62	1.53	3.37E-01	1.00E+00	MF	3

GO Term	Genome	Count	Expected	Fold Enrichment	P-value	FDR	Term	Cluster
myosin phosphatase activity (GO:0017018)	243	1	0.65	1.53	4.82E-01	1.00E+00	MF	3
protein serine/threonine phosphatase activity (GO:0004722)	249	1	0.67	1.49	4.90E-01	1.00E+00	MF	3
ribonucleoside triphosphate phosphatase activity (GO:0017111)	755	3	2.03	1.48	4.61E-01	1.00E+00	MF	3
transferase activity, transferring phosphorus-containing groups (GO:0016772)	2537	10	6.83	1.46	2.29E-01	1.00E+00	MF	3
oxidoreductase activity, acting on paired donors, with incorporation or reduction of molecular oxygen (GO:0016705)	513	2	1.38	1.45	4.03E-01	1.00E+00	MF	3
hydrolase activity, acting on glycosyl bonds (GO:0016798)	525	2	1.41	1.42	6.55E-01	1.00E+00	MF	3
monoatomic ion transmembrane transporter activity (GO:0015075)	530	2	1.43	1.4	6.56E-01	1.00E+00	MF	3
acyltransferase activity, transferring groups other than amino-acyl groups (GO:0016747)	531	2	1.43	1.4	6.56E-01	1.00E+00	MF	3
enzyme binding (GO:0019899)	271	1	0.73	1.37	5.20E-01	1.00E+00	MF	3
ribonucleotide binding (GO:0032553)	3896	14	10.48	1.34	2.53E-01	1.00E+00	MF	3
carbohydrate derivative binding (GO:0097367)	3928	14	10.57	1.32	2.56E-01	1.00E+00	MF	3
inorganic molecular entity transmembrane transporter activity (GO:0015318)	562	2	1.51	1.32	6.65E-01	1.00E+00	MF	3
pyrophosphatase activity (GO:0016462)	851	3	2.29	1.31	5.01E-01	1.00E+00	MF	3
nuclease activity (GO:0004518)	287	1	0.77	1.29	5.40E-01	1.00E+00	MF	3
hydrolase activity, acting on acid anhydrides, in phosphorus-containing anhydrides (GO:0016818)	864	3	2.33	1.29	5.07E-01	1.00E+00	MF	3
DNA binding (GO:0003677)	3182	11	8.56	1.28	3.70E-01	1.00E+00	MF	3
hydrolase activity, acting on acid anhydrides (GO:0016817)	875	3	2.35	1.27	5.12E-01	1.00E+00	MF	3
phosphoprotein phosphatase activity (GO:0004721)	294	1	0.79	1.26	5.49E-01	1.00E+00	MF	3
purine ribonucleotide binding (GO:0032555)	3854	13	10.37	1.25	4.11E-01	1.00E+00	MF	3
primary active transmembrane transporter activity (GO:0015399)	298	1	0.8	1.25	5.54E-01	1.00E+00	MF	3
anion binding (GO:0043168)	4484	15	12.07	1.24	3.58E-01	1.00E+00	MF	3
cis-regulatory region sequence-specific DNA binding (GO:0000987)	299	1	0.8	1.24	5.55E-01	1.00E+00	MF	3
transferase activity (GO:0016740)	5764	19	15.51	1.22	3.35E-01	1.00E+00	MF	3
small molecule binding (GO:0036094)	4599	15	12.38	1.21	4.47E-01	1.00E+00	MF	3
protein serine/threonine kinase activity (GO:0004674)	923	3	2.48	1.21	7.40E-01	1.00E+00	MF	3
purine ribonucleoside triphosphate binding (GO:0035639)	3719	12	10.01	1.2	5.04E-01	1.00E+00	MF	3
nucleotide binding (GO:0000166)	4340	14	11.68	1.2	4.38E-01	1.00E+00	MF	3
nucleoside phosphate binding (GO:1901265)	4340	14	11.68	1.2	4.38E-01	1.00E+00	MF	3
purine nucleotide binding (GO:0017076)	4059	13	10.92	1.19	5.20E-01	1.00E+00	MF	3
DNA-binding transcription factor activity (GO:0003700)	1586	5	4.27	1.17	6.20E-01	1.00E+00	MF	3
adenyl ribonucleotide binding (GO:0032559)	3497	11	9.41	1.17	6.06E-01	1.00E+00	MF	3
protein dimerization activity (GO:0046983)	649	2	1.75	1.15	6.95E-01	1.00E+00	MF	3
catalytic activity, acting on a protein (GO:0140096)	3926	12	10.57	1.14	6.25E-01	1.00E+00	MF	3
ATP binding (GO:0005524)	3362	10	9.05	1.11	7.27E-01	1.00E+00	MF	3
adenyl nucleotide binding (GO:0030554)	3702	11	9.96	1.1	7.38E-01	1.00E+00	MF	3
ion binding (GO:0043167)	7841	23	21.1	1.09	6.27E-01	1.00E+00	MF	3
lipid binding (GO:0008289)	348	1	0.94	1.07	6.10E-01	1.00E+00	MF	3
transcription regulator activity (GO:0140110)	1753	5	4.72	1.06	8.12E-01	1.00E+00	MF	3
ubiquitin protein ligase activity (GO:0061630)	354	1	0.95	1.05	6.16E-01	1.00E+00	MF	3
ubiquitin-like protein ligase activity (GO:0061659)	360	1	0.97	1.03	6.23E-01	1.00E+00	MF	3
Unclassified (UNCLASSIFIED)	16329	45	43.94	1.02	8.44E-01	1.00E+00	MF	3
heterocyclic compound binding (GO:1901363)	10349	28	27.85	1.01	1.00E+00	1.00E+00	MF	3
organic cyclic compound binding (GO:0097159)	10373	28	27.91	1	1.00E+00	1.00E+00	MF	3
binding (GO:0005488)	14290	38	38.46	0.99	1.00E+00	1.00E+00	MF	3
molecular_function (GO:0003674)	23060	61	62.06	0.98	8.44E-01	1.00E+00	MF	3
catalytic activity (GO:0003824)	13387	35	36.03	0.97	9.18E-01	1.00E+00	MF	3
acyltransferase activity (GO:0016746)	1155	3	3.11	0.97	1.00E+00	1.00E+00	MF	3
hydrolase activity (GO:0016787)	4566	11	12.29	0.9	8.79E-01	1.00E+00	MF	3
hydrolase activity, acting on ester bonds (GO:0016788)	1254	3	3.37	0.89	1.00E+00	1.00E+00	MF	3
transition metal ion binding (GO:0046914)	1290	3	3.47	0.86	1.00E+00	1.00E+00	MF	3

GO Term	Genome	Count	Expected	Fold Enrichment	P-value	FDR	Term	Cluster
nucleic acid binding (GO:0003676)	5685	13	15.3	0.85	6.77E-01	1.00E+00	MF	3
cytoskeletal protein binding (GO:0008092)	440	1	1.18	0.84	1.00E+00	1.00E+00	MF	3
monooxygenase activity (GO:0004497)	441	1	1.19	0.84	1.00E+00	1.00E+00	MF	3
protein binding (GO:0005515)	2323	5	6.25	0.8	8.35E-01	1.00E+00	MF	3
iron ion binding (GO:0005506)	465	1	1.25	0.8	1.00E+00	1.00E+00	MF	3
metal ion binding (GO:0046872)	3774	8	10.16	0.79	6.19E-01	1.00E+00	MF	3
cation binding (GO:0043169)	3802	8	10.23	0.78	6.20E-01	1.00E+00	MF	3
ATP hydrolysis activity (GO:0016887)	477	1	1.28	0.78	1.00E+00	1.00E+00	MF	3
phosphatase activity (GO:0016791)	505	1	1.36	0.74	1.00E+00	1.00E+00	MF	3
oxidoreductase activity (GO:0016491)	2048	4	5.51	0.73	6.63E-01	1.00E+00	MF	3
ligase activity (GO:0016874)	529	1	1.42	0.7	1.00E+00	1.00E+00	MF	3
methyltransferase activity (GO:0008168)	532	1	1.43	0.7	1.00E+00	1.00E+00	MF	3
transferase activity, transferring one-carbon groups (GO:0016741)	549	1	1.48	0.68	1.00E+00	1.00E+00	MF	3
ubiquitin-protein transferase activity (GO:0004842)	558	1	1.5	0.67	1.00E+00	1.00E+00	MF	3
heme binding (GO:0020037)	565	1	1.52	0.66	1.00E+00	1.00E+00	MF	3
phosphoric ester hydrolase activity (GO:0042578)	576	1	1.55	0.65	1.00E+00	1.00E+00	MF	3
ubiquitin-like protein transferase activity (GO:0019787)	578	1	1.56	0.64	1.00E+00	1.00E+00	MF	3
aminoacyltransferase activity (GO:0016755)	583	1	1.57	0.64	1.00E+00	1.00E+00	MF	3
structural constituent of ribosome (GO:0003735)	593	1	1.6	0.63	1.00E+00	1.00E+00	MF	3
tetrapyrrole binding (GO:0046906)	609	1	1.64	0.61	1.00E+00	1.00E+00	MF	3
catalytic activity, acting on DNA (GO:0140097)	617	1	1.66	0.6	1.00E+00	1.00E+00	MF	3
mRNA binding (GO:0003729)	651	1	1.75	0.57	1.00E+00	1.00E+00	MF	3
zinc ion binding (GO:0008270)	651	1	1.75	0.57	1.00E+00	1.00E+00	MF	3
structural molecule activity (GO:0005198)	803	1	2.16	0.46	7.28E-01	1.00E+00	MF	3
RNA binding (GO:0003723)	2087	2	5.62	0.36	1.30E-01	1.00E+00	MF	3
catalytic activity, acting on a nucleic acid (GO:0140640)	1293	1	3.48	0.29	2.69E-01	1.00E+00	MF	3
ATP-dependent activity (GO:0140657)	1364	1	3.67	0.27	2.76E-01	1.00E+00	MF	3
ERCC4-ERCC1 complex (GO:0070522)	1	1	0	> 100	5.36E-03	1.00E+00	CC	3
nucleotide-excision repair factor 1 complex (GO:0000110)	2	1	0.01	> 100	8.03E-03	1.00E+00	CC	3
nucleotide-excision repair complex (GO:0000109)	5	1	0.01	74.32	1.60E-02	1.00E+00	CC	3
NSL complex (GO:0044545)	5	1	0.01	74.32	1.60E-02	1.00E+00	CC	3
cell projection (GO:0042995)	16	1	0.04	23.22	4.47E-02	1.00E+00	CC	3
H4 histone acetyltransferase complex (GO:1902562)	28	1	0.08	13.27	7.50E-02	1.00E+00	CC	3
kinetochore (GO:0000776)	36	1	0.1	10.32	9.47E-02	1.00E+00	CC	3
condensed chromosome, centromeric region (GO:0000779)	38	1	0.1	9.78	9.96E-02	1.00E+00	CC	3
chromosome, centromeric region (GO:0000775)	41	1	0.11	9.06	1.07E-01	1.00E+00	CC	3
histone acetyltransferase complex (GO:0000123)	60	1	0.16	6.19	1.51E-01	1.00E+00	CC	3
chromosomal region (GO:0098687)	67	1	0.18	5.55	1.67E-01	1.00E+00	CC	3
trans-Golgi network (GO:0005802)	68	1	0.18	5.46	1.69E-01	1.00E+00	CC	3
condensed chromosome (GO:0000793)	71	1	0.19	5.23	1.76E-01	1.00E+00	CC	3
acetyltransferase complex (GO:1902493)	72	1	0.19	5.16	1.78E-01	1.00E+00	CC	3
protein acetyltransferase complex (GO:0031248)	72	1	0.19	5.16	1.78E-01	1.00E+00	CC	3
SCF ubiquitin ligase complex (GO:0019005)	76	1	0.2	4.89	1.87E-01	1.00E+00	CC	3
Golgi apparatus subcompartment (GO:0098791)	115	1	0.31	3.23	2.68E-01	1.00E+00	CC	3
Golgi membrane (GO:0000139)	394	3	1.06	2.83	9.16E-02	1.00E+00	CC	3
Golgi apparatus (GO:0005794)	959	7	2.58	2.71	1.55E-02	1.00E+00	CC	3
cullin-RING ubiquitin ligase complex (GO:0031461)	142	1	0.38	2.62	3.20E-01	1.00E+00	CC	3
vacuolar membrane (GO:0005774)	149	1	0.4	2.49	3.32E-01	1.00E+00	CC	3
vacuole (GO:0005773)	298	2	0.8	2.49	1.93E-01	1.00E+00	CC	3
plasma membrane (GO:0005886)	1449	9	3.9	2.31	1.67E-02	1.00E+00	CC	3
apoplast (GO:0048046)	175	1	0.47	2.12	3.78E-01	1.00E+00	CC	3

GO Term	Genome	Count	Expected	Fold Enrichment	P-value	FDR	Term	Cluster
cell periphery (GO:0071944)	1669	9	4.49	2	4.67E-02	1.00E+00	CC	3
ubiquitin ligase complex (GO:0000151)	212	1	0.57	1.75	4.37E-01	1.00E+00	CC	3
membrane (GO:0016020)	8074	38	21.73	1.75	2.54E-04	2.21E-01	CC	3
cytoplasmic vesicle (GO:0031410)	429	2	1.15	1.73	3.22E-01	1.00E+00	CC	3
intracellular vesicle (GO:0097708)	429	2	1.15	1.73	3.22E-01	1.00E+00	CC	3
vesicle (GO:0031982)	459	2	1.24	1.62	3.51E-01	1.00E+00	CC	3
endomembrane system (GO:0012505)	2115	9	5.69	1.58	1.88E-01	1.00E+00	CC	3
endosome (GO:0005768)	246	1	0.66	1.51	4.86E-01	1.00E+00	CC	3
chromatin (GO:0000785)	246	1	0.66	1.51	4.86E-01	1.00E+00	CC	3
bounding membrane of organelle (GO:0098588)	1033	4	2.78	1.44	3.63E-01	1.00E+00	CC	3
organelle subcompartment (GO:0031984)	563	2	1.52	1.32	6.66E-01	1.00E+00	CC	3
mitochondrial inner membrane (GO:0005743)	282	1	0.76	1.32	5.34E-01	1.00E+00	CC	3
chromosome (GO:0005694)	570	2	1.53	1.3	6.68E-01	1.00E+00	CC	3
cellular_component (GO:0005575)	19828	66	53.36	1.24	1.49E-02	1.00E+00	CC	3
cellular anatomical entity (GO:0110165)	19592	65	52.72	1.23	1.93E-02	1.00E+00	CC	3
organelle membrane (GO:0031090)	1851	6	4.98	1.2	6.41E-01	1.00E+00	CC	3
organelle inner membrane (GO:0019866)	332	1	0.89	1.12	5.93E-01	1.00E+00	CC	3
mitochondrial membrane (GO:0031966)	352	1	0.95	1.06	6.14E-01	1.00E+00	CC	3
mitochondrial envelope (GO:0005740)	366	1	0.98	1.02	6.29E-01	1.00E+00	CC	3
protein-DNA complex (GO:0032993)	378	1	1.02	0.98	1.00E+00	1.00E+00	CC	3
transferase complex (GO:1990234)	772	2	2.08	0.96	1.00E+00	1.00E+00	CC	3
intracellular membrane-bounded organelle (GO:0043231)	10826	28	29.13	0.96	9.13E-01	1.00E+00	CC	3
supramolecular complex (GO:0099080)	387	1	1.04	0.96	1.00E+00	1.00E+00	CC	3
membrane-bounded organelle (GO:0043227)	10848	28	29.19	0.96	9.13E-01	1.00E+00	CC	3
intracellular protein-containing complex (GO:0140535)	792	2	2.13	0.94	1.00E+00	1.00E+00	CC	3
intracellular anatomical structure (GO:0005622)	14114	35	37.98	0.92	6.12E-01	1.00E+00	CC	3
cytoplasm (GO:0005737)	8535	21	22.97	0.91	7.24E-01	1.00E+00	CC	3
nucleus (GO:0005634)	6118	15	16.46	0.91	7.89E-01	1.00E+00	CC	3
intracellular organelle (GO:0043229)	11979	29	32.24	0.9	5.28E-01	1.00E+00	CC	3
organelle (GO:0043226)	11985	29	32.25	0.9	5.28E-01	1.00E+00	CC	3
endoplasmic reticulum membrane (GO:0005789)	446	1	1.2	0.83	1.00E+00	1.00E+00	CC	3
endoplasmic reticulum subcompartment (GO:0098827)	449	1	1.21	0.83	1.00E+00	1.00E+00	CC	3
nuclear outer membrane-endoplasmic reticulum membrane network (GO:0042175)	452	1	1.22	0.82	1.00E+00	1.00E+00	CC	3
endoplasmic reticulum (GO:0005783)	954	2	2.57	0.78	1.00E+00	1.00E+00	CC	3
cytoskeleton (GO:0005856)	478	1	1.29	0.78	1.00E+00	1.00E+00	CC	3
Unclassified (UNCLASSIFIED)	19561	40	52.64	0.76	1.49E-02	1.00E+00	CC	3
mitochondrion (GO:0005739)	1131	2	3.04	0.66	7.72E-01	1.00E+00	CC	3
intracellular non-membrane-bounded organelle (GO:0043232)	2611	4	7.03	0.57	3.26E-01	1.00E+00	CC	3
non-membrane-bounded organelle (GO:0043228)	2611	4	7.03	0.57	3.26E-01	1.00E+00	CC	3
ribosome (GO:0005840)	694	1	1.87	0.54	1.00E+00	1.00E+00	CC	3
extracellular region (GO:0005576)	698	1	1.88	0.53	1.00E+00	1.00E+00	CC	3
catalytic complex (GO:1902494)	1505	2	4.05	0.49	4.44E-01	1.00E+00	CC	3
envelope (GO:0031975)	844	1	2.27	0.44	7.31E-01	1.00E+00	CC	3
organelle envelope (GO:0031967)	844	1	2.27	0.44	7.31E-01	1.00E+00	CC	3
nuclear protein-containing complex (GO:0140513)	991	1	2.67	0.37	5.28E-01	1.00E+00	CC	3
protein-containing complex (GO:0032991)	4069	4	10.95	0.37	2.38E-02	1.00E+00	CC	3
ribonucleoprotein complex (GO:1990904)	1113	1	3	0.33	3.76E-01	1.00E+00	CC	3
chloroplast (GO:0009507)	1128	1	3.04	0.33	3.77E-01	1.00E+00	CC	3
plastid (GO:0009536)	1326	1	3.57	0.28	2.72E-01	1.00E+00	CC	3
cytosol (GO:0005829)	1383	1	3.72	0.27	1.89E-01	1.00E+00	CC	3
regulation of monopolar cell growth (GO:0051513)	5	1	0.01	> 100	9.39E-03	1.00E+00	BP	4

GO Term	Genome	Count	Expected	Fold Enrichment	P-value	FDR	Term	Cluster
post-chaperonin tubulin folding pathway (GO:0007023)	6	1	0.01	> 100	1.10E-02	1.00E+00	BP	4
tubulin complex assembly (GO:0007021)	8	1	0.01	79.41	1.41E-02	1.00E+00	BP	4
cell population proliferation (GO:0008283)	9	1	0.01	70.59	1.56E-02	1.00E+00	BP	4
heat acclimation (GO:0010286)	10	1	0.02	63.53	1.72E-02	1.00E+00	BP	4
regulation of unidimensional cell growth (GO:0051510)	11	1	0.02	57.76	1.87E-02	1.00E+00	BP	4
L-methionine salvage from methylthioadenosine (GO:0019509)	13	1	0.02	48.87	2.18E-02	1.00E+00	BP	4
L-methionine salvage (GO:0071267)	13	1	0.02	48.87	2.18E-02	1.00E+00	BP	4
amino acid salvage (GO:0043102)	13	1	0.02	48.87	2.18E-02	1.00E+00	BP	4
L-methionine biosynthetic process (GO:0071265)	14	1	0.02	45.38	2.33E-02	1.00E+00	BP	4
regulation of cell growth (GO:0001558)	23	1	0.04	27.62	3.71E-02	1.00E+00	BP	4
vegetative to reproductive phase transition of meristem (GO:0010228)	30	1	0.05	21.18	4.76E-02	1.00E+00	BP	4
regulation of cell morphogenesis (GO:0022604)	34	1	0.05	18.69	5.36E-02	1.00E+00	BP	4
methionine biosynthetic process (GO:0009086)	37	1	0.06	17.17	5.80E-02	1.00E+00	BP	4
methionine metabolic process (GO:0006555)	38	1	0.06	16.72	5.95E-02	1.00E+00	BP	4
histone lysine methylation (GO:0034968)	40	1	0.06	15.88	6.25E-02	1.00E+00	BP	4
cytokinin metabolic process (GO:0009690)	45	1	0.07	14.12	6.98E-02	1.00E+00	BP	4
regulation of developmental growth (GO:0048638)	45	1	0.07	14.12	6.98E-02	1.00E+00	BP	4
spindle assembly (GO:0051225)	51	1	0.08	12.46	7.86E-02	1.00E+00	BP	4
cellular metabolic compound salvage (GO:0043094)	52	1	0.08	12.22	8.00E-02	1.00E+00	BP	4
peptidyl-lysine methylation (GO:0018022)	54	1	0.08	11.76	8.29E-02	1.00E+00	BP	4
histone methylation (GO:0016571)	55	1	0.09	11.55	8.44E-02	1.00E+00	BP	4
protein import into nucleus (GO:0006606)	57	1	0.09	11.15	8.72E-02	1.00E+00	BP	4
import into nucleus (GO:0051170)	59	1	0.09	10.77	9.01E-02	1.00E+00	BP	4
sulfur amino acid biosynthetic process (GO:0000097)	59	1	0.09	10.77	9.01E-02	1.00E+00	BP	4
microtubule cytoskeleton organization (GO:0000226)	181	3	0.28	10.53	3.08E-03	1.00E+00	BP	4
peptidyl-threonine dephosphorylation (GO:0035970)	61	1	0.1	10.41	9.30E-02	1.00E+00	BP	4
protein localization to nucleus (GO:0034504)	62	1	0.1	10.25	9.44E-02	1.00E+00	BP	4
chromosome segregation (GO:0007059)	126	2	0.2	10.08	1.74E-02	1.00E+00	BP	4
sulfur amino acid metabolic process (GO:0000096)	64	1	0.1	9.93	9.73E-02	1.00E+00	BP	4
nuclear transport (GO:0051169)	136	2	0.21	9.34	2.00E-02	1.00E+00	BP	4
nucleocytoplasmic transport (GO:0006913)	136	2	0.21	9.34	2.00E-02	1.00E+00	BP	4
xylan acetylation (GO:1990937)	69	1	0.11	9.21	1.04E-01	1.00E+00	BP	4
regulation of anatomical structure morphogenesis (GO:0022603)	73	1	0.11	8.7	1.10E-01	1.00E+00	BP	4
microtubule-based process (GO:0007017)	293	4	0.46	8.67	1.25E-03	1.00E+00	BP	4
aspartate family amino acid biosynthetic process (GO:0009067)	74	1	0.12	8.59	1.11E-01	1.00E+00	BP	4
ethylene-activated signaling pathway (GO:0009873)	75	1	0.12	8.47	1.13E-01	1.00E+00	BP	4
cellular response to ethylene stimulus (GO:0071369)	76	1	0.12	8.36	1.14E-01	1.00E+00	BP	4
spindle organization (GO:0007051)	80	1	0.13	7.94	1.20E-01	1.00E+00	BP	4
protein methylation (GO:0006479)	85	1	0.13	7.47	1.27E-01	1.00E+00	BP	4
protein alkylation (GO:0008213)	85	1	0.13	7.47	1.27E-01	1.00E+00	BP	4
aspartate family amino acid metabolic process (GO:0009066)	92	1	0.14	6.91	1.36E-01	1.00E+00	BP	4
regulation of growth (GO:0040008)	92	1	0.14	6.91	1.36E-01	1.00E+00	BP	4
hormone metabolic process (GO:0042445)	98	1	0.15	6.48	1.44E-01	1.00E+00	BP	4
response to ethylene (GO:0009723)	100	1	0.16	6.35	1.47E-01	1.00E+00	BP	4
response to heat (GO:0009408)	109	1	0.17	5.83	1.59E-01	1.00E+00	BP	4
defense response to other organism (GO:0098542)	332	3	0.52	5.74	1.59E-02	1.00E+00	BP	4
cell differentiation (GO:0030154)	223	2	0.35	5.7	4.90E-02	1.00E+00	BP	4
cytoskeleton organization (GO:0007010)	340	3	0.54	5.61	1.69E-02	1.00E+00	BP	4
cellular developmental process (GO:0048869)	240	2	0.38	5.29	5.57E-02	1.00E+00	BP	4
amine metabolic process (GO:0009308)	130	1	0.2	4.89	1.86E-01	1.00E+00	BP	4
xylan metabolic process (GO:0045491)	131	1	0.21	4.85	1.88E-01	1.00E+00	BP	4

GO Term	Genome	Count	Expected	Fold Enrichment	P-value	FDR	Term	Cluster
regulation of hormone levels (GO:0010817)	133	1	0.21	4.78	1.90E-01	1.00E+00	BP	4
sulfur compound biosynthetic process (GO:0044272)	134	1	0.21	4.74	1.92E-01	1.00E+00	BP	4
histone modification (GO:0016570)	143	1	0.23	4.44	2.03E-01	1.00E+00	BP	4
response to other organism (GO:0051707)	437	3	0.69	4.36	3.21E-02	1.00E+00	BP	4
response to external biotic stimulus (GO:0043207)	437	3	0.69	4.36	3.21E-02	1.00E+00	BP	4
non-membrane-bounded organelle assembly (GO:0140694)	149	1	0.23	4.26	2.11E-01	1.00E+00	BP	4
phosphorelay signal transduction system (GO:0000160)	149	1	0.23	4.26	2.11E-01	1.00E+00	BP	4
biological process involved in interspecies interaction between organisms (GO:0044419)	457	3	0.72	4.17	3.59E-02	1.00E+00	BP	4
response to biotic stimulus (GO:0009607)	470	3	0.74	4.06	3.85E-02	1.00E+00	BP	4
hydrogen peroxide catabolic process (GO:0042744)	171	1	0.27	3.72	2.37E-01	1.00E+00	BP	4
defense response (GO:0006952)	515	3	0.81	3.7	4.82E-02	1.00E+00	BP	4
hydrogen peroxide metabolic process (GO:0042743)	172	1	0.27	3.69	2.39E-01	1.00E+00	BP	4
regulation of cellular component organization (GO:0051128)	176	1	0.28	3.61	2.43E-01	1.00E+00	BP	4
protein dephosphorylation (GO:0006470)	186	1	0.29	3.42	2.55E-01	1.00E+00	BP	4
response to external stimulus (GO:0009605)	583	3	0.92	3.27	6.48E-02	1.00E+00	BP	4
organelle assembly (GO:0070925)	195	1	0.31	3.26	2.66E-01	1.00E+00	BP	4
macromolecule methylation (GO:0043414)	198	1	0.31	3.21	2.69E-01	1.00E+00	BP	4
reactive oxygen species metabolic process (GO:0072593)	200	1	0.31	3.18	2.72E-01	1.00E+00	BP	4
response to temperature stimulus (GO:0009266)	202	1	0.32	3.15	2.74E-01	1.00E+00	BP	4
cell cycle process (GO:0022402)	425	2	0.67	2.99	1.45E-01	1.00E+00	BP	4
sulfur compound metabolic process (GO:0006790)	231	1	0.36	2.75	3.06E-01	1.00E+00	BP	4
alpha-amino acid biosynthetic process (GO:1901607)	237	1	0.37	2.68	3.13E-01	1.00E+00	BP	4
peptidyl-lysine modification (GO:0018205)	240	1	0.38	2.65	3.16E-01	1.00E+00	BP	4
hemicellulose metabolic process (GO:0010410)	240	1	0.38	2.65	3.16E-01	1.00E+00	BP	4
regulation of developmental process (GO:0050793)	245	1	0.39	2.59	3.22E-01	1.00E+00	BP	4
dephosphorylation (GO:0016311)	248	1	0.39	2.56	3.25E-01	1.00E+00	BP	4
hormone-mediated signaling pathway (GO:0009755)	497	2	0.78	2.56	1.85E-01	1.00E+00	BP	4
cell wall polysaccharide metabolic process (GO:0010383)	250	1	0.39	2.54	3.27E-01	1.00E+00	BP	4
cellular response to hormone stimulus (GO:0032870)	504	2	0.79	2.52	1.89E-01	1.00E+00	BP	4
cellular response to endogenous stimulus (GO:0071495)	510	2	0.8	2.49	1.92E-01	1.00E+00	BP	4
cell division (GO:0051301)	256	1	0.4	2.48	3.33E-01	1.00E+00	BP	4
cellular oxidant detoxification (GO:0098869)	266	1	0.42	2.39	3.44E-01	1.00E+00	BP	4
reproductive structure development (GO:0048608)	270	1	0.42	2.35	3.48E-01	1.00E+00	BP	4
reproductive system development (GO:0061458)	273	1	0.43	2.33	3.51E-01	1.00E+00	BP	4
cell wall macromolecule metabolic process (GO:0044036)	274	1	0.43	2.32	3.52E-01	1.00E+00	BP	4
amino acid biosynthetic process (GO:0008652)	283	1	0.45	2.24	3.61E-01	1.00E+00	BP	4
cellular response to toxic substance (GO:0097237)	283	1	0.45	2.24	3.61E-01	1.00E+00	BP	4
cellular detoxification (GO:1990748)	283	1	0.45	2.24	3.61E-01	1.00E+00	BP	4
cellular response to organic substance (GO:0071310)	578	2	0.91	2.2	2.31E-01	1.00E+00	BP	4
cell cycle (GO:0007049)	586	2	0.92	2.17	2.36E-01	1.00E+00	BP	4
establishment of protein localization to organelle (GO:0072594)	295	1	0.46	2.15	3.73E-01	1.00E+00	BP	4
developmental process involved in reproduction (GO:0003006)	307	1	0.48	2.07	3.85E-01	1.00E+00	BP	4
cellular response to chemical stimulus (GO:0070887)	934	3	1.47	2.04	1.83E-01	1.00E+00	BP	4
signal transduction (GO:0007165)	1258	4	1.98	2.02	1.37E-01	1.00E+00	BP	4
response to oxidative stress (GO:0006979)	319	1	0.5	1.99	3.97E-01	1.00E+00	BP	4
signaling (GO:0023052)	1277	4	2.01	1.99	1.42E-01	1.00E+00	BP	4
developmental process (GO:0032502)	965	3	1.52	1.98	1.95E-01	1.00E+00	BP	4
cell communication (GO:0007154)	1326	4	2.09	1.92	1.56E-01	1.00E+00	BP	4
detoxification (GO:0098754)	334	1	0.53	1.9	4.11E-01	1.00E+00	BP	4
chromatin remodeling (GO:0006338)	346	1	0.54	1.84	4.22E-01	1.00E+00	BP	4
protein localization to organelle (GO:0033365)	346	1	0.54	1.84	4.22E-01	1.00E+00	BP	4

GO Term	Genome	Count	Expected	Fold Enrichment	P-value	FDR	Term	Cluster
post-embryonic development (GO:0009791)	348	1	0.55	1.83	4.24E-01	1.00E+00	BP	4
response to toxic substance (GO:0009636)	358	1	0.56	1.77	4.33E-01	1.00E+00	BP	4
protein folding (GO:0006457)	368	1	0.58	1.73	4.42E-01	1.00E+00	BP	4
alpha-amino acid metabolic process (GO:1901605)	370	1	0.58	1.72	4.44E-01	1.00E+00	BP	4
positive regulation of DNA-templated transcription (GO:0045893)	376	1	0.59	1.69	4.49E-01	1.00E+00	BP	4
positive regulation of RNA biosynthetic process (GO:1902680)	376	1	0.59	1.69	4.49E-01	1.00E+00	BP	4
response to hormone (GO:0009725)	756	2	1.19	1.68	3.35E-01	1.00E+00	BP	4
response to endogenous stimulus (GO:0009719)	762	2	1.2	1.67	3.38E-01	1.00E+00	BP	4
regulation of RNA biosynthetic process (GO:2001141)	2705	7	4.26	1.64	1.99E-01	1.00E+00	BP	4
regulation of DNA-templated transcription (GO:0006355)	2705	7	4.26	1.64	1.99E-01	1.00E+00	BP	4
cellular component assembly (GO:0022607)	774	2	1.22	1.64	3.45E-01	1.00E+00	BP	4
positive regulation of macromolecule biosynthetic process (GO:0010557)	394	1	0.62	1.61	4.64E-01	1.00E+00	BP	4
positive regulation of cellular biosynthetic process (GO:0031328)	399	1	0.63	1.59	4.69E-01	1.00E+00	BP	4
positive regulation of biosynthetic process (GO:0009891)	403	1	0.63	1.58	4.72E-01	1.00E+00	BP	4
regulation of RNA metabolic process (GO:0051252)	2843	7	4.48	1.56	2.14E-01	1.00E+00	BP	4
regulation of nucleobase-containing compound metabolic process (GO:0019219)	2913	7	4.59	1.53	2.24E-01	1.00E+00	BP	4
chromatin organization (GO:0006325)	417	1	0.66	1.52	4.84E-01	1.00E+00	BP	4
regulation of macromolecule biosynthetic process (GO:0010556)	2942	7	4.63	1.51	2.28E-01	1.00E+00	BP	4
regulation of cellular biosynthetic process (GO:0031326)	2959	7	4.66	1.5	2.30E-01	1.00E+00	BP	4
regulation of biosynthetic process (GO:0009889)	2969	7	4.67	1.5	2.32E-01	1.00E+00	BP	4
regulation of biological quality (GO:0065008)	433	1	0.68	1.47	4.97E-01	1.00E+00	BP	4
intracellular transport (GO:0046907)	896	2	1.41	1.42	6.53E-01	1.00E+00	BP	4
RNA modification (GO:0009451)	453	1	0.71	1.4	5.12E-01	1.00E+00	BP	4
response to stress (GO:0006950)	2296	5	3.61	1.38	4.11E-01	1.00E+00	BP	4
regulation of cellular process (GO:0050794)	4625	10	7.28	1.37	3.19E-01	1.00E+00	BP	4
intracellular signal transduction (GO:0035556)	463	1	0.73	1.37	5.20E-01	1.00E+00	BP	4
establishment of localization in cell (GO:0051649)	932	2	1.47	1.36	6.59E-01	1.00E+00	BP	4
regulation of cellular metabolic process (GO:0031323)	3270	7	5.15	1.36	3.56E-01	1.00E+00	BP	4
positive regulation of RNA metabolic process (GO:0051254)	469	1	0.74	1.35	5.25E-01	1.00E+00	BP	4
regulation of gene expression (GO:0010468)	3303	7	5.2	1.35	3.61E-01	1.00E+00	BP	4
regulation of nitrogen compound metabolic process (GO:0051171)	3361	7	5.29	1.32	3.68E-01	1.00E+00	BP	4
protein-DNA complex organization (GO:0071824)	481	1	0.76	1.32	5.34E-01	1.00E+00	BP	4
system development (GO:0048731)	482	1	0.76	1.32	5.34E-01	1.00E+00	BP	4
regulation of primary metabolic process (GO:0080090)	3400	7	5.35	1.31	4.92E-01	1.00E+00	BP	4
positive regulation of nucleobase-containing compound metabolic process (GO:0045935)	488	1	0.77	1.3	5.39E-01	1.00E+00	BP	4
response to stimulus (GO:0050896)	4415	9	6.95	1.3	4.17E-01	1.00E+00	BP	4
response to organic substance (GO:0010033)	1000	2	1.57	1.27	6.72E-01	1.00E+00	BP	4
reproductive process (GO:0022414)	503	1	0.79	1.26	5.50E-01	1.00E+00	BP	4
response to chemical (GO:0042221)	1513	3	2.38	1.26	5.16E-01	1.00E+00	BP	4
protein-containing complex organization (GO:0043933)	1011	2	1.59	1.26	6.74E-01	1.00E+00	BP	4
biological regulation (GO:0065007)	5626	11	8.86	1.24	4.65E-01	1.00E+00	BP	4
organic acid biosynthetic process (GO:0016053)	521	1	0.82	1.22	5.62E-01	1.00E+00	BP	4
carboxylic acid biosynthetic process (GO:0046394)	521	1	0.82	1.22	5.62E-01	1.00E+00	BP	4
regulation of macromolecule metabolic process (GO:0060255)	3684	7	5.8	1.21	5.16E-01	1.00E+00	BP	4
regulation of biological process (GO:0050789)	5297	10	8.34	1.2	5.74E-01	1.00E+00	BP	4
positive regulation of cellular metabolic process (GO:0031325)	535	1	0.84	1.19	5.72E-01	1.00E+00	BP	4
regulation of metabolic process (GO:0019222)	3766	7	5.93	1.18	6.63E-01	1.00E+00	BP	4
methylation (GO:0032259)	541	1	0.85	1.17	5.76E-01	1.00E+00	BP	4
cellular response to stimulus (GO:0051716)	2710	5	4.27	1.17	6.16E-01	1.00E+00	BP	4
peptidyl-amino acid modification (GO:0018193)	544	1	0.86	1.17	5.78E-01	1.00E+00	BP	4
protein-containing complex assembly (GO:0065003)	554	1	0.87	1.15	5.85E-01	1.00E+00	BP	4

GO Term	Genome	Count	Expected	Fold Enrichment	P-value	FDR	Term	Cluster
amino acid metabolic process (GO:0006520)	555	1	0.87	1.14	5.86E-01	1.00E+00	BP	4
polysaccharide metabolic process (GO:0005976)	562	1	0.88	1.13	5.90E-01	1.00E+00	BP	4
reproduction (GO:0000003)	562	1	0.88	1.13	5.90E-01	1.00E+00	BP	4
organelle organization (GO:0006996)	1700	3	2.68	1.12	7.50E-01	1.00E+00	BP	4
protein ubiquitination (GO:0016567)	573	1	0.9	1.11	5.97E-01	1.00E+00	BP	4
Unclassified (UNCLASSIFIED)	17419	30	27.42	1.09	5.24E-01	1.00E+00	BP	4
cell wall organization or biogenesis (GO:0071554)	586	1	0.92	1.08	6.06E-01	1.00E+00	BP	4
positive regulation of nitrogen compound metabolic process (GO:0051173)	593	1	0.93	1.07	6.10E-01	1.00E+00	BP	4
positive regulation of cellular process (GO:0048522)	597	1	0.94	1.06	6.12E-01	1.00E+00	BP	4
positive regulation of macromolecule metabolic process (GO:0010604)	603	1	0.95	1.05	6.16E-01	1.00E+00	BP	4
response to abiotic stimulus (GO:0009628)	611	1	0.96	1.04	6.21E-01	1.00E+00	BP	4
intracellular protein transport (GO:0006886)	617	1	0.97	1.03	6.25E-01	1.00E+00	BP	4
protein modification by small protein conjugation (GO:0032446)	618	1	0.97	1.03	6.25E-01	1.00E+00	BP	4
positive regulation of metabolic process (GO:0009893)	620	1	0.98	1.02	6.26E-01	1.00E+00	BP	4
macromolecule modification (GO:0043412)	3738	6	5.88	1.02	8.30E-01	1.00E+00	BP	4
multicellular organism development (GO:0007275)	627	1	0.99	1.01	1.00E+00	1.00E+00	BP	4
cellular localization (GO:0051641)	1297	2	2.04	0.98	1.00E+00	1.00E+00	BP	4
regulation of transcription by RNA polymerase II (GO:0006357)	659	1	1.04	0.96	1.00E+00	1.00E+00	BP	4
multicellular organismal process (GO:0032501)	686	1	1.08	0.93	1.00E+00	1.00E+00	BP	4
biological_process (GO:0008150)	21970	32	34.58	0.93	5.24E-01	1.00E+00	BP	4
cellular component biogenesis (GO:0044085)	1401	2	2.21	0.91	1.00E+00	1.00E+00	BP	4
small molecule biosynthetic process (GO:0044283)	708	1	1.11	0.9	1.00E+00	1.00E+00	BP	4
cellular process (GO:0009987)	15372	21	24.2	0.87	4.37E-01	1.00E+00	BP	4
cellular component organization (GO:0016043)	2935	4	4.62	0.87	1.00E+00	1.00E+00	BP	4
positive regulation of biological process (GO:0048518)	749	1	1.18	0.85	1.00E+00	1.00E+00	BP	4
cellular catabolic process (GO:0044248)	783	1	1.23	0.81	1.00E+00	1.00E+00	BP	4
protein modification process (GO:0036211)	3194	4	5.03	0.8	8.17E-01	1.00E+00	BP	4
DNA-templated transcription (GO:0006351)	824	1	1.3	0.77	1.00E+00	1.00E+00	BP	4
anatomical structure development (GO:0048856)	825	1	1.3	0.77	1.00E+00	1.00E+00	BP	4
RNA biosynthetic process (GO:0032774)	840	1	1.32	0.76	1.00E+00	1.00E+00	BP	4
protein modification by small protein conjugation or removal (GO:0070647)	847	1	1.33	0.75	1.00E+00	1.00E+00	BP	4
cellular component organization or biogenesis (GO:0071840)	3437	4	5.41	0.74	6.57E-01	1.00E+00	BP	4
protein transport (GO:0015031)	950	1	1.5	0.67	1.00E+00	1.00E+00	BP	4
establishment of protein localization (GO:0045184)	962	1	1.51	0.66	1.00E+00	1.00E+00	BP	4
organic substance metabolic process (GO:0071704)	12450	12	19.6	0.61	4.00E-02	1.00E+00	BP	4
protein localization (GO:0008104)	1049	1	1.65	0.61	1.00E+00	1.00E+00	BP	4
cellular macromolecule localization (GO:0070727)	1050	1	1.65	0.61	1.00E+00	1.00E+00	BP	4
transport (GO:0006810)	3186	3	5.01	0.6	4.84E-01	1.00E+00	BP	4
organonitrogen compound metabolic process (GO:1901564)	6443	6	10.14	0.59	1.72E-01	1.00E+00	BP	4
establishment of localization (GO:0051234)	3225	3	5.08	0.59	4.85E-01	1.00E+00	BP	4
localization (GO:0051179)	3340	3	5.26	0.57	4.89E-01	1.00E+00	BP	4
carboxylic acid metabolic process (GO:0019752)	1117	1	1.76	0.57	1.00E+00	1.00E+00	BP	4
oxoacid metabolic process (GO:0043436)	1130	1	1.78	0.56	1.00E+00	1.00E+00	BP	4
organic acid metabolic process (GO:0006082)	1131	1	1.78	0.56	1.00E+00	1.00E+00	BP	4
metabolic process (GO:0008152)	13613	12	21.43	0.56	1.09E-02	1.00E+00	BP	4
RNA metabolic process (GO:0016070)	2384	2	3.75	0.53	5.89E-01	1.00E+00	BP	4
nitrogen compound metabolic process (GO:0006807)	9609	8	15.12	0.53	3.74E-02	1.00E+00	BP	4
macromolecule localization (GO:0033036)	1264	1	1.99	0.5	7.25E-01	1.00E+00	BP	4
nucleobase-containing compound biosynthetic process (GO:0034654)	1266	1	1.99	0.5	7.25E-01	1.00E+00	BP	4
macromolecule metabolic process (GO:0043170)	8871	7	13.96	0.5	3.29E-02	1.00E+00	BP	4
protein metabolic process (GO:0019538)	5254	4	8.27	0.48	1.34E-01	1.00E+00	BP	4

GO Term	Genome	Count	Expected	Fold Enrichment	P-value	FDR	Term	Cluster
carbohydrate metabolic process (GO:0005975)	1347	1	2.12	0.47	7.26E-01	1.00E+00	BP	4
cellular metabolic process (GO:0044237)	9471	7	14.91	0.47	1.68E-02	1.00E+00	BP	4
nitrogen compound transport (GO:0071705)	1381	1	2.17	0.46	7.27E-01	1.00E+00	BP	4
heterocycle metabolic process (GO:0046483)	4164	3	6.55	0.46	2.10E-01	1.00E+00	BP	4
primary metabolic process (GO:0044238)	11484	8	18.08	0.44	4.63E-03	1.00E+00	BP	4
cellular aromatic compound metabolic process (GO:0006725)	4327	3	6.81	0.44	1.53E-01	1.00E+00	BP	4
heterocycle biosynthetic process (GO:0018130)	1487	1	2.34	0.43	7.32E-01	1.00E+00	BP	4
phosphate-containing compound metabolic process (GO:0006796)	2984	2	4.7	0.43	3.29E-01	1.00E+00	BP	4
phosphorus metabolic process (GO:0006793)	3030	2	4.77	0.42	2.37E-01	1.00E+00	BP	4
protein phosphorylation (GO:0006468)	1543	1	2.43	0.41	5.21E-01	1.00E+00	BP	4
aromatic compound biosynthetic process (GO:0019438)	1547	1	2.44	0.41	5.21E-01	1.00E+00	BP	4
organic cyclic compound biosynthetic process (GO:1901362)	1677	1	2.64	0.38	5.24E-01	1.00E+00	BP	4
nucleic acid metabolic process (GO:0090304)	3395	2	5.34	0.37	1.72E-01	1.00E+00	BP	4
transmembrane transport (GO:0055085)	1724	1	2.71	0.37	5.26E-01	1.00E+00	BP	4
cellular biosynthetic process (GO:0044249)	3461	2	5.45	0.37	1.73E-01	1.00E+00	BP	4
organic substance transport (GO:0071702)	1759	1	2.77	0.36	5.29E-01	1.00E+00	BP	4
organic substance catabolic process (GO:1901575)	1863	1	2.93	0.34	3.71E-01	1.00E+00	BP	4
small molecule metabolic process (GO:0044281)	1894	1	2.98	0.34	3.72E-01	1.00E+00	BP	4
nucleobase-containing compound metabolic process (GO:0006139)	3868	2	6.09	0.33	8.73E-02	1.00E+00	BP	4
catabolic process (GO:0009056)	1947	1	3.06	0.33	3.73E-01	1.00E+00	BP	4
organonitrogen compound biosynthetic process (GO:1901566)	2013	1	3.17	0.32	3.77E-01	1.00E+00	BP	4
organic substance biosynthetic process (GO:1901576)	4099	2	6.45	0.31	6.16E-02	1.00E+00	BP	4
biosynthetic process (GO:0009058)	4259	2	6.7	0.3	6.20E-02	1.00E+00	BP	4
phosphorylation (GO:0016310)	2164	1	3.41	0.29	2.63E-01	1.00E+00	BP	4
organic cyclic compound metabolic process (GO:1901360)	4430	2	6.97	0.29	4.29E-02	1.00E+00	BP	4
cellular nitrogen compound biosynthetic process (GO:0044271)	2423	1	3.81	0.26	1.84E-01	1.00E+00	BP	4
macromolecule biosynthetic process (GO:0009059)	2436	1	3.83	0.26	1.84E-01	1.00E+00	BP	4
cellular nitrogen compound metabolic process (GO:0034641)	4908	2	7.73	0.26	2.05E-02	1.00E+00	BP	4
acireductone synthase activity (GO:0043874)	2	1	0	> 100	4.71E-03	1.00E+00	MF	4
growth factor activity (GO:0008083)	7	1	0.01	90.76	1.25E-02	1.00E+00	MF	4
signaling receptor activator activity (GO:0030546)	10	1	0.02	63.53	1.72E-02	1.00E+00	MF	4
signaling receptor regulator activity (GO:0030545)	10	1	0.02	63.53	1.72E-02	1.00E+00	MF	4
dynein intermediate chain binding (GO:0045505)	10	1	0.02	63.53	1.72E-02	1.00E+00	MF	4
receptor ligand activity (GO:0048018)	10	1	0.02	63.53	1.72E-02	1.00E+00	MF	4
cytokinin dehydrogenase activity (GO:0019139)	17	1	0.03	37.37	2.79E-02	1.00E+00	MF	4
nuclear import signal receptor activity (GO:0061608)	20	1	0.03	31.77	3.25E-02	1.00E+00	MF	4
nucleocytoplasmic carrier activity (GO:0140142)	27	1	0.04	23.53	4.31E-02	1.00E+00	MF	4
minor groove of adenine-thymine-rich DNA binding (GO:0003680)	39	1	0.06	16.29	6.10E-02	1.00E+00	MF	4
signaling receptor binding (GO:0005102)	39	1	0.06	16.29	6.10E-02	1.00E+00	MF	4
histone methyltransferase activity (GO:0042054)	42	1	0.07	15.13	6.54E-02	1.00E+00	MF	4
DNA secondary structure binding (GO:0000217)	44	1	0.07	14.44	6.84E-02	1.00E+00	MF	4
oxidoreductase activity, acting on the CH-NH group of donors (GO:0016645)	55	1	0.09	11.55	8.44E-02	1.00E+00	MF	4
xylan O-acetyltransferase activity (GO:1990538)	61	1	0.1	10.41	9.30E-02	1.00E+00	MF	4
ADP binding (GO:0043531)	134	2	0.21	9.48	1.95E-02	1.00E+00	MF	4
molecular carrier activity (GO:0140104)	71	1	0.11	8.95	1.07E-01	1.00E+00	MF	4
O-acetyltransferase activity (GO:0016413)	75	1	0.12	8.47	1.13E-01	1.00E+00	MF	4
tubulin binding (GO:0015631)	259	3	0.41	7.36	8.21E-03	1.00E+00	MF	4
polysaccharide binding (GO:0030247)	87	1	0.14	7.3	1.29E-01	1.00E+00	MF	4
protein methyltransferase activity (GO:0008276)	88	1	0.14	7.22	1.31E-01	1.00E+00	MF	4
FAD binding (GO:0071949)	89	1	0.14	7.14	1.32E-01	1.00E+00	MF	4
histone modifying activity (GO:0140993)	119	1	0.19	5.34	1.72E-01	1.00E+00	MF	4

GO Term	Genome	Count	Expected	Fold Enrichment	P-value	FDR	Term	Cluster
microtubule binding (GO:0008017)	248	2	0.39	5.12	5.90E-02	1.00E+00	MF	4
lactoperoxidase activity (GO:0140825)	145	1	0.23	4.38	2.06E-01	1.00E+00	MF	4
cytoskeletal protein binding (GO:0008092)	440	3	0.69	4.33	3.27E-02	1.00E+00	MF	4
O-acyltransferase activity (GO:0008374)	147	1	0.23	4.32	2.08E-01	1.00E+00	MF	4
acetyltransferase activity (GO:0016407)	148	1	0.23	4.29	2.09E-01	1.00E+00	MF	4
carbohydrate binding (GO:0030246)	314	2	0.49	4.05	8.84E-02	1.00E+00	MF	4
magnesium ion binding (GO:0000287)	178	1	0.28	3.57	2.46E-01	1.00E+00	MF	4
heme binding (GO:0020037)	565	3	0.89	3.37	6.02E-02	1.00E+00	MF	4
tetrapyrrole binding (GO:0046906)	609	3	0.96	3.13	7.18E-02	1.00E+00	MF	4
molecular function activator activity (GO:0140677)	203	1	0.32	3.13	2.75E-01	1.00E+00	MF	4
monooxygenase activity (GO:0004497)	441	2	0.69	2.88	1.54E-01	1.00E+00	MF	4
oxidoreductase activity, acting on peroxide as acceptor (GO:0016684)	224	1	0.35	2.84	2.99E-01	1.00E+00	MF	4
peroxidase activity (GO:0004601)	224	1	0.35	2.84	2.99E-01	1.00E+00	MF	4
flavin adenine dinucleotide binding (GO:0050660)	230	1	0.36	2.76	3.05E-01	1.00E+00	MF	4
iron ion binding (GO:0005506)	465	2	0.73	2.73	1.67E-01	1.00E+00	MF	4
double-stranded DNA binding (GO:0003690)	723	3	1.14	2.64	1.06E-01	1.00E+00	MF	4
myosin phosphatase activity (GO:0017018)	243	1	0.38	2.61	3.20E-01	1.00E+00	MF	4
protein serine/threonine phosphatase activity (GO:0004722)	249	1	0.39	2.55	3.26E-01	1.00E+00	MF	4
phosphatase activity (GO:0016791)	505	2	0.79	2.52	1.90E-01	1.00E+00	MF	4
oxidoreductase activity, acting on paired donors, with incorporation or reduction of molecular oxygen (GO:0016705)	513	2	0.81	2.48	1.94E-01	1.00E+00	MF	4
antioxidant activity (GO:0016209)	261	1	0.41	2.43	3.39E-01	1.00E+00	MF	4
acyltransferase activity, transferring groups other than amino-acyl groups (GO:0016747)	531	2	0.84	2.39	2.04E-01	1.00E+00	MF	4
phosphoric ester hydrolase activity (GO:0042578)	576	2	0.91	2.21	2.30E-01	1.00E+00	MF	4
phosphoprotein phosphatase activity (GO:0004721)	294	1	0.46	2.16	3.72E-01	1.00E+00	MF	4
UDP-glucosyltransferase activity (GO:0035251)	298	1	0.47	2.13	3.76E-01	1.00E+00	MF	4
sequence-specific double-stranded DNA binding (GO:1990837)	608	2	0.96	2.09	2.49E-01	1.00E+00	MF	4
glucosyltransferase activity (GO:0046527)	340	1	0.54	1.87	4.16E-01	1.00E+00	MF	4
acyltransferase activity (GO:0016746)	1155	3	1.82	1.65	4.31E-01	1.00E+00	MF	4
UDP-glycosyltransferase activity (GO:0008194)	428	1	0.67	1.48	4.93E-01	1.00E+00	MF	4
transition metal ion binding (GO:0046914)	1290	3	2.03	1.48	4.59E-01	1.00E+00	MF	4
protein binding (GO:0005515)	2323	5	3.66	1.37	4.15E-01	1.00E+00	MF	4
Unclassified (UNCLASSIFIED)	16329	33	25.7	1.28	7.05E-02	1.00E+00	MF	4
oxidoreductase activity (GO:0016491)	2048	4	3.22	1.24	5.65E-01	1.00E+00	MF	4
DNA-binding transcription factor activity (GO:0003700)	1586	3	2.5	1.2	7.40E-01	1.00E+00	MF	4
DNA binding (GO:0003677)	3182	6	5.01	1.2	6.37E-01	1.00E+00	MF	4
methyltransferase activity (GO:0008168)	532	1	0.84	1.19	5.70E-01	1.00E+00	MF	4
metal ion binding (GO:0046872)	3774	7	5.94	1.18	6.63E-01	1.00E+00	MF	4
cation binding (GO:0043169)	3802	7	5.98	1.17	6.64E-01	1.00E+00	MF	4
transferase activity, transferring one-carbon groups (GO:0016741)	549	1	0.86	1.16	5.82E-01	1.00E+00	MF	4
ubiquitin-protein transferase activity (GO:0004842)	558	1	0.88	1.14	5.87E-01	1.00E+00	MF	4
ubiquitin-like protein transferase activity (GO:0019787)	578	1	0.91	1.1	6.00E-01	1.00E+00	MF	4
aminoacyltransferase activity (GO:0016755)	583	1	0.92	1.09	6.04E-01	1.00E+00	MF	4
transcription regulator activity (GO:0140110)	1753	3	2.76	1.09	7.56E-01	1.00E+00	MF	4
sequence-specific DNA binding (GO:0043565)	1176	2	1.85	1.08	7.09E-01	1.00E+00	MF	4
transcription regulatory region nucleic acid binding (GO:0001067)	589	1	0.93	1.08	6.07E-01	1.00E+00	MF	4
transcription cis-regulatory region binding (GO:0000976)	589	1	0.93	1.08	6.07E-01	1.00E+00	MF	4
binding (GO:0005488)	14290	23	22.49	1.02	8.95E-01	1.00E+00	MF	4
hydrolase activity, acting on ester bonds (GO:0016788)	1254	2	1.97	1.01	1.00E+00	1.00E+00	MF	4
hexosyltransferase activity (GO:0016758)	637	1	1	1	1.00E+00	1.00E+00	MF	4
zinc ion binding (GO:0008270)	651	1	1.02	0.98	1.00E+00	1.00E+00	MF	4

GO Term	Genome	Count	Expected	Fold Enrichment	P-value	FDR	Term	Cluster
molecular function regulator activity (GO:0098772)	652	1	1.03	0.97	1.00E+00	1.00E+00	MF	4
ion binding (GO:0043167)	7841	11	12.34	0.89	7.52E-01	1.00E+00	MF	4
heterocyclic compound binding (GO:1901363)	10349	14	16.29	0.86	5.66E-01	1.00E+00	MF	4
organic cyclic compound binding (GO:0097159)	10373	14	16.33	0.86	5.66E-01	1.00E+00	MF	4
molecular_function (GO:0003674)	23060	29	36.3	0.8	7.05E-02	1.00E+00	MF	4
nucleic acid binding (GO:0003676)	5685	7	8.95	0.78	5.89E-01	1.00E+00	MF	4
transferase activity (GO:0016740)	5764	6	9.07	0.66	3.67E-01	1.00E+00	MF	4
glycosyltransferase activity (GO:0016757)	972	1	1.53	0.65	1.00E+00	1.00E+00	MF	4
catalytic activity, acting on a protein (GO:0140096)	3926	4	6.18	0.65	5.22E-01	1.00E+00	MF	4
catalytic activity (GO:0003824)	13387	13	21.07	0.62	3.15E-02	1.00E+00	MF	4
nucleotide binding (GO:0000166)	4340	4	6.83	0.59	3.13E-01	1.00E+00	MF	4
nucleoside phosphate binding (GO:1901265)	4340	4	6.83	0.59	3.13E-01	1.00E+00	MF	4
anion binding (GO:0043168)	4484	4	7.06	0.57	3.14E-01	1.00E+00	MF	4
small molecule binding (GO:0036094)	4599	4	7.24	0.55	2.39E-01	1.00E+00	MF	4
adenyl ribonucleotide binding (GO:0032559)	3497	3	5.5	0.55	3.70E-01	1.00E+00	MF	4
adenyl nucleotide binding (GO:0030554)	3702	3	5.83	0.51	2.78E-01	1.00E+00	MF	4
purine ribonucleotide binding (GO:0032555)	3854	3	6.07	0.49	2.81E-01	1.00E+00	MF	4
ribonucleotide binding (GO:0032553)	3896	3	6.13	0.49	2.82E-01	1.00E+00	MF	4
carbohydrate derivative binding (GO:0097367)	3928	3	6.18	0.49	2.83E-01	1.00E+00	MF	4
purine nucleotide binding (GO:0017076)	4059	3	6.39	0.47	2.08E-01	1.00E+00	MF	4
protein kinase activity (GO:0004672)	1604	1	2.52	0.4	5.21E-01	1.00E+00	MF	4
transmembrane transporter activity (GO:0022857)	1612	1	2.54	0.39	5.21E-01	1.00E+00	MF	4
transporter activity (GO:0005215)	1678	1	2.64	0.38	5.24E-01	1.00E+00	MF	4
phosphotransferase activity, alcohol group as acceptor (GO:0016773)	1823	1	2.87	0.35	3.71E-01	1.00E+00	MF	4
RNA binding (GO:0003723)	2087	1	3.29	0.3	2.62E-01	1.00E+00	MF	4
kinase activity (GO:0016301)	2226	1	3.5	0.29	2.65E-01	1.00E+00	MF	4
hydrolase activity (GO:0016787)	4566	2	7.19	0.28	4.37E-02	1.00E+00	MF	4
transferase activity, transferring phosphorus-containing groups (GO:0016772)	2537	1	3.99	0.25	1.87E-01	1.00E+00	MF	4
ATP binding (GO:0005524)	3362	1	5.29	0.19	6.30E-02	1.00E+00	MF	4
purine ribonucleoside triphosphate binding (GO:0035639)	3719	1	5.85	0.17	2.81E-02	1.00E+00	MF	4
Ndc80 complex (GO:0031262)	5	1	0.01	> 100	9.39E-03	1.00E+00	CC	4
nuclear pore central transport channel (GO:0044613)	8	1	0.01	79.41	1.41E-02	1.00E+00	CC	4
outer kinetochore (GO:0000940)	10	1	0.02	63.53	1.72E-02	1.00E+00	CC	4
dynein complex (GO:0030286)	10	1	0.02	63.53	1.72E-02	1.00E+00	CC	4
cytoplasmic dynein complex (GO:0005868)	10	1	0.02	63.53	1.72E-02	1.00E+00	CC	4
nuclear microtubule (GO:0005880)	21	1	0.03	30.25	3.40E-02	1.00E+00	CC	4
cytoplasmic microtubule (GO:0005881)	27	1	0.04	23.53	4.31E-02	1.00E+00	CC	4
kinetochore (GO:0000776)	36	1	0.06	17.65	5.66E-02	1.00E+00	CC	4
condensed chromosome, centromeric region (GO:0000779)	38	1	0.06	16.72	5.95E-02	1.00E+00	CC	4
chromosome, centromeric region (GO:0000775)	41	1	0.06	15.5	6.40E-02	1.00E+00	CC	4
nuclear pore (GO:0005643)	65	1	0.1	9.77	9.87E-02	1.00E+00	CC	4
microtubule associated complex (GO:0005875)	66	1	0.1	9.63	1.00E-01	1.00E+00	CC	4
chromosomal region (GO:0098687)	67	1	0.11	9.48	1.02E-01	1.00E+00	CC	4
condensed chromosome (GO:0000793)	71	1	0.11	8.95	1.07E-01	1.00E+00	CC	4
microtubule (GO:0005874)	259	3	0.41	7.36	8.21E-03	1.00E+00	CC	4
polymeric cytoskeletal fiber (GO:0099513)	267	3	0.42	7.14	8.91E-03	1.00E+00	CC	4
supramolecular polymer (GO:0099081)	268	3	0.42	7.11	9.00E-03	1.00E+00	CC	4
supramolecular fiber (GO:0099512)	268	3	0.42	7.11	9.00E-03	1.00E+00	CC	4
plant-type cell wall (GO:0009505)	94	1	0.15	6.76	1.39E-01	1.00E+00	CC	4
supramolecular complex (GO:0099080)	387	4	0.61	6.57	3.37E-03	1.00E+00	CC	4
microtubule cytoskeleton (GO:0015630)	304	3	0.48	6.27	1.26E-02	1.00E+00	CC	4

GO Term	Genome	Count	Expected	Fold Enrichment	P-value	FDR	Term	Cluster
nuclear envelope (GO:0005635)	103	1	0.16	6.17	1.51E-01	1.00E+00	CC	4
cell wall (GO:0005618)	112	1	0.18	5.67	1.63E-01	1.00E+00	CC	4
external encapsulating structure (GO:0030312)	116	1	0.18	5.48	1.68E-01	1.00E+00	CC	4
extracellular space (GO:0005615)	132	1	0.21	4.81	1.89E-01	1.00E+00	CC	4
cytoskeleton (GO:0005856)	478	3	0.75	3.99	4.01E-02	1.00E+00	CC	4
extracellular region (GO:0005576)	698	3	1.1	2.73	9.81E-02	1.00E+00	CC	4
chromosome (GO:0005694)	570	2	0.9	2.23	2.27E-01	1.00E+00	CC	4
Golgi membrane (GO:0000139)	394	1	0.62	1.61	4.64E-01	1.00E+00	CC	4
intracellular non-membrane-bounded organelle (GO:0043232)	2611	5	4.11	1.22	6.06E-01	1.00E+00	CC	4
non-membrane-bounded organelle (GO:0043228)	2611	5	4.11	1.22	6.06E-01	1.00E+00	CC	4
nucleus (GO:0005634)	6118	11	9.63	1.14	5.99E-01	1.00E+00	CC	4
Unclassified (UNCLASSIFIED)	19561	35	30.79	1.14	3.11E-01	1.00E+00	CC	4
cellular anatomical entity (GO:0110165)	19592	27	30.84	0.88	3.74E-01	1.00E+00	CC	4
cellular_component (GO:0005575)	19828	27	31.21	0.87	3.11E-01	1.00E+00	CC	4
cell periphery (GO:0071944)	1669	2	2.63	0.76	1.00E+00	1.00E+00	CC	4
envelope (GO:0031975)	844	1	1.33	0.75	1.00E+00	1.00E+00	CC	4
organelle envelope (GO:0031967)	844	1	1.33	0.75	1.00E+00	1.00E+00	CC	4
intracellular organelle (GO:0043229)	11979	14	18.86	0.74	2.14E-01	1.00E+00	CC	4
organelle (GO:0043226)	11985	14	18.86	0.74	2.14E-01	1.00E+00	CC	4
intracellular anatomical structure (GO:0005622)	14114	16	22.22	0.72	1.12E-01	1.00E+00	CC	4
membrane (GO:0016020)	8074	9	12.71	0.71	2.74E-01	1.00E+00	CC	4
intracellular membrane-bounded organelle (GO:0043231)	10826	12	17.04	0.7	1.99E-01	1.00E+00	CC	4
membrane-bounded organelle (GO:0043227)	10848	12	17.08	0.7	1.99E-01	1.00E+00	CC	4
Golgi apparatus (GO:0005794)	959	1	1.51	0.66	1.00E+00	1.00E+00	CC	4
nuclear protein-containing complex (GO:0140513)	991	1	1.56	0.64	1.00E+00	1.00E+00	CC	4
bounding membrane of organelle (GO:0098588)	1033	1	1.63	0.62	1.00E+00	1.00E+00	CC	4
endomembrane system (GO:0012505)	2115	2	3.33	0.6	7.74E-01	1.00E+00	CC	4
cytoplasm (GO:0005737)	8535	8	13.43	0.6	1.21E-01	1.00E+00	CC	4
protein-containing complex (GO:0032991)	4069	3	6.4	0.47	2.08E-01	1.00E+00	CC	4
cytosol (GO:0005829)	1383	1	2.18	0.46	7.27E-01	1.00E+00	CC	4
plasma membrane (GO:0005886)	1449	1	2.28	0.44	7.30E-01	1.00E+00	CC	4
catalytic complex (GO:1902494)	1505	1	2.37	0.42	7.33E-01	1.00E+00	CC	4
organelle membrane (GO:0031090)	1851	1	2.91	0.34	3.71E-01	1.00E+00	CC	4
box H/ACA RNA processing (GO:0034964)	3	1	0.01	> 100	7.58E-03	1.00E+00	BP	5
box H/ACA RNA 3'-end processing (GO:0000495)	3	1	0.01	> 100	7.58E-03	1.00E+00	BP	5
tRNA-guanine transglycosylation (GO:0101030)	3	1	0.01	> 100	7.58E-03	1.00E+00	BP	5
RNA 3' uridylation (GO:0071076)	3	1	0.01	> 100	7.58E-03	1.00E+00	BP	5
box H/ACA RNA metabolic process (GO:0033979)	3	1	0.01	> 100	7.58E-03	1.00E+00	BP	5
phytoalexin biosynthetic process (GO:0052315)	4	1	0.01	> 100	9.47E-03	1.00E+00	BP	5
phytoalexin metabolic process (GO:0052314)	4	1	0.01	> 100	9.47E-03	1.00E+00	BP	5
diterpene phytoalexin biosynthetic process (GO:0051502)	4	1	0.01	> 100	9.47E-03	1.00E+00	BP	5
diterpene phytoalexin metabolic process (GO:0051501)	4	1	0.01	> 100	9.47E-03	1.00E+00	BP	5
toxin metabolic process (GO:0009404)	4	1	0.01	> 100	9.47E-03	1.00E+00	BP	5
toxin biosynthetic process (GO:0009403)	4	1	0.01	> 100	9.47E-03	1.00E+00	BP	5
snRNA pseudouridine synthesis (GO:0031120)	6	1	0.01	87.53	1.32E-02	1.00E+00	BP	5
snRNA modification (GO:0040031)	8	1	0.02	65.65	1.70E-02	1.00E+00	BP	5
mRNA pseudouridine synthesis (GO:1990481)	10	1	0.02	52.52	2.07E-02	1.00E+00	BP	5
regulation of salicylic acid biosynthetic process (GO:0080142)	13	1	0.02	40.4	2.63E-02	1.00E+00	BP	5
regulation of salicylic acid metabolic process (GO:0010337)	13	1	0.02	40.4	2.63E-02	1.00E+00	BP	5
rRNA pseudouridine synthesis (GO:0031118)	17	1	0.03	30.89	3.37E-02	1.00E+00	BP	5
sno(s)RNA 3'-end processing (GO:0031126)	21	1	0.04	25.01	4.10E-02	1.00E+00	BP	5

GO Term	Genome	Count	Expected	Fold Enrichment	P-value	FDR	Term	Cluster
sno(s)RNA metabolic process (GO:0016074)	22	1	0.04	23.87	4.28E-02	1.00E+00	BP	5
sno(s)RNA processing (GO:0043144)	22	1	0.04	23.87	4.28E-02	1.00E+00	BP	5
activation of GTPase activity (GO:0090630)	23	1	0.04	22.83	4.46E-02	1.00E+00	BP	5
positive regulation of GTPase activity (GO:0043547)	27	1	0.05	19.45	5.19E-02	1.00E+00	BP	5
regulation of cellular ketone metabolic process (GO:0010565)	27	1	0.05	19.45	5.19E-02	1.00E+00	BP	5
mRNA modification (GO:0016556)	28	1	0.05	18.76	5.37E-02	1.00E+00	BP	5
regulation of GTPase activity (GO:0043087)	31	1	0.06	16.94	5.91E-02	1.00E+00	BP	5
positive regulation of hydrolase activity (GO:0051345)	34	1	0.06	15.45	6.44E-02	1.00E+00	BP	5
pseudouridine synthesis (GO:0001522)	38	1	0.07	13.82	7.15E-02	1.00E+00	BP	5
snRNA metabolic process (GO:0016073)	38	1	0.07	13.82	7.15E-02	1.00E+00	BP	5
regulation of small molecule metabolic process (GO:0062012)	40	1	0.08	13.13	7.51E-02	1.00E+00	BP	5
DNA-templated transcription termination (GO:0006353)	41	1	0.08	12.81	7.68E-02	1.00E+00	BP	5
regulatory ncRNA 3'-end processing (GO:0043628)	44	1	0.08	11.94	8.21E-02	1.00E+00	BP	5
cytokinin metabolic process (GO:0009690)	45	1	0.09	11.67	8.38E-02	1.00E+00	BP	5
diterpenoid biosynthetic process (GO:0016102)	49	1	0.09	10.72	9.08E-02	1.00E+00	BP	5
RNA 3'-end processing (GO:0031123)	111	2	0.21	9.46	1.97E-02	1.00E+00	BP	5
rRNA modification (GO:0000154)	57	1	0.11	9.21	1.05E-01	1.00E+00	BP	5
xylan biosynthetic process (GO:0045492)	59	1	0.11	8.9	1.08E-01	1.00E+00	BP	5
diterpenoid metabolic process (GO:0016101)	64	1	0.12	8.21	1.16E-01	1.00E+00	BP	5
amine metabolic process (GO:0009308)	130	2	0.25	8.08	2.63E-02	1.00E+00	BP	5
tRNA processing (GO:0008033)	148	2	0.28	7.1	3.32E-02	1.00E+00	BP	5
embryo development ending in seed dormancy (GO:0009793)	78	1	0.15	6.73	1.40E-01	1.00E+00	BP	5
embryo development (GO:0009790)	79	1	0.15	6.65	1.41E-01	1.00E+00	BP	5
RNA destabilization (GO:0050779)	80	1	0.15	6.56	1.43E-01	1.00E+00	BP	5
mRNA destabilization (GO:0061157)	80	1	0.15	6.56	1.43E-01	1.00E+00	BP	5
positive regulation of mRNA catabolic process (GO:0061014)	81	1	0.15	6.48	1.45E-01	1.00E+00	BP	5
positive regulation of cellular catabolic process (GO:0031331)	81	1	0.15	6.48	1.45E-01	1.00E+00	BP	5
cell surface receptor signaling pathway (GO:0007166)	85	1	0.16	6.18	1.51E-01	1.00E+00	BP	5
regulation of mRNA stability (GO:0043488)	89	1	0.17	5.9	1.58E-01	1.00E+00	BP	5
regulation of mRNA catabolic process (GO:0061013)	90	1	0.17	5.84	1.59E-01	1.00E+00	BP	5
positive regulation of mRNA metabolic process (GO:1903313)	91	1	0.17	5.77	1.61E-01	1.00E+00	BP	5
RNA phosphodiester bond hydrolysis, endonucleolytic (GO:0090502)	91	1	0.17	5.77	1.61E-01	1.00E+00	BP	5
regulation of RNA stability (GO:0043487)	91	1	0.17	5.77	1.61E-01	1.00E+00	BP	5
positive regulation of catalytic activity (GO:0043085)	93	1	0.18	5.65	1.64E-01	1.00E+00	BP	5
hormone metabolic process (GO:0042445)	98	1	0.19	5.36	1.72E-01	1.00E+00	BP	5
secondary metabolite biosynthetic process (GO:0044550)	98	1	0.19	5.36	1.72E-01	1.00E+00	BP	5
positive regulation of molecular function (GO:0044093)	102	1	0.19	5.15	1.78E-01	1.00E+00	BP	5
regulation of cellular catabolic process (GO:0031329)	105	1	0.2	5	1.83E-01	1.00E+00	BP	5
tRNA modification (GO:0006400)	106	1	0.2	4.95	1.84E-01	1.00E+00	BP	5
RNA-mediated gene silencing (GO:0031047)	117	1	0.22	4.49	2.01E-01	1.00E+00	BP	5
terpenoid biosynthetic process (GO:0016114)	120	1	0.23	4.38	2.06E-01	1.00E+00	BP	5
seed development (GO:0048316)	123	1	0.23	4.27	2.10E-01	1.00E+00	BP	5
regulation of mRNA metabolic process (GO:1903311)	124	1	0.24	4.24	2.12E-01	1.00E+00	BP	5
mRNA catabolic process (GO:0006402)	125	1	0.24	4.2	2.13E-01	1.00E+00	BP	5
negative regulation of amide metabolic process (GO:0034249)	129	1	0.25	4.07	2.19E-01	1.00E+00	BP	5
negative regulation of translation (GO:0017148)	129	1	0.25	4.07	2.19E-01	1.00E+00	BP	5
fruit development (GO:0010154)	131	1	0.25	4.01	2.22E-01	1.00E+00	BP	5
xylan metabolic process (GO:0045491)	131	1	0.25	4.01	2.22E-01	1.00E+00	BP	5
regulation of hormone levels (GO:0010817)	133	1	0.25	3.95	2.25E-01	1.00E+00	BP	5
positive regulation of catabolic process (GO:0009896)	133	1	0.25	3.95	2.25E-01	1.00E+00	BP	5
tRNA metabolic process (GO:0006399)	273	2	0.52	3.85	9.65E-02	1.00E+00	BP	5

GO Term	Genome	Count	Expected	Fold Enrichment	P-value	FDR	Term	Cluster
regulation of hydrolase activity (GO:0051336)	143	1	0.27	3.67	2.40E-01	1.00E+00	BP	5
RNA catabolic process (GO:0006401)	146	1	0.28	3.6	2.44E-01	1.00E+00	BP	5
terpenoid metabolic process (GO:0006721)	146	1	0.28	3.6	2.44E-01	1.00E+00	BP	5
cell wall polysaccharide biosynthetic process (GO:0070592)	146	1	0.28	3.6	2.44E-01	1.00E+00	BP	5
lipid transport (GO:0006869)	150	1	0.29	3.5	2.50E-01	1.00E+00	BP	5
RNA phosphodiester bond hydrolysis (GO:0090501)	151	1	0.29	3.48	2.52E-01	1.00E+00	BP	5
RNA modification (GO:0009451)	453	3	0.86	3.48	5.63E-02	1.00E+00	BP	5
positive regulation of RNA metabolic process (GO:0051254)	469	3	0.89	3.36	6.11E-02	1.00E+00	BP	5
auxin-activated signaling pathway (GO:0009734)	161	1	0.31	3.26	2.66E-01	1.00E+00	BP	5
cellular response to auxin stimulus (GO:0071365)	161	1	0.31	3.26	2.66E-01	1.00E+00	BP	5
positive regulation of nucleobase-containing compound metabolic process (GO:0045935)	488	3	0.93	3.23	6.71E-02	1.00E+00	BP	5
lipid localization (GO:0010876)	163	1	0.31	3.22	2.68E-01	1.00E+00	BP	5
defense response to other organism (GO:0098542)	332	2	0.63	3.16	1.33E-01	1.00E+00	BP	5
regulation of catabolic process (GO:0009894)	169	1	0.32	3.11	2.77E-01	1.00E+00	BP	5
negative regulation of gene expression (GO:0010629)	342	2	0.65	3.07	1.39E-01	1.00E+00	BP	5
defense response (GO:0006952)	515	3	0.98	3.06	7.61E-02	1.00E+00	BP	5
ncRNA processing (GO:0034470)	521	3	0.99	3.02	7.81E-02	1.00E+00	BP	5
lipid catabolic process (GO:0016042)	174	1	0.33	3.02	2.84E-01	1.00E+00	BP	5
nucleic acid phosphodiester bond hydrolysis (GO:0090305)	176	1	0.34	2.98	2.86E-01	1.00E+00	BP	5
secondary metabolic process (GO:0019748)	177	1	0.34	2.97	2.88E-01	1.00E+00	BP	5
positive regulation of cellular metabolic process (GO:0031325)	535	3	1.02	2.94	8.30E-02	1.00E+00	BP	5
isoprenoid biosynthetic process (GO:0008299)	182	1	0.35	2.89	2.95E-01	1.00E+00	BP	5
positive regulation of DNA-templated transcription (GO:0045893)	376	2	0.72	2.79	1.62E-01	1.00E+00	BP	5
positive regulation of RNA biosynthetic process (GO:1902680)	376	2	0.72	2.79	1.62E-01	1.00E+00	BP	5
positive regulation of macromolecule biosynthetic process (GO:0010557)	394	2	0.75	2.67	1.74E-01	1.00E+00	BP	5
positive regulation of nitrogen compound metabolic process (GO:0051173)	593	3	1.13	2.66	1.05E-01	1.00E+00	BP	5
nucleobase-containing compound catabolic process (GO:0034655)	198	1	0.38	2.65	3.16E-01	1.00E+00	BP	5
positive regulation of cellular process (GO:0048522)	597	3	1.14	2.64	1.06E-01	1.00E+00	BP	5
positive regulation of cellular biosynthetic process (GO:0031328)	399	2	0.76	2.63	1.77E-01	1.00E+00	BP	5
positive regulation of macromolecule metabolic process (GO:0010604)	603	3	1.15	2.61	1.09E-01	1.00E+00	BP	5
positive regulation of biosynthetic process (GO:0009891)	403	2	0.77	2.61	1.80E-01	1.00E+00	BP	5
positive regulation of metabolic process (GO:0009893)	620	3	1.18	2.54	1.15E-01	1.00E+00	BP	5
ncRNA metabolic process (GO:0034660)	637	3	1.21	2.47	1.22E-01	1.00E+00	BP	5
regulation of biological quality (GO:0065008)	433	2	0.82	2.43	2.00E-01	1.00E+00	BP	5
negative regulation of protein metabolic process (GO:0051248)	218	1	0.42	2.41	3.41E-01	1.00E+00	BP	5
response to other organism (GO:0051707)	437	2	0.83	2.4	2.03E-01	1.00E+00	BP	5
response to external biotic stimulus (GO:0043207)	437	2	0.83	2.4	2.03E-01	1.00E+00	BP	5
cell differentiation (GO:0030154)	223	1	0.42	2.36	3.48E-01	1.00E+00	BP	5
biological process involved in interspecies interaction between organisms (GO:0044419)	457	2	0.87	2.3	2.17E-01	1.00E+00	BP	5
regulation of amide metabolic process (GO:0034248)	230	1	0.44	2.28	3.56E-01	1.00E+00	BP	5
regulation of translation (GO:0006417)	230	1	0.44	2.28	3.56E-01	1.00E+00	BP	5
isoprenoid metabolic process (GO:0006720)	231	1	0.44	2.27	3.58E-01	1.00E+00	BP	5
heterocycle catabolic process (GO:0046700)	233	1	0.44	2.25	3.60E-01	1.00E+00	BP	5
response to biotic stimulus (GO:0009607)	470	2	0.89	2.23	2.26E-01	1.00E+00	BP	5
cellular nitrogen compound catabolic process (GO:0044270)	235	1	0.45	2.23	3.63E-01	1.00E+00	BP	5
cellular developmental process (GO:0048869)	240	1	0.46	2.19	3.69E-01	1.00E+00	BP	5
hemicellulose metabolic process (GO:0010410)	240	1	0.46	2.19	3.69E-01	1.00E+00	BP	5
positive regulation of biological process (GO:0048518)	749	3	1.43	2.1	1.72E-01	1.00E+00	BP	5
cell wall polysaccharide metabolic process (GO:0010383)	250	1	0.48	2.1	3.81E-01	1.00E+00	BP	5
RNA processing (GO:0006396)	1041	4	1.98	2.02	1.38E-01	1.00E+00	BP	5

GO Term	Genome	Count	Expected	Fold Enrichment	P-value	FDR	Term	Cluster
cellular oxidant detoxification (GO:0098869)	266	1	0.51	1.97	3.99E-01	1.00E+00	BP	5
reproductive structure development (GO:0048608)	270	1	0.51	1.95	4.04E-01	1.00E+00	BP	5
negative regulation of macromolecule biosynthetic process (GO:0010558)	271	1	0.52	1.94	4.05E-01	1.00E+00	BP	5
reproductive system development (GO:0061458)	273	1	0.52	1.92	4.07E-01	1.00E+00	BP	5
cell wall macromolecule metabolic process (GO:0044036)	274	1	0.52	1.92	4.08E-01	1.00E+00	BP	5
DNA-templated transcription (GO:0006351)	824	3	1.57	1.91	2.08E-01	1.00E+00	BP	5
regulation of gene expression (GO:0010468)	3303	12	6.29	1.91	3.29E-02	1.00E+00	BP	5
negative regulation of cellular biosynthetic process (GO:0031327)	279	1	0.53	1.88	4.14E-01	1.00E+00	BP	5
RNA biosynthetic process (GO:0032774)	840	3	1.6	1.88	2.16E-01	1.00E+00	BP	5
polysaccharide biosynthetic process (GO:0000271)	281	1	0.54	1.87	4.16E-01	1.00E+00	BP	5
negative regulation of biosynthetic process (GO:0009890)	281	1	0.54	1.87	4.16E-01	1.00E+00	BP	5
response to auxin (GO:0009733)	283	1	0.54	1.86	4.19E-01	1.00E+00	BP	5
cellular response to toxic substance (GO:0097237)	283	1	0.54	1.86	4.19E-01	1.00E+00	BP	5
cellular detoxification (GO:1990748)	283	1	0.54	1.86	4.19E-01	1.00E+00	BP	5
regulation of RNA metabolic process (GO:0051252)	2843	10	5.41	1.85	6.77E-02	1.00E+00	BP	5
aromatic compound catabolic process (GO:0019439)	285	1	0.54	1.84	4.21E-01	1.00E+00	BP	5
post-transcriptional regulation of gene expression (GO:0010608)	289	1	0.55	1.82	4.25E-01	1.00E+00	BP	5
organic cyclic compound catabolic process (GO:1901361)	290	1	0.55	1.81	4.26E-01	1.00E+00	BP	5
regulation of nucleobase-containing compound metabolic process (GO:0019219)	2913	10	5.55	1.8	7.12E-02	1.00E+00	BP	5
response to external stimulus (GO:0009605)	583	2	1.11	1.8	3.06E-01	1.00E+00	BP	5
regulation of macromolecule biosynthetic process (GO:0010556)	2942	10	5.6	1.79	7.29E-02	1.00E+00	BP	5
regulation of cellular biosynthetic process (GO:0031326)	2959	10	5.63	1.77	7.40E-02	1.00E+00	BP	5
regulation of biosynthetic process (GO:0009889)	2969	10	5.65	1.77	7.47E-02	1.00E+00	BP	5
RNA metabolic process (GO:0016070)	2384	8	4.54	1.76	1.37E-01	1.00E+00	BP	5
regulation of RNA biosynthetic process (GO:2001141)	2705	9	5.15	1.75	1.03E-01	1.00E+00	BP	5
regulation of DNA-templated transcription (GO:0006355)	2705	9	5.15	1.75	1.03E-01	1.00E+00	BP	5
negative regulation of macromolecule metabolic process (GO:0010605)	602	2	1.15	1.74	3.19E-01	1.00E+00	BP	5
developmental process involved in reproduction (GO:0003006)	307	1	0.58	1.71	4.45E-01	1.00E+00	BP	5
regulation of macromolecule metabolic process (GO:0060255)	3684	12	7.01	1.71	6.95E-02	1.00E+00	BP	5
mRNA metabolic process (GO:0016071)	615	2	1.17	1.71	3.28E-01	1.00E+00	BP	5
negative regulation of metabolic process (GO:0009892)	615	2	1.17	1.71	3.28E-01	1.00E+00	BP	5
regulation of metabolic process (GO:0019222)	3766	12	7.17	1.67	7.33E-02	1.00E+00	BP	5
response to oxidative stress (GO:0006979)	319	1	0.61	1.65	4.57E-01	1.00E+00	BP	5
regulation of cellular metabolic process (GO:0031323)	3270	10	6.23	1.61	1.37E-01	1.00E+00	BP	5
regulation of catalytic activity (GO:0050790)	330	1	0.63	1.59	4.69E-01	1.00E+00	BP	5
negative regulation of cellular metabolic process (GO:0031324)	332	1	0.63	1.58	4.71E-01	1.00E+00	BP	5
detoxification (GO:0098754)	334	1	0.64	1.57	4.73E-01	1.00E+00	BP	5
regulation of nitrogen compound metabolic process (GO:0051171)	3361	10	6.4	1.56	1.45E-01	1.00E+00	BP	5
rRNA processing (GO:0006364)	340	1	0.65	1.54	4.79E-01	1.00E+00	BP	5
regulation of primary metabolic process (GO:0080090)	3400	10	6.47	1.54	1.48E-01	1.00E+00	BP	5
regulation of molecular function (GO:0065009)	344	1	0.66	1.53	4.83E-01	1.00E+00	BP	5
rRNA metabolic process (GO:0016072)	347	1	0.66	1.51	4.86E-01	1.00E+00	BP	5
post-embryonic development (GO:0009791)	348	1	0.66	1.51	4.87E-01	1.00E+00	BP	5
response to toxic substance (GO:0009636)	358	1	0.68	1.47	4.96E-01	1.00E+00	BP	5
biological regulation (GO:0065007)	5626	15	10.71	1.4	1.84E-01	1.00E+00	BP	5
negative regulation of nitrogen compound metabolic process (GO:0051172)	393	1	0.75	1.34	5.29E-01	1.00E+00	BP	5
carbohydrate biosynthetic process (GO:0016051)	396	1	0.75	1.33	5.32E-01	1.00E+00	BP	5
negative regulation of biological process (GO:0048519)	801	2	1.53	1.31	6.66E-01	1.00E+00	BP	5
regulation of biological process (GO:0050789)	5297	13	10.09	1.29	3.10E-01	1.00E+00	BP	5
Unclassified (UNCLASSIFIED)	17419	42	33.17	1.27	4.74E-02	1.00E+00	BP	5
regulation of cellular process (GO:0050794)	4625	11	8.81	1.25	4.70E-01	1.00E+00	BP	5

GO Term	Genome	Count	Expected	Fold Enrichment	P-value	FDR	Term	Cluster
nucleobase-containing compound biosynthetic process (GO:0034654)	1266	3	2.41	1.24	5.21E-01	1.00E+00	BP	5
nucleic acid metabolic process (GO:0090304)	3395	8	6.46	1.24	5.33E-01	1.00E+00	BP	5
heterocycle metabolic process (GO:0046483)	4164	9	7.93	1.14	7.05E-01	1.00E+00	BP	5
cellular response to chemical stimulus (GO:0070887)	934	2	1.78	1.12	6.99E-01	1.00E+00	BP	5
cellular aromatic compound metabolic process (GO:0006725)	4327	9	8.24	1.09	7.13E-01	1.00E+00	BP	5
system development (GO:0048731)	482	1	0.92	1.09	6.03E-01	1.00E+00	BP	5
developmental process (GO:0032502)	965	2	1.84	1.09	7.07E-01	1.00E+00	BP	5
nucleobase-containing compound metabolic process (GO:0006139)	3868	8	7.37	1.09	7.01E-01	1.00E+00	BP	5
negative regulation of cellular process (GO:0048523)	484	1	0.92	1.09	6.05E-01	1.00E+00	BP	5
heterocycle biosynthetic process (GO:0018130)	1487	3	2.83	1.06	7.62E-01	1.00E+00	BP	5
hormone-mediated signaling pathway (GO:0009755)	497	1	0.95	1.06	6.15E-01	1.00E+00	BP	5
ribosome biogenesis (GO:0042254)	499	1	0.95	1.05	6.16E-01	1.00E+00	BP	5
reproductive process (GO:0022414)	503	1	0.96	1.04	6.19E-01	1.00E+00	BP	5
cellular response to hormone stimulus (GO:0032870)	504	1	0.96	1.04	6.20E-01	1.00E+00	BP	5
cellular response to endogenous stimulus (GO:0071495)	510	1	0.97	1.03	6.24E-01	1.00E+00	BP	5
protein phosphorylation (GO:0006468)	1543	3	2.94	1.02	7.70E-01	1.00E+00	BP	5
aromatic compound biosynthetic process (GO:0019438)	1547	3	2.95	1.02	7.70E-01	1.00E+00	BP	5
gene expression (GO:0010467)	2102	4	4	1	1.00E+00	1.00E+00	BP	5
regulation of protein metabolic process (GO:0051246)	533	1	1.01	0.99	1.00E+00	1.00E+00	BP	5
organic cyclic compound metabolic process (GO:1901360)	4430	8	8.44	0.95	1.00E+00	1.00E+00	BP	5
organic cyclic compound biosynthetic process (GO:1901362)	1677	3	3.19	0.94	1.00E+00	1.00E+00	BP	5
polysaccharide metabolic process (GO:0005976)	562	1	1.07	0.93	1.00E+00	1.00E+00	BP	5
reproduction (GO:0000003)	562	1	1.07	0.93	1.00E+00	1.00E+00	BP	5
response to stress (GO:0006950)	2296	4	4.37	0.91	1.00E+00	1.00E+00	BP	5
cellular response to organic substance (GO:0071310)	578	1	1.1	0.91	1.00E+00	1.00E+00	BP	5
cell wall organization or biogenesis (GO:0071554)	586	1	1.12	0.9	1.00E+00	1.00E+00	BP	5
lipid metabolic process (GO:0006629)	1209	2	2.3	0.87	1.00E+00	1.00E+00	BP	5
macromolecule biosynthetic process (GO:0009059)	2436	4	4.64	0.86	1.00E+00	1.00E+00	BP	5
ribonucleoprotein complex biogenesis (GO:0022613)	613	1	1.17	0.86	1.00E+00	1.00E+00	BP	5
cellular nitrogen compound metabolic process (GO:0034641)	4908	8	9.35	0.86	8.60E-01	1.00E+00	BP	5
macromolecule modification (GO:0043412)	3738	6	7.12	0.84	8.44E-01	1.00E+00	BP	5
multicellular organism development (GO:0007275)	627	1	1.19	0.84	1.00E+00	1.00E+00	BP	5
signal transduction (GO:0007165)	1258	2	2.4	0.83	1.00E+00	1.00E+00	BP	5
signaling (GO:0023052)	1277	2	2.43	0.82	1.00E+00	1.00E+00	BP	5
organic substance metabolic process (GO:0071704)	12450	19	23.71	0.8	2.65E-01	1.00E+00	BP	5
cell communication (GO:0007154)	1326	2	2.52	0.79	1.00E+00	1.00E+00	BP	5
biological_process (GO:0008150)	21970	33	41.83	0.79	4.74E-02	1.00E+00	BP	5
carbohydrate metabolic process (GO:0005975)	1347	2	2.56	0.78	1.00E+00	1.00E+00	BP	5
multicellular organismal process (GO:0032501)	686	1	1.31	0.77	1.00E+00	1.00E+00	BP	5
lipid biosynthetic process (GO:0008610)	706	1	1.34	0.74	1.00E+00	1.00E+00	BP	5
metabolic process (GO:0008152)	13613	19	25.92	0.73	1.13E-01	1.00E+00	BP	5
phosphorylation (GO:0016310)	2164	3	4.12	0.73	7.99E-01	1.00E+00	BP	5
cellular metabolic process (GO:0044237)	9471	13	18.03	0.72	2.23E-01	1.00E+00	BP	5
response to stimulus (GO:0050896)	4415	6	8.41	0.71	4.66E-01	1.00E+00	BP	5
nitrogen compound metabolic process (GO:0006807)	9609	13	18.3	0.71	1.79E-01	1.00E+00	BP	5
macromolecule metabolic process (GO:0043170)	8871	12	16.89	0.71	2.13E-01	1.00E+00	BP	5
response to hormone (GO:0009725)	756	1	1.44	0.69	1.00E+00	1.00E+00	BP	5
response to chemical (GO:0042221)	1513	2	2.88	0.69	1.00E+00	1.00E+00	BP	5
response to endogenous stimulus (GO:0009719)	762	1	1.45	0.69	1.00E+00	1.00E+00	BP	5
primary metabolic process (GO:0044238)	11484	15	21.87	0.69	9.75E-02	1.00E+00	BP	5
cellular catabolic process (GO:0044248)	783	1	1.49	0.67	1.00E+00	1.00E+00	BP	5

GO Term	Genome	Count	Expected	Fold Enrichment	P-value	FDR	Term	Cluster
cellular nitrogen compound biosynthetic process (GO:0044271)	2423	3	4.61	0.65	6.29E-01	1.00E+00	BP	5
organic substance biosynthetic process (GO:1901576)	4099	5	7.8	0.64	3.48E-01	1.00E+00	BP	5
anatomical structure development (GO:0048856)	825	1	1.57	0.64	1.00E+00	1.00E+00	BP	5
biosynthetic process (GO:0009058)	4259	5	8.11	0.62	3.49E-01	1.00E+00	BP	5
cellular biosynthetic process (GO:0044249)	3461	4	6.59	0.61	4.12E-01	1.00E+00	BP	5
cellular response to stimulus (GO:0051716)	2710	3	5.16	0.58	4.90E-01	1.00E+00	BP	5
cellular process (GO:0009987)	15372	17	29.27	0.58	4.13E-03	1.00E+00	BP	5
cellular lipid metabolic process (GO:0044255)	907	1	1.73	0.58	1.00E+00	1.00E+00	BP	5
organic substance catabolic process (GO:1901575)	1863	2	3.55	0.56	5.86E-01	1.00E+00	BP	5
catabolic process (GO:0009056)	1947	2	3.71	0.54	5.89E-01	1.00E+00	BP	5
phosphate-containing compound metabolic process (GO:0006796)	2984	3	5.68	0.53	3.77E-01	1.00E+00	BP	5
response to organic substance (GO:0010033)	1000	1	1.9	0.53	1.00E+00	1.00E+00	BP	5
phosphorus metabolic process (GO:0006793)	3030	3	5.77	0.52	2.83E-01	1.00E+00	BP	5
protein modification process (GO:0036211)	3194	3	6.08	0.49	2.85E-01	1.00E+00	BP	5
macromolecule catabolic process (GO:0009057)	1069	1	2.04	0.49	7.25E-01	1.00E+00	BP	5
macromolecule localization (GO:0033036)	1264	1	2.41	0.42	7.35E-01	1.00E+00	BP	5
organonitrogen compound metabolic process (GO:1901564)	6443	5	12.27	0.41	1.88E-02	1.00E+00	BP	5
cellular component biogenesis (GO:0044085)	1401	1	2.67	0.37	5.26E-01	1.00E+00	BP	5
protein metabolic process (GO:0019538)	5254	3	10	0.3	1.56E-02	1.00E+00	BP	5
organic substance transport (GO:0071702)	1759	1	3.35	0.3	2.64E-01	1.00E+00	BP	5
transport (GO:0006810)	3186	1	6.07	0.16	3.03E-02	1.00E+00	BP	5
establishment of localization (GO:0051234)	3225	1	6.14	0.16	3.09E-02	1.00E+00	BP	5
localization (GO:0051179)	3340	1	6.36	0.16	2.02E-02	1.00E+00	BP	5
cellular component organization or biogenesis (GO:0071840)	3437	1	6.54	0.15	2.10E-02	1.00E+00	BP	5
queuine tRNA-ribosyltransferase activity (GO:0008479)	2	1	0	> 100	5.69E-03	1.00E+00	MF	5
phenethylamine:oxygen oxidoreductase (deaminating) activity (GO:0052596)	3	1	0.01	> 100	7.58E-03	1.00E+00	MF	5
aliphatic amine oxidase activity (GO:0052595)	3	1	0.01	> 100	7.58E-03	1.00E+00	MF	5
aminoacetone:oxygen oxidoreductase(deaminating) activity (GO:0052594)	3	1	0.01	> 100	7.58E-03	1.00E+00	MF	5
tryptamine:oxygen oxidoreductase (deaminating) activity (GO:0052593)	3	1	0.01	> 100	7.58E-03	1.00E+00	MF	5
RNA uridylyltransferase activity (GO:0050265)	3	1	0.01	> 100	7.58E-03	1.00E+00	MF	5
primary amine oxidase activity (GO:0008131)	6	1	0.01	87.53	1.32E-02	1.00E+00	MF	5
oxidoreductase activity, acting on the CH-NH2 group of donors, oxygen as acceptor (GO:0016641)	8	1	0.02	65.65	1.70E-02	1.00E+00	MF	5
myosin XI tail binding (GO:0080115)	20	2	0.04	52.52	8.03E-04	1.00E+00	MF	5
myosin heavy chain binding (GO:0032036)	20	2	0.04	52.52	8.03E-04	1.00E+00	MF	5
myosin tail binding (GO:0032029)	20	2	0.04	52.52	8.03E-04	6.74E-01	MF	5
myosin binding (GO:0017022)	24	2	0.05	43.77	1.12E-03	7.08E-01	MF	5
mannan endo-1,4-beta-mannosidase activity (GO:0016985)	13	1	0.02	40.4	2.63E-02	1.00E+00	MF	5
beta-mannosidase activity (GO:0004567)	14	1	0.03	37.51	2.81E-02	1.00E+00	MF	5
cytokinin dehydrogenase activity (GO:0019139)	17	1	0.03	30.89	3.37E-02	1.00E+00	MF	5
oxidoreductase activity, acting on the CH-NH2 group of donors (GO:0016638)	19	1	0.04	27.64	3.73E-02	1.00E+00	MF	5
phospholipase A1 activity (GO:0008970)	19	1	0.04	27.64	3.73E-02	1.00E+00	MF	5
uridylyltransferase activity (GO:0070569)	22	1	0.04	23.87	4.28E-02	1.00E+00	MF	5
pseudouridine synthase activity (GO:0009982)	29	1	0.06	18.11	5.55E-02	1.00E+00	MF	5
glucan endo-1,3-beta-D-glucosidase activity (GO:0042973)	30	1	0.06	17.51	5.73E-02	1.00E+00	MF	5
mannosidase activity (GO:0015923)	33	1	0.06	15.91	6.27E-02	1.00E+00	MF	5
quinone binding (GO:0048038)	34	1	0.06	15.45	6.44E-02	1.00E+00	MF	5
FAD binding (GO:0071949)	89	2	0.17	11.8	1.31E-02	1.00E+00	MF	5
transcription coactivator activity (GO:0003713)	48	1	0.09	10.94	8.91E-02	1.00E+00	MF	5
terpene synthase activity (GO:0010333)	53	1	0.1	9.91	9.77E-02	1.00E+00	MF	5
oxidoreductase activity, acting on the CH-NH group of donors (GO:0016645)	55	1	0.1	9.55	1.01E-01	1.00E+00	MF	5
carbon-oxygen lyase activity, acting on phosphates (GO:0016838)	61	1	0.12	8.61	1.11E-01	1.00E+00	MF	5

GO Term	Genome	Count	Expected	Fold Enrichment	P-value	FDR	Term	Cluster
copper ion binding (GO:0005507)	69	1	0.13	7.61	1.25E-01	1.00E+00	MF	5
beta-glucosidase activity (GO:0008422)	80	1	0.15	6.56	1.43E-01	1.00E+00	MF	5
phospholipase activity (GO:0004620)	89	1	0.17	5.9	1.58E-01	1.00E+00	MF	5
glucosidase activity (GO:0015926)	94	1	0.18	5.59	1.66E-01	1.00E+00	MF	5
intramolecular transferase activity (GO:0016866)	95	1	0.18	5.53	1.67E-01	1.00E+00	MF	5
GTPase activator activity (GO:0005096)	110	1	0.21	4.77	1.91E-01	1.00E+00	MF	5
pentosyltransferase activity (GO:0016763)	112	1	0.21	4.69	1.94E-01	1.00E+00	MF	5
oxidoreductase activity, acting on paired donors, with incorporation or reduction of molecular oxygen, NAD(P)H as one donor, and incorporation of one atom of oxygen (GO:0016709)	113	1	0.22	4.65	1.95E-01	1.00E+00	MF	5
flavin adenine dinucleotide binding (GO:0050660)	230	2	0.44	4.57	7.22E-02	1.00E+00	MF	5
ADP binding (GO:0043531)	134	1	0.26	3.92	2.27E-01	1.00E+00	MF	5
lipase activity (GO:0016298)	141	1	0.27	3.72	2.37E-01	1.00E+00	MF	5
transcription coregulator activity (GO:0003712)	165	1	0.31	3.18	2.71E-01	1.00E+00	MF	5
carboxylic ester hydrolase activity (GO:0052689)	165	1	0.31	3.18	2.71E-01	1.00E+00	MF	5
calmodulin binding (GO:0005516)	177	1	0.34	2.97	2.88E-01	1.00E+00	MF	5
magnesium ion binding (GO:0000287)	178	1	0.34	2.95	2.89E-01	1.00E+00	MF	5
enzyme activator activity (GO:0008047)	184	1	0.35	2.85	2.97E-01	1.00E+00	MF	5
nucleoside-triphosphatase regulator activity (GO:0060589)	191	1	0.36	2.75	3.07E-01	1.00E+00	MF	5
carbon-oxygen lyase activity (GO:0016835)	191	1	0.36	2.75	3.07E-01	1.00E+00	MF	5
GTPase regulator activity (GO:0030695)	191	1	0.36	2.75	3.07E-01	1.00E+00	MF	5
catalytic activity, acting on a tRNA (GO:0140101)	191	1	0.36	2.75	3.07E-01	1.00E+00	MF	5
molecular function activator activity (GO:0140677)	203	1	0.39	2.59	3.22E-01	1.00E+00	MF	5
cytoskeletal protein binding (GO:0008092)	440	2	0.84	2.39	2.05E-01	1.00E+00	MF	5
monooxygenase activity (GO:0004497)	441	2	0.84	2.38	2.06E-01	1.00E+00	MF	5
oxidoreductase activity, acting on peroxide as acceptor (GO:0016684)	224	1	0.43	2.34	3.49E-01	1.00E+00	MF	5
peroxidase activity (GO:0004601)	224	1	0.43	2.34	3.49E-01	1.00E+00	MF	5
hydrolase activity, hydrolyzing O-glycosyl compounds (GO:0004553)	462	2	0.88	2.27	2.21E-01	1.00E+00	MF	5
antioxidant activity (GO:0016209)	261	1	0.5	2.01	3.94E-01	1.00E+00	MF	5
hydrolase activity, acting on glycosyl bonds (GO:0016798)	525	2	1	2	2.65E-01	1.00E+00	MF	5
nucleotidyltransferase activity (GO:0016779)	279	1	0.53	1.88	4.14E-01	1.00E+00	MF	5
heme binding (GO:0020037)	565	2	1.08	1.86	2.93E-01	1.00E+00	MF	5
catalytic activity, acting on RNA (GO:0140098)	595	2	1.13	1.77	3.14E-01	1.00E+00	MF	5
tetrapyrrole binding (GO:0046906)	609	2	1.16	1.72	3.24E-01	1.00E+00	MF	5
protein serine/threonine kinase activity (GO:0004674)	923	3	1.76	1.71	2.58E-01	1.00E+00	MF	5
lipid binding (GO:0008289)	348	1	0.66	1.51	4.87E-01	1.00E+00	MF	5
transcription regulator activity (GO:0140110)	1753	5	3.34	1.5	3.89E-01	1.00E+00	MF	5
DNA-binding transcription factor activity (GO:0003700)	1586	4	3.02	1.32	5.47E-01	1.00E+00	MF	5
DNA binding (GO:0003677)	3182	8	6.06	1.32	3.93E-01	1.00E+00	MF	5
oxidoreductase activity (GO:0016491)	2048	5	3.9	1.28	5.96E-01	1.00E+00	MF	5
hydrolase activity, acting on ester bonds (GO:0016788)	1254	3	2.39	1.26	5.17E-01	1.00E+00	MF	5
isomerase activity (GO:0016853)	429	1	0.82	1.22	5.61E-01	1.00E+00	MF	5
transition metal ion binding (GO:0046914)	1290	3	2.46	1.22	7.38E-01	1.00E+00	MF	5
Unclassified (UNCLASSIFIED)	16329	36	31.09	1.16	2.91E-01	1.00E+00	MF	5
iron ion binding (GO:0005506)	465	1	0.89	1.13	5.90E-01	1.00E+00	MF	5
metal ion binding (GO:0046872)	3774	8	7.19	1.11	6.94E-01	1.00E+00	MF	5
nucleic acid binding (GO:0003676)	5685	12	10.82	1.11	6.25E-01	1.00E+00	MF	5
cation binding (GO:0043169)	3802	8	7.24	1.11	6.96E-01	1.00E+00	MF	5
glycosyltransferase activity (GO:0016757)	972	2	1.85	1.08	7.09E-01	1.00E+00	MF	5
binding (GO:0005488)	14290	29	27.21	1.07	7.19E-01	1.00E+00	MF	5
phosphatase activity (GO:0016791)	505	1	0.96	1.04	6.20E-01	1.00E+00	MF	5
oxidoreductase activity, acting on paired donors, with incorporation or reduction of molecular oxygen (GO:0016705)	513	1	0.98	1.02	6.26E-01	1.00E+00	MF	5

GO Term	Genome	Count	Expected	Fold Enrichment	P-value	FDR	Term	Cluster
heterocyclic compound binding (GO:1901363)	10349	20	19.71	1.01	8.96E-01	1.00E+00	MF	5
organic cyclic compound binding (GO:0097159)	10373	20	19.75	1.01	1.00E+00	1.00E+00	MF	5
RNA binding (GO:0003723)	2087	4	3.97	1.01	1.00E+00	1.00E+00	MF	5
ligase activity (GO:0016874)	529	1	1.01	0.99	1.00E+00	1.00E+00	MF	5
acyltransferase activity, transferring groups other than amino-acyl groups (GO:0016747)	531	1	1.01	0.99	1.00E+00	1.00E+00	MF	5
protein kinase activity (GO:0004672)	1604	3	3.05	0.98	1.00E+00	1.00E+00	MF	5
ubiquitin-protein transferase activity (GO:0004842)	558	1	1.06	0.94	1.00E+00	1.00E+00	MF	5
ion binding (GO:0043167)	7841	14	14.93	0.94	8.85E-01	1.00E+00	MF	5
lyase activity (GO:0016829)	572	1	1.09	0.92	1.00E+00	1.00E+00	MF	5
phosphoric ester hydrolase activity (GO:0042578)	576	1	1.1	0.91	1.00E+00	1.00E+00	MF	5
acyltransferase activity (GO:0016746)	1155	2	2.2	0.91	1.00E+00	1.00E+00	MF	5
ubiquitin-like protein transferase activity (GO:0019787)	578	1	1.1	0.91	1.00E+00	1.00E+00	MF	5
aminoacyltransferase activity (GO:0016755)	583	1	1.11	0.9	1.00E+00	1.00E+00	MF	5
sequence-specific DNA binding (GO:0043565)	1176	2	2.24	0.89	1.00E+00	1.00E+00	MF	5
molecular_function (GO:0003674)	23060	39	43.91	0.89	2.91E-01	1.00E+00	MF	5
enzyme regulator activity (GO:0030234)	604	1	1.15	0.87	1.00E+00	1.00E+00	MF	5
phosphotransferase activity, alcohol group as acceptor (GO:0016773)	1823	3	3.47	0.86	1.00E+00	1.00E+00	MF	5
catalytic activity (GO:0003824)	13387	22	25.49	0.86	4.64E-01	1.00E+00	MF	5
transferase activity, transferring phosphorus-containing groups (GO:0016772)	2537	4	4.83	0.83	1.00E+00	1.00E+00	MF	5
transferase activity (GO:0016740)	5764	9	10.98	0.82	6.25E-01	1.00E+00	MF	5
catalytic activity, acting on a nucleic acid (GO:0140640)	1293	2	2.46	0.81	1.00E+00	1.00E+00	MF	5
zinc ion binding (GO:0008270)	651	1	1.24	0.81	1.00E+00	1.00E+00	MF	5
molecular function regulator activity (GO:0098772)	652	1	1.24	0.81	1.00E+00	1.00E+00	MF	5
double-stranded DNA binding (GO:0003690)	723	1	1.38	0.73	1.00E+00	1.00E+00	MF	5
nucleotide binding (GO:0000166)	4340	6	8.26	0.73	5.78E-01	1.00E+00	MF	5
nucleoside phosphate binding (GO:1901265)	4340	6	8.26	0.73	5.78E-01	1.00E+00	MF	5
kinase activity (GO:0016301)	2226	3	4.24	0.71	8.00E-01	1.00E+00	MF	5
anion binding (GO:0043168)	4484	6	8.54	0.7	4.66E-01	1.00E+00	MF	5
hydrolase activity (GO:0016787)	4566	6	8.69	0.69	4.68E-01	1.00E+00	MF	5
small molecule binding (GO:0036094)	4599	6	8.76	0.69	4.69E-01	1.00E+00	MF	5
protein binding (GO:0005515)	2323	3	4.42	0.68	6.29E-01	1.00E+00	MF	5
adenyl ribonucleotide binding (GO:0032559)	3497	4	6.66	0.6	4.13E-01	1.00E+00	MF	5
adenyl nucleotide binding (GO:0030554)	3702	4	7.05	0.57	3.19E-01	1.00E+00	MF	5
purine ribonucleotide binding (GO:0032555)	3854	4	7.34	0.55	2.44E-01	1.00E+00	MF	5
ribonucleotide binding (GO:0032553)	3896	4	7.42	0.54	2.44E-01	1.00E+00	MF	5
catalytic activity, acting on a protein (GO:0140096)	3926	4	7.48	0.54	2.44E-01	1.00E+00	MF	5
carbohydrate derivative binding (GO:0097367)	3928	4	7.48	0.53	2.44E-01	1.00E+00	MF	5
purine nucleotide binding (GO:0017076)	4059	4	7.73	0.52	1.85E-01	1.00E+00	MF	5
ATP binding (GO:0005524)	3362	3	6.4	0.47	2.12E-01	1.00E+00	MF	5
purine ribonucleoside triphosphate binding (GO:0035639)	3719	3	7.08	0.42	1.16E-01	1.00E+00	MF	5
nucleolar ribonuclease P complex (GO:0005655)	3	1	0.01	> 100	7.58E-03	6.60E-01	CC	5
multimeric ribonuclease P complex (GO:0030681)	3	1	0.01	> 100	7.58E-03	6.00E-01	CC	5
ribonuclease P complex (GO:0030677)	6	1	0.01	87.53	1.32E-02	9.60E-01	CC	5
box H/ACA snoRNP complex (GO:0031429)	9	1	0.02	58.35	1.88E-02	1.00E+00	CC	5
box H/ACA RNP complex (GO:0072588)	9	1	0.02	58.35	1.88E-02	1.00E+00	CC	5
endoribonuclease complex (GO:1902555)	11	1	0.02	47.74	2.26E-02	1.00E+00	CC	5
endonuclease complex (GO:1905348)	14	1	0.03	37.51	2.81E-02	1.00E+00	CC	5
sno(s)RNA-containing ribonucleoprotein complex (GO:0005732)	44	1	0.08	11.94	8.21E-02	1.00E+00	CC	5
CCR4-NOT complex (GO:0030014)	61	1	0.12	8.61	1.11E-01	1.00E+00	CC	5
extracellular space (GO:0005615)	132	1	0.25	3.98	2.24E-01	1.00E+00	CC	5
nucleolus (GO:0005730)	338	2	0.64	3.11	1.37E-01	1.00E+00	CC	5

GO Term	Genome	Count	Expected	Fold Enrichment	P-value	FDR	Term	Cluster
DNA-directed RNA polymerase complex (GO:0000428)	178	1	0.34	2.95	2.89E-01	1.00E+00	CC	5
RNA polymerase complex (GO:0030880)	184	1	0.35	2.85	2.97E-01	1.00E+00	CC	5
intracellular protein-containing complex (GO:0140535)	792	3	1.51	1.99	1.92E-01	1.00E+00	CC	5
transferase complex, transferring phosphorus-containing groups (GO:0061695)	372	1	0.71	1.41	5.10E-01	1.00E+00	CC	5
Unclassified (UNCLASSIFIED)	19561	52	37.25	1.4	7.13E-04	1.24E-01	CC	5
nuclear lumen (GO:0031981)	823	2	1.57	1.28	6.72E-01	1.00E+00	CC	5
nuclear protein-containing complex (GO:0140513)	991	2	1.89	1.06	7.14E-01	1.00E+00	CC	5
catalytic complex (GO:1902494)	1505	3	2.87	1.05	7.64E-01	1.00E+00	CC	5
intracellular organelle lumen (GO:0070013)	1025	2	1.95	1.02	7.23E-01	1.00E+00	CC	5
membrane-enclosed lumen (GO:0031974)	1025	2	1.95	1.02	7.23E-01	1.00E+00	CC	5
organelle lumen (GO:0043233)	1025	2	1.95	1.02	7.23E-01	1.00E+00	CC	5
ribonucleoprotein complex (GO:1990904)	1113	2	2.12	0.94	1.00E+00	1.00E+00	CC	5
nucleus (GO:0005634)	6118	9	11.65	0.77	5.22E-01	1.00E+00	CC	5
extracellular region (GO:0005576)	698	1	1.33	0.75	1.00E+00	1.00E+00	CC	5
plasma membrane (GO:0005886)	1449	2	2.76	0.72	1.00E+00	1.00E+00	CC	5
transferase complex (GO:1990234)	772	1	1.47	0.68	1.00E+00	1.00E+00	CC	5
membrane (GO:0016020)	8074	10	15.37	0.65	1.51E-01	1.00E+00	CC	5
cell periphery (GO:0071944)	1669	2	3.18	0.63	7.72E-01	1.00E+00	CC	5
cellular_component (GO:0005575)	19828	23	37.75	0.61	7.13E-04	1.55E-01	CC	5
cellular anatomical entity (GO:0110165)	19592	22	37.3	0.59	4.52E-04	1.97E-01	CC	5
protein-containing complex (GO:0032991)	4069	4	7.75	0.52	1.85E-01	1.00E+00	CC	5
intracellular membrane-bounded organelle (GO:0043231)	10826	10	20.61	0.49	6.02E-03	5.83E-01	CC	5
membrane-bounded organelle (GO:0043227)	10848	10	20.66	0.48	4.29E-03	4.67E-01	CC	5
chloroplast (GO:0009507)	1128	1	2.15	0.47	7.27E-01	1.00E+00	CC	5
intracellular anatomical structure (GO:0005622)	14114	12	26.87	0.45	2.42E-04	2.10E-01	CC	5
intracellular organelle (GO:0043229)	11979	10	22.81	0.44	9.37E-04	1.36E-01	CC	5
organelle (GO:0043226)	11985	10	22.82	0.44	9.37E-04	1.17E-01	CC	5
intracellular non-membrane-bounded organelle (GO:0043232)	2611	2	4.97	0.4	2.41E-01	1.00E+00	CC	5
non-membrane-bounded organelle (GO:0043228)	2611	2	4.97	0.4	2.41E-01	1.00E+00	CC	5
plastid (GO:0009536)	1326	1	2.52	0.4	5.22E-01	1.00E+00	CC	5
cytoplasm (GO:0005737)	8535	5	16.25	0.31	6.59E-04	1.91E-01	CC	5

Note : Genome, the gene number of some GO term in genome; Count, ribosome paused transcripts unmber of some GO term; Expected, the expected count of ribosome paused transcripts obtained based on the proportion of GO term genes to the genome; Fold enrichment, the ratio between the actual number of enriched genes and the expected value; BP, biological process; MF, molecular function; CC, cellular component.
