# Supplementary material for: Ribosome Pausing Negatively Regulates Protein Translation in Maize Seedlings during Dark-to-Light Transitions
Source: Int J Mol Sci. 2024 Jul 22;25(14):7985. doi: 10.3390/ijms25147985 (PMC11277263; doi:10.3390/ijms25147985)
Supplement: Supplementary file 1 [file ijms-25-07985-s001.zip › Table S4.pdf]

Table S4 KEGG of ribosome paused transcripts

ID	Description	GeneRatio	BgRatio	*P* value	*P* value adjust	qvalue	geneID	Count	Cluster
zma03013	Nucleocytoplasmic transport	1/4	143/6579	0.084168045	0.242336026	0.24233603	100278395	1	1
zma04120	Ubiquitin mediated proteolysis	1/4	170/6579	0.099443363	0.242336026	0.24233603	100282479	1	1
zma03040	Spliceosome	1/4	267/6579	0.152749849	0.242336026	0.24233603	100281694	1	1
zma04075	Plant hormone signal transduction	1/4	345/6579	0.193868821	0.242336026	0.24233603	100286212	1	1
zma03010	Ribosome	1/4	475/6579	0.259052015	0.259052015	0.25905202	100282479	1	1
zma01240	Biosynthesis of cofactors	4/18	296/6579	0.007447075	0.059576596	0.04703416	732837/100280079/103633593/103640501	4	2
zma00940	Phenylpropanoid biosynthesis	3/18	205/6579	0.017220841	0.068883365	0.0543816	100279351/100216881/542029	3	2
zma00790	Folate biosynthesis	2/18	33/6579	0.003550413	0.05680661	0.04484732	100280079/103638882	2	2
zma00053	Ascorbate and aldarate metabolism	2/18	70/6579	0.015296744	0.068883365	0.0543816	103633593/103640501	2	2
zma04141	Protein processing in endoplasmic reticulum	2/18	287/6579	0.183967184	0.420496421	0.33197086	100191719/100194240	2	2
zma00908	Zeatin biosynthesis	1/18	41/6579	0.106556512	0.340980838	0.2691954	103627131	1	2
zma00130	Ubiquinone and other terpenoid-quinone biosynthesis	1/18	53/6579	0.135655	0.361746667	0.28558947	732837	1	2
zma00970	Aminoacyl-tRNA biosynthesis	1/18	101/6579	0.243339123	0.442280271	0.34916864	103635898	1	2
zma01250	Biosynthesis of nucleotide sugars	1/18	114/6579	0.270244871	0.442280271	0.34916864	103633593	1	2
zma00564	Glycerophospholipid metabolism	1/18	130/6579	0.302119812	0.442280271	0.34916864	100272401	1	2
zma03008	Ribosome biogenesis in eukaryotes	1/18	131/6579	0.304067687	0.442280271	0.34916864	103643809	1	2
zma03015	mRNA surveillance pathway	1/18	165/6579	0.367321671	0.469408899	0.37058597	100192067	1	2
zma00520	Amino sugar and nucleotide sugar metabolism	1/18	173/6579	0.381394731	0.469408899	0.37058597	103633593	1	2
zma04016	MAPK signaling pathway - plant	1/18	192/6579	0.413642648	0.472734454	0.37321141	100281832	1	2
zma04626	Plant-pathogen interaction	1/18	246/6579	0.496847311	0.512368704	0.40450161	732824	1	2
zma00190	Oxidative phosphorylation	1/18	257/6579	0.512368704	0.512368704	0.40450161	100280838	1	2
zma00511	Other glycan degradation	1/9	20/6579	0.027045686	0.172456207	0.16136253	103638898	1	3
zma04130	SNARE interactions in vesicular transport	1/9	46/6579	0.06123217	0.172456207	0.16136253	100282605	1	3
zma00592	alpha-Linolenic acid metabolism	1/9	52/6579	0.068968044	0.172456207	0.16136253	732731	1	3

ID	Description	GeneRatio	BgRatio	*P* value	*P* value adjust	qvalue	geneID	Count	Cluster
zma02010	ABC transporters	1/9	58/6579	0.076647203	0.172456207	0.16136253	100286184	1	3
zma03420	Nucleotide excision repair	1/9	95/6579	0.122770497	0.182665364	0.17091496	100192592	1	3
zma00040	Pentose and glucuronate interconversions	1/9	96/6579	0.12398812	0.182665364	0.17091496	100285523	1	3
zma04145	Phagosome	1/9	111/6579	0.142073061	0.182665364	0.17091496	542758	1	3
zma00564	Glycerophospholipid metabolism	1/9	130/6579	0.16450382	0.185066798	0.17316192	100192922	1	3
zma04626	Plant-pathogen interaction	1/9	246/6579	0.290497349	0.290497349	0.27181038	103654474	1	3
zma00908	Zeatin biosynthesis	1/6	41/6579	0.03682774	0.238542856	0.21522664	100272805	1	4
zma03082	ATP-dependent chromatin remodeling	1/6	88/6579	0.077647552	0.238542856	0.21522664	100193816	1	4
zma00270	Cysteine and methionine metabolism	1/6	143/6579	0.12357475	0.238542856	0.21522664	100193096	1	4
zma04016	MAPK signaling pathway - plant	1/6	192/6579	0.162870983	0.238542856	0.21522664	100283932	1	4
zma00940	Phenylpropanoid biosynthesis	1/6	205/6579	0.173046343	0.238542856	0.21522664	100273061	1	4
zma04626	Plant-pathogen interaction	1/6	246/6579	0.204465305	0.238542856	0.21522664	103637688	1	4
zma04075	Plant hormone signal transduction	1/6	345/6579	0.276253228	0.276253228	0.24925103	100283932	1	4
zma00750	Vitamin B6 metabolism	1/6	15/6579	0.013607295	0.072207591	0.03167	100502503	1	5
zma00960	Tropane, piperidine and pyridine alkaloid biosynthesis	1/6	20/6579	0.018108623	0.072207591	0.03167	100279491	1	5
zma00950	Isoquinoline alkaloid biosynthesis	1/6	22/6579	0.019904355	0.072207591	0.03167	100279491	1	5
zma00360	Phenylalanine metabolism	1/6	35/6579	0.031510031	0.072207591	0.03167	100279491	1	5
zma00908	Zeatin biosynthesis	1/6	41/6579	0.03682774	0.072207591	0.03167	542056	1	5
zma00350	Tyrosine metabolism	1/6	43/6579	0.038594893	0.072207591	0.03167	100279491	1	5
zma00410	beta-Alanine metabolism	1/6	47/6579	0.042121094	0.072207591	0.03167	100279491	1	5
zma00900	Terpenoid backbone biosynthesis	1/6	71/6579	0.063052755	0.094579132	0.04148208	100283522	1	5
zma00260	Glycine, serine and threonine metabolism	1/6	86/6579	0.075940414	0.101253886	0.0444096	100279491	1	5
zma03008	Ribosome biogenesis in eukaryotes	1/6	131/6579	0.113720455	0.136464546	0.05985287	107521957	1	5
zma01240	Biosynthesis of cofactors	1/6	296/6579	0.241428775	0.263376845	0.11551616	100502503	1	5
zma04075	Plant hormone signal transduction	1/6	345/6579	0.276253228	0.276253228	0.1211637	103650054	1	5
