# Supplementary material for: Ribosome Pausing Negatively Regulates Protein Translation in Maize Seedlings during Dark-to-Light Transitions
Source: Int J Mol Sci. 2024 Jul 22;25(14):7985. doi: 10.3390/ijms25147985 (PMC11277263; doi:10.3390/ijms25147985)
Supplement: Supplementary file 1 [file ijms-25-07985-s001.zip › Table S5.pdf]

Table S5 The coverage of RPFs on the ribosome paused transcripts

Gene	Max pausing score 0h	Gene coverage 0h	Max pausing score 0.5h	Gene coverage 0.5h	Max pausing score 1h	Gene coverage 1h	Max pausing score 2h	Gene coverage 2h	Max pausing score 4h	Gene coverage 4h
ZeamMp030	511.42	5.00	885.47	3.60	706.08	5.30	801.83	4.50	766.05	21.40
ZeamMp033	782.61	8.90	226.39	5.06	625.00	0.30	285.71	16.19	54.35	10.60
ZeamMp034	400.00	0.40	600.00	0.30	625.00	0.20	560.00	0.50	888.89	0.40
ZeamMp041	1000.00	0.10	600.00	0.20	937.50	0.20	931.03	0.30	368.42	1.10
ZeamMp137	812.50	0.60	452.38	0.90	428.57	0.50	166.67	1.00	48.22	20.10
Zm00001d000035	878.79	0.50	500.00	0.30	150.00	1.20	304.35	1.10	272.73	0.80
Zm00001d000390	843.75	0.50	333.33	0.30	259.26	1.20	100.00	1.60	114.29	2.30
Zm00001d002058	885.71	2.71	333.33	0.99	600.00	1.30	167.00	4.09	90.91	1.80
Zm00001d002086	400.00	0.40	958.33	0.74	500.00	3.64	500.00	1.76	333.33	1.88
Zm00001d002131	900.00	0.40	102.56	2.70	55.56	3.80	200.00	0.40	333.33	0.30
Zm00001d002542	862.07	1.16	500.00	0.35	333.33	5.70	422.22	0.72	333.33	2.72
Zm00001d002684	465.56	3.80	954.55	1.66	750.00	4.84	300.00	3.57	236.37	5.49
Zm00001d002757	857.14	0.40	500.00	0.20	NA	0.00	312.50	0.90	142.86	5.26
Zm00001d002782	812.50	0.40	520.00	0.90	235.29	0.80	333.33	0.50	125.00	1.40
Zm00001d002899	954.55	0.20	166.67	1.60	285.71	0.60	333.33	0.30	333.33	0.30
Zm00001d003088	875.00	0.50	500.00	0.20	750.00	0.20	277.78	0.80	428.57	0.40
Zm00001d003183	NA	0.00	954.55	0.20	NA	0.00	333.33	0.30	NA	0.00
Zm00001d003281	900.00	0.38	333.33	0.30	533.34	1.43	428.57	4.39	142.86	3.47
Zm00001d003399	952.38	1.81	500.00	0.20	503.50	3.38	222.22	10.84	285.71	3.34
Zm00001d003400	875.00	0.40	NA	0.00	461.54	0.60	500.00	0.50	166.67	0.60
Zm00001d003427	828.57	5.94	333.33	3.88	333.33	10.19	222.22	5.99	105.26	7.74
Zm00001d003435	964.29	0.20	NA	0.00	500.00	0.20	400.00	0.40	200.00	0.50
Zm00001d003463	815.79	0.60	142.86	0.70	250.00	1.20	200.00	0.50	NA	0.00

Gene	Max pausing score 0h	Gene coverage 0h	Max pausing score 0.5h	Gene coverage 0.5h	Max pausing score 1h	Gene coverage 1h	Max pausing score 2h	Gene coverage 2h	Max pausing score 4h	Gene coverage 4h
Zm00001d003516	960.00	3.46	333.33	0.20	250.00	0.35	750.00	0.20	142.86	0.35
Zm00001d003538	1000.00	0.10	250.00	0.90	166.67	1.00	333.33	0.40	500.00	0.20
Zm00001d003593	850.00	0.60	250.00	0.90	300.00	1.30	80.00	3.30	142.86	0.90
Zm00001d003743	833.33	0.60	142.86	1.20	150.00	1.60	206.90	1.80	222.22	0.70
Zm00001d003763	925.93	0.30	333.33	0.20	400.00	0.30	500.00	0.30	500.00	0.20
Zm00001d004301	333.33	7.87	947.66	0.26	250.00	0.40	157.89	25.12	166.67	12.27
Zm00001d004310	333.33	0.30	958.33	0.20	666.67	0.20	571.43	0.30	NA	0.00
Zm00001d004910	821.43	0.40	NA	0.00	NA	0.00	545.45	0.50	500.00	0.20
Zm00001d005109	900.00	0.30	500.00	0.20	750.00	0.30	500.00	0.30	250.00	0.80
Zm00001d005480	880.00	1.62	884.62	0.86	250.72	2.86	500.00	0.51	250.00	1.72
Zm00001d005504	828.57	0.60	1000.00	0.10	111.11	0.90	272.73	0.60	333.33	0.30
Zm00001d005612	870.97	0.40	333.33	0.30	83.33	0.70	95.24	0.30	166.67	0.60
Zm00001d005680	1000.00	0.10	500.00	0.20	333.33	0.30	1000.00	0.10	1000.00	0.17
Zm00001d005909	837.65	1.27	747.70	2.84	200.00	3.22	68.53	3.54	142.86	4.52
Zm00001d005936	1000.00	0.10	139.43	1.88	181.82	2.34	250.00	2.29	200.00	0.50
Zm00001d005962	1000.00	0.10	500.00	0.20	NA	0.00	666.67	0.20	NA	0.00
Zm00001d005989	782.61	0.90	769.23	0.40	285.71	1.20	137.93	1.80	150.00	1.30
Zm00001d006000	1000.00	0.10	NA	0.00	NA	0.00	NA	0.00	NA	0.00
Zm00001d006011	848.48	2.01	809.52	1.04	200.00	4.81	533.34	4.32	431.82	1.96
Zm00001d006045	875.00	0.30	500.00	0.20	333.33	0.60	333.33	0.40	285.71	0.60
Zm00001d006132	1000.00	0.10	500.00	0.20	500.00	4.24	518.52	1.43	501.90	3.63
Zm00001d006193	956.52	0.26	NA	0.00	666.67	0.20	857.14	0.20	NA	0.00
Zm00001d006321	900.00	0.40	250.00	0.40	294.12	0.80	142.86	1.50	133.33	1.20
Zm00001d006619	814.81	7.01	703.70	7.39	283.34	6.16	117.65	10.20	306.82	9.33

Gene	Max pausing score 0h	Gene coverage 0h	Max pausing score 0.5h	Gene coverage 0.5h	Max pausing score 1h	Gene coverage 1h	Max pausing score 2h	Gene coverage 2h	Max pausing score 4h	Gene coverage 4h
Zm00001d006631	250.00	4.32	1000.00	0.10	583.33	6.39	666.67	0.81	666.67	0.20
Zm00001d006638	875.00	2.74	250.00	4.40	1000.00	0.17	170.84	11.05	181.82	12.08
Zm00001d006894	1000.00	0.17	500.00	0.40	1000.00	0.10	225.00	2.05	267.86	3.20
Zm00001d006947	956.52	1.92	NA	0.00	333.33	6.24	450.00	1.13	428.57	0.40
Zm00001d006950	311.83	1.70	920.00	0.30	310.34	0.90	137.93	1.80	78.95	2.90
Zm00001d007015	166.67	0.50	888.89	0.40	333.33	0.40	500.00	0.20	500.00	0.20
Zm00001d007050	956.52	0.20	309.52	0.53	230.77	0.80	125.00	6.79	250.00	0.40
Zm00001d007162	785.71	6.52	501.18	1.13	250.00	1.26	333.33	6.15	666.67	0.40
Zm00001d007197	818.18	5.88	200.00	2.56	250.00	1.20	142.86	0.67	250.00	0.75
Zm00001d007258	830.48	3.87	187.50	1.30	125.00	4.48	62.50	6.77	85.49	12.04
Zm00001d007259	794.12	0.80	200.00	0.50	428.57	0.90	125.00	1.50	133.33	1.30
Zm00001d007294	916.67	2.82	500.00	0.20	222.22	3.06	500.00	1.15	142.86	0.36
Zm00001d007478	1000.00	0.10	NA	0.00	NA	0.00	666.67	0.20	250.00	0.40
Zm00001d007503	1000.00	0.10	500.00	0.20	1000.00	0.10	NA	0.00	NA	0.00
Zm00001d007518	NA	0.00	1000.00	0.10	1000.00	0.10	NA	0.00	500.00	0.30
Zm00001d007606	846.15	0.40	NA	0.00	666.67	0.20	500.00	0.35	666.67	1.79
Zm00001d007839	838.71	3.64	90.91	2.93	119.05	2.63	85.71	6.18	166.67	4.93
Zm00001d007869	954.55	3.22	333.33	0.30	384.62	3.64	200.00	1.67	250.00	7.59
Zm00001d008187	906.98	5.29	NA	0.00	500.00	0.86	666.67	3.64	666.67	0.20
Zm00001d008219	1000.00	0.10	NA	0.00	NA	0.00	666.67	0.20	NA	NA
Zm00001d008297	828.57	0.70	181.82	0.90	235.29	1.00	151.52	1.90	105.26	1.50
Zm00001d008298	1000.00	0.10	NA	0.00	1000.00	0.10	500.00	0.30	500.00	7.40
Zm00001d008329	857.14	0.30	100.00	0.80	129.63	2.80	81.63	2.20	95.24	1.40
Zm00001d008409	884.62	1.18	333.33	3.88	416.67	6.25	238.10	2.85	222.22	2.31

Gene	Max pausing score 0h	Gene coverage 0h	Max pausing score 0.5h	Gene coverage 0.5h	Max pausing score 1h	Gene coverage 1h	Max pausing score 2h	Gene coverage 2h	Max pausing score 4h	Gene coverage 4h
Zm00001d008764	810.81	1.26	318.68	3.64	62.58	6.92	37.04	9.48	42.42	9.49
Zm00001d008827	962.96	0.20	666.67	0.20	400.00	1.69	166.67	0.70	111.11	0.98
Zm00001d008859	866.67	0.50	500.00	0.20	187.50	1.00	272.73	0.70	166.67	4.27
Zm00001d009008	931.03	0.90	NA	0.00	500.00	7.81	497.55	5.63	300.00	11.68
Zm00001d009108	931.03	0.30	125.00	3.06	250.00	3.88	215.91	2.75	333.33	3.19
Zm00001d009138	937.50	0.30	333.33	0.30	NA	0.00	1000.00	0.10	500.00	0.20
Zm00001d009336	823.53	0.70	888.89	0.40	142.86	1.10	333.33	0.70	166.67	1.00
Zm00001d009568	846.15	0.50	250.00	0.40	155.56	2.50	105.26	2.30	133.33	1.00
Zm00001d009747	923.08	5.35	222.22	0.64	333.33	0.66	500.00	9.74	250.00	0.70
Zm00001d009787	931.03	0.30	222.22	0.80	142.86	1.10	212.12	1.90	272.73	0.90
Zm00001d010044	878.79	0.40	250.00	0.40	357.14	0.70	133.33	1.60	136.36	1.80
Zm00001d010222	916.67	0.30	764.71	0.30	500.00	0.40	500.00	0.20	1000.00	0.10
Zm00001d010325	880.00	0.30	NA	0.00	NA	0.00	222.22	1.20	181.82	0.80
Zm00001d010388	965.52	0.20	875.00	0.40	571.43	0.30	NA	0.00	NA	0.00
Zm00001d010564	783.78	0.20	166.67	0.20	50.00	1.73	54.10	0.37	46.65	2.60
Zm00001d010590	806.45	5.34	251.75	2.89	363.64	2.60	140.63	6.00	88.46	3.13
Zm00001d010594	1000.00	0.10	166.67	0.60	333.33	0.70	333.33	0.30	333.33	0.30
Zm00001d010610	1000.00	0.10	500.00	0.20	444.44	0.81	600.00	0.55	325.00	1.67
Zm00001d010621	1000.00	0.10	500.00	0.20	NA	0.00	NA	0.00	NA	0.00
Zm00001d010785	920.00	0.30	500.00	0.20	1000.00	0.10	500.00	5.43	333.33	0.40
Zm00001d010788	842.11	0.50	555.56	0.80	157.89	1.10	250.00	0.60	500.00	0.40
Zm00001d010868	969.70	0.48	333.33	2.60	142.86	2.32	222.22	3.24	117.65	4.25
Zm00001d010872	833.33	0.60	250.00	0.70	150.00	1.40	214.29	1.10	200.00	1.20
Zm00001d010925	1000.00	0.10	NA	0.00	500.00	0.30	NA	0.00	NA	0.00

Gene	Max pausing score 0h	Gene coverage 0h	Max pausing score 0.5h	Gene coverage 0.5h	Max pausing score 1h	Gene coverage 1h	Max pausing score 2h	Gene coverage 2h	Max pausing score 4h	Gene coverage 4h
Zm00001d011068	833.33	3.06	185.90	0.73	166.67	1.62	353.85	1.23	500.00	0.20
Zm00001d011620	NA	0.00	916.67	0.20	600.00	0.30	NA	0.00	500.00	0.20
Zm00001d011881	878.38	0.75	666.67	2.37	333.33	0.30	162.59	6.34	600.00	0.43
Zm00001d011890	843.75	0.60	251.36	6.55	500.00	0.20	333.33	0.40	500.00	0.75
Zm00001d011964	159.60	4.92	928.57	1.24	59.30	4.43	54.28	7.51	40.89	4.15
Zm00001d012041	880.00	0.40	NA	0.00	NA	0.00	NA	0.00	NA	0.00
Zm00001d012237	878.79	0.40	NA	0.00	NA	0.00	250.00	0.90	400.00	0.30
Zm00001d012275	826.09	0.66	500.00	0.20	83.33	4.01	250.00	5.28	119.61	3.02
Zm00001d012289	961.54	5.33	285.71	1.50	250.00	2.92	181.82	0.97	333.33	0.66
Zm00001d012387	NA	0.00	1000.00	0.10	NA	0.00	NA	0.00	NA	0.00
Zm00001d012612	781.25	0.62	333.33	2.08	235.29	2.23	171.88	4.19	117.67	3.54
Zm00001d012626	920.00	0.30	NA	0.00	NA	0.00	NA	0.00	NA	0.00
Zm00001d012785	1000.00	0.10	250.00	2.65	307.69	0.60	333.33	0.44	NA	0.00
Zm00001d012934	777.78	6.82	300.00	2.08	400.00	2.68	375.00	1.64	250.00	6.39
Zm00001d013069	333.33	0.40	903.23	0.40	400.00	0.50	214.29	0.80	333.33	0.40
Zm00001d013162	888.89	6.75	188.04	3.70	166.67	7.03	137.93	6.52	113.96	5.97
Zm00001d013311	906.25	0.40	NA	0.00	NA	0.00	750.00	0.20	NA	0.00
Zm00001d013339	838.71	0.60	166.67	0.50	400.00	0.70	192.31	1.60	222.22	1.20
Zm00001d013342	1000.00	0.10	250.00	1.11	333.33	1.35	222.22	2.53	200.00	0.50
Zm00001d013399	843.75	0.60	333.33	0.20	285.71	2.33	187.50	0.90	166.67	3.01
Zm00001d013794	972.97	0.20	1000.00	0.10	500.00	0.70	181.82	4.06	500.00	0.26
Zm00001d013923	861.11	3.02	250.00	0.60	200.00	2.69	238.10	5.77	125.00	2.36
Zm00001d014196	1000.00	0.17	333.33	1.59	444.44	2.43	666.67	0.50	444.44	1.04
Zm00001d014253	NA	0.00	962.96	0.20	101.45	3.20	1000.00	0.10	NA	0.00

Gene	Max pausing score 0h	Gene coverage 0h	Max pausing score 0.5h	Gene coverage 0.5h	Max pausing score 1h	Gene coverage 1h	Max pausing score 2h	Gene coverage 2h	Max pausing score 4h	Gene coverage 4h
Zm00001d014414	964.29	0.20	NA	0.00	428.57	0.40	253.97	2.15	500.00	0.99
Zm00001d014463	939.39	0.30	200.00	0.50	138.89	2.10	150.00	1.50	181.82	1.40
Zm00001d014704	916.67	0.30	200.00	0.30	157.89	1.10	117.65	1.40	333.33	0.80
Zm00001d014820	1000.00	0.17	58.82	5.14	57.69	7.32	34.71	20.48	21.87	18.94
Zm00001d014994	846.15	0.81	125.00	2.59	121.21	4.24	111.11	3.19	125.00	13.09
Zm00001d015059	920.00	5.10	NA	0.00	500.00	0.30	333.33	5.06	334.41	5.44
Zm00001d015129	843.75	2.90	500.00	0.20	500.00	0.20	500.00	0.20	375.00	2.58
Zm00001d015202	1000.00	0.10	NA	0.00	384.56	1.82	NA	0.00	400.00	1.27
Zm00001d015215	961.54	0.20	250.00	0.40	250.00	0.70	129.63	2.40	142.86	1.20
Zm00001d015407	805.56	10.86	250.00	0.70	202.02	14.72	519.23	7.87	333.33	0.30
Zm00001d015412	820.51	0.70	250.00	0.40	242.42	1.70	153.85	1.60	142.86	1.00
Zm00001d015744	400.00	0.40	1000.00	0.10	400.00	0.47	333.33	0.50	255.75	1.05
Zm00001d015779	294.88	1.40	312.08	1.20	500.00	1.30	451.08	1.30	810.73	1.00
Zm00001d015884	1000.00	5.72	500.00	0.20	417.14	4.58	400.00	0.61	142.86	0.50
Zm00001d015990	1000.00	0.15	NA	0.00	333.33	0.30	500.00	0.20	700.00	5.35
Zm00001d016154	900.00	0.40	NA	0.00	444.44	3.57	307.69	0.80	166.67	1.40
Zm00001d016262	1000.00	0.10	NA	0.00	NA	0.00	NA	0.00	NA	NA
Zm00001d016301	941.18	0.30	NA	0.00	625.00	0.20	250.00	0.40	153.85	1.20
Zm00001d016322	1000.00	0.10	1000.00	0.10	400.00	0.50	375.00	0.50	500.00	4.64
Zm00001d016417	880.00	0.40	NA	0.00	333.33	0.20	181.82	0.70	90.91	1.20
Zm00001d016648	1000.00	0.10	NA	0.00	545.45	0.40	375.00	0.50	500.00	0.30
Zm00001d016806	843.75	1.76	NA	0.00	500.00	0.20	363.64	8.92	250.00	6.39
Zm00001d016831	837.84	0.60	125.00	0.80	153.85	1.77	666.67	2.76	333.33	0.50
Zm00001d016844	1000.00	0.10	NA	0.00	500.00	0.30	200.42	4.32	286.37	1.74

Gene	Max pausing score 0h	Gene coverage 0h	Max pausing score 0.5h	Gene coverage 0.5h	Max pausing score 1h	Gene coverage 1h	Max pausing score 2h	Gene coverage 2h	Max pausing score 4h	Gene coverage 4h
Zm00001d016896	852.94	4.33	500.00	1.73	444.44	2.36	307.69	1.48	250.00	2.56
Zm00001d017351	807.69	0.50	800.00	0.50	260.87	1.30	157.89	1.20	285.71	0.50
Zm00001d017353	166.67	0.60	913.04	0.30	500.00	0.40	428.57	0.40	214.29	4.24
Zm00001d017462	842.11	0.50	333.33	0.30	518.52	0.70	571.43	0.40	500.00	0.30
Zm00001d017530	862.07	0.50	333.33	0.30	312.50	0.90	250.00	0.80	125.00	1.50
Zm00001d017696	809.52	0.40	250.00	0.20	151.52	1.00	142.86	0.50	111.11	0.20
Zm00001d017746	956.52	0.20	100.00	5.14	230.77	6.99	119.05	9.12	76.92	9.29
Zm00001d017851	800.00	0.70	821.43	0.50	380.95	0.70	173.91	1.10	NA	NA
Zm00001d017991	971.43	0.20	NA	0.00	250.00	0.50	NA	0.00	NA	0.00
Zm00001d018058	1000.00	0.10	1000.00	0.10	333.72	12.93	200.00	0.50	285.71	7.74
Zm00001d018081	846.15	0.50	470.59	0.80	166.67	1.20	176.47	1.20	32.79	4.10
Zm00001d018117	NA	0.00	1000.00	0.10	NA	0.00	NA	0.00	NA	0.00
Zm00001d018133	665.55	4.56	313.36	4.57	359.07	6.46	615.25	4.16	794.86	15.79
Zm00001d018191	333.33	0.20	962.96	0.20	200.00	0.40	NA	0.00	250.00	0.20
Zm00001d018475	956.52	5.88	NA	0.00	334.73	1.74	500.00	1.43	500.00	1.11
Zm00001d018696	971.43	0.88	700.00	6.21	263.16	3.24	400.00	3.10	142.86	5.15
Zm00001d018806	954.55	0.20	166.67	7.31	300.00	1.11	307.69	0.60	181.82	5.47
Zm00001d019002	864.86	0.30	166.67	0.60	300.00	0.70	100.00	1.50	400.00	0.30
Zm00001d019400	931.03	0.30	500.00	0.20	500.00	0.30	375.00	0.60	500.00	0.30
Zm00001d019422	900.00	0.40	500.00	0.20	625.00	4.67	333.33	0.30	250.00	3.87
Zm00001d019582	1000.00	0.10	NA	0.00	666.67	0.20	NA	0.00	333.33	0.30
Zm00001d019669	550.35	7.99	916.67	0.30	166.67	4.11	78.55	5.28	76.92	4.63
Zm00001d019925	954.55	0.20	NA	0.00	1000.00	0.10	NA	0.00	NA	0.00
Zm00001d019989	974.36	0.20	500.00	1.40	555.56	0.40	416.67	0.50	428.57	0.40

Gene	Max pausing score 0h	Gene coverage 0h	Max pausing score 0.5h	Gene coverage 0.5h	Max pausing score 1h	Gene coverage 1h	Max pausing score 2h	Gene coverage 2h	Max pausing score 4h	Gene coverage 4h
Zm00001d019990	913.04	0.30	NA	0.00	NA	0.00	333.33	0.30	250.00	0.60
Zm00001d020176	1000.00	0.10	NA	0.00	692.31	0.50	NA	0.00	506.14	1.18
Zm00001d020277	414.18	0.71	430.00	2.53	577.71	3.53	697.74	1.88	333.33	2.60
Zm00001d020403	1000.00	0.10	225.00	1.50	333.33	1.60	285.71	5.52	222.22	2.64
Zm00001d020425	807.69	0.40	346.15	0.20	190.48	0.70	250.00	1.00	400.00	0.40
Zm00001d020497	241.76	5.67	892.86	0.40	384.62	1.49	181.82	4.27	62.67	4.87
Zm00001d020610	833.33	0.60	133.33	1.30	64.94	4.60	65.04	7.90	33.61	7.60
Zm00001d020620	805.56	0.60	181.82	0.90	285.71	0.90	214.29	1.20	166.67	6.35
Zm00001d020651	896.55	0.30	NA	0.00	666.67	0.30	333.33	0.60	250.00	0.90
Zm00001d020909	1000.00	0.10	1000.00	0.10	NA	0.00	NA	0.00	NA	0.00
Zm00001d021024	888.89	0.51	200.00	6.60	600.00	0.88	227.42	9.14	181.82	2.21
Zm00001d021216	1000.00	0.10	166.67	0.64	71.43	5.91	84.75	3.93	78.95	8.20
Zm00001d021294	1000.00	0.10	NA	0.00	500.00	0.20	200.00	0.50	NA	0.00
Zm00001d021338	905.09	4.49	200.00	1.27	318.18	4.68	103.45	6.41	208.34	7.13
Zm00001d021439	1000.00	0.10	1000.00	0.10	600.00	0.30	NA	0.00	500.00	0.20
Zm00001d021576	812.50	3.28	NA	0.00	428.57	8.60	600.00	3.80	333.33	7.24
Zm00001d021744	862.07	0.53	869.57	0.40	333.33	0.60	428.57	20.05	166.67	0.60
Zm00001d021778	823.53	0.60	NA	0.00	285.71	1.00	285.71	0.60	250.00	0.40
Zm00001d021967	961.54	0.20	NA	0.00	333.33	0.40	125.00	1.10	136.36	1.60
Zm00001d021995	848.48	0.30	333.33	0.30	500.00	0.30	200.00	0.40	333.33	0.30
Zm00001d022040	920.00	1.58	400.00	0.44	278.35	1.12	183.34	1.49	238.10	1.61
Zm00001d022045	785.71	0.57	183.34	1.43	250.00	0.36	230.77	0.88	187.50	1.10
Zm00001d022067	1000.00	0.10	NA	0.00	333.33	0.50	NA	0.00	500.00	0.20
Zm00001d022265	958.33	0.20	250.00	0.40	666.67	0.46	353.29	1.90	200.00	0.40

Gene	Max pausing score 0h	Gene coverage 0h	Max pausing score 0.5h	Gene coverage 0.5h	Max pausing score 1h	Gene coverage 1h	Max pausing score 2h	Gene coverage 2h	Max pausing score 4h	Gene coverage 4h
Zm00001d022350	960.00	0.20	NA	0.00	NA	0.00	NA	0.00	666.67	0.20
Zm00001d022474	807.69	0.50	285.71	0.60	250.00	0.80	166.67	2.20	235.29	3.11
Zm00001d022529	900.00	5.23	NA	0.00	666.67	0.20	202.43	1.50	176.47	2.20
Zm00001d023240	272.73	5.18	1000.00	0.10	333.33	0.40	374.34	2.86	500.00	1.59
Zm00001d023253	966.67	0.44	241.17	1.91	416.67	6.04	1000.00	0.17	250.00	3.65
Zm00001d023291	818.18	3.12	176.47	1.75	185.19	4.21	142.86	7.58	200.00	4.35
Zm00001d023300	1000.00	0.23	333.33	0.30	NA	0.00	83.34	1.34	99.21	1.81
Zm00001d023312	925.93	0.30	333.33	0.30	NA	0.00	375.00	0.60	333.33	0.70
Zm00001d023396	892.86	0.40	666.67	0.20	285.71	0.60	333.33	0.60	333.33	0.30
Zm00001d023455	875.00	0.40	500.00	0.20	250.00	0.40	300.00	0.70	333.33	0.50
Zm00001d023654	897.44	0.33	250.00	0.40	400.00	8.14	300.00	5.64	333.33	6.61
Zm00001d023700	NA	0.00	960.00	0.20	NA	0.00	250.00	0.70	NA	0.00
Zm00001d023767	793.10	0.60	200.00	0.50	400.00	0.60	250.00	0.80	500.00	0.30
Zm00001d024088	838.71	1.17	285.71	3.75	208.34	11.10	400.00	3.33	250.00	6.20
Zm00001d024253	1000.00	0.10	500.00	0.20	714.29	0.30	333.33	0.20	142.86	0.60
Zm00001d024322	813.95	0.70	250.00	0.40	222.22	0.70	125.00	1.60	176.47	1.20
Zm00001d024324	NA	0.00	956.52	0.20	333.33	0.50	500.00	0.30	333.33	0.30
Zm00001d024327	777.78	12.43	714.29	3.11	272.73	4.72	69.77	12.49	111.11	9.37
Zm00001d024647	933.33	2.96	216.67	1.84	222.22	7.85	165.22	6.41	353.85	4.58
Zm00001d024681	862.07	2.72	500.00	0.20	1000.00	1.45	500.00	5.38	236.11	5.55
Zm00001d024687	1000.00	0.52	400.00	3.26	83.33	3.98	185.19	12.08	166.67	5.47
Zm00001d024717	1000.00	0.10	NA	0.00	NA	0.00	1000.00	0.10	NA	0.00
Zm00001d024768	875.00	5.96	400.00	1.60	333.33	0.50	NA	0.00	333.33	0.30
Zm00001d024823	840.00	2.43	333.33	3.96	363.64	1.46	428.57	9.61	160.26	2.73

Gene	Max pausing score 0h	Gene coverage 0h	Max pausing score 0.5h	Gene coverage 0.5h	Max pausing score 1h	Gene coverage 1h	Max pausing score 2h	Gene coverage 2h	Max pausing score 4h	Gene coverage 4h
Zm00001d024873	1000.00	0.10	500.00	0.20	400.00	0.30	NA	0.00	666.67	3.89
Zm00001d025027	250.00	9.36	896.55	2.64	30.30	4.03	27.66	5.46	583.34	2.20
Zm00001d025040	925.93	0.30	NA	0.00	1000.00	0.10	1000.00	0.10	NA	0.00
Zm00001d025247	851.85	0.50	700.00	0.40	111.11	0.90	250.00	0.80	117.65	0.80
Zm00001d025656	954.55	10.67	636.36	17.08	466.67	1.46	221.62	2.45	303.03	16.85
Zm00001d025746	769.23	4.70	902.44	0.41	500.00	0.77	416.67	4.28	200.00	7.72
Zm00001d025804	928.57	14.73	500.00	0.20	176.47	1.10	105.26	1.52	103.71	6.55
Zm00001d025807	785.71	0.96	414.93	11.98	428.57	7.51	148.55	11.07	153.41	7.58
Zm00001d025842	947.37	0.30	500.00	0.20	208.33	1.30	125.00	1.90	181.82	0.90
Zm00001d026032	807.69	10.48	NA	0.00	NA	0.00	NA	0.00	166.67	0.50
Zm00001d026397	214.29	5.65	958.33	0.40	600.00	1.62	176.47	1.89	95.24	1.87
Zm00001d026406	1000.00	0.10	666.67	0.20	400.00	0.50	500.00	0.30	500.00	0.30
Zm00001d026592	NA	0.00	913.04	0.25	500.00	0.20	1000.00	0.10	NA	0.00
Zm00001d027292	851.85	4.93	333.33	0.30	333.33	10.21	222.22	6.32	300.00	3.51
Zm00001d027308	843.75	0.40	NA	0.00	NA	0.00	714.29	0.24	350.00	0.55
Zm00001d027338	947.37	0.30	NA	0.00	222.22	0.30	333.33	0.30	500.00	0.20
Zm00001d027472	225.00	8.64	961.54	8.78	217.56	7.01	166.91	4.88	236.11	7.87
Zm00001d027530	804.88	0.20	285.82	2.29	416.67	1.43	NA	0.00	333.33	0.30
Zm00001d027622	937.50	1.34	500.00	1.05	400.00	6.65	416.67	4.58	NA	0.00
Zm00001d027673	1000.00	0.10	944.44	1.04	750.00	11.37	285.71	7.86	1000.00	0.34
Zm00001d027741	956.52	0.20	500.00	0.20	NA	0.00	282.05	4.51	300.00	0.70
Zm00001d027751	1000.00	0.17	500.00	0.20	333.33	3.85	174.25	3.08	285.71	5.25
Zm00001d027946	944.44	0.20	333.33	0.30	800.00	0.20	100.00	10.94	100.00	9.65
Zm00001d028004	943.74	6.84	487.50	10.01	307.69	12.78	333.33	16.61	206.67	2.92

Gene	Max pausing score 0h	Gene coverage 0h	Max pausing score 0.5h	Gene coverage 0.5h	Max pausing score 1h	Gene coverage 1h	Max pausing score 2h	Gene coverage 2h	Max pausing score 4h	Gene coverage 4h
Zm00001d028025	1000.00	0.10	200.00	0.40	NA	0.00	375.00	0.50	117.65	1.30
Zm00001d028073	903.23	0.20	NA	0.00	250.00	0.20	NA	0.00	333.33	0.25
Zm00001d028362	900.00	0.40	250.00	0.40	NA	0.00	444.44	0.30	500.00	0.20
Zm00001d028427	785.71	0.92	264.36	4.36	114.04	2.47	129.03	3.93	74.07	4.09
Zm00001d028447	965.52	0.20	500.00	0.20	800.00	0.20	200.00	1.93	142.86	1.26
Zm00001d028615	1000.00	0.10	NA	0.00	NA	0.00	NA	0.00	NA	0.00
Zm00001d028697	916.67	0.30	500.00	0.20	500.00	0.20	800.00	4.57	666.67	0.20
Zm00001d028714	1000.00	0.10	200.00	0.40	181.82	0.60	260.87	1.00	142.86	1.00
Zm00001d028835	954.55	0.20	500.00	0.20	666.67	0.20	181.82	0.50	NA	0.00
Zm00001d028899	1000.00	0.10	NA	0.00	NA	0.00	NA	0.00	NA	0.00
Zm00001d028925	1000.00	0.10	NA	0.00	NA	0.00	NA	0.00	NA	0.00
Zm00001d029059	1000.00	0.81	500.00	1.57	347.62	2.97	307.69	2.49	203.95	2.89
Zm00001d029241	935.48	2.04	666.67	0.20	214.94	6.83	125.00	5.87	105.26	3.69
Zm00001d029257	833.33	0.60	NA	0.00	428.57	0.40	1000.00	0.10	150.00	1.50
Zm00001d029402	807.69	0.40	166.67	0.60	227.27	1.00	230.77	1.00	83.33	1.20
Zm00001d029427	962.96	0.20	NA	0.00	333.33	0.30	NA	0.00	NA	0.00
Zm00001d029579	868.42	0.50	333.33	0.30	272.73	0.70	333.33	0.60	285.71	0.50
Zm00001d029676	1000.00	0.10	NA	0.00	500.00	0.20	428.57	0.40	333.33	0.30
Zm00001d029921	777.78	2.09	NA	0.00	500.00	0.20	333.33	0.40	714.29	3.57
Zm00001d029950	966.67	0.20	NA	0.00	125.00	0.80	500.00	0.30	400.00	0.30
Zm00001d029969	1000.00	0.10	400.00	0.40	285.71	0.80	266.67	0.80	250.00	0.70
Zm00001d030016	1000.00	0.38	416.67	5.17	500.00	11.15	875.00	0.18	600.00	0.57
Zm00001d030299	960.00	0.20	NA	0.00	501.93	6.30	333.33	0.30	416.67	9.12
Zm00001d030305	954.55	0.20	NA	0.00	375.00	0.50	272.73	0.80	166.67	0.60

Gene	Max pausing score 0h	Gene coverage 0h	Max pausing score 0.5h	Gene coverage 0.5h	Max pausing score 1h	Gene coverage 1h	Max pausing score 2h	Gene coverage 2h	Max pausing score 4h	Gene coverage 4h
Zm00001d030470	862.07	0.50	750.00	0.50	500.00	0.40	222.22	0.60	333.33	0.70
Zm00001d030661	703.70	2.97	958.33	0.20	500.00	1.53	333.33	1.25	182.15	2.03
Zm00001d030775	107.69	3.90	1000.00	0.10	113.64	2.10	45.98	3.70	50.63	4.90
Zm00001d030851	807.69	0.56	333.33	0.30	193.55	3.61	150.38	3.03	200.00	2.26
Zm00001d030877	956.52	0.20	NA	0.00	NA	0.00	NA	0.00	NA	0.00
Zm00001d030942	1000.00	0.10	920.00	3.64	400.00	0.40	500.32	1.61	500.00	1.11
Zm00001d031189	500.00	0.20	1000.00	0.10	500.00	0.30	NA	0.00	NA	0.00
Zm00001d031230	818.18	0.61	782.61	2.72	500.00	3.72	500.38	9.44	250.00	1.50
Zm00001d031454	965.52	0.41	333.33	8.13	666.67	3.45	333.33	0.75	500.00	5.44
Zm00001d031465	666.67	0.20	1000.00	0.10	350.00	1.00	428.57	0.50	NA	0.00
Zm00001d031533	971.43	0.26	333.33	0.30	666.67	0.33	314.29	3.54	500.00	0.33
Zm00001d031717	884.62	0.40	676.47	1.00	166.67	1.50	193.55	1.80	100.00	1.80
Zm00001d031730	916.67	0.56	1000.00	0.43	200.00	2.50	130.43	4.75	81.08	4.46
Zm00001d031782	1000.00	0.10	NA	0.00	833.34	0.18	NA	0.00	666.67	0.20
Zm00001d031858	967.74	0.20	250.00	2.63	500.00	0.20	750.00	1.39	545.45	5.22
Zm00001d032274	884.62	2.54	509.26	0.68	1000.00	0.25	500.39	4.15	714.29	0.99
Zm00001d032324	787.88	0.80	222.22	0.70	388.89	0.80	166.67	0.90	666.67	0.20
Zm00001d032433	958.33	0.20	NA	0.00	500.00	0.26	500.75	6.38	502.38	7.48
Zm00001d032439	888.89	0.70	500.00	0.20	500.00	0.20	333.33	0.30	196.72	1.60
Zm00001d032496	842.11	0.60	111.11	1.40	173.91	0.63	181.82	0.53	117.65	0.96
Zm00001d032587	862.07	0.25	142.86	0.50	375.00	1.49	125.00	2.93	222.22	2.16
Zm00001d032616	142.86	0.20	960.00	0.20	200.00	0.20	NA	0.00	NA	0.00
Zm00001d032858	967.74	0.20	222.22	2.21	571.43	0.36	NA	0.00	NA	0.00
Zm00001d032870	914.29	5.25	535.72	1.36	375.00	0.86	416.67	3.75	500.00	9.72

Gene	Max pausing score 0h	Gene coverage 0h	Max pausing score 0.5h	Gene coverage 0.5h	Max pausing score 1h	Gene coverage 1h	Max pausing score 2h	Gene coverage 2h	Max pausing score 4h	Gene coverage 4h
Zm00001d032978	941.18	0.33	500.00	0.20	333.33	2.04	333.33	10.29	333.33	5.77
Zm00001d033068	833.34	0.19	920.00	0.24	382.81	1.26	600.00	0.70	NA	0.00
Zm00001d033139	1000.00	0.10	NA	0.00	NA	0.00	500.00	0.20	NA	0.00
Zm00001d033374	1000.00	0.10	NA	0.00	500.00	0.30	500.00	0.20	333.33	0.30
Zm00001d033505	827.59	1.56	250.00	4.48	269.01	2.34	176.47	2.71	250.00	3.21
Zm00001d033583	1000.00	0.10	NA	0.00	500.00	0.20	NA	0.00	NA	0.00
Zm00001d033595	800.00	0.50	NA	0.00	571.43	0.30	285.71	0.60	222.22	0.80
Zm00001d033836	875.00	2.36	117.65	1.82	227.27	5.35	166.67	4.50	225.00	0.95
Zm00001d033879	793.10	0.60	787.88	1.46	307.69	1.95	153.85	1.06	333.33	1.48
Zm00001d034064	813.95	0.90	609.76	1.30	103.45	1.90	166.67	2.10	100.00	2.80
Zm00001d034068	843.75	1.49	347.73	3.83	200.00	4.80	222.22	2.25	300.00	3.27
Zm00001d034501	818.18	6.14	291.21	5.68	93.39	4.76	133.33	2.25	150.49	2.85
Zm00001d034738	916.67	1.10	400.00	0.93	285.71	1.81	166.67	6.00	285.71	1.20
Zm00001d034888	333.37	21.15	960.00	0.20	1000.00	0.10	NA	0.00	500.00	0.20
Zm00001d035020	870.97	0.30	NA	0.00	333.33	0.60	181.82	0.60	250.00	0.40
Zm00001d035163	862.07	2.09	166.67	0.53	181.82	0.70	181.82	0.30	250.00	0.20
Zm00001d035322	896.55	0.40	NA	0.00	NA	0.00	666.67	0.20	333.33	0.30
Zm00001d035383	892.86	0.40	250.00	0.40	500.00	0.40	333.33	0.30	NA	0.00
Zm00001d035457	800.00	0.20	920.00	0.20	1000.00	0.10	NA	0.00	NA	0.00
Zm00001d035592	925.93	0.30	NA	0.00	444.44	2.15	250.00	7.04	600.00	0.55
Zm00001d035963	868.42	0.50	333.33	0.30	307.69	0.70	500.00	0.20	111.11	0.80
Zm00001d036152	892.86	0.40	500.00	0.20	666.67	0.20	333.33	0.80	250.00	0.40
Zm00001d036532	916.67	12.53	166.67	13.94	500.00	7.36	357.14	4.34	166.67	1.00
Zm00001d036550	100.00	0.50	916.67	0.30	333.33	0.40	166.67	0.70	250.00	0.40

Gene	Max pausing score 0h	Gene coverage 0h	Max pausing score 0.5h	Gene coverage 0.5h	Max pausing score 1h	Gene coverage 1h	Max pausing score 2h	Gene coverage 2h	Max pausing score 4h	Gene coverage 4h
Zm00001d036615	793.10	0.70	714.29	0.70	250.00	0.60	1000.00	0.10	333.33	0.50
Zm00001d036690	777.78	0.60	NA	0.00	NA	0.00	1000.00	0.10	500.00	0.30
Zm00001d036946	956.52	0.20	NA	0.00	333.33	0.30	666.67	0.20	NA	0.00
Zm00001d037017	892.86	6.09	500.00	0.20	428.57	2.77	333.33	4.68	266.67	11.36
Zm00001d037182	964.29	0.20	500.00	0.30	500.00	0.20	400.00	1.45	NA	0.00
Zm00001d037197	960.00	0.20	500.00	0.20	666.67	0.20	NA	0.00	NA	0.00
Zm00001d037666	785.30	1.14	333.33	0.87	300.00	2.64	183.34	1.78	166.67	3.08
Zm00001d037799	880.00	0.40	400.00	0.40	166.67	0.50	285.71	0.60	444.44	0.60
Zm00001d037958	875.00	0.40	625.00	0.40	250.00	0.40	375.00	0.50	666.67	0.20
Zm00001d038067	965.52	0.20	500.00	0.20	400.00	0.30	666.67	0.20	500.00	0.30
Zm00001d038117	250.00	0.80	920.00	0.30	166.67	0.80	150.00	1.00	250.00	0.70
Zm00001d038281	966.67	0.20	333.33	0.30	222.22	0.80	241.38	1.40	166.67	1.20
Zm00001d038342	920.00	0.30	500.00	0.30	166.67	1.79	166.67	0.99	78.25	9.37
Zm00001d038485	976.19	2.32	500.00	3.12	410.26	8.54	500.00	14.08	255.28	11.14
Zm00001d038626	285.71	0.60	958.33	0.20	750.00	0.20	500.00	0.30	222.22	0.70
Zm00001d038645	1000.00	0.10	NA	0.00	1000.00	0.10	500.00	0.20	NA	0.00
Zm00001d038793	1000.00	0.10	250.00	0.40	214.29	0.90	NA	0.00	1000.00	0.17
Zm00001d038850	854.55	0.80	515.15	1.00	285.71	0.80	235.29	0.80	200.00	1.72
Zm00001d038937	875.00	5.06	100.00	2.06	157.89	8.26	164.99	3.62	250.00	1.53
Zm00001d038960	880.00	0.40	142.86	1.20	125.00	0.80	75.00	2.40	162.50	3.17
Zm00001d039057	937.50	0.30	NA	0.00	NA	0.00	NA	0.00	NA	0.00
Zm00001d039101	1000.00	0.10	NA	0.00	NA	0.00	NA	0.00	571.43	0.40
Zm00001d039103	838.71	0.60	NA	0.00	500.00	0.30	785.71	0.40	142.86	0.70
Zm00001d039132	1000.00	0.10	NA	0.00	NA	0.00	600.00	0.20	NA	0.00

Gene	Max pausing score 0h	Gene coverage 0h	Max pausing score 0.5h	Gene coverage 0.5h	Max pausing score 1h	Gene coverage 1h	Max pausing score 2h	Gene coverage 2h	Max pausing score 4h	Gene coverage 4h
Zm00001d039219	896.55	0.40	NA	0.00	400.00	0.40	666.67	0.20	500.00	10.42
Zm00001d039240	882.35	0.50	NA	0.00	375.00	0.50	160.00	1.40	90.91	1.80
Zm00001d039310	828.57	4.17	166.67	5.72	375.00	2.21	250.00	8.17	83.33	7.79
Zm00001d039510	1000.00	0.15	NA	0.00	NA	0.00	NA	0.00	500.00	0.20
Zm00001d039579	954.55	0.20	NA	0.00	500.00	0.20	800.00	0.20	285.71	8.05
Zm00001d039637	381.77	3.79	890.11	0.99	571.43	9.86	422.22	2.36	243.75	3.84
Zm00001d039642	1000.00	0.10	140.39	4.63	181.82	3.67	333.33	4.94	NA	0.00
Zm00001d039694	1000.00	0.10	NA	0.00	NA	0.00	NA	0.00	NA	0.00
Zm00001d039946	228.07	6.05	916.67	0.53	101.69	5.02	64.52	3.93	83.33	4.93
Zm00001d040047	500.00	0.35	961.54	0.27	167.86	2.33	125.00	1.87	250.00	4.34
Zm00001d040112	827.59	0.50	250.00	0.40	352.94	1.00	176.47	0.90	166.67	1.10
Zm00001d040220	935.48	0.27	NA	0.00	400.00	0.30	500.00	0.35	200.00	5.42
Zm00001d040541	857.14	0.40	500.00	0.20	750.00	0.20	166.67	0.60	333.33	0.30
Zm00001d040544	794.87	0.60	500.00	0.20	260.87	1.10	300.00	0.60	333.33	0.50
Zm00001d040628	916.67	0.30	1000.00	0.10	NA	0.00	NA	0.00	428.57	0.40
Zm00001d040702	785.71	1.63	160.72	2.55	141.44	6.05	176.47	8.35	127.72	4.93
Zm00001d040724	857.14	2.30	500.00	0.25	500.00	0.27	250.00	2.90	200.00	1.48
Zm00001d040743	973.69	0.24	500.00	0.26	500.00	1.84	666.67	0.33	500.34	10.88
Zm00001d041774	250.00	0.40	931.03	0.30	NA	0.00	666.67	0.20	NA	0.00
Zm00001d042169	777.78	0.60	500.00	0.20	625.00	0.40	250.00	0.40	NA	0.00
Zm00001d042508	840.91	3.76	250.00	1.94	142.86	3.51	117.65	9.62	83.33	4.46
Zm00001d042676	427.63	3.65	920.00	9.54	214.29	7.64	111.11	7.93	200.00	2.39
Zm00001d042765	1000.00	0.10	NA	0.00	750.00	0.20	222.22	0.80	500.00	0.30
Zm00001d042766	900.00	0.40	500.00	0.20	800.00	0.20	500.00	0.20	166.67	0.50

Gene	Max pausing score 0h	Gene coverage 0h	Max pausing score 0.5h	Gene coverage 0.5h	Max pausing score 1h	Gene coverage 1h	Max pausing score 2h	Gene coverage 2h	Max pausing score 4h	Gene coverage 4h
Zm00001d042801	880.00	0.30	714.29	2.43	250.00	0.70	500.00	0.30	250.00	10.71
Zm00001d042909	857.14	0.50	200.00	0.90	214.29	1.60	333.33	0.70	500.00	0.20
Zm00001d042936	1000.00	0.10	NA	0.00	NA	0.00	NA	0.00	NA	0.00
Zm00001d043145	814.81	0.60	125.00	0.80	333.33	0.70	130.43	1.60	200.00	0.90
Zm00001d043234	1000.00	0.10	NA	0.00	666.67	0.20	666.67	0.20	666.67	0.20
Zm00001d043293	793.10	2.18	333.33	0.50	126.11	2.66	333.33	6.57	500.00	0.69
Zm00001d043339	806.45	0.20	200.00	0.20	142.86	1.00	111.11	0.60	100.00	0.70
Zm00001d043391	965.52	0.20	250.00	0.40	250.00	0.40	500.00	0.30	1000.00	0.10
Zm00001d043400	875.00	0.63	333.33	0.66	300.00	1.58	333.33	0.30	NA	0.00
Zm00001d043558	945.95	0.30	NA	0.00	500.00	0.30	400.00	0.40	666.67	0.20
Zm00001d043596	961.54	0.20	NA	0.00	NA	0.00	500.00	0.40	NA	0.00
Zm00001d043702	923.08	0.20	900.00	0.40	350.00	1.10	500.00	0.50	666.67	0.20
Zm00001d043751	1000.00	0.10	NA	0.00	333.33	0.30	400.00	0.20	NA	0.00
Zm00001d043870	851.85	1.37	607.76	1.83	590.28	3.97	331.20	2.76	500.00	3.73
Zm00001d044052	789.47	0.90	NA	0.00	750.00	0.20	357.14	0.70	285.71	0.60
Zm00001d044059	954.55	0.20	500.00	0.26	251.15	7.53	256.45	13.49	666.67	0.20
Zm00001d044202	961.54	12.12	1000.00	0.15	NA	0.00	428.57	5.31	181.82	12.16
Zm00001d044417	971.43	0.20	222.22	0.80	187.50	1.00	97.56	2.30	143.48	5.16
Zm00001d044515	860.47	0.60	333.33	0.30	363.64	0.60	333.33	0.50	250.00	0.40
Zm00001d044597	821.43	2.30	250.11	5.59	500.00	3.81	153.85	15.98	500.00	2.29
Zm00001d044802	690.48	1.20	928.57	0.30	461.54	0.50	222.22	0.80	125.00	1.30
Zm00001d044874	1000.00	0.10	500.00	0.20	NA	0.00	NA	0.00	NA	0.00
Zm00001d044895	954.55	0.20	500.00	0.20	454.55	0.40	225.00	2.66	666.67	1.59
Zm00001d044911	1000.00	0.10	NA	0.00	NA	0.00	NA	0.00	NA	0.00

Gene	Max pausing score 0h	Gene coverage 0h	Max pausing score 0.5h	Gene coverage 0.5h	Max pausing score 1h	Gene coverage 1h	Max pausing score 2h	Gene coverage 2h	Max pausing score 4h	Gene coverage 4h
Zm00001d044918	962.96	0.20	800.00	0.20	312.50	1.69	333.33	0.30	NA	0.00
Zm00001d045370	777.78	0.90	333.33	0.30	200.00	0.90	210.53	0.80	NA	0.00
Zm00001d045495	1000.00	0.10	NA	0.00	285.71	0.50	300.00	0.70	666.67	0.20
Zm00001d045515	880.00	0.20	500.00	0.20	500.00	0.20	404.04	7.16	291.67	2.35
Zm00001d045598	913.04	0.26	500.00	0.20	750.00	0.50	250.00	6.21	333.33	3.08
Zm00001d045755	1000.00	1.50	500.00	6.99	250.00	6.06	293.65	4.07	333.33	4.55
Zm00001d045788	864.86	6.71	151.70	2.08	142.86	11.35	162.16	7.56	200.00	1.09
Zm00001d045888	903.23	0.40	NA	0.00	583.33	0.50	285.71	0.80	500.00	0.40
Zm00001d045913	882.35	0.30	1000.00	0.10	333.33	0.20	625.00	0.40	500.00	0.30
Zm00001d046383	931.03	2.71	NA	0.00	400.00	0.36	125.58	17.31	163.24	7.71
Zm00001d046501	846.15	0.40	NA	0.00	411.76	0.50	250.00	0.90	150.00	1.30
Zm00001d046621	888.89	12.88	NA	0.00	500.00	1.39	130.43	0.70	400.00	5.34
Zm00001d046672	835.62	4.36	142.86	4.35	172.41	6.56	236.37	6.15	87.31	5.45
Zm00001d046696	1000.00	0.10	NA	0.00	NA	0.00	500.00	0.20	500.00	0.20
Zm00001d046729	880.00	0.40	NA	0.00	533.33	0.60	200.00	0.50	500.00	0.20
Zm00001d046743	875.00	0.40	562.50	1.30	250.00	1.50	74.07	2.20	300.00	1.30
Zm00001d046759	846.15	0.32	121.43	0.81	NA	0.00	178.57	5.13	153.85	2.28
Zm00001d046882	935.48	0.55	333.33	0.30	454.55	1.42	428.57	1.65	200.00	6.04
Zm00001d046947	777.78	2.21	500.00	0.26	250.00	0.73	194.45	11.24	166.67	6.26
Zm00001d046979	894.02	4.78	250.00	9.04	250.00	8.24	363.64	6.58	333.33	6.52
Zm00001d047013	NA	0.00	1000.00	0.10	750.00	0.20	NA	0.00	NA	0.00
Zm00001d047069	954.55	0.20	NA	0.00	NA	0.00	NA	0.00	250.00	0.90
Zm00001d047202	783.78	0.60	500.00	0.30	400.00	0.40	166.67	0.60	200.00	0.50
Zm00001d047499	888.89	0.30	NA	0.00	600.00	0.30	1000.00	0.10	500.00	0.20

Gene	Max pausing score 0h	Gene coverage 0h	Max pausing score 0.5h	Gene coverage 0.5h	Max pausing score 1h	Gene coverage 1h	Max pausing score 2h	Gene coverage 2h	Max pausing score 4h	Gene coverage 4h
Zm00001d047637	1000.00	0.10	1000.00	0.10	1000.00	0.10	750.00	3.70	500.00	0.20
Zm00001d047708	1000.00	0.10	NA	0.00	NA	0.00	NA	0.00	333.33	0.30
Zm00001d047753	1000.00	0.10	500.00	0.20	NA	0.00	NA	0.00	666.67	0.20
Zm00001d047921	909.09	0.40	NA	0.00	NA	0.00	1000.00	0.10	333.33	0.30
Zm00001d047937	800.00	1.71	403.23	1.57	116.40	5.00	103.45	4.18	166.67	2.56
Zm00001d048032	NA	0.00	1000.00	0.10	NA	0.00	NA	0.00	133.33	6.83
Zm00001d048113	833.33	0.60	333.33	0.30	500.00	0.50	153.85	1.30	125.00	1.30
Zm00001d048131	888.89	0.40	321.43	3.41	166.67	1.61	NA	0.00	NA	0.00
Zm00001d048192	1000.00	0.10	NA	0.00	625.00	0.30	NA	0.00	333.33	0.30
Zm00001d048234	333.33	0.30	913.04	0.30	363.64	0.70	1000.00	0.10	333.33	0.30
Zm00001d048451	862.07	0.27	333.33	0.91	257.15	1.69	108.39	4.16	111.11	4.09
Zm00001d048595	1000.00	0.10	960.00	0.20	NA	0.00	600.00	0.30	NA	0.00
Zm00001d048635	964.29	3.13	NA	0.00	666.67	6.85	500.00	1.73	424.25	3.64
Zm00001d048695	928.57	8.35	1000.00	0.10	666.67	14.07	500.00	9.43	333.33	0.63
Zm00001d048711	818.18	1.58	81.08	5.55	58.25	9.19	18.47	24.13	31.11	18.72
Zm00001d048991	818.18	0.50	500.00	0.30	285.71	0.70	500.00	0.20	NA	0.00
Zm00001d049145	1000.00	5.18	254.00	1.45	666.67	0.33	307.69	2.81	341.67	4.33
Zm00001d049228	964.29	0.20	400.00	0.40	352.94	0.60	375.00	0.40	333.33	9.55
Zm00001d049332	805.56	0.40	200.00	0.50	333.33	0.50	187.50	0.80	250.00	0.60
Zm00001d049400	291.67	3.67	888.89	0.40	416.67	1.94	285.71	7.61	142.86	2.67
Zm00001d049499	780.49	15.34	250.00	1.39	222.22	1.33	238.10	3.62	143.29	5.05
Zm00001d049585	250.00	0.40	1000.00	0.10	400.00	0.40	333.33	0.30	NA	0.00
Zm00001d049595	848.48	3.26	500.00	0.20	1000.00	0.25	294.12	3.24	500.00	5.05
Zm00001d050092	814.81	2.53	333.33	2.46	187.50	4.81	136.93	8.60	130.43	5.00

Gene	Max pausing score 0h	Gene coverage 0h	Max pausing score 0.5h	Gene coverage 0.5h	Max pausing score 1h	Gene coverage 1h	Max pausing score 2h	Gene coverage 2h	Max pausing score 4h	Gene coverage 4h
Zm00001d050141	925.93	0.20	NA	0.00	NA	0.00	NA	0.00	NA	0.00
Zm00001d050172	870.97	0.50	166.67	1.00	130.43	1.40	250.00	1.10	214.29	1.00
Zm00001d050294	875.00	0.30	NA	0.00	NA	0.00	500.00	0.20	NA	0.00
Zm00001d050308	1000.00	0.10	NA	0.00	500.00	0.30	1000.00	0.17	517.24	0.33
Zm00001d050335	761.90	0.98	965.52	1.26	350.00	2.87	325.63	6.60	250.00	2.67
Zm00001d050350	956.52	0.27	500.00	0.20	333.33	3.01	333.33	7.06	500.00	2.22
Zm00001d050498	NA	0.00	920.00	0.30	500.00	0.20	NA	0.00	NA	0.00
Zm00001d050558	793.10	0.60	400.00	0.40	666.67	0.20	NA	0.00	333.33	0.30
Zm00001d050830	965.52	0.20	750.00	0.50	500.00	0.30	714.29	0.30	NA	0.00
Zm00001d051140	333.33	0.30	1000.00	0.10	NA	0.00	NA	0.00	NA	0.00
Zm00001d051424	937.50	0.30	NA	0.00	NA	0.00	NA	0.00	NA	0.00
Zm00001d051474	666.67	0.20	1000.00	0.93	666.67	0.32	200.00	11.01	NA	0.00
Zm00001d051660	777.78	0.80	333.33	1.10	173.91	1.60	108.11	2.00	133.33	1.10
Zm00001d051672	810.81	0.70	454.55	0.60	192.31	1.40	162.16	1.90	166.67	1.30
Zm00001d051788	870.97	0.50	NA	0.00	333.33	0.30	437.50	0.80	500.00	0.20
Zm00001d052209	861.11	0.86	496.72	2.57	116.28	5.45	333.33	0.30	250.00	1.50
Zm00001d052212	875.00	0.40	466.67	0.30	142.86	0.20	90.91	0.30	NA	0.00
Zm00001d052239	827.59	1.50	294.12	2.00	100.00	3.48	125.00	2.92	233.77	2.36
Zm00001d052385	777.78	0.84	250.00	0.20	166.67	3.81	250.00	0.75	250.00	0.93
Zm00001d052471	898.34	1.06	600.00	0.20	333.33	1.18	250.00	2.09	200.00	2.20
Zm00001d052618	891.30	0.98	300.00	1.76	263.16	2.96	166.67	2.33	117.65	4.70
Zm00001d052701	785.71	2.76	333.33	0.64	130.43	2.79	103.65	4.65	214.29	1.21
Zm00001d052847	903.23	0.40	666.67	0.20	NA	0.00	500.00	0.20	400.00	0.30
Zm00001d052933	1000.00	0.10	500.00	0.27	500.00	2.33	359.15	6.75	500.00	0.20

Gene	Max pausing score 0h	Gene coverage 0h	Max pausing score 0.5h	Gene coverage 0.5h	Max pausing score 1h	Gene coverage 1h	Max pausing score 2h	Gene coverage 2h	Max pausing score 4h	Gene coverage 4h
Zm00001d052944	865.74	0.56	337.69	0.38	200.00	0.60	375.00	1.07	222.22	3.31
Zm00001d052981	862.07	1.66	285.71	0.83	416.67	1.58	1000.00	0.34	200.00	2.78
Zm00001d053017	777.78	2.18	250.00	0.40	200.00	3.39	120.00	7.41	266.67	4.94
Zm00001d053156	840.00	0.50	500.00	0.20	333.33	2.80	130.67	4.59	200.00	3.31
Zm00001d053244	958.33	0.20	900.00	0.30	1000.00	0.10	700.00	0.36	334.17	2.39
Zm00001d053306	846.15	1.23	426.13	10.71	260.70	2.89	157.95	12.55	400.00	1.98
Zm00001d053346	1000.00	0.10	NA	0.00	500.00	0.30	NA	0.00	400.00	0.30
Zm00001d053695	961.54	6.31	153.85	7.08	117.65	10.94	166.94	24.35	250.00	5.79
Zm00001d053826	875.00	0.86	111.11	7.25	101.62	8.59	133.33	8.40	250.00	6.25
Zm00001d054009	852.94	0.50	250.64	7.38	166.67	0.60	250.00	8.28	444.44	5.79
Zm00001d054071	913.04	12.20	NA	0.00	258.07	9.48	400.00	13.84	257.58	4.98

Note: NA, null-able values.
