# Supplementary material for: Ribosome Pausing Negatively Regulates Protein Translation in Maize Seedlings during Dark-to-Light Transitions
Source: Int J Mol Sci. 2024 Jul 22;25(14):7985. doi: 10.3390/ijms25147985 (PMC11277263; doi:10.3390/ijms25147985)
Supplement: Supplementary file 1 [file ijms-25-07985-s001.zip › Table S6.pdf]

Table S6 rDNA removing probes for ribo-seq library

Probe name	Probe sequence
Zea-rRNA-OUT-1	5'Biotin-TEG- AGCCCGCGTCAGCCTTTTATCTAATAAATGCGCCC
Zea-rRNA-OUT-2	5'Biotin-TEG- GGCGTGACGCCCAGGCAGACGTGCCCTCCGCCAG
Zea-rRNA-OUT-3	5'Biotin-TEG-CACTCTGCTGGGCCGACACTGACACTGAGAGACG
Zea-rRNA-OUT-4	5'Biotin-TEG-CAAGGTAGCCGTACTGGAAGGTGCGGCTGGATCACCT
Zea-rRNA-OUT-5	5'Biotin-TEG-AAATCGAGGCAAACTCTGAATACTAGATATGACCC
Zea-rRNA-OUT-6	5'Biotin-TEG-GAACGGGGCTAAGCGATCTGCCGAAGCTGTGGG
Zea-rRNA-OUT-7	5'Biotin-TEG-GTTGGTACGGAGGGACGGAGGAGGCTAGGTTAGC
Zea-rRNA-OUT-8	5'Biotin-TEG-TGTAACCCATGCCATACTCCCAGGAAAAGCTCG
Zea-rRNA-OUT-9	5'Biotin-TEG-TATTCTGGTGTCCTAGGCGTAGAGGAACCACACC
Zea-rRNA-OUT-10	5'Biotin-TEG-GGGGCGCATTTATTAGATAAAAGGCTGACGCGGGCT
Zea-rRNA-OUT-11	5'Biotin-TEG-ACGGCGAGAACCGACCGGCTCGACCCTTCTGCCGGC
Zea-rRNA-OUT-12	5'Biotin-TEG-CGAAAGATGGTTATAGGTTTAAGGACACAAGGTGACC
Zea-rRNA-OUT-13	5'Biotin-TEG-CACTCTGCTGGGCCGACACTGACACTGAGAGACGGAACGGGGCTAAGCGATCTGCCGAAGCTGTGGG
Zea-rRNA-OUT-14	5'Biotin-TEG-GAACGGGGCTAAGCGATCTGCCGAAGCTGTGGGAAATCGAGGCAAACTCTGAATACTAGATATGACCC
Zea-rRNA-OUT-15	5'Biotin-TEG-GTTGGTACGGAGGGACGGAGGAGGCTAGGTTAGCCGAAAGATGGTTATAGGTTTAAGGACACAAGGTGACCCTG
Zea-rRNA-OUT-16	5'Biotin-TEG-TGTAACCCATGCCATACTCCCAGGAAAAGCTCGAACGACC
Zea-rRNA-OUT-17	5'Biotin-TEG-AGGGCTATAGCTCAGTTCGGTAGAGCAACTCGTGCGCTCTTAGTTCAGTTTGGTAGAACGCGGGTC
Zea-rRNA-OUT-18	5'Biotin-TEG-ATTCTGGTGTCCTAGGCGTAGAGGAACCACACCAGTAACCCATGCCATACTCCCAGGAAAAGCTCGAACGACCT
