# Supplementary material for: Ribosome Pausing Negatively Regulates Protein Translation in Maize Seedlings during Dark-to-Light Transitions
Source: Int J Mol Sci. 2024 Jul 22;25(14):7985. doi: 10.3390/ijms25147985 (PMC11277263; doi:10.3390/ijms25147985)
Supplement: Supplementary file 1 [file ijms-25-07985-s001.zip › Table S7.pdf]

Table S7 Primers for Ribo-seq library amplification

Primer name	Primer sequence
RP1	5'-AATGATACGGCGACCACCGAGATCTACACGTTCAGAGTTCTACAGTCCGACG
RPI1	5'-CAAGCAGAAGACGGCATACGAGATCGTGATGTGACTGGAGTTCAGACGTGTGCTCTTCCGATCT
RPI2	5'-CAAGCAGAAGACGGCATACGAGATACATCGGTGACTGGAGTTCAGACGTGTGCTCTTCCGATCT
RPI3	5'-CAAGCAGAAGACGGCATACGAGATGCCTAAGTGACTGGAGTTCAGACGTGTGCTCTTCCGATCT
RPI4	5'-CAAGCAGAAGACGGCATACGAGATTGGTCAGTGACTGGAGTTCAGACGTGTGCTCTTCCGATCT
RPI5	5'-CAAGCAGAAGACGGCATACGAGATCACTGTGTGACTGGAGTTCAGACGTGTGCTCTTCCGATCT
RPI6	5'-CAAGCAGAAGACGGCATACGAGATATTGGCGTGACTGGAGTTCAGACGTGTGCTCTTCCGATCT
RPI7	5'-CAAGCAGAAGACGGCATACGAGATGATCTGGTGACTGGAGTTCAGACGTGTGCTCTTCCGATCT
RPI8	5'-CAAGCAGAAGACGGCATACGAGATTCAAGTGTGACTGGAGTTCAGACGTGTGCTCTTCCGATCT
RPI9	5'-CAAGCAGAAGACGGCATACGAGATCTGATCGTGACTGGAGTTCAGACGTGTGCTCTTCCGATCT
RPI10	5'-CAAGCAGAAGACGGCATACGAGATAAGCTAGTGACTGGAGTTCAGACGTGTGCTCTTCCGATCT
RPI11	5'-CAAGCAGAAGACGGCATACGAGATGTAGCCGTGACTGGAGTTCAGACGTGTGCTCTTCCGATCT
RPI12	5'-CAAGCAGAAGACGGCATACGAGATTACAAGGTGACTGGAGTTCAGACGTGTGCTCTTCCGATCT

Note : RP1 is the forward primer, and RPI1 to RPI12 are reverse primers with differnet indexes for sequencing.
