# Supplementary material for: Ribosome Pausing Negatively Regulates Protein Translation in Maize Seedlings during Dark-to-Light Transitions
Source: Int J Mol Sci. 2024 Jul 22;25(14):7985. doi: 10.3390/ijms25147985 (PMC11277263; doi:10.3390/ijms25147985)
Supplement: Supplementary file 1 [file ijms-25-07985-s001.zip › Table S8.pdf]

Table S8 Primers of the ROS genes used for qRT-PCR

Gene	Primer name	Primer sequence
Zm00001d002436	*AOX1a* F	5'-AGTCGCTGCGCTTCCCCA
	AOX1a R	5'-CGACGCGGTGCGCGAACT
Zm00001d039245	*WRKY6* F	5'-ATCCGTCGACAACAGCAAGG
	WRKY6 R	5'-CCTCGTAGGTAGTGATGAGGAT
Zm00001d012322	*CYP81D8* F	5'-GTGCGGCCAACCTGTGGA
	CYP81D8 R	5'-GTTAAGCAAGAGCGACATTGC
Zm00001d053838	*Ubi* F	5'-GTACCTGGGTATCTGCGTCG
	Ubi R	5'-CGAAGCCACAACCACGATTC
